# Cannabidiol's Upregulation of *N*-acyl Ethanolamines in the Central Nervous System Requires *N*-acyl Phosphatidyl Ethanolamine-Specific Phospholipase D

**DOI:** 10.1089/can.2018.0031

**Published:** 2018-11-30

**Authors:** Emma Leishman, Meera Manchanda, Rachel Thelen, Sally Miller, Ken Mackie, Heather B. Bradshaw

**Affiliations:** ^1^ Program in Neuroscience, Indiana University Bloomington, Bloomington, Indiana.; ^2^ Department of Psychological and Brain Sciences, Indiana University Bloomington, Bloomington, Indiana.; ^3^ Gill Center for Biomolecular Science, Indiana University Bloomington, Bloomington, Indiana.

**Keywords:** lipidomics, CBD, *N*-acyl ethanolamine, NAPE-PLD, THC, FAAH

## Abstract

**Introduction:** Δ^9^-tetrahydrocannabinol (THC) and cannabidiol (CBD) are bioactive cannabinoids. We recently showed that acute THC administration drives region-dependent changes in the mouse brain lipidome. This study tested the hypothesis that cell lines representing cell types present in the central nervous system (CNS), neurons (N18 cells), astrocytes (C6 glioma cells), and microglia (BV2 cells) would respond differently to THC, CBD, or their combination. This experimental strategy also allowed us to test the hypothesis that THC and CBD are metabolized differently if presented in combination and to test the hypothesis that responses to CBD are not like the fatty acid amide hydrolase (FAAH) inhibitor URB597. Finally, we tested the hypothesis that CBD's CNS effects would differ in the *N*-acyl phosphatidyl ethanolamine-specific phospholipase D (NAPE-PLD) knockout (KO) compared to wild-type (WT) mice.

**Methods:** N18, C6, and BV2 cells were stimulated with 1 μM THC, 1 μM CBD, 1 μM THC:CBD, 1 μM URB597, or vehicle for 2 h and lipids extracted. Adult female WT and NAPE-PLD KO mice were injected with 3 mg/kg CBD or vehicle i.p., brains collected 2 h later, eight brain regions dissected, and lipids extracted. Extracted lipids were characterized and quantified using high-pressure liquid chromatography coupled with tandem mass spectrometry (HPLC/MS/MS).

**Results:** Lipid levels in each cell type were differentially affected by THC, CBD, or THC:CBD with a few exceptions. In all cell lines, THC increased levels of arachidonic acid and CBD increased levels of *N*-acyl ethanolamines (NAEs), including *N*-arachidonoyl ethanolamine. More THC remained when cells were coincubated with CBD; however, levels of THC metabolites were cell-type dependent. CBD and URB597 caused very different lipid profiles in the cell-based assays with the primary similarity being increases in NAEs. CBD increased levels of NAEs in the WT hippocampus, cerebellum, thalamus, cortex, midbrain, and brainstem; however, NAEs *did not* increase in any brain region after CBD in NAPE-PLD KO mice.

**Conclusions:** CBD and THC differentially modify the lipidome of the brain and CNS-type cell lines. Increases in NAEs observed after CBD treatment had previously been attributed to FAAH inhibition; however, data here suggest the alternative hypothesis that CBD is activating NAPE-PLD to increase NAE levels.

## Introduction

Δ^9^-tetrahydrocannabinol (THC) and cannabidiol (CBD) are cannabinoids that were isolated from *Cannabis* in the 1960s.^1,2^ THC is mainly responsible for the “high” associated with *Cannabis* primarily through activity at the CB_1_ receptor.^3,4^ CBD is considered the “non-psychoactive” cannabinoid in that ingestion does not produce the characteristic euphoria or abuse potential associated with THC.^5,6^ However, emerging evidence demonstrates that CBD has therapeutic benefits for central nervous system (CNS) disorders, including schizophrenia^7,8^ and childhood epilepsy.^9,10^

We recently showed that acute THC drives significant changes in the brain lipidome,^11^ including but not limited to the endogenous cannabinoid (eCB) ligands. The eCB ligands, *N*-arachidonoyl ethanolamine (AEA)^12^ and 2-arachidonoyl glycerol (2-AG),^13,14^ are derived from arachidonic acid (AA) and belong to larger families of signaling lipids called lipoamines and 2-acyl glycerols, respectively. Collectively, these 2-acyl glycerols, lipoamines, their parent fatty acids, and the AA-derived prostaglandins (PGs) form a wider lipid signaling network, regulated in part by eCB system enzymes^15–17^ and altered in various disease models.^18–20^ Our data showed that in adult mice treated with THC, levels of AEA, 2-AG, PGs, and related lipids are significantly decreased across most of the brain, suggesting an effect of THC that goes beyond traditional CB_1_ activity.^11^

In this study, we examined how CBD treatment alters the brain lipidome and finds very different results compared to THC treatment. Given that the brain areas analyzed in both the THC and CBD lipidomics experiments are heterogeneous mixtures of cell types, we additionally sought to test the hypothesis that THC, CBD, and their combination would drive differential changes in the lipidome of specific cell types. To do so, we used cell line proxies for CNS-derived neurons (N18 cells), astrocytes (C6 glioma cells), and microglia (BV2 cells). BV2 cells possess CB_2_ receptors, but not CB_1_ receptors,^21^ and THC alters cytokine production in these cells.^22^ C6 glioma can express both CB_1_ and CB_2_,^23,24^ and the N18 cell line expresses CB_1_.^25–27^ Using these cell-based models for lipidomics analysis provides complementary and simplified biochemical systems to study the effects of THC, CBD, and their combination on the lipidome and on local THC metabolism. These cell lines also allowed us to test the popular hypothesis that CBD functions as a fatty acid amide hydrolase (FAAH) inhibitor. In a final experiment, we test the hypothesis that CBD acts through *N*-acyl phosphatidyl ethanolamine-specific phospholipase D (NAPE-PLD) to drive the changes in *N*-acyl ethanolamines (NAEs).

## Methods

### Mice and drug injections, tissue collection, and lipid extraction

The Bloomington Institutional Animal Care and Use Committee of Indiana University approved the procedures used here, which comply with Animal Research: Reporting of *In Vivo* Experiments (ARRIVE) guidelines.^28^ These are international guidelines developed by scientists with the goal of improving the reporting of data obtained from animal studies and to minimize unnecessary studies. Female adult (3–7 months) C57 wild-type (WT) and NAPE-PLD knockout (KO)^17^ mice were given a single i.p. injection of 3 mg/kg CBD (*n*=9 for WT, 8 for KO) or vehicle (*n*=8 per genotype). Two hours later, mice were sacrificed. Brains were removed and dissected into these regions: striatum (STR), hippocampus (HIPP), cerebellum (CER), thalamus (THAL), cortex (CTX), hypothalamus (HYP), midbrain (MID), and brainstem (STEM).^19^ Methanolic extracts from each of the eight brain areas were partially purified as previously described.^11,15–19,29–31^

### Cell culture, stimulation with drugs or vehicle, and cell lipid extraction

Cells were grown under standard cell culture conditions.^18^ Upon reaching ∼75% confluence in T-25 cm^2^ (C6s) or T-75 cm^2^ flasks (BV2s and N18s), sets of 12 flasks were stimulated with 1 μM THC, CBD, THC:CBD (all cell lines), or URB597 (BV2s only). Six flasks received drug-supplemented growth media, and six received vehicle (1:1:18 cremophor:ethanol:saline). After 2 h incubation, media was replaced with 4 mL high-pressure liquid chromatography (HPLC)-grade methanol (Thermo Fisher Scientific, Waltham, MA). Cells were scraped from their growth surface and transferred with the methanol to a centrifuge tube. Solutions were spiked with 500 picomoles deuterium-labeled *N*-arachidonoyl glycine (d_8_NAGly; Cayman Chemical, Ann Arbor, MI) or deuterium-labeled AEA (d_8_AEA; Cayman Chemical) to determine extraction efficiency and were centrifuged at 3,000 rpm for 15 min at 24°C. Supernatants were diluted with HPLC water (purified in house) to make a 15% supernatant solution. Lipid extractions were performed as previously described using C18 solid phase extraction columns (Agilent, Palo Alto, CA).^11,15–18,29–31^ Briefly, columns were conditioned with 5 mL HPLC methanol followed by 2.5 mL HPLC water. Then, the supernatant/water solution was loaded onto the column. Impurities were washed off with 2.5 mL HPLC water. A series of five elutions with 1.5 mL 40%, 60%, 75%, 85%, and 100% methanol were collected. The 12 flasks from each experiment were processed together; experiments took place on different days.

### HPLC coupled with tandem mass spectrometry

As previously described,^11,15–19,29–31^ extracts were analyzed using an Applied Biosystems API 3000 triple quadrupole mass spectrometer (Foster City, CA). Twenty microliters from each elution was chromatographed using a 2.1×50 mm XDB-C18 reversed phase HPLC analytical column with a 3.5 μm particle size (Agilent) using optimized mobile phase ingredients (Supplementary Methods). Two Shimadzu 10ADvp pumps (Columbia, MD) provided pressure for gradient elution. Analysis of the HPLC coupled with tandem mass spectrometry (HPLC/MS/MS) data was performed using Analyst software (Applied Biosystems).^11,15–19,29–31^ Chromatograms (Supplementary Fig. S1) displaying the retention time of analytes matching programmed parent and fragment ion masses (Supplementary Fig. S2) were generated by running each sample using a multiple reactions monitoring method. Retention times were then compared to those from standards for the suspected compound. If retention times matched, then concentrations were determined by calculating the area under the curve for the unknown and comparing it to the calibration curve obtained from the standards. Extraction efficiency was calculated using a recovery vial spiked with 500 pmol d_8_AEA or d_8_NAGly as a standard, and analyte levels were adjusted for extraction efficiency. d_8_AEA and d_8_NAGly produce similar recovery values.^32^ For each individual lipid in each experiment, concentrations from the drug-treated cells or brain areas were compared to vehicle using a one-way analysis of variance in SPSS Statistics (IBM, Armonk, NY). Statistical significance was defined as *p*<0.05 and *p*<0.10.

A series of calculations were performed to generate the figures in the Results section; please refer to Supplementary Methods for more information.

## Results

### Signal detection and overall effects in CBD-treated WT mice, THC and CBD-treated BV2, C6, and N18 cells, and CBD-treated NAPE-PLD KO mice

The HPLC/MS/MS screening library contains 85 lipids (Supplementary Fig. S2), and over 50 were detected in each experiment. The specific lipids affected varied by drug, cell line, and KO status (Supplementary Figs. S3–S10). The full list of analyte levels and statistical analyses is in Supplementary Tables S1–S64. The focus of the results here will be on a selected subset of this lipidome: the NAEs, AA, AA-derived lipoamines, 2-AG, and PGs.

### CBD increases levels of NAEs and many other lipoamines across the WT mouse brain

**FIG. 1.**
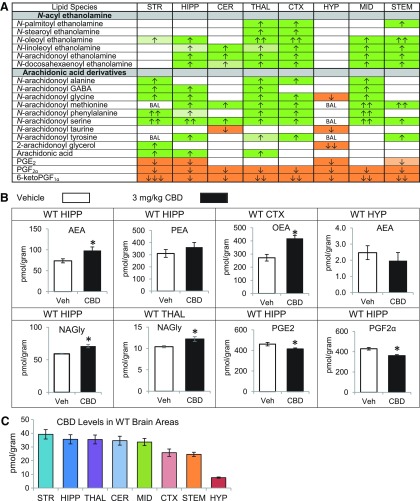
Effects of systemic 3 mg/kg CBD on levels of six different NAE lipids, targeted AA-derived lipoamines, 2-AG, free AA, and PGs 2 h after injection in the adult C57 female mouse striatum (STR), hippocampus (HIPP), cerebellum (CER), thalamus (THAL), cortex (CTX), hypothalamus (HYP), midbrain (MID), and brainstem (STEM) and levels of CBD in each region. **(A)** Cells with shaded arrows indicate a change for that lipid in the CBD-exposed brain area relative to the same vehicle-exposed area. The arrow color indicates the direction of a significant result relative to control. Green colors represent increases, with darker green representing a significant (*p*<0.05) increase and lighter green representing a trending (*p*<0.10) increase. Orange colors represent decreases, with darker orange indicating a significant (*p*<0.05) decrease and light orange representing a trending (*p*<0.10) decrease. The number of arrows indicates the magnitude of the difference. One arrow indicates a magnitude difference of less than 1.5-fold, two arrows indicates a 1.5–1.99-fold change, and three arrows indicate a 2–2.99-fold change. BAL stands for “below analytical limit,” whereas a blank cell indicates that there was no change in the lipid's level. See Supplementary Methods and Supplementary Figure S3 for more detailed description of analysis. **(B)** Bar graphs showing mean levels of AEA in the WT HIPP, PEA in the WT HIPP, OEA in the WT cortex (CTX), AEA in the WT hypothalamus (HYP), NAGly in the WT HIPP, and prostaglandins PGE_2_ and PGF_2α_ in the WT HIPP 2 h after a vehicle injection (open bars) or a 3 mg/kg CBD injection (black bars). The units on the y-axis are moles of lipid per gram of tissue. Error bars are±standard error. “*” represents a difference of *p*<0.05 between CBD and vehicle groups. In the WT HIPP, levels of AEA increased (corresponding to a darker green cell with one up arrow in **A**), whereas levels of PEA showed no change in this region (corresponding to a blank cell in **A**). Levels of OEA increased in the CTX (two up arrows in a darker green cell in **A**). In the HYP, there was no significant difference in AEA. Levels of NAGly were significantly higher in the CBD-exposed HIPP (corresponding to a darker green cell with one up arrow in **A**). In addition in the HIPP, levels of PGE_2_ and PGF_2α_ decreased (corresponding to darker orange cells with one down arrow). **(C)** Levels of CBD in eight brain regions of female mice 2 h after an acute 3 mg/kg CBD injection. Units on the y-axis are moles of CBD per gram of tissue. Error bars are±standard error. Brain areas are ordered to correspond with their CBD levels, with the area having the highest concentration shown on the left and the area with the lowest on the furthest right. 2-AG, 2-arachidonoyl glycerol; AA, arachidonic acid; AEA, *N*-arachidonoyl ethanolamine; CBD, cannabidiol; NAE, *N*-acyl ethanolamine; NAGly, *N*-arachidonoyl glycine; OEA, *N*-oleoyl ethanolamine; PEA, *N*-palmitoyl ethanolamine; PG, prostaglandin; WT HIPP, wild-type hippocampus.

In WT mice, CBD treatment increased AEA and at least one and up to five additional NAEs in the hippocampus (HIPP), cerebellum (CER), thalamus (THAL), cortex (CTX), midbrain (MID), and brainstem (STEM). In every region except the hypothalamus (HYP), CBD increased multiple AA-derived lipoamines, including *N*-arachidonoyl glycine (NAGly), which increased in five regions: striatum (STR), HIPP, THAL, CTX, and MID. The STR was the only region with elevated NAGly without a concurrent increase in AEA. NAGly decreased in the HYP, while AEA was unchanged. The only other decreases in an AA-derived lipoamine were reductions in *N*-arachidonoyl taurine (A-Taur) in the CER and HYP. Similarly, 2-AG changed in a region-dependent manner, increasing in the STR, while decreasing in the HYP. AA levels significantly increased in the STR, HIPP, and THAL. PGF_2α_ and 6-ketoPGF_1α_ were reduced in all eight regions examined, and PGE_2_ was reduced in four regions (Fig. 1A, 1B; Supplementary Fig. S4). Levels of CBD were relatively consistent across brain regions, with only CTX and STEM having ∼25% less than other regions with the exception of the HYP, which had ∼10-fold less CBD (Fig. 1C; Supplementary Tables S59–S61).

### Effects of THC on endogenous lipids in BV2, C6, and N18 cell lines

**FIG. 2.**
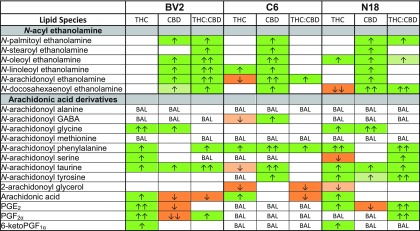
Comparison of significant effects of 1 μM Δ^9^-tetrahydrocannabinol (THC) stimulation, 1 μM CBD stimulation, and combined 1 μM Δ^9^-tetrahydrocannabinol (THC) and CBD stimulation for 2 h on levels of six different NAE lipids, targeted AA-derived lipoamines, 2-AG, free AA, and PGs between BV2, C6, and N18 cell lines. The color of the cell indicates the direction of change of a lipid's concentration with drug relative to vehicle: orange is a decrease and green is an increase. Darker colors indicate *p*<0.05, whereas lighter colors indicate *p*<0.10. The number of arrows represents the magnitude of the change. One arrow=1–1.5-fold change; two arrows=1.5–2-fold change. See Supplementary Methods and Supplementary Figure S3 for more details.

In adult mice, a 2-h THC treatment caused significant decreases in NAEs, including AEA, some additional lipoamines, 2-AG, and PGs, in many of the brain regions examined.^11^ In this study, AEA was significantly decreased and *N*-linoleoyl ethanolamine increased in C6s, whereas *N*-docosahexaenoyl ethanolamine (DEA) was decreased and *N*-oleoyl ethanolamine (OEA) increased in N18s. THC caused no changes in NAEs in BV2s. Overall, different modulations in lipids were measured in each cell type with the exception that *N*-arachidonoyl phenylalanine (A-Phe) and AA were increased in all cell lines after THC treatment. The responses of BV2 and N18 cells were most similar with shared increases in NAGly, A-Taur, PGE_2_, and 6-ketoPGF_1α_, whereas 2-AG decreased in both C6 and N18 cells (Fig. 2, Supplementary Figs. S5–S7).

### Effects of CBD on endogenous lipids in BV2, C6, and N18 cell lines

Similar to the results from brain tissue, CBD increased NAEs in all three cell lines. Unlike results from brain, A-Taur increased across cell lines. Responses of the BV2 and N18 cells were most similar, with NAGly increasing and PGE_2_ decreasing. In contrast to THC's effects on these cell lines, AA and PGs decreased with CBD exposure, whereas 2-AG was unaffected in all three cell lines (Fig. 2, Supplementary Figs. S5–S7).

### Effects of THC:CBD on endogenous lipids in BV2, C6, and N18 cell lines

Except for an increase in A-Phe in all three cell types, treatment with THC:CBD generated a different lipidomic phenotype than the individual drugs. BV2s were the only cells with significant increases in all NAEs, wherein THC:CBD increased NAEs more than CBD alone. C6s only had an increase in AEA, and N18s had modest increases in DEA, *N*-palmitoyl ethanolamine, and OEA. Some of the THC:CBD results were a mosaic of the individual drug results. For example, in BV2s in the THC:CBD group, AA decreased (as with CBD), but PGF_2α_ increased (as with THC). Likewise, in C6s given THC:CBD, 2-AG decreased (as with THC), but AEA increased (as with CBD). With THC:CBD in N18s DEA increased (as with CBD), which was the opposite with THC alone where it decreased. These specific data add to the hypothesis that the THC:CBD combination drives a third phenotype and not just a combination of responses (Fig. 2, Supplementary Figs. S5–S7).

### THC and metabolite levels in BV2, C6, and N18 cells

**FIG. 3.**
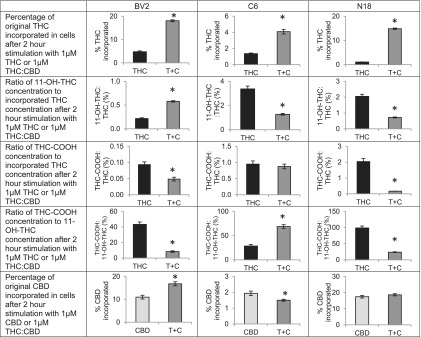
Effects of combining Δ^9^-tetrahydrocannabinol (THC) and CBD on THC and CBD incorporation and THC metabolism in BV2, C6, and N18 cells. BV2, C6, and N18 cells were either stimulated with 1 μM THC (black bars) or 1 μM THC:CBD (dark gray bars) and the proportion of THC added to each flask that remained incorporated in the cells after the 2 h stimulation was calculated, averaged, and compared between the single treatment and the combined treatment. Graphs comparing these percentages of incorporated THC are shown on the first row of Figure 3. The y axis is expressed as the mean percentage of drug incorporated, and error bars are±standard error. “*” indicates a significant difference between groups. For all three cell lines, levels of THC incorporated increased when combined with CBD. The ratio of concentrations of incorporated THC metabolites, 11-OH-THC and THC-COOH, to concentrations of incorporated THC at 2 h was calculated and shown in rows 2 and 3, and the ratio of incorporated THC-COOH concentration to incorporated 11-OH-THC concentration is shown in row 4. Bar graphs are these average ratios expressed as a percentage, and the error bars are±standard error. “*” indicates a significant difference between groups of *p*<0.05. The ratio of 11-OH-THC to THC was higher in BV2 cells but lower in C6 and N18 cells when combined with CBD. The ratio of THC-COOH to THC was lower in BV2 and N18 cells but did not significantly differ in C6 cells. The ratio of THC-COOH to 11-OH-THC was lower in BV2 and N18 cells and was higher in C6 cells when THC and CBD were coadministered. The bottom row of figure examines the percentage of CBD incorporated in cells. BV2, C6, and N18 cells were either stimulated 1 μM CBD (light gray bars) or 1 μM THC:CBD (dark gray bars) and the proportion of CBD added to each flask that remained incorporated in the cells after the 2 h stimulation was calculated, averaged, and compared between the single treatment and the combined treatment. The y axis is expressed as the mean percentage of drug incorporated, and error bars are±standard error. “*” indicates a significant difference between groups. The effects of the combination treatment on levels of incorporated CBD at 2 h varied by cell line. See Supplementary Methods for more detail on how values in figure were calculated.

One hypothesis of how the THC:CBD combination generates a different phenotype is that the metabolism of each of the cannabinoids is regulated at least, in part, by the same CYP enzymes.^33,34^ An explanation for this is that the rate of metabolism of one (e.g., THC) would be modified in the presence of the other (e.g., CBD).^35,36^ In this study, we measured the amount of THC, two THC metabolites, and CBD that remained in the cell after the 2-h incubation with the different treatment strategies. In all cell types, we show that the percentage of THC incorporated and remaining into cells after 2 h was significantly higher in the presence of CBD; however, the levels incorporating and remaining of CBD were cell-line dependent. Likewise, levels of THC metabolites also changed in a cell-dependent manner. Thus, elevated THC when combined with CBD cannot be attributed to a decrease in these THC metabolites alone (Fig. 3).

### Effects of URB597 on lipid levels in BV2 microglia

**FIG. 4.**
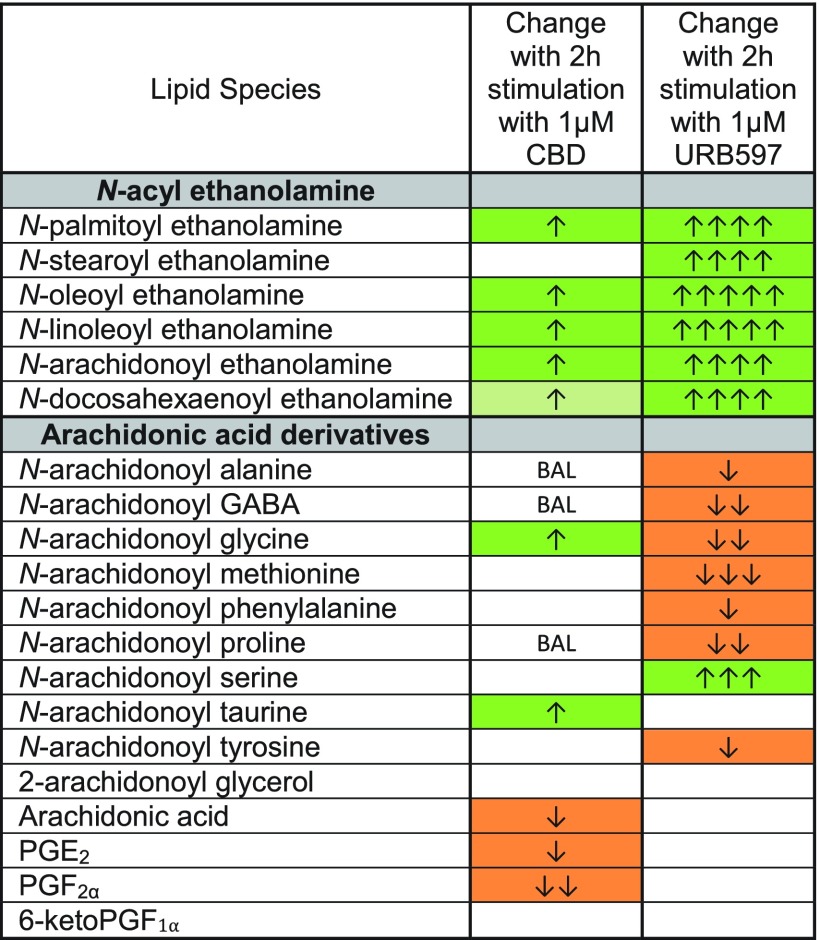
Comparison of effects of 2 h stimulation with 1 μM CBD and 2 h stimulation with 1 μM URB597 on levels of NAEs, targeted AA-derived lipoamines, 2-AG, free AA, and PGs in BV2 microglia. Cells with shaded arrows indicate a change for that lipid in the drug-exposed BV2 cells relative to vehicle-exposed BV2 cells. The arrow color indicates the direction of a significant result relative to vehicle. Green colors represent increases, with darker green representing a significant (*p*<0.05) increase and lighter green representing a trending (*p*<0.10) increase. Orange color indicates a significant (*p*<0.05) decrease. The number of arrows indicates the magnitude of the difference. One arrow indicates a magnitude difference of less than 1.5-fold, two arrows indicate a 1.5–1.99-fold change, three arrows indicate a 2–2.99-fold change, four arrows indicate a 3–9.99-fold change, and five arrows indicate a fold change greater than 10. BAL stands for “Below Analytical Limit,” whereas a blank cell indicates that there was no change in the lipid's level. See Supplementary Methods and Supplementary Figure S3 for more detailed description of analysis. Levels of five of the six NAEs measured increased with CBD, whereas all six increased with URB597. The increases were of a larger magnitude when cells were given URB597 compared to CBD. CBD increased levels of three AA-derived lipoamines and decreased AA and PG levels. In contrast, URB597 decreased levels of seven different arachidonic-acid derived lipoamines and did not affect AA or PG levels. The increase in AEA was the only change in an AA-derived lipid common to both CBD and URB597.

The increases in NAEs after CBD treatment appear to support the hypothesis that CBD is acting as a FAAH inhibitor; however, additional lipidomics data following CBD treatment do not align with FAAH KO and FAAH inhibition profiles.^16,32^ In this study, we show a direct comparison of the BV2 lipid profiles after CBD or URB597 treatment. More lipids changed in response to URB597 than CBD, URB597 caused a higher proportion of decreases, and URB597 increased NAEs with a greater magnitude than CBD. Importantly, there were several key differences between the effects of CBD and URB597, especially in lipids derived from AA. Under CBD stimulation, AA-derived lipoamines either did not change or were upregulated (AEA, NAGly, A-Taur); however, with URB597, six AA-derived lipoamines decreased. Furthermore, URB597 did not affect AA or PG levels, whereas CBD caused decreases in AA and PGs (Fig. 4, Supplementary Figs. S5, S6, S8).

### Effects of acute CBD on NAPE-PLD KO mice

**FIG. 5.**
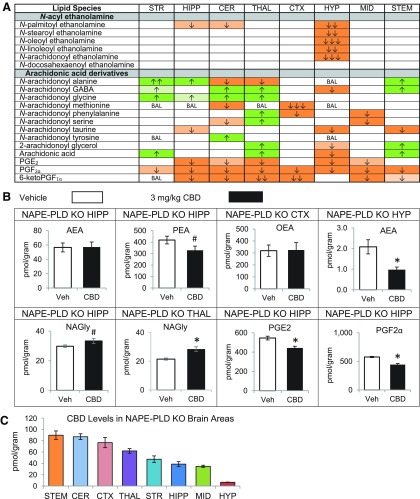
Effects of systemic 3 mg/kg CBD on levels of six different NAE lipids, targeted AA-derived lipoamines, 2-AG, free AA, and PGs 2 h after injection in the NAPE-PLD KO female mouse striatum (STR), hippocampus (HIPP), cerebellum (CER), thalamus (THAL), cortex (CTX), hypothalamus (HYP), midbrain (MID), and brainstem (STEM). **(A)** Cells with shaded arrows indicate a change for that lipid in the CBD-exposed brain area relative to the same vehicle-exposed area in NAPE-PLD KO. The arrow color indicates the direction of a significant result relative to control. Green colors represent increases, with darker green representing a significant (*p*<0.05) increase and lighter green representing a trending (*p*<0.10) increase. Orange colors represent decreases, with darker orange indicating a significant (*p*<0.05) decrease and light orange representing a trending (*p*<0.10) decrease. The number of arrows indicates the magnitude of the difference between CBD and vehicle. One arrow indicates a magnitude difference of less than 1.5-fold, two arrows indicate a 1.5–1.99-fold change, and three arrows indicate a 2–2.99-fold change. BAL stands for “Below Analytical Limit,” whereas a blank cell indicates that there was no change in the lipid's level due to CBD. See Supplementary Methods and Supplementary Figure S3 for more detailed description of analysis. **(B)** Bar graphs showing mean levels of AEA and PEA in the NAPE-PLD KO hippocampus (HIPP), OEA in the NAPE-PLD KO cortex (CTX), AEA in the NAPE-PLD KO hypothalamus (HYP), NAGly in the NAPE-PLD KO thalamus (THAL), and prostaglandins PGE_2_ and PGF_2α_ in the NAPE-PLD KO HIPP 2 h after a vehicle injection (open bars) or a 3 mg/kg CBD injection (black bars). The units on the y-axis are moles of lipid per gram of tissue. Error bars are±standard error. “*” represents a difference of *p*<0.05 between CBD and vehicle groups, and “#” represents a difference of *p*<0.10 between CBD and vehicle groups. There are no differences in AEA for the NAPE-PLD KO HIPP (blank cell in **A**). Levels of PEA showed a trending decrease in the NAPE-PLD KO HIPP (corresponding to a lighter orange cell with one down arrow in **A**). Levels of OEA did not change in the CTX of NAPE-PLD KO animals. In the HYP, there was a large decrease in AEA levels (corresponding to three down arrows in a darker orange cell in **A**). Levels of NAGly were significantly higher in the CBD-exposed THAL (corresponding to darker green cells with one up arrow in **A**). In the HIPP, levels of PGE_2_ and PGF_2α_ decreased with CBD treatment (corresponding to darker orange cells with one down arrow). **(C)** Levels of CBD in eight brain regions of NAPE-PLD KO female mice 2 h after an acute 3 mg/kg CBD injection. Units on the y-axis are moles of CBD per gram of tissue. Error bars are±standard error. Brain areas are shown on the x-axis. In each graph, brain areas are ordered to correspond with their levels of CBD, with the area having the highest concentration shown on the left and the area with the lowest on the furthest right. KO, knockout; NAPE-PLD, *N*-acyl phosphatidyl ethanolamine-specific phospholipase D.

CBD treatment of NAPE-PLD KO mice did not increase NAEs in any brain region analyzed. In contrast, alterations in AA-derived lipoamines in the NAPE-PLD KO brain were widespread. NAGly was the only AA-derived lipoamine for which all the detected changes were increases. 2-AG increased in the THAL and STEM, and AA increased in the STR, STEM, and THAL, but 2-AG and AA decreased in the HYP. Widespread downregulation of PGs was also measured, including reduced PGF_2α_ in all eight areas (Fig. 5A, B; Supplementary Fig. S9). Notably, the increases in NAGly and decreases in PGs were also observed in the WT brains after CBD treatment (Figs. 1 and 5; Supplementary Figs. S4 and S9). More so than in the WT mice, levels of CBD varied by brain region. Levels of CBD were still significantly lower in HYP than any other brain region as was observed in the WT; however, levels in STEM were among the highest and levels in STR were among the lowest, which was the opposite in the WT mice (Fig. 5C; Supplementary Tables S62–S64). This may suggest a role of NAPE-PLD in CBD transport or metabolism.

## Discussion

### Distinct lipidomic profiles after cannabinoid treatment in different cell types may be influenced by cannabinoid metabolism

The nine different lipid profiles resulting from the three cannabinoid treatments across three cell types emphasize the complexity of the interactions between cannabinoids and lipid signaling. While individual changes in lipids are important, discussion of each of the specific changes and what they mean for cellular signaling is largely speculative. Why they are distinctive and why the combination of THC and CBD is not simply an additive phenotype are likely due to these compounds' interactions at the cellular level. As each cell line expresses eCB system genes at different levels,^37^ signal transduction may differ between cell lines stimulated with cannabinoids. CBD and THC may also compete for the same intracellular carriers, such as fatty acid binding proteins, which also shuttle eCBs in the cell.^38^ Differences in metabolism might also underlie the distinctive effects of THC and CBD and contribute to why their combination has emergent effects on the lipidome. The conversion of THC to 11-OH-THC and (±)-11-nor-9-carboxyTHC is carried out by the cytochrome P450 family of enzymes, mainly CYP2C9^39^ and CYP3A4.^33^ Uridine 5′-diphospho-glucuronosyltransferases (UGTs) then conjugate these metabolites with glucuronic acid before excretion.^40^ CBD is also metabolized by P450 and UGT enzymes, but much less is known about the metabolic fate of CBD.^41^ CYP450 enzymes also metabolize AA and other endogenous lipids,^42,43^ meaning that the effects of THC and CBD on these enzymes might contribute to effects on the lipidome.

Most of the metabolism of THC is thought to take place in the liver,^35^ where expression of P450 enzymes and UGTs is high; however, these enzymes are also expressed in the brain suggesting that local metabolism is also likely.^40,42–45^ Providing evidence of extrahepatic THC metabolism, we detected THC metabolites in all cell lines after THC treatment. These data demonstrate that some of the effects on the lipidome attributed to THC may also be due to 11-OH-THC, as this metabolite is cannabimimetic.^46^ To test this hypothesis, future studies could measure the effect of 11-OH-THC on lipid levels. It is also possible that CBD metabolites such as 7-OH-CBD and 7-COOH-CBD are bioactive.^41^ However, we do not currently have these CBD metabolites in the screening library.

The surprising variability of THC and CBD integration and THC metabolite ratios across cell types also adds to the complexity of the responses. Potentially contributing to the variability, expression of CYP450 enzymes differs by cell type in the CNS,^37^ and CYP450 expression is highly dynamic.^42,43^ For example, astrocytes tend to express a different set of CYP450 enzymes, which is relevant to responses to circulating drugs because astrocyte end-feet cover cerebral microvasculature.^43^ Given that astrocytic CYP450s metabolize AA and related lipids,^43^ the differential expression of CYP450 enzymes in astrocytes may explain why the responses of N18 and BV2 cells were more similar in terms of changes in lipid levels. Unique responses to CBD in the hypothalamus could be due to specialized astrocytes that maintain hormonal and metabolic homeostasis.^47^ These astrocytes may also be contributing to the lower levels of CBD found in this region 2 h after the acute injection by preventing the entry of CBD into this area. Low levels of CBD might explain why CBD failed to upregulate AA-derived lipids in the HYP. These data suggest that the cell composition within CNS regions will ultimately dictate the extent of THC and CBD metabolism and, therefore, signaling properties.

### How does CBD activity differ from FAAH inhibition?

FAAH is a ubiquitous enzyme hypothesized to be responsible for the majority of AEA hydrolysis. Several studies reported that CBD inhibits FAAH at IC_50_ values of ∼10–20 μM, with a primary outcome of an increase in AEA and other NAEs.^5,7,38,48–52^ As the mouse brain^37^ and all three cell lines express FAAH,^53–55^ a potential explanation for the CBD-driven increases in NAEs could be FAAH inhibition. However, we recently showed that many lipoamines decrease in the broader lipidome in the FAAH KO mouse, supporting a more complex lipid fingerprint for FAAH inhibition than just increases in NAEs.^16^ These mass spectrometric techniques allow a unique view into lipid production and regulation. In earlier work we demonstrated that NAGly significantly decreased in the rat striatum when FAAH was inhibited with URB597, suggesting that FAAH also participates in synthesis of endogenous lipids and is not solely a degradative enzyme for NAEs.^56,57^ In those studies, it was also revealed that AEA was a direct precursor for NAGly biosynthesis and that FAAH was a rate-limiting enzyme in NAGly production.^56^ Those data provided evidence that AA may not be “released” as much as it is being rapidly conjugated with glycine and potentially additional amino acids. More recent evidence using broader-scale lipidomics in FAAH KO mice showed that the majority of AA-derived lipoamines are significantly reduced in the CNS and that AA is unaffected.^16^ These data are consistent with a lipidomics study from the Barker Lab examining the consequences of pharmacological FAAH blockade with URB597 on AA-derived signaling molecules in mouse brain wherein the authors, likewise, showed reductions in these AA-derived lipoamines.^32^

In this study, we showed that the FAAH inhibitor URB597 and CBD produced very different results in our lipidomics panel in BV2 cells. Although both treatments elevated NAE levels, the effects deviated regarding AA-derived lipoamines. For example, levels of NAGly increased in BV2 cells exposed to CBD but decreased, along with six additional AA-derived lipoamines, in BV2 cells when exposed to URB597. Adding to evidence that the effects of CBD and FAAH inhibition on lipid levels are divergent, no changes in AA or PGE_2_ were seen throughout in the FAAH KO brain^16^ or in cells treated with URB597 here, suggesting that AA liberated by FAAH does not contribute to steady-state levels of AA or PGE_2_. In contrast, monoacylglycerol lipase (MAGL)-catalyzed hydrolysis of 2-AG maintains brain levels of AA and PGs, as evidenced by decreased AA and PGs in MAGL KO mice.^16,58^ This suggests that the AA released from 2-AG's hydrolysis through MAGL is an important substrate for PGs.^16,58^ It is possible that AA released by FAAH's hydrolysis of AEA is rapidly conjugated to form a lipoamine before it can be measured, which would explain why levels of AA-derived lipoamines decrease when FAAH is blocked without affecting AA levels.^16,32,56,57^ In contrast to FAAH inhibition, CBD reliably reduced PG levels and often modulated AA levels. Given that the effects of CBD and FAAH inhibition on the lipidome diverged, we hypothesized that CBD is acting at an alternative site to FAAH to increase NAEs. To further examine whether CBD is acting as a FAAH inhibitor, follow-up studies should more directly examine the effects of CBD on FAAH expression in CNS tissues and CNS-derived cell lines using RT-PCR.

### CBD as a regulator of NAPE-PLD

There are several other pathways that can influence NAE levels that haven't been investigated in the context of CBD. Recently, we confirmed that NAPE-PLD is an important enzyme in regulating levels of NAEs, including AEA, in the mouse brain.^17^ In this study, we show that CBD failed to increase NAE levels in NAPE-PLD KO mice, suggesting that NAPE-PLD is required for CBD's ability to elevate NAEs. There are data showing the modulation of NAPE-PLD activity in BV2,^59^ C6,^60^ and N18 cells,^26^ supporting that NAPE-PLD is expressed in these cell lines and can be a potential mechanism to increase NAE levels in multiple CNS cell types. Furthermore, NAPE-PLD is expressed throughout the brain, with highest expression in the dentate gyrus.^61,62^ NAPE-PLD activity was reported to be low in the rat hypothalamus and high in the thalamus,^63^ which could explain why CBD failed to increase NAEs in the WT HYP and increased all six NAEs in the THAL. However, studies in mice demonstrated that NAPE-PLD is moderately expressed in the ventromedial hypothalamus,^61,62,64^ and NAPE-PLD contributes to NAE production in the hypothalamus.^17^ Therefore, additional studies that measure NAPE-PLD expression and activity in specific brain areas and cell lines will be needed to confirm that NAPE-PLD is a target for CBD to validate that the effects on lipid levels by CBD are happening where NAPE-PLD is expressed.

There are a number of avenues for future study that will be important to pursue to test the hypothesis that there is an interaction between NAPE-PLD and CBD that is driving these changes in the lipidome. Evidence that small hydrophobic molecules can allosterically modify NAPE-PLD activity to enhance NAE formation provides an interesting pathway for CBD to exert its effects.^65^ One of the primary lipids shown to regulate NAPE-PLD is deoxycholic acid (DA). CBD and DA are both small molecule lipids and share some characteristics, in much the same way that 2-AG and AEA are similar to THC. The possibility that CBD has affinity for the bile acid binding sites on NAPE-PLD has not yet been investigated. This could be done with more traditional biochemical methods or another avenue of study will be to investigate the molecular dynamics data using modeling of the DA active site with CBD. It is also possible that even if CBD does not directly bind to these sites it could still modify NAPE-PLD activity by altering levels of bile acids, which are endogenous activators of NAPE-PLD.^65^ One hypothesis to test in follow-up studies would be that CBD modulates CYP450 enzymes that metabolize bile acids.^66^ Data presented here do not provide a direct mechanism of action between CBD and NAPE-PLD; however, they do provide data that there is a relationship between the two that ultimately drives changes in the lipidome.

## Conclusions

Lipidomics is an important research tool that can help generate hypotheses regarding novel signaling pathways. In this study, we demonstrate that CBD drives broad-ranging effects in the brain lipidome that may improve the understanding of CBD's mechanisms of action. Furthermore, we show that THC, CBD, and THC:CBD generate different cell-type dependent patterns of lipid regulation. The distinct lipid profile of FAAH deletion and inhibition compared to the profile of CBD treatment revealed important distinctions between the two. These data provide evidence to pursue the novel hypothesis that CBD regulates NAPE-PLD activity, adding to the many potential protein targets for CBD.

## Supplementary Material

Supplemental data

## Abbreviations Used

2-AG: 2-arachidonoyl glycerol
AA: arachidonic acid
AEA: *N*-arachidonoyl ethanolamine
A-Phe: *N*-arachidonoyl phenylalanine
A-Taur: *N*-arachidonoyl taurine
BAL: below analytical limit
CBD: cannabidiol
CER: cerebellum
CNS: central nervous system
CTX: cortex
d_8_AEA: deuterium-labeled *N*-arachidonoyl ethanolamine
d_8_NAGly: deuterium-labeled *N*-arachidonoyl glycine
DA: deoxycholic acid
DEA: *N*-docosahexaenoyl ethanolamine
eCB: endogenous cannabinoid
FAAH: fatty acid amide hydrolase
HIPP: hippocampus
HPLC: high-pressure liquid chromatography
HPLC/MS/MS: HPLC coupled with tandem mass spectrometry
HYP: hypothalamus
KO: knockout
MAGL: monoacylglycerol lipase
MID: midbrain
NAE: *N*-acyl ethanolamine
NAGly: *N*-arachidonoyl glycine
NAPE-PLD: *N*-acyl phosphatidyl ethanolamine-specific phospholipase D
OEA: *N*-oleoyl ethanolamine
PEA: *N*-palmitoyl ethanolamine
PG: prostaglandin
STEM: brainstem
STR: striatum
THAL: thalamus
UGT: uridine 5′-diphospho-glucuronosyltransferase
WT HIPP: wild-type hippocampus
