# Supplementary material for: Cannabidiol's Upregulation of N-acyl Ethanolamines in the Central Nervous System Requires N-acyl Phosphatidyl Ethanolamine-Specific Phospholipase D
Source: Cannabis Cannabinoid Res. 2018 Nov 30;3(1):228–41. doi: 10.1089/can.2018.0031 (PMC6277981; doi:10.1089/can.2018.0031)
Supplement: Supplemental data [file Supp_Data.docx]

Supplemental Methods, Figures, and Tables

**Supplemental Figure Contents and Legends**

**Supplemental Figure 1:** Example overlay chromatograms for *N*-arachidonoyl ethanolamine (AEA; panel A) and cannabidiol (CBD; panel B). The chromatogram is the visual output from the HPLC/MS/MS analysis. After being separated by HPLC, each analyte produces its own peak (if present in the sample) on the chromatogram. The x-axis of the chromatogram is the retention time in minutes, based on when the analyte elutes from the C18 analytical column. The y-axis is the signal intensity in counts per second. The area under the represents the concentration of analyte present in the sample. For each lipid screened, a standard of a known concentration is run first, and unknown analytes with matching parent and ion fragment ion masses and matching retention times are compared to these standards. Panel A shows the 10 fmol AEA standard peak (10fmol standard; solid blue line) overlaid with AEA peaks found in 2 samples from the wild-type (WT) cerebellum, one after treatment with 3mg/kg CBD (CER_CBD3; dashed green line), and the other after vehicle (CER_V3; solid red line). Panel B shows a 30 fmol CBD standard peak (30fmol standard; solid blue line) overlaid with a CBD peak from a sample extracted from a single WT cerebellum after acute CBD (CER_CBD1; solid red line).

**Supplemental Figure 2:** Lipids in HPLC/MS/MS screening library with parent ion and fragment ion masses. Lipids are grouped by amide family. Negative ionization mode, resulting in a [M – H]^-^ parent ion, is used for all methods except the *N*-acyl ethanolamine, 2-acyl glycerol and phytocannabinoid methods, which use positive ionization and generate a [M + H]^+^ parent ion.

**Supplemental Figure 3:** Key for heatmaps showing effects of 2 hour stimulation with 1 µM THC, 1 µM CBD, and 1 µM THC:CBD on a lipid’s concentration within a particular cell line, effects of 2 hour stimulation with 1 µM URB597 in BV2 cells, and effects of 3 mg/kg CBD on lipid levels in 8 brain regions of WT and NAPE-PLD KO mice 2 hours post injection. The arrow color (top section) indicates the direction of a significant result in a drug-treatment condition relative to a vehicle condition. Green colors represent increases and orange color represents decreases in a lipid’s concentration. The number of arrows (bottom section) indicates the magnitude of the difference between levels of that lipid in drug and vehicle conditions. See Supplemental Methods for details in how the magnitudes were calculated.

**Supplemental Figure 4: Significant effects of 3 mg/kg systemic cannabidiol (CBD) in 8 brain regions of adult female C57 mice on lipid levels 2 hours post-injection.** Cells with shaded arrows indicate a change for that lipid in the CBD-exposed brain area relative to the same vehicle-exposed area. Only lipids that changed in at least 1 brain area are shown here. The arrow color indicates the direction of a significant result relative to control. Green colors represent increases, whereas orange colors represent decreases in a lipid’s concentration. Darker colors represent a difference of p < 0.05 and lighter colors represent a change of p < 0.10. The number of arrows indicates the magnitude of the difference between CBD and vehicle. One arrow indicates a magnitude difference of less than 1.5 fold, 2 arrows indicate a 1.5-1.99 fold change, and 3 arrows indicate a 2-2.99 fold change. BAL stands for “Below Analytical Limit,” whereas a blank cell indicates that there was no change in the lipid’s level due to CBD. See Supplemental Methods and Supplemental Figure 3 for more detailed description of analysis. STR=striatum; HIPP=hippocampus; CER=cerebellum; THAL=thalamus; CTX=cortex; HYP=hypothalamus; MID=midbrain; STEM=brainstem.

**Supplemental Figure 5: Overall impact of 2 hour stimulation with 1 µM Δ9-tetrahydrocannabinol (THC), 1 µM cannabidiol (CBD) or 1 µM THC:CBD on targeted lipid levels in BV2, C6 and N18 cell lines and impact of 2 hour stimulation with 1 µM URB597 on targeted lipid levels in BV2 cells.** The column shaded in blue shows, for each cell line, the percentage of lipids detected (out of 85 lipids screened) with concentrations that did not significantly differ between drug and vehicle treated groups. The middle column shaded in green shows, for each cell line, the percentage of the lipids detected with concentrations that were significantly higher in the drug treated cells relative to the vehicle treated cells. Finally, the furthest right column shaded in orange shows, for each cell line, the percentage of the lipids detected that were significantly lower in concentration in the drug treated cells relative to the vehicle treated cells. See Supplemental Methods for a more detailed description of how percentages were obtained.

**Supplemental Figure 6: Comparison of significant effects of 1 µM Δ^9^-tetrahydrocannabinol (THC) stimulation, 1 µM cannabidiol (CBD) stimulation, and combined 1 µM Δ^9^-tetrahydrocannabinol (THC) and cannabidiol (CBD) stimulation for 2 hours on the lipidome between BV2, C6, and N18 cell lines.** BAL = below analytical limit. Only lipids that were significantly affected by one of the drug treatments in at least 1 of the cell lines are shown here. The color of the cell indicates the direction of change of a lipid’s concentration with drug relative to vehicle: orange is a decrease and green is an increase. Darker colors indicate p< 0.05 whereas lighter colors indicate p< 0.10. The number of arrows represents the magnitude of the change. 1 arrow = 1 – 1.5 fold change; 2 arrows = 1.5 – 2 fold change; 3 arrows = 2-3 fold change; 4 arrows = 3-10 fold change; 5 arrows = more than 10 fold change. See Supplemental Methods and Supplemental Figure 3 for more details

**Supplemental Figure 7: Bar graphs showing the effects of 2 hour stimulation with 1 µM Δ^9^-tetrahydrocannabinol (THC), 1 µM cannabidiol (CBD) and 1 µM THC:CBD on mean levels of the endogenous cannabinoid *N*-arachidonoyl ethanolamine (AEA).** Units on the y axis are picomoles of AEA per gram cell pellet. Error bars are ± standard error. Open bars represent vehicle, black bars represent THC, light gray bars represent CBD, and dark gray bars represent the combination. Asterisk (*) indicates a significant (p < 0.05) change between drug and vehicle.

**Supplemental Figure 8: Significant effects of 2 hour stimulation with 1 µM URB597 on targeted lipid levels in BV2 microglia.** Only lipids that significantly increased (green cells) or decreased (orange cells) relative to vehicle at p < 0.05 are shown. The number of arrows represents the magnitude of change; 1 arrow = 1 – 1.5 fold change; 2 arrows = 1.5 – 2 fold change; 3 arrows = 2-3 fold change; 4 arrows = 3-10 fold change; 5 arrows = more than 10 fold change. Please refer to Supplemental Figure 3 and Supplemental Methods for details.

**Supplemental Figure 9: Significant effects of 3 mg/kg systemic cannabidiol (CBD) on lipid levels in 8 brain regions of adult female *N*-acyl phosphatidyl ethanolamine-specific phospholipase D (NAPE-PLD) knockout mice 2 hours post-injection.** Cells with shaded arrows indicate a change for that lipid in the CBD-exposed brain area relative to the same vehicle-exposed area. Only lipids that changed in at least 1 brain area are shown here. The arrow color indicates the direction of a significant result relative to control. Green colors represent increases, whereas orange colors represent decreases in a lipid’s concentration. Darker colors represent a difference of p < 0.05 and lighter colors represent a change of p < 0.10. The number of arrows indicates the magnitude of the difference between CBD and vehicle. One arrow indicates a magnitude difference of less than 1.5 fold, 2 arrows indicate a 1.5-1.99 fold change, 3 arrows indicate a 2-2.99 fold change, and 4 arrows indicate a 3-10 fold change. BAL stands for “Below Analytical Limit,” whereas a blank cell indicates that there was no change in the lipid’s level due to CBD. See Supplemental Methods and Supplemental Figure 3 for more detailed description of analysis. STR=striatum; HIPP=hippocampus; CER=cerebellum; THAL=thalamus; CTX=cortex; HYP=hypothalamus; MID=midbrain; STEM=brainstem.

**Supplemental Figure 10: Percentage of significant changes in the C57 WT female mouse brain lipidome and NAPE-PLD KO mouse brain 2 hours after a 3 mg/kg systemic cannabidiol (CBD) injection.** The left, blue part of the figure represents the percentage of the lipids detected within each of brain region (row) within each genotype (column) with concentrations that were unaffected by acute CBD. The middle, green part represents the percentage of lipids detected that increased with CBD treatment and the right, orange proportion represents the percentage of lipids detected that decreased with CBD treatment relative to vehicle. STR=striatum; HIPP=hippocampus; CER=cerebellum; THAL=thalamus; CTX=cortex; HYP=hypothalamus; MID=midbrain; STEM=brainstem. See Supplemental Methods for more details.

**Supplemental Table Contents and Legends**

Abbreviations: BDL, below detection limit; PISSR, present in some samples at random (not detected in ALL samples for that brain region); SD, standard deviation, SE, standard error

**Supplemental Table 1:** Lipid levels in BV2 microglia after 2 hours stimulation with vehicle or 1 μM THC

**Supplemental Table 2:** List of lipids in BV2 microglia significantly affected by 1 μM THC

**Supplemental Table 3:** Lipid levels in C6 glioma after 2 hours stimulation with vehicle or 1 μM THC

**Supplemental Table 4:** List of lipids in C6 glioma significantly affected by 1 μM THC

**Supplemental Table 5:** Lipid levels in N18 neuroblastoma after 2 hours stimulation with vehicle or 1 μM THC

**Supplemental Table 6:** List of lipids in N18 neuroblastoma significantly affected by 1 μM THC

**Supplemental Table 7:** Lipid levels in BV2 microglia after 2 hours stimulation with vehicle or 1 μM CBD

**Supplemental Table 8:** List of lipids in BV2 microglia significantly affected by 1 μM CBD

**Supplemental Table 9:** Lipid levels in C6 glioma after 2 hours stimulation with vehicle or 1 μM CBD

**Supplemental Table 10:** List of lipids in C6 glioma significantly affected by 1 μM CBD

**Supplemental Table 11:** Lipid levels in N18 neuroblastoma after 2 hours stimulation with vehicle or 1 μM CBD

**Supplemental Table 12:** List of lipids in N18 neuroblastoma significantly affected by 1 μM CBD

**Supplemental Table 13:** Lipid levels in BV2 microglia after 2 hours stimulation with vehicle or 1 μM THC:CBD

**Supplemental Table 14:** List of lipids in BV2 microglia significantly affected by 1 μM THC:CBD

**Supplemental Table 15:** Lipid levels in C6 glioma after 2 hours stimulation with vehicle or 1 μM THC:CBD

**Supplemental Table 16:** List of lipids in C6 glioma significantly affected by 1 μM THC:CBD

**Supplemental Table 17:** Lipid levels in N18 neuroblastoma after 2 hours stimulation with vehicle or 1 μM THC:CBD

**Supplemental Table 18:** List of lipids in N18 neuroblastoma significantly affected by 1 μM THC:CBD

**Supplemental Table 19:** Lipid levels in BV2 microglia after 2 hours stimulation with vehicle or 1 μM URB597

**Supplemental Table 20:** List of lipids in BV2 microglia significantly affected by 1 μM URB597

**Supplemental Table 21:** Levels of THC, CBD, and THC metabolites between BV2 cells treated with either drug alone or in combination

**Supplemental Table 22:** ANOVA comparing levels of THC, CBD, and THC metabolites between BV2 cells treated with either drug alone or in combination

**Supplemental Table 23:** Levels of THC, CBD, and THC metabolites between C6 cells treated with either drug alone or in combination

**Supplemental Table 24:** ANOVA comparing levels of THC, CBD, and THC metabolites between C6 cells treated with either drug alone or in combination

**Supplemental Table 25:** Levels of THC, CBD, and THC metabolites between N18 cells treated with either drug alone or in combination

**Supplemental Table 26:** ANOVA comparing levels of THC, CBD, and THC metabolites between N18 cells treated with either drug alone or in combination

**Supplemental Table 27:** Lipid levels in the striatum of WT female mice treated with Vehicle or 3 mg/kg CBD

**Supplemental Table 28:** List of lipids in the WT striatum significantly affected by 3mg/kg CBD

**Supplemental Table 29:** Lipid levels in the hippocampus of WT female mice treated with Vehicle or 3 mg/kg CBD

**Supplemental Table 30:** List of lipids in the WT hippocampus significantly affected by 3mg/kg CBD

**Supplemental Table 31:** Lipid levels in the cerebellum of WT female mice treated with Vehicle or 3 mg/kg CBD

**Supplemental Table 32:** List of lipids in the WT cerebellum significantly affected by 3mg/kg CBD

**Supplemental Table 33:** Lipid levels in the thalamus of WT female mice treated with Vehicle or 3 mg/kg CBD

**Supplemental Table 34:** List of lipids in the WT thalamus significantly affected by 3mg/kg CBD

**Supplemental Table 35:** Lipid levels in the cortex of WT female mice treated with Vehicle or 3 mg/kg CBD

**Supplemental Table 36:** List of lipids in the WT cortex significantly affected by 3mg/kg CBD

**Supplemental Table 37:** Lipid levels in the hypothalamus of WT female mice treated with Vehicle or 3 mg/kg CBD

**Supplemental Table 38:** List of lipids in the WT hypothalamus significantly affected by 3mg/kg CBD

**Supplemental Table 39:** Lipid levels in the midbrain of WT female mice treated with Vehicle or 3 mg/kg CBD

**Supplemental Table 40:** List of lipids in the WT midbrain significantly affected by 3mg/kg CBD

**Supplemental Table 41:** Lipid levels in the brainstem of WT female mice treated with Vehicle or 3 mg/kg CBD

**Supplemental Table 42:** List of lipids in the WT brainstem significantly affected by 3mg/kg CBD

**Supplemental Table 43:** Lipid levels in the striatum of NAPE-PLD KO female mice treated with Vehicle or 3 mg/kg CBD

**Supplemental Table 44:** List of lipids in the NAPE-PLD KO striatum significantly affected by 3mg/kg CBD

**Supplemental Table 45:** Lipid levels in the hippocampus of NAPE-PLD KO female mice treated with Vehicle or 3 mg/kg CBD

**Supplemental Table 46:** List of lipids in the NAPE-PLD KO hippocampus significantly affected by 3mg/kg CBD

**Supplemental Table 47:** Lipid levels in the cerebellum of NAPE-PLD KO female mice treated with Vehicle or 3 mg/kg CBD

**Supplemental Table 48:** List of lipids in the NAPE-PLD KO cerebellum significantly affected by 3mg/kg CBD

**Supplemental Table 49:** Lipid levels in the thalamus of NAPE-PLD KO female mice treated with Vehicle or 3 mg/kg CBD

**Supplemental Table 50:** List of lipids in the NAPE-PLD KO thalamus significantly affected by 3mg/kg CBD

**Supplemental Table 51:** Lipid levels in the cortex of NAPE-PLD KO female mice treated with Vehicle or 3 mg/kg CBD

**Supplemental Table 52:** List of lipids in the NAPE-PLD KO cortex significantly affected by 3mg/kg CBD

**Supplemental Table 53:** Lipid levels in the hypothalamus of NAPE-PLD KO female mice treated with Vehicle or 3 mg/kg CBD

**Supplemental Table 54:** List of lipids in the NAPE-PLD KO hypothalamus significantly affected by 3mg/kg CBD

**Supplemental Table 55:** Lipid levels in the midbrain of NAPE-PLD KO female mice treated with Vehicle or 3 mg/kg CBD

**Supplemental Table 56:** List of lipids in the NAPE-PLD KO midbrain significantly affected by 3mg/kg CBD

**Supplemental Table 57:** Lipid levels in the brainstem of NAPE-PLD KO female mice treated with Vehicle or 3 mg/kg CBD

**Supplemental Table 58:** List of lipids in the NAPE-PLD KO brainstem significantly affected by 3mg/kg CBD

**Supplemental Table 59:** Descriptive statistics for CBD levels in 8 brain areas of WT animals

**Supplemental Table 60:** Results of ANOVA for WT animals to determine effect of brain region on levels of CBD

**Supplemental Table 61:** Results of post-hoc Fisher’s LSD for WT animals

**Supplemental Table 62:** Descriptive statistics for CBD levels in 8 brain areas of NAPE-PLD KO animals

**Supplemental Table 63:** Results of ANOVA for NAPE-PLD KO animals to determine effect of brain region on levels of CBD

**Supplemental Table 64:** Results of post-hoc Fisher’s LSD for NAPE-PLD KO animals

**Supplemental Methods**

**Mobile Phase Ingredients**

Mobile phase A: 20% methanol, 80% water (v/v) and 1 mM ammonium acetate (Sigma Aldrich, St. Louis, MO, USA). Mobile phase B: 100% methanol, 1 mM ammonium acetate.

**Interpreting the effects of combining THC and CBD on THC and CBD incorporation and THC metabolism in BV2, C6, and N18 cell lines**

Analysis of the effects of CBD on THC metabolism used ratios for calculations instead of absolute values to normalize across experiments. The first step involved calculating the percentage of THC given to the cells that was incorporated in the sample 2 hours later. For example, the treatment media for C6 cells was 4mL of 1µM THC. We need to know how many moles of THC went into each flask. In the case of the C6 cells, 1x10^-6^ moles per liter is multiplied by 4 mL and divided by 1000 mL to give 1x10^- 9^ moles per flask. However, the number of cells varied in each flask. To adjust for the cell number, the moles of THC per flask were divided by the pellet weight in grams. This was done for each flask, meaning that there were 6 values per experiment. For our C6 cells, the pellet for THC 1 weighed 0.00109 g. 1.0x10^-9^ divided by 0.00109 yields 3.656x10^-6^ moles per gram. This is the maximum theoretical THC concentration that could be incorporated by the cells. We acknowledge that the cells will not incorporate all of the THC and that some will remain in the treatment media, but this was not measured. The concentration of THC in moles per gram measured in each flask after the 2 hour stimulation was then divided by the corresponding maximum theoretical incorporated THC value and multiplied by 100 to give the percentage of THC incorporated into cells after 2 hours. For C6 THC 1, the concentration of THC measured was 5.77x10^-8^ moles per gram. Dividing this value by the maximum THC concentration and multiplying by 100 gives 1.58%. The fractions of THC incorporated after 2 hours in each experiment were averaged so that a mean percentage of THC incorporated could be compared between THC and THC:CBD treated cells within a cell line using a one-way ANOVA. In the case of the C6 cells, the mean percentage of THC incorporated was 1.36% when cells were treated with THC alone but was 4.09% when cells were treated with the combination of THC and CBD. For cells treated with CBD, a similar procedure as described above was used to calculate the percentage of the original CBD given to each flask that was incorporated in cells after 2 hours. The percentage of CBD incorporated was then compared between cells treated with CBD alone and cells treated with a combination of THC and CBD.

The next stages of analysis involved calculating the ratios between concentrations of incorporated THC and metabolites measured after 2 hours (Figure 3). Each ratio was calculated and then averaged to give a mean ratio of 11-OH-THC to incorporated THC and a mean ratio of THC-COOH to incorporated THC for each cell line treated with THC and THC:CBD. The ratios were calculated on an individual flask level by taking the concentration of the metabolite and dividing it by the concentration of incorporated THC. Ratios were then multiplied by 100 to be expressed as a percentage. These ratios were then averaged so that THC and THC:CBD groups could be compared. For example, the concentration of 11-OH-THC in C6 THC 1 was 1.98x10^-9^ moles per gram. Dividing that value by the level of incorporated THC in the same flask (5.77x10^-8^ moles per gram) and multiplying by 100 yielded 3.44%. Averaging across samples gave 3.38% as the ratio between 11-OH-THC and incorporated THC in C6 cells treated with THC, which was significantly higher than the percentage when the same cell line was treated with THC:CBD. A similar procedure was used to find the ratio between THC-COOH and incorporated THC – concentrations of THC-COOH were divided by THC concentrations at 2 hours and averaged. The final ratio to be examined was between THC-COOH and 11-OH-THC. This was calculated by taking the concentration of THC-COOH and dividing it by the concentration of 11-OH-THC and averaging it across THC and THC:CBD treatment groups.

**Interpreting the effects of THC and CBD on the lipidome in BV2, C6, and N18 cells**

As a first level of analysis, the percentage of the lipidome affected by each of the treatments in each cell line was calculated (Supplemental Figure 4). To do this, we first counted how many lipids were detected in each experiment. For example, in N18 cells treated with THC (as well as their corresponding vehicle-treated cells), 58 endogenous lipids were detected. The percentage of the lipidome that was unchanged was calculated by subtracting the number of lipids that significantly changed from the number detected. In the case of N18 cells given THC, 22 lipids significantly changed out of 58 detected, 58 minus 22 equals 36. This number is then divided by the number of lipids detected and multiplied by 100 to give the percentage unchanged. 36 divided by 58 times 100 is 62.07%, meaning that 62.07% of the detected lipids did not change concentration significantly in N18 cells treated with THC relative to vehicle. The percentage of the lipidome that increased in concentration was calculated by taking the number of lipids that significantly increased with treatment and dividing it by the number of lipids detected and multiplying that by 100. In the case of the N18 THC experiment, 19 out of 58 times 100 equals 32.76%, meaning that 32.76% of the lipids detected were upregulated by THC in N18 cells. The process for calculating the percent decrease is the same for the percent increase, but the number of lipids that were significantly downregulated is used instead. Continuing with the same example, 3 divided by 58 times 100 is 5.17, meaning that 5.17% of the lipids detected in N18 cells were downregulated. The percentage of the lipidome that was unaffected, the percentage that was upregulated and the percentage that was downregulated should total 100%.

Analyzed lipidomics data are represented in tabular format illustrating both the direction and magnitude of change (Supplemental Figure 2). To determine the magnitude change, the mean level of a lipid in the drug-treated group of a specific cell line was divided by that same lipid’s mean level in the corresponding vehicle-treated cells. For example, the average level of AA in the THC-stimulated C6 glioma cells was 8.14x10^-9^ moles per gram and the average level of AA in the vehicle-stimulated C6 cells was 6.99x10^-9^ moles per gram; 8.14x10^-9^ divided by 6.99x10^-9^ equals 1.16, assigning it 1 up arrow in the figures because the magnitude of change was between 1 and 1.5 times higher than vehicle. For decreases the process was very similar: the mean level in the drug group was divided by the mean level in the vehicle; however, the reciprocal of the decimal was taken to express a fold decrease (if the level in the THC group is ½ of the vehicle level then that is a 2-fold decrease). As an example, the mean level of 2-AG was 1.1x10^-8^ moles per gram in the THC-treated C6 glioma cells and 1.4x10^-8^ moles per gram in the corresponding vehicle C6 glioma cells. 1.1x10^-8^ divided by 1.4x10^-8^ is 0.79 and the reciprocal of 0.79 is 1.27, meaning that the decrease is between 1 and 1.5 times vehicle levels and giving it 1 down arrow on our scale. The same instructions were used to calculate the direction and magnitude of change in lipid levels caused by CBD or the combination of THC:CBD versus vehicle.

**Interpreting Results Generated from Mouse Brain Area Samples**

The first section of the results for the brain data describes analysis to calculate the proportion of the lipidome that was detected in the screenings. For example, per genotype, across the entire brain there are 656 endogenous lipids that could be detected in all the samples, as there are 82 endogenous lipids screened for in each of the 8 brain areas. The number of lipids detected in each brain region was summed and then divided by 656 and multiplied by 100 to result in the percent detected. For example, in the WT brain, 60 lipids were detected in the STR, 66 in HIPP, 71 in CER, 67 in THAL, 72 in CTX, 58 in HYP, 64 in MID, and 67 in the STEM. Adding those numbers together gives us 525. 525 divided by 656 and multiplied by 100 gives us 80.03% as the overall percent detection for the adult brain. This also means that there are only 525 lipids for which we could possibly measure a significant change due to acute CBD in the WT brain, as lipids that were not detected in all samples were not analyzed.

The next level of analysis was to take the number of lipids that significantly changed with acute CBD in each of the 8 brain regions and add them together and divide by the number of lipids detected to gain an overall percentage of the lipidome affected by acute CBD in each genotype. For example, levels of 28 lipids changed with acute CBD in the WT STR, 29 in the HIPP, 13 in the CER, 33 in the THAL, 31 in the CTX, 12 in the HYP, 25 in the MID, and 23 in the STEM. Adding those numbers together gives us 194. 194 divided by 525 and multiplied by 100 gives us 36.95%. This means that of the potential changes in the WT lipidome, 36.95% of them occurred.

Like the analysis for the cell lines shown in Supplemental Figure 4, for the analysis in Supplemental Figure 9, the percentage of the lipidome affected by acute CBD in each brain area was calculated for each genotype. Only a portion of the lipids detected in each brain region were significantly affected by acute CBD. To calculate the percent of the lipids detected in each brain area that were unaffected by acute CBD, the number of significant changes in a brain area was first subtracted from the number of endogenous lipids detected in that area (which represents the number of possible changes). This number is then divided by the number of lipids detected and multiplied by 100. For example, in the WT HIPP, there were 66 lipids detected and 29 of them changed with acute CBD. 66 minus 29 equals 37. 37 divided by 66 times 100 is 56.06%. This means that 56.06% of the detected lipidome did not change with acute CBD in the WT HIPP. This percentage can be viewed in Supplemental Figure 9. The next portion of Supplemental Figure 9 (middle, green) gives the percentage of the lipids detected in each brain area with concentrations significantly increased in the CBD group relative to vehicle. Sticking with the WT HIPP, of the 29 changes that were detected, 25 of them were increases. 25 is then divided by the number of lipids detected, which in this case was 66, and multiplied by 100 to yield the percentage increased. For the WT HIPP, the percentage of the detected lipidome that was upregulated by CBD was 37.88%. The right portion of Supplemental Figure 9 shows the percentage of the lipids detected in each brain area that decreased in concentration with acute CBD. The percentage is calculated by taking the number of lipids with significantly decreased concentrations in each brain region and dividing it by the number of lipids detected in that brain region. For the WT HIPP, 4 lipids decreased with acute CBD, giving a percentage of 6.06% for the proportion of the lipidome that decreased. When the 3 different percentages for a brain region are added, they should equal 100. For the WT HIPP, 56.06 plus 37.88 plus 6.06 equals 100.

Using the same key as the cell data, analyzed lipidomics data from the brain regions are represented in tabular format illustrating both the direction and magnitude of change (Supplemental Figure 2). For example, the mean level of AEA was 9.82x10^-11^ moles per gram in the HIPP of the WT CBD mice and 7.36x10^-11^ moles per gram in the corresponding vehicle HIPP. 9.82x10^-11^ divided by 7.36x10^-11^ is 1.33, assigning it 1 up arrow in the figures because the magnitude of change was between 1 and 1.5 times higher than vehicle. Providing an example of a decrease, the average level of PGE_2_ in the HIPP of WT CBD-treated mice was 4.14x10^-10^ moles per gram and the average level of PGE_2_ in the HIPP of the vehicle was 4.61x10^-10^ moles per gram; 4.14x10^-10^ divided by 4.61x10^-10^ equals 0.90, and the reciprocal of 0.90 is 1.11, meaning that the decrease is between 1 and 1.5 times vehicle levels and giving it 1 down arrow on our scale.

**Supplemental Figure 1: Example overlay chromatograms**

**A.**

**B.**

**Supplemental Figure 2: Lipids in HPLC/MS/MS screening library**

| *N*-acyl alanine | [M–H]^-^ | Fragment |  | *N*-acyl proline | [M–H]^-^ | Fragment |
| --- | --- | --- | --- | --- | --- | --- |
| *N*-palmitoyl alanine | 326.5 | 88.09 |  | *N*-palmitoyl proline | 352.53 | 114.12 |
| *N*-stearoyl alanine | 354.55 | 88.09 |  | *N*-stearoyl proline | 380.59 | 114.12 |
| *N*-oleoyl alanine | 352.53 | 88.09 |  | *N*-oleoyl proline | 378.31 | 114.12 |
| *N*-linoleoyl alanine | 350.52 | 88.09 |  | *N*-linoleoyl proline | 376.56 | 114.12 |
| *N*-arachidonoyl alanine | 374.5 | 88.09 |  | *N*-arachidonoyl proline | 400.58 | 114.12 |
| *N*-docosahexaenoyl alanine | 398.56 | 88.09 |  | *N*-docosahexaenoyl proline | 424.6 | 114.12 |
| *N*-acyl ethanolamine | [M+H]^+^ | Fragment |  | *N*-acyl serine | [M–H]^-^ | Fragment |
| *N*-palmitoyl ethanolamine | 300.29 | 62.1 |  | *N*-palmitoyl serine | 342.3 | 74 |
| *N*-stearoyl ethanolamine | 328.3 | 62.1 |  | *N*-stearoyl serine | 370.3 | 74 |
| *N*-oleoyl ethanolamine | 326.3 | 62.1 |  | *N*-oleoyl serine | 368.3 | 74 |
| *N*-linoleoyl ethanolamine | 324.3 | 62.1 |  | *N*-linoleoyl serine | 366.27 | 74 |
| *N*-arachidonoyl ethanolamine | 348.29 | 62.1 |  | *N*-arachidonoyl serine | 390.3 | 74 |
| *N*-docosahexaenoyl ethanolamine | 372.6 | 62.1 |  | *N*-docosahexaenoyl serine | 414.3 | 74 |
| *N*-acyl GABA | [M–H]^-^ | Fragment |  | *N*-acyl taurine | [M–H]^-^ | Fragment |
| *N*-palmitoyl GABA | 340.54 | 102.1 |  | *N-*palmitoyl taurine | 362.6 | 124 |
| *N*-stearoyl GABA | 368.58 | 102.1 |  | *N*-stearoyl taurine | 390.6 | 124 |
| *N*-oleoyl GABA | 366.57 | 102.1 |  | *N-*oleoyl taurine | 388.6 | 124 |
| *N*-linoleoyl GABA | 364.54 | 102.1 |  | *N*-arachidonoyl taurine | 410.6 | 124 |
| *N*-arachidonoyl GABA | 388.57 | 102.1 |  | *N*-acyl tryptophan | [M–H]^-^ | Fragment |
| *N*-docosahexaenoyl GABA | 412.59 | 102.1 |  | *N*-palmitoyl tryptophan | 441.63 | 203.1 |
| *N*-acyl glycine | [M–H]^-^ | Fragment |  | *N*-stearoyl tryptophan | 469.68 | 203.1 |
| *N*-palmitoyl glycine | 312.26 | 74.2 |  | *N*-oleoyl tryptophan | 467.67 | 203.1 |
| *N*-stearoyl glycine | 340.3 | 74.2 |  | *N*-linoleoyl tryptophan | 465.65 | 203.1 |
| *N*-oleoyl glycine | 338.3 | 74.2 |  | *N*-arachidonoyl tryptophan | 489.67 | 203.1 |
| *N*-linoleoyl glycine | 336.3 | 74.2 |  | *N*-docosahexaenoyl tryptophan | 513.69 | 203.1 |
| *N*-arachidonoyl glycine | 360.3 | 74.2 |  | *N*-acyl tyrosine | [M–H]^-^ | Fragment |
| *N*-docosahexaenoyl glycine | 384.3 | 74.2 |  | *N*-palmitoyl tyrosine | 418.59 | 180.18 |
| *N*-acyl leucine | [M–H]^-^ | Fragment |  | *N*-stearoyl tyrosine | 446.65 | 180.18 |
| *N*-palmitoyl leucine | 368.58 | 130.1 |  | *N*-oleoyl tyrosine | 444.63 | 180.18 |
| *N*-stearoyl leucine | 396.63 | 130.1 |  | *N*-linoleoyl tyrosine | 442.61 | 180.18 |
| *N*-oleoyl leucine | 394.61 | 130.1 |  | *N*-arachidonoyl tyrosine | 466 | 180.18 |
| *N*-linoleoyl leucine | 392.6 | 130.1 |  | *N*-docosahexaenoyl tyrosine | 490.66 | 180.18 |
| *N*-docosahexaenoyl leucine | 440.64 | 130.1 |  | *N*-acyl valine | [M–H]^-^ | Fragment |
| *N*-acyl methionine | [M–H]^-^ | Fragment |  | *N*-palmitoyl valine | 354.31 | 116.31 |
| *N*-palmitoyl methionine | 386.62 | 148.2 |  | *N*-stearoyl valine | 382.6 | 116.14 |
| *N*-stearoyl methionine | 414.64 | 148.2 |  | *N*-oleoyl valine | 380.59 | 116.14 |
| *N*-oleoyl methionine | 412.65 | 148.2 |  | *N*-linoleoyl valine | 378.58 | 116.14 |
| *N*-linoleoyl methionine | 410.64 | 148.2 |  | *N*-docosahexaenoyl valine | 426.62 | 116.14 |
| *N*-arachidonoyl methionine | 434.66 | 148.2 |  | Free Fatty Acids | [M–H]^-^ | Fragment |
| *N*-docosahexaenoyl methionine | 458.68 | 148.2 |  | Oleic acid | 281.5 | 263 |
| *N*-acyl phenylalanine | [M–H]^-^ | Fragment |  | Linoleic acid | 279.5 | 261 |
| *N*-palmitoyl phenylalanine | 402.59 | 164.1 |  | Arachidonic acid | 303.5 | 285 |
| *N*-stearoyl phenylalanine | 430.65 | 164.1 |  | PhosphoLEA | [M–H]^-^ | Fragment |
| *N*-oleoyl phenylalanine | 428.63 | 164.1 |  | phosphoLEA | 403.5 | 58.5 |
| *N*-linoleoyl phenylalanine | 426.61 | 164.1 |  | 2-acyl glycerol | [M+H]^+^ | Fragment |
| *N*-arachidonoyl phenylalanine | 450.64 | 164.1 |  | 2-arachidonoyl glycerol | 379.3 | 287.5 |
| *N*-docosahexaenoyl phenylalanine | 474.66 | 164.1 |  | 2-linoleoyl glycerol | 355.5 | 245 |
| Phytocannabinoids | [M+H]^+^ | Fragment |  | 2-oleoyl glycerol | 357.5 | 265.2 |
| CBD | 315.2 | 191 |  | 2-palmitoyl glycerol | 331.5 | 239.5 |
| THC | 315.2 | 123.2 |  | Prostaglandins | [M–H]^-^ | Fragment |
| THC Metabolites | [M–H]^-^ | Fragment |  | PGE_2_ | 351.2 | 315 |
| (±)-11-nor-9-carboxy-Δ9-THC | 343.5 | 191 |  | PGF_2α_ | 353.3 | 309.2 |
| 11-OH-THC | 329.5 | 173 |  | 6-ketoPGF_1α_ | 369.3 | 206.9 |

**Supplemental Figure 3:** **Key for interpreting heatmaps**

| Decrease relative to vehicle (p≤.05) | ↓ |
| --- | --- |
| Decrease relative to vehicle (.05<p≤.10) | ↓ |
| Increase relative to vehicle (.05<p≤.10) | ↑ |
| Increase relative to vehicle (p≤.05) | ↑ |

| ↑↑↑↑↑ | 10 or more times higher than vehicle |
| --- | --- |
| ↑↑↑↑ | 3-9.99 times higher than vehicle |
| ↑↑↑ | 2-2.99 times higher than vehicle |
| ↑↑ | 1.50-1.99 times higher than vehicle |
| ↑ | 1-1.49 times higher than vehicle |
| ↓ | 1-1.49 times lower than vehicle |
| ↓↓ | 1.50-1.99 times lower than vehicle |
| ↓↓↓ | 2-2.99 times lower than vehicle |
| ↓↓↓↓ | 3-9.99 times lower than vehicle |
| ↓↓↓↓↓ | 10 or more times lower than vehicle |

**Supplemental Figure 4: WT CBD Significant Differences**

| **Lipid Species** | **STR** | **HIPP** | **CER** | **THAL** | **CTX** | **HYP** | **MID** | **STEM** |
| --- | --- | --- | --- | --- | --- | --- | --- | --- |
| ***N*-acyl alanine** |  |  |  |  |  |  |  |  |
| *N*-stearoyl alanine | ↑ | ↑ |  |  |  |  |  |  |
| *N*-oleoyl alanine | ↑ | ↓ | ↓ |  | ↓ | ↓ | ↓ | ↓↓ |
| *N*-linoleoyl alanine |  |  |  | ↓ | ↓↓ |  | BAL | ↓↓ |
| *N*-arachidonoyl alanine | ↑ |  |  | ↑ | ↑ |  | ↑ |  |
| *N*-docosahexaenoyl alanine | BAL |  |  | ↑ |  |  | BAL |  |
| ***N*-acyl ethanolamine** |  |  |  |  |  |  |  |  |
| *N*-palmitoyl ethanolamine |  |  |  | ↑ | ↑ |  |  | ↑ |
| *N*-stearoyl ethanolamine |  |  |  | ↑ | ↑ |  |  |  |
| *N*-oleoyl ethanolamine | ↑ | ↑ |  | ↑↑ | ↑↑ |  | ↑ | ↑↑ |
| *N*-linoleoyl ethanolamine |  | ↑ | ↑ | ↑ | ↑ |  | ↑ | ↑ |
| *N*-arachidonoyl ethanolamine |  | ↑ | ↑ | ↑ | ↑ |  | ↑ | ↑ |
| *N*-docosahexaenoyl ethanolamine |  | ↑ | ↑ | ↑ | ↑ |  | ↑ | ↑ |
| ***N*-acyl GABA** |  |  |  |  |  |  |  |  |
| *N*-stearoyl GABA | ↑ |  |  |  |  |  |  |  |
| *N*-oleoyl GABA | ↑ | ↑ |  |  |  |  |  |  |
| *N*-linoleoyl GABA | BAL | ↑↑ |  |  |  | BAL | ↑ |  |
| *N*-arachidonoyl GABA | ↑ | ↑ |  | ↑ |  |  | ↑ |  |
| *N*-docosahexaenoyl GABA |  | ↑ | ↑ |  |  |  |  |  |
| ***N*-acyl glycine** |  |  |  |  |  |  |  |  |
| *N*-stearoyl glycine |  |  |  |  |  | ↓ |  |  |
| *N*-oleoyl glycine | ↑ | ↑ |  | ↑ |  |  |  |  |
| *N*-linoleoyl glycine |  | ↑ | ↑ | ↑ | ↑ |  | ↑ |  |
| *N*-arachidonoyl glycine | ↑ | ↑ |  | ↑ | ↑ | ↓ | ↑ |  |
| *N*-docosahexaenoyl glycine | ↑ | ↑ |  | ↑ | ↑ | ↑ |  |  |
| ***N*-acyl leucine** |  |  |  |  |  |  |  |  |
| *N*-palmitoyl leucine | ↑ |  |  | ↑ | ↑ |  |  | ↑ |
| *N*-oleoyl leucine | ↓ |  | ↑ |  |  |  |  |  |
| *N*-linoleoyl leucine | ↓ |  |  |  |  |  | BAL |  |
| *N*-docosahexaenoyl leucine | BAL |  |  |  |  |  |  | ↑ |
| ***N*-acyl methionine** |  |  |  |  |  |  |  |  |
| *N*-palmitoyl methionine | ↑ |  |  |  |  |  |  |  |
| *N*-stearoyl methionine |  |  |  |  | ↑ |  |  |  |
| *N*-arachidonoyl methionine | BAL | ↑ | ↑ | ↑ | ↑ | BAL | ↑↑ | ↑↑ |
| ***N*-acyl phenylalanine** |  |  |  |  |  |  |  |  |
| *N*-palmitoyl phenylalanine |  |  |  |  |  |  |  | ↑ |
| *N*-oleoyl phenylalanine | ↑ |  |  |  |  |  | ↑ |  |
| *N*-linoleoyl phenylalanine | BAL | ↑ |  |  |  | BAL | BAL |  |
| *N*-arachidonoyl phenylalanine | ↑↑ | ↑ |  | ↑ | ↑ |  | ↑↑ |  |
| *N*-docosahexaenoyl phenylalanine |  |  |  |  |  | BAL | ↑ |  |
| ***N*-acyl proline** |  |  |  |  |  |  |  |  |
| *N*-palmitoyl proline | BAL | BAL | ↑ |  | ↑↑ |  |  |  |
| ***N*-acyl serine** |  |  |  |  |  |  |  |  |
| *N*-palmitoyl serine |  |  | ↑ | ↑ |  | ↓ |  |  |
| *N*-oleoyl serine |  |  | ↑ | ↑ | ↑ |  | ↑ |  |
| *N*-linoleoyl serine |  |  |  | ↑ |  |  | ↑ |  |
| *N*-arachidonoyl serine | ↑↑ | ↑↑ | ↑ | ↑ | ↑ | BAL | ↑↑ | ↑ |
| *N*-docosahexaenoyl serine |  | ↑ | ↑ | ↑ | ↑ | BAL | ↑ |  |
| ***N*-acyl taurine** |  |  |  |  |  |  |  |  |
| *N*-arachidonoyl taurine |  |  | ↓ |  |  | ↓ |  |  |
| ***N*-acyl tryptophan** |  |  |  |  |  |  |  |  |
| *N*-palmitoyl tryptophan |  | ↑ |  |  |  | BAL |  | BAL |
| *N*-stearoyl tryptophan |  |  |  |  | ↑ | BAL |  | BAL |
| *N*-oleoyl tryptophan | BAL | BAL |  | BAL | ↑ | BAL | BAL | BAL |
| ***N*-acyl tyrosine** |  |  |  |  |  |  |  |  |
| *N*-palmitoyl tyrosine |  |  |  |  |  |  | ↑ |  |
| *N*-arachidonoyl tyrosine | BAL | ↑ |  | ↑ | ↑ | BAL |  | ↑ |
| *N*-docosahexaenoyl tyrosine | BAL | BAL |  | BAL | ↑ | BAL |  | ↑ |
| ***N*-acyl valine** |  |  |  |  |  |  |  |  |
| *N*-palmitoyl valine |  | ↑ |  | ↑ |  |  |  | ↑ |
| *N*- stearoyl valine |  |  |  | ↑ |  | BAL |  |  |
| *N*-oleoyl valine |  |  |  |  | ↑ | BAL |  |  |
| *N*-docosahexaenoyl valine | BAL | BAL | BAL | BAL | ↑↑ | BAL | BAL | BAL |
| **2-acyl glycerol** |  |  |  |  |  |  |  |  |
| 2-palmitoyl glycerol | ↑ |  |  | ↑ |  |  | ↑ |  |
| 2-oleoyl glycerol | ↑ |  |  | ↑ |  |  |  | ↑ |
| 2-linoleoyl glycerol | ↑ | ↑ |  |  |  |  |  | ↑ |
| 2-arachidonoyl glycerol | ↑ |  |  |  |  | ↓↓ |  |  |
| **Free Fatty Acids** |  |  |  |  |  |  |  |  |
| Oleic acid | ↑↑ | ↑↑ | ↑ | ↑ | ↑ | ↑↑ | ↑ | ↑ |
| Linoleic acid | ↑ | ↑↑ |  | ↑ | ↑ |  | ↑ | ↑ |
| Arachidonic acid | ↑ | ↑ |  | ↑ |  |  |  |  |
| **phosphoLEA** |  |  |  |  |  |  |  |  |
| phosphoLEA | ↑↑ |  | ↑ | ↑ | ↑ | ↑ | ↑ | ↑ |
| **Prostaglandins** |  |  |  |  |  |  |  |  |
| PGE_2_ | ↓ | ↓ |  |  |  | ↓ |  | ↓ |
| PGF_2α_ | ↓ | ↓ | ↓ | ↓ | ↓ | ↓ | ↓ | ↓ |
| 6-ketoPGF_1α_ | ↓↓↓ | ↓ | ↓ | ↓↓ | ↓↓ | ↓ | ↓↓ | ↓↓ |

**Supplemental Figure 5: Percentages of lipids altered by drug treatments in cell lines**

| Cell line | Drug | % Unchanged | % Increased | % Decreased |
| --- | --- | --- | --- | --- |
| BV2 | THC | 73.53% | 22.06% | 4.41% |
|  | CBD | 74.19% | 20.97% | 4.84% |
|  | THC:CBD | 55.88% | 30.88% | 13.24% |
|  | URB597 | 55.26% | 34.21% | 10.53% |
| C6 | THC | 71.70% | 13.21% | 15.09% |
|  | CBD | 69.64% | 28.57% | 1.79% |
|  | THC:CBD | 72.41% | 5.17% | 22.41% |
| N18 | THC | 62.07% | 32.76% | 5.17% |
|  | CBD | 66.67% | 31.82% | 1.51% |
|  | THC:CBD | 44.61% | 53.85% | 1.54% |

**Supplemental Figure 6: Effects of THC, CBD, and THC:CBD on the lipidome of cell lines**

|  | BV2 Microglia | | | C6 Glioma Astrocytes | | | N18 Neurons | | |
| --- | --- | --- | --- | --- | --- | --- | --- | --- | --- |
| Lipid Species | THC | CBD | THC:CBD | THC | CBD | THC:CBD | THC | CBD | THC:CBD |
| *N*-stearoyl alanine |  |  |  |  |  |  |  |  | ↑ |
| *N*-oleoyl alanine |  |  | ↑ |  |  |  |  |  |  |
| *N*-palmitoyl ethanolamine |  | ↑ | ↑ |  | ↑ |  |  | ↑ | ↑ |
| *N*-stearoyl ethanolamine |  |  | ↑ |  | ↑ |  |  | ↑ |  |
| *N*-oleoyl ethanolamine |  | ↑ | ↑↑ |  | ↑↑ |  | ↑ | ↑ | ↑ |
| *N*-linoleoyl ethanolamine |  | ↑ | ↑↑ | ↑ | ↑ |  |  | ↑ |  |
| *N*-arachidonoyl ethanolamine |  | ↑ | ↑↑ | ↓ | ↑↑ | ↑ |  | ↑ |  |
| *N*-docosahexaenoyl ethanolamine |  | ↑ | ↑ |  | ↑ |  | ↓↓ | ↑↑ | ↑↑ |
| *N*-palmitoyl GABA |  |  |  |  |  | ↓↓ |  |  | ↑ |
| *N*-stearoyl GABA |  |  | ↑ | BAL |  |  |  | ↑ | ↑ |
| *N*-oleoyl GABA |  |  |  | BAL |  |  |  |  | ↑ |
| *N*-arachidonoyl GABA | BAL | BAL | BAL | ↓ | ↑ |  | BAL | BAL | BAL |
| *N*-docosahexaenoyl GABA | BAL | BAL |  | BAL |  |  | BAL | ↑↑ |  |
| *N*-palmitoyl glycine | ↑↑ |  | ↑ |  |  |  | ↑ |  | ↑ |
| *N*-stearoyl glycine | ↑ |  | ↑ |  |  |  |  | ↑ | ↑ |
| *N*-oleoyl glycine |  |  | ↑ |  |  |  | ↑ | ↑ | ↑ |
| *N*-linoleoyl glycine | ↑ | ↑ | ↑ |  |  |  | ↑ |  |  |
| *N*-arachidonoyl glycine | ↑↑ | ↑ |  |  |  |  | ↑ | ↑↑ |  |
| *N*-docosahexaenoyl glycine | ↑ |  | ↑ |  |  |  |  |  | ↑ |
| *N*-palmitoyl leucine |  |  |  | ↑ |  |  |  |  |  |
| *N*-stearoyl leucine |  | ↑ | ↓ |  |  |  |  |  | ↑ |
| *N*-oleoyl leucine |  | ↑ |  | ↑ | ↑ |  |  |  | ↑ |
| *N*-linoleoyl leucine |  |  | ↓ | BAL |  | ↓ | BAL |  | ↑↑ |
| *N*-docosahexaenoyl leucine |  |  |  | BAL | ↑ |  | BAL | ↑ | ↑ |
| *N*-palmitoyl methionine |  |  | ↓ |  |  |  |  |  | ↓ |
| *N*-stearoyl methionine |  |  | ↓ | BAL | BAL | BAL | BAL |  | ↑↑ |
| *N*-palmitoyl phenylalanine |  |  |  | ↑ |  | ↓↓ |  |  |  |
| *N*-stearoyl phenylalanine |  |  |  |  |  |  |  |  | ↑ |
| *N*-oleoyl phenylalanine |  |  | ↓ | ↑ | ↑↑ |  |  |  | ↑ |
| *N*-arachidonoyl phenylalanine | ↑ |  | ↑ | ↑ | ↑↑ | ↑ | ↑↑ |  | ↑↑ |
| *N*-palmitoyl proline |  |  | ↓↓ |  | ↑ |  |  |  |  |
| *N-*oleoyl proline |  |  |  |  | BAL |  | BAL | ↑ |  |
| *N*-palmitoyl serine |  |  |  |  |  |  |  |  | ↑ |
| *N*-stearoyl serine |  |  | ↑ |  |  |  |  |  | ↑ |
| *N*-oleoyl serine | ↑ | ↑ | ↑ |  |  |  |  |  | ↑ |
| *N*-linoleoyl serine | ↑ |  |  |  |  |  |  |  | ↑ |
| *N*-arachidonoyl serine | ↑ |  | BAL | BAL | BAL |  | ↓ |  | ↑ |
| *N*-palmitoyl taurine | ↓ |  | ↑ |  |  |  | ↑ |  | ↑ |
| *N*-stearoyl taurine |  |  | ↑ |  |  |  | ↑ |  | ↑ |
| *N*-oleoyl taurine |  |  | ↑ | ↓ | ↑ |  | ↑ |  |  |
| *N*-arachidonoyl taurine | ↑ | ↑ | ↑↑ | ↓ | ↑↑ |  | ↑ | ↑ | ↑ |
| *N*-stearoyl tryptophan |  | BAL |  |  | ↓ | BAL |  |  |  |
| *N*-palmitoyl tyrosine |  |  |  |  |  | ↓ |  |  |  |
| *N*-stearoyl tyrosine |  |  |  |  |  | ↓↓ |  |  | ↑ |
| *N-*oleoyl tyrosine |  |  |  |  |  |  | ↑ |  |  |
| *N*-linoleoyl tyrosine | ↑↑ | BAL |  | BAL | BAL | BAL |  |  |  |
| *N*-arachidonoyl tyrosine |  |  |  | BAL | ↑ |  | ↑ | ↑ | ↑↑ |
| *N*-docosahexaenoyl tyrosine |  | BAL |  | BAL | BAL | BAL |  |  | ↑ |
| *N*-palmitoyl valine |  |  |  |  |  | ↓ |  |  |  |
| *N*- stearoyl valine |  |  |  |  |  |  |  | ↑ | ↑ |
| *N*-oleoyl valine |  |  |  |  | ↑↑ |  |  |  |  |
| 2-palmitoyl glycerol |  |  |  | ↓ |  | ↓ |  | ↑ |  |
| 2-oleoyl glycerol | ↓↓ |  |  | ↓↓ |  | ↓ | ↑ | ↑ | ↑ |
| 2-linoleoyl glycerol | ↓ | ↑ | ↓ | ↓ |  | ↓ | ↑ | ↑ | ↑ |
| 2-arachidonoyl glycerol |  |  |  | ↓ |  | ↓ | ↓ |  |  |
| Oleic acid |  |  |  |  |  | ↓ |  | ↑ |  |
| Linoleic acid |  |  |  |  |  | ↓ | ↑ |  |  |
| Arachidonic acid | ↑ | ↓ | ↓ | ↑ |  | ↓ | ↑ |  |  |
| phosphoLEA |  | ↑ | ↓↓ | BAL |  | ↑ | ↑ | ↑ |  |
| PGE_2_ | ↑↑ | ↓ |  | BAL | BAL | BAL | ↑ | ↓ | ↑↑ |
| PGF_2α_ | ↑↑ | ↓↓ | ↑ | BAL | BAL | BAL | BAL | BAL | ↑↑ |
| 6-ketoPGF_1α_ | ↑ |  |  | BAL | BAL | BAL | ↑ | BAL | BAL |

**Supplemental Figure 7: Effects of THC, CBD, and THC:CBD on AEA levels in cell lines**

| Vehicle |  |  | THC |  |  | CBD |  |  | THC:CBD |  |
| --- | --- | --- | --- | --- | --- | --- | --- | --- | --- | --- |

| Effects on levels of AEA | | | |
| --- | --- | --- | --- |
|  | Drug Treatment | | |
| Cell line | THC | CBD | THC:CBD |
| BV2 |  | * | *  * |
| C6 | * | * |  |
| N18 |  | * |  |

**Supplemental Figure 8: Significant effects of URB597 on lipid levels in BV2 microglia**

| **Lipid Species** | **Change with 2h stimulation with 1µM URB597** |
| --- | --- |
| ***N*-acyl alanine** |  |
| *N*-oleoyl alanine | ↑ |
| *N*-linoleoyl alanine | ↑↑↑ |
| *N*-arachidonoyl alanine | ↓ |
| ***N*-acyl ethanolamine** |  |
| *N*-palmitoyl ethanolamine | ↑↑↑↑ |
| *N*-stearoyl ethanolamine | ↑↑↑↑ |
| *N*-oleoyl ethanolamine | ↑↑↑↑↑ |
| *N*-linoleoyl ethanolamine | ↑↑↑↑↑ |
| *N*-arachidonoyl ethanolamine | ↑↑↑↑ |
| *N*-docosahexaenoyl ethanolamine | ↑↑↑↑ |
| ***N*-acyl GABA** |  |
| *N*-palmitoyl GABA | ↑↑↑ |
| *N*-stearoyl GABA | ↑↑ |
| *N*-oleoyl GABA | ↑↑ |
| *N*-linoleoyl GABA | ↑↑ |
| *N*-arachidonoyl GABA | ↓↓ |
| *N*-docosahexaenoyl GABA | ↑↑ |
| ***N*-acyl glycine** |  |
| *N*-palmitoyl glycine | ↑ |
| *N*-stearoyl glycine | ↑↑ |
| *N*-oleoyl glycine | ↑↑↑ |
| *N*-linoleoyl glycine | ↑↑↑ |
| *N*-arachidonoyl glycine | ↓↓ |
| ***N*-acyl leucine** |  |
| *N*-stearoyl leucine | ↓ |
| ***N*-acyl methionine** |  |
| *N*-arachidonoyl methionine | ↓↓↓ |
| ***N*-acyl phenylalanine** |  |
| *N*-arachidonoyl phenylalanine | ↓ |
| ***N*-acyl proline** |  |
| *N*-arachidonoyl proline | ↓↓ |
| ***N*-acyl serine** |  |
| *N*-oleoyl serine | ↑↑↑ |
| *N*-linoleoyl serine | ↑↑↑ |
| *N*-arachidonoyl serine | ↑↑↑ |
| ***N*-acyl taurine** |  |
| *N*-palmitoyl taurine | ↑↑ |
| *N*-stearoyl taurine | ↑↑↑ |
| *N*-oleoyl taurine | ↑↑↑ |
| ***N*-acyl tyrosine** |  |
| *N*-arachidonoyl tyrosine | ↓ |
| **2-acyl glycerol** |  |
| 2-palmitoyl glycerol | ↑↑↑↑ |
| 2-oleoyl glycerol | ↑↑↑ |
| 2-linoleoyl glycerol | ↑ |

**Supplemental Figure 9: NAPE-PLD KO CBD Significant Differences**

| **Lipid Species** | **STR** | **HIPP** | **CER** | **THAL** | **CTX** | **HYP** | **MID** | **STEM** |
| --- | --- | --- | --- | --- | --- | --- | --- | --- |
| ***N*-acyl alanine** |  |  |  |  |  |  |  |  |
| *N*-stearoyl alanine |  |  |  | ↓ |  |  |  |  |
| *N*-oleoyl alanine |  |  | ↓↓ | ↓ | ↓↓ | ↓ | ↓ |  |
| *N*-linoleoyl alanine | BAL |  | ↓↓↓ |  | ↓↓ | BAL |  |  |
| *N*-arachidonoyl alanine | ↑↑ | ↑ | ↓ | ↓ |  | BAL |  | ↑ |
| *N*-docosahexaenoyl alanine | BAL | BAL |  | ↓↓ |  | BAL | BAL |  |
| ***N*-acyl ethanolamine** |  |  |  |  |  |  |  |  |
| *N*-palmitoyl ethanolamine |  | ↓ | ↓ |  |  | ↓↓ |  |  |
| *N*-stearoyl ethanolamine |  |  |  |  |  | ↓↓ |  |  |
| *N*-oleoyl ethanolamine |  |  |  |  |  | ↓↓↓ |  |  |
| *N*-linoleoyl ethanolamine |  |  |  |  |  | ↓↓ |  |  |
| *N*-arachidonoyl ethanolamine |  |  |  |  |  | ↓↓↓ |  |  |
| ***N*-acyl GABA** |  |  |  |  |  |  |  |  |
| *N*-stearoyl GABA |  |  |  | ↓ |  | ↓ |  |  |
| *N*-oleoyl GABA |  |  |  |  |  | ↓ |  |  |
| *N*-arachidonoyl GABA | ↑ |  | ↑ | ↑ |  | ↓ |  | ↑ |
| *N*-docosahexaenoyl GABA |  |  |  |  |  |  |  | ↑ |
| ***N*-acyl glycine** |  |  |  |  |  |  |  |  |
| *N*-palmitoyl glycine |  |  |  | ↓↓ |  |  |  |  |
| *N*-oleoyl glycine |  |  |  |  |  |  | ↓ |  |
| *N*-linoleoyl glycine |  |  | ↓ | ↑ |  | ↓↓ |  |  |
| *N*-arachidonoyl glycine | ↑ | ↑ | ↑ | ↑ |  |  |  |  |
| *N*-docosahexaenoyl glycine |  |  |  | ↑ |  |  |  | ↑ |
| ***N*-acyl leucine** |  |  |  |  |  |  |  |  |
| *N*-palmitoyl leucine |  |  |  |  |  | ↓ |  |  |
| *N*-oleoyl leucine |  |  | ↓ | ↑ |  | ↓ |  |  |
| *N*-linoleoyl leucine |  | ↑ |  | BAL |  | BAL |  | BAL |
| *N*-docosahexaenoyl leucine | BAL | BAL |  | ↓ |  | BAL |  | BAL |
| ***N*-acyl methionine** |  |  |  |  |  |  |  |  |
| *N*-palmitoyl methionine |  |  | ↓ |  |  |  |  |  |
| *N*-stearoyl methionine | ↑ |  | ↓ | ↑ |  | BAL |  |  |
| *N*-oleoyl methionine |  |  | ↓ |  |  | BAL | ↑ |  |
| *N*-arachidonoyl methionine | BAL | BAL | ↓ | BAL | ↓↓↓ | BAL |  |  |
| ***N*-acyl phenylalanine** |  |  |  |  |  |  |  |  |
| *N*-palmitoyl phenylalanine |  |  |  | ↓ |  | ↓ |  |  |
| *N*-oleoyl phenylalanine |  |  | ↓ | ↓ |  | ↓ |  |  |
| *N*-linoleoyl phenylalanine | BAL |  | ↓ |  |  | BAL | ↓ |  |
| *N*-arachidonoyl phenylalanine |  |  |  | ↑ | ↓ |  | ↓ |  |
| *N*-docosahexaenoyl phenylalanine | ↓ |  |  |  |  | BAL |  |  |
| ***N*-acyl serine** |  |  |  |  |  |  |  |  |
| *N*-palmitoyl serine |  |  |  |  |  | ↑ |  |  |
| *N*-stearoyl serine | ↑ |  |  |  |  |  |  |  |
| *N*-oleoyl serine |  | ↑ |  |  | ↓ | ↑ |  |  |
| *N*-linoleoyl serine |  |  |  |  |  | ↑ |  |  |
| *N*-arachidonoyl serine |  |  | ↓ | ↑ |  |  | ↓ |  |
| *N*-docosahexaenoyl serine |  |  |  |  |  | ↑↑ |  |  |
| ***N*-acyl taurine** |  |  |  |  |  |  |  |  |
| *N*-arachidonoyl taurine |  | ↓ |  |  |  | ↓ |  | ↓ |
| ***N*-acyl tyrosine** |  |  |  |  |  |  |  |  |
| *N*-palmitoyl tyrosine |  |  | ↓ |  |  |  |  |  |
| *N*-arachidonoyl tyrosine | BAL |  | ↑ |  |  | BAL |  |  |
| ***N*-acyl valine** |  |  |  |  |  |  |  |  |
| *N*-palmitoyl valine |  |  |  | ↑↑ |  |  |  |  |
| *N*- stearoyl valine |  |  |  | ↑↑ |  |  |  |  |
| *N*-oleoyl valine | ↑ |  |  | ↑↑ |  |  |  |  |
| **2-acyl-glycerol** |  |  |  |  |  |  |  |  |
| 2-palmitoyl glycerol |  | ↑ |  |  |  |  |  |  |
| 2-oleoyl glycerol |  |  |  | ↑ |  | ↓↓↓↓ |  |  |
| 2-linoleoyl glycerol |  | ↑ |  |  |  | ↓↓↓ |  | ↑ |
| 2-arachidonoyl glycerol |  |  |  | ↑ |  | ↓ |  | ↑ |
| **Free Fatty Acids** |  |  |  |  |  |  |  |  |
| Oleic acid | ↑ |  |  |  |  | ↓ |  | ↑ |
| Linoleic acid | ↑ |  | ↓ | ↑ |  | ↓ |  | ↑ |
| Arachidonic acid | ↑ |  |  | ↑ |  | ↓ |  | ↑ |
| **phosphoLEA** |  |  |  |  |  |  |  |  |
| phosphoLEA |  |  | ↑ | ↑↑ | ↑ | ↑↑ | ↑ | ↑ |
| **Prostaglandins** |  |  |  |  |  |  |  |  |
| PGE_2_ |  | ↓ | ↓ | ↓ |  | ↓ | ↓ |  |
| PGF_2α_ | ↓ | ↓ | ↓ | ↓ | ↓ | ↓ | ↓ | ↓ |
| 6-ketoPGF_1α_ | BAL | ↓ | ↓ | ↓↓ | ↓↓ |  | ↓ | ↓ |

**Supplemental Figure 10: Percentages of lipids in mouse brain areas affected by CBD**

|  | % Lipids detected with concentration unchanged by acute 3 mg/kg CBD | | % Lipids detected with concentration increased by acute 3 mg/kg CBD | | % Lipids detected with concentration decreased by acute 3 mg/kg CBD | |
| --- | --- | --- | --- | --- | --- | --- |
|  | WT | NAPE-PLD KO | WT | NAPE-PLD KO | WT | NAPE-PLD KO |
| STR | 53.33% | 81.36% | 38.33% | 15.25% | 8.33% | 3.39% |
| HIPP | 56.06% | 83.08% | 37.88% | 9.23% | 6.06% | 7.69% |
| CER | 74.65% | 68.57% | 19.72% | 5.71% | 5.63% | 25.71% |
| THAL | 50.74% | 56.92% | 44.78% | 24.62% | 4.48% | 18.46% |
| CTX | 56.94% | 88.41% | 37.50% | 1.45% | 5.56% | 10.14% |
| HYP | 79.31% | 46.15% | 5.17% | 9.62% | 15.52% | 44.23% |
| MID | 60.94% | 84.85% | 34.38% | 3.03% | 4.69% | 12.12% |
| STEM | 65.67% | 79.69% | 26.87% | 15.63% | 7.46% | 4.69% |

**Supplemental Table 1: Lipid levels in BV2 microglia cells treated with Vehicle or 1 µM THC for 2 hours**

|  | BV2 | | | | | | |
| --- | --- | --- | --- | --- | --- | --- | --- |
|  | Vehicle | | | 1 µM THC | | | |
| ***N*-acyl alanine** | Mean | Std Dev | Std Error | | Mean | Std Dev | Std Error |
| *N*-palmitoyl alanine | 1.09E-10 | 4.23E-11 | 1.73E-11 | | 1.2E-10 | 2.69E-11 | 1.1E-11 |
| *N*-stearoyl alanine | 8.9E-11 | 4.37E-11 | 1.78E-11 | | 1.08E-10 | 3.99E-11 | 1.63E-11 |
| *N*-oleoyl alanine | 7.15E-11 | 3.09E-11 | 1.26E-11 | | 7.52E-11 | 1.16E-11 | 4.72E-12 |
| *N*-linoleoyl alanine | PISSR |  |  | | PISSR |  |  |
| *N*-arachidonoyl alanine | BDL |  |  | | BDL |  |  |
| *N*-docosahexaenoyl alanine | BDL |  |  | | BDL |  |  |
| ***N*-acyl dopamine** |  |  |  | |  |  |  |
| *N*-oleoyl dopamine | BDL |  |  | | BDL |  |  |
| *N*-arachidonoyl dopamine | BDL |  |  | | BDL |  |  |
| ***N*-acyl ethanolamine** |  |  |  | |  |  |  |
| *N*-palmitoyl ethanolamine | 1.92E-10 | 7.68E-11 | 3.14E-11 | | 1.9E-10 | 9.22E-11 | 3.76E-11 |
| *N*-stearoyl ethanolamine | 2.24E-10 | 2.17E-11 | 8.84E-12 | | 2.43E-10 | 1E-10 | 4.1E-11 |
| *N*-oleoyl ethanolamine | 2.82E-10 | 4.9E-11 | 2E-11 | | 2.78E-10 | 1.09E-10 | 4.44E-11 |
| *N*-linoleoyl ethanolamine | 7.05E-11 | 2.21E-11 | 9.02E-12 | | 6.95E-11 | 2.3E-11 | 9.39E-12 |
| *N*-arachidonoyl ethanolamine | 1.63E-11 | 6.13E-12 | 2.5E-12 | | 1.88E-11 | 7.37E-12 | 3.01E-12 |
| *N*-docosahexaenoyl ethanolamine | 2.14E-11 | 8.1E-12 | 3.31E-12 | | 1.72E-11 | 6.7E-12 | 2.74E-12 |
| ***N*-acyl GABA** |  |  |  | |  |  |  |
| *N*-palmitoyl GABA | 3.83E-11 | 1.61E-11 | 6.57E-12 | | 4.21E-11 | 1.79E-11 | 7.29E-12 |
| *N*-stearoyl GABA | 2.64E-11 | 1.04E-11 | 4.26E-12 | | 2.87E-11 | 1.19E-11 | 4.85E-12 |
| *N*-oleoyl GABA | 2.29E-11 | 4.84E-12 | 1.97E-12 | | 1.94E-11 | 1.93E-12 | 7.89E-13 |
| *N*-linoleoyl GABA | PISSR |  |  | | PISSR |  |  |
| *N*-arachidonoyl GABA | PISSR |  |  | | PISSR |  |  |
| *N*-docosahexaenoyl GABA | PISSR |  |  | | PISSR |  |  |
| ***N*-acyl glycine** |  |  |  | |  |  |  |
| *N*-palmitoyl glycine | 3.64E-10 | 5.92E-11 | 2.42E-11 | | 6.39E-10 | 9.45E-11 | 3.86E-11 |
| *N*-stearoyl glycine | 2.68E-10 | 8.54E-11 | 3.49E-11 | | 3.81E-10 | 7.21E-11 | 2.94E-11 |
| *N*-oleoyl glycine | 1.54E-10 | 4.03E-11 | 1.64E-11 | | 1.81E-10 | 1.79E-11 | 7.33E-12 |
| *N*-linoleoyl glycine | 4.61E-11 | 6.67E-12 | 2.72E-12 | | 6.12E-11 | 8.27E-12 | 3.37E-12 |
| *N*-arachidonoyl glycine | 1.62E-11 | 2.5E-12 | 1.02E-12 | | 2.54E-11 | 2E-12 | 8.15E-13 |
| *N*-docosahexaenoyl glycine | 1.76E-11 | 5.41E-12 | 2.21E-12 | | 2.63E-11 | 4.25E-12 | 1.74E-12 |
| ***N*-acyl leucine** |  |  |  | |  |  |  |
| *N*-palmitoyl leucine | 1.86E-10 | 6.87E-11 | 2.81E-11 | | 1.94E-10 | 3.58E-11 | 1.46E-11 |
| *N*-stearoyl leucine | 1.22E-10 | 4.44E-11 | 1.81E-11 | | 1.31E-10 | 2.64E-11 | 1.08E-11 |
| *N*-oleoyl leucine | 1.74E-10 | 6.61E-11 | 2.7E-11 | | 1.68E-10 | 2.65E-11 | 1.08E-11 |
| *N*-linoleoyl leucine | 4.14E-11 | 1.52E-11 | 6.2E-12 | | 4E-11 | 8.16E-12 | 3.33E-12 |
| *N*-docosahexaenoyl leucine | 2.75E-11 | 1.24E-11 | 5.05E-12 | | 2.79E-11 | 6.16E-12 | 2.51E-12 |
| ***N*-acyl methionine** |  |  |  | |  |  |  |
| *N*-palmitoyl methionine | 1.87E-10 | 1.01E-10 | 4.13E-11 | | 1.78E-10 | 8.05E-11 | 3.29E-11 |
| *N*-stearoyl methionine | 3.42E-11 | 2.27E-11 | 9.28E-12 | | 2.89E-11 | 8.88E-12 | 3.62E-12 |
| *N*-oleoyl methionine | 4.31E-11 | 2.08E-11 | 8.5E-12 | | 4.1E-11 | 1.21E-11 | 4.94E-12 |
| *N*-linoleoyl methionine | PISSR |  |  | | PISSR |  |  |
| *N*-arachidonoyl methionine | PISSR |  |  | | PISSR |  |  |
| *N*-docosahexaenoyl methionine | BDL |  |  | | BDL |  |  |
| ***N*-acyl phenylalanine** |  |  |  | |  |  |  |
| *N*-palmitoyl phenylalanine | 1.4E-10 | 2.09E-11 | 8.55E-12 | | 1.59E-10 | 3.47E-11 | 1.42E-11 |
| *N*-stearoyl phenylalanine | 8.37E-11 | 2.85E-11 | 1.16E-11 | | 8.93E-11 | 1.58E-11 | 6.45E-12 |
| *N*-oleoyl phenylalanine | 9.2E-11 | 3.39E-11 | 1.38E-11 | | 9.02E-11 | 1.71E-11 | 6.96E-12 |
| *N*-linoleoyl phenylalanine | 1.33E-11 | 4.61E-12 | 1.88E-12 | | 1.41E-11 | 5.96E-12 | 2.44E-12 |
| *N*-arachidonoyl phenylalanine | 2.8E-11 | 5.1E-12 | 2.08E-12 | | 4.17E-11 | 5.64E-12 | 2.3E-12 |
| *N*-docosahexaenoyl phenylalanine | 1.64E-11 | 6.65E-12 | 2.72E-12 | | 1.68E-11 | 5.36E-12 | 2.19E-12 |

**Supplemental Table 1: Continued**

|  | BV2 | | | | | |
| --- | --- | --- | --- | --- | --- | --- |
|  | Vehicle | | | 1 µM THC | | |
| ***N*-acyl proline** | Mean | Std Dev | Std Error | Mean | Std Dev | Std Error |
| *N*-palmitoyl proline | 1.47E-11 | 6.02E-12 | 2.46E-12 | 1.86E-11 | 5.95E-12 | 2.43E-12 |
| *N*-stearoyl proline | 6.12E-12 | 2.4E-12 | 9.79E-13 | 6.98E-12 | 2.16E-12 | 8.83E-13 |
| *N-*oleoyl proline | 5.63E-12 | 2.47E-12 | 1.01E-12 | 5.62E-12 | 1.12E-12 | 4.56E-13 |
| *N*-linoleoyl proline | BDL |  |  | BDL |  |  |
| *N*-arachidonoyl proline | BDL |  |  | BDL |  |  |
| *N*-docosahexaenoyl proline | BDL |  |  | BDL |  |  |
| ***N*-acyl serine** |  |  |  |  |  |  |
| *N*-palmitoyl serine | 1.93E-10 | 7.61E-11 | 3.11E-11 | 1.79E-10 | 4.52E-11 | 1.84E-11 |
| *N*-stearoyl serine | 9.29E-11 | 1.57E-11 | 6.4E-12 | 9.83E-11 | 3.59E-11 | 1.46E-11 |
| *N*-oleoyl serine | 1.35E-08 | 6.28E-10 | 2.56E-10 | 1.84E-08 | 2.57E-09 | 1.05E-09 |
| *N*-linoleoyl serine | 2.77E-09 | 2.32E-10 | 9.47E-11 | 3.33E-09 | 3.72E-10 | 1.52E-10 |
| *N*-arachidonoyl serine | 8.09E-12 | 2.41E-12 | 9.84E-13 | 1.1E-11 | 1.24E-12 | 5.07E-13 |
| *N*-docosahexaenoyl serine | 5.89E-11 | 1.64E-11 | 6.69E-12 | 5.85E-11 | 1.15E-11 | 4.67E-12 |
| ***N*-acyl taurine** |  |  |  |  |  |  |
| *N*-palmitoyl taurine | 6.48E-10 | 1.37E-10 | 5.6E-11 | 4.75E-10 | 1.42E-10 | 5.8E-11 |
| *N*-stearoyl taurine | 7.24E-10 | 1.07E-10 | 4.35E-11 | 6.82E-10 | 1.14E-10 | 4.65E-11 |
| *N*-oleoyl taurine | 2.13E-10 | 9.38E-11 | 3.83E-11 | 2.21E-10 | 5.53E-11 | 2.26E-11 |
| *N*-arachidonoyl taurine | 5.08E-11 | 8.12E-12 | 3.32E-12 | 6.33E-11 | 8.56E-12 | 3.5E-12 |
| ***N*-acyl tryptophan** |  |  |  |  |  |  |
| *N*-palmitoyl tryptophan | 6.7E-11 | 2.48E-11 | 1.01E-11 | 7.25E-11 | 1.25E-11 | 5.09E-12 |
| *N*-stearoyl tryptophan | 1.27E-10 | 3.69E-11 | 1.5E-11 | 1.29E-10 | 2.37E-11 | 9.69E-12 |
| *N*-oleoyl tryptophan | 5.57E-11 | 1.81E-11 | 7.4E-12 | 6.81E-11 | 6.75E-12 | 2.75E-12 |
| *N*-linoleoyl tryptophan | BDL |  |  | BDL |  |  |
| *N*-arachidonoyl tryptophan | PISSR |  |  | PISSR |  |  |
| *N*-docosahexaenoyl tryptophan | PISSR |  |  | PISSR |  |  |
| ***N*-acyl tyrosine** |  |  |  |  |  |  |
| *N*-palmitoyl tyrosine | 5.13E-11 | 1.22E-11 | 4.99E-12 | 5.2E-11 | 8.29E-12 | 3.38E-12 |
| *N*-stearoyl tyrosine | 8.68E-12 | 3.13E-12 | 1.28E-12 | 7.48E-12 | 1.49E-12 | 6.09E-13 |
| *N-*oleoyl tyrosine | 7.29E-11 | 1.98E-11 | 8.06E-12 | 7.8E-11 | 9.57E-12 | 3.91E-12 |
| *N*-linoleoyl tyrosine | 6.65E-12 | 1.9E-12 | 7.77E-13 | 1.09E-11 | 2.84E-12 | 1.16E-12 |
| *N*-arachidonoyl tyrosine | 1.1E-11 | 3.44E-12 | 1.4E-12 | 1.14E-11 | 3.66E-12 | 1.49E-12 |
| *N*-docosahexaenoyl tyrosine | 1E-11 | 3.83E-12 | 1.57E-12 | 1.19E-11 | 3.86E-12 | 1.58E-12 |
| ***N*-acyl valine** |  |  |  |  |  |  |
| *N*-palmitoyl valine | 2.55E-11 | 7.94E-12 | 3.24E-12 | 3.07E-11 | 1.07E-11 | 4.38E-12 |
| *N*- stearoyl valine | 1.4E-11 | 3.55E-12 | 1.45E-12 | 1.68E-11 | 3.95E-12 | 1.61E-12 |
| *N*-oleoyl valine | 1.57E-11 | 4.98E-12 | 2.03E-12 | 1.59E-11 | 5.24E-12 | 2.14E-12 |
| *N*-nervonoyl valine | BDL |  |  | BDL |  |  |
| *N*-linoleoyl valine | PISSR |  |  | PISSR |  |  |
| *N*-docosahexaenoyl valine | PISSR |  |  | PISSR |  |  |
| **2-acyl glycerols** |  |  |  |  |  |  |
| 2-palmitoyl glycerol | 1.17E-09 | 3.52E-10 | 1.44E-10 | 1.02E-09 | 4.72E-10 | 1.93E-10 |
| 2-oleoyl glycerol | 6.48E-09 | 9.91E-10 | 4.04E-10 | 4.31E-09 | 1.21E-09 | 4.95E-10 |
| 2-linoleoyl glycerol | 4.79E-10 | 7.07E-11 | 2.89E-11 | 3.34E-10 | 9.41E-11 | 3.84E-11 |
| 2-arachidonoyl glycerol | 2.19E-09 | 5.08E-10 | 2.07E-10 | 1.79E-09 | 4.41E-10 | 1.8E-10 |
| **Free Fatty Acids** |  |  |  |  |  |  |
| Oleic acid | 1.16E-08 | 5.43E-09 | 2.22E-09 | 1.28E-08 | 1.28E-09 | 5.22E-10 |
| Linoleic acid | 5.01E-09 | 7.19E-10 | 2.94E-10 | 4.55E-09 | 2.61E-10 | 1.06E-10 |
| Arachidonic acid | 1.93E-09 | 2.56E-10 | 1.05E-10 | 2.66E-09 | 3.97E-10 | 1.62E-10 |
| **PhosphoLEA** |  |  |  |  |  |  |
| PhosphoLEA | 2.57E-09 | 5.1E-10 | 2.08E-10 | 3.07E-09 | 8.11E-10 | 3.31E-10 |
| **Prostaglandins** |  |  |  |  |  |  |
| PGE_2_ | 2.71E-09 | 8.04E-10 | 3.28E-10 | 4.28E-09 | 9.47E-10 | 3.86E-10 |
| PGF_2α_ | 8.52E-10 | 9.01E-11 | 3.68E-11 | 1.39E-09 | 1.91E-10 | 7.82E-11 |
| 6-ketoPGF_1α_ | 4.51E-11 | 3.58E-12 | 1.46E-12 | 5.37E-11 | 3.5E-12 | 1.43E-12 |
| **THC/CBD** |  |  |  |  |  |  |
| Cannabidiol | BDL |  |  | BDL |  |  |
| THC | BDL |  |  | 2.53E-07 | 6.26E-08 | 2.56E-08 |
| **THC/CBD** |  |  |  |  |  |  |
| 11-nor-9-carboxyTHC | BDL |  |  | 2.29E-10 | 3.65E-11 | 1.49E-11 |
| 11-OH-THC | BDL |  |  | 5.35E-10 | 9.58E-11 | 3.91E-11 |

**Supplemental Table 2: List of lipids in BV2 microglia cells significantly affected by 1 µM THC**

| BV2 THC Significant Differences in One-Way ANOVA | | | | |
| --- | --- | --- | --- | --- |
| Lipid | F | p | Direction (relative to Veh) | Magnitude (x Veh level) |
| *N*-palmitoyl glycine | 36.43 | .000 | ↑ | 1.75 |
| *N*-stearoyl glycine | 6.04 | .034 | ↑ | 1.42 |
| *N*-linoleoyl glycine | 12.11 | .006 | ↑ | 1.33 |
| *N*-arachidonoyl glycine | 49.32 | .000 | ↑ | 1.57 |
| *N*-docosahexaenoyl glycine | 9.56 | .011 | ↑ | 1.49 |
| *N*-arachidonoyl phenylalanine | 19.50 | .001 | ↑ | 1.49 |
| *N*-oleoyl serine | 21.14 | .001 | ↑ | 1.37 |
| *N*-linoleoyl serine | 9.65 | .011 | ↑ | 1.20 |
| *N*-arachidonoyl serine | 7.04 | .024 | ↑ | 1.36 |
| *N*-palmitoyl taurine | 4.59 | .058 | ↓ | 0.73 |
| *N*-arachidonoyl taurine | 6.72 | .027 | ↑ | 1.25 |
| *N*-linoleoyl tyrosine | 9.18 | .013 | ↑ | 1.64 |
| 2-oleoyl glycerol | 11.54 | .007 | ↓ | 0.66 |
| 2-linoleoyl glycerol | 9.09 | .013 | ↓ | 0.70 |
| Arachidonic acid | 14.16 | .004 | ↑ | 1.39 |
| PGE_2_ | 9.66 | .011 | ↑ | 1.58 |
| PGF_2α_ | 38.81 | .000 | ↑ | 1.63 |
| 6-ketoPGF_1α_ | 17.61 | .002 | ↑ | 1.19 |
| THC | 98.01 | .000 | ↑ | infinite |
| 11-nor-9-carboxyTHC | 235.69 | .000 | ↑ | infinite |
| 11-OH-THC | 187.58 | .000 | ↑ | infinite |

**Supplemental Table 3: Lipid levels in C6 glioma cells treated with Vehicle or 1 µM THC for 2 hours**

|  | C6 | | | | | | |
| --- | --- | --- | --- | --- | --- | --- | --- |
|  | Vehicle | | | 1 µM THC | | | |
| ***N*-acyl alanine** | Mean | Std Dev | Std Error | | Mean | Std Dev | Std Error |
| *N*-palmitoyl alanine | 1.08E-10 | 8.92E-12 | 3.64E-12 | | 1.08E-10 | 7.89E-12 | 3.22E-12 |
| *N*-stearoyl alanine | 1.73E-11 | 2.42E-12 | 9.88E-13 | | 1.49E-11 | 3.76E-12 | 1.53E-12 |
| *N*-oleoyl alanine | 3.15E-11 | 1.73E-12 | 7.08E-13 | | 3.01E-11 | 2.54E-12 | 1.04E-12 |
| *N*-linoleoyl alanine | PISSR |  |  | | PISSR |  |  |
| *N*-arachidonoyl alanine | PISSR |  |  | | PISSR |  |  |
| *N*-docosahexaenoyl alanine | PISSR |  |  | | PISSR |  |  |
| ***N*-acyl dopamine** |  |  |  | |  |  |  |
| *N*-oleoyl dopamine | BDL |  |  | | BDL |  |  |
| *N*-arachidonoyl dopamine | BDL |  |  | | BDL |  |  |
| ***N*-acyl ethanolamine** |  |  |  | |  |  |  |
| *N*-palmitoyl ethanolamine | 2.53E-09 | 6.22E-10 | 2.54E-10 | | 2.75E-09 | 3.75E-10 | 1.53E-10 |
| *N*-stearoyl ethanolamine | 8.71E-10 | 2.83E-10 | 1.16E-10 | | 8.53E-10 | 2.28E-10 | 9.3E-11 |
| *N*-oleoyl ethanolamine | 3.25E-09 | 7.06E-10 | 2.88E-10 | | 3.45E-09 | 4.51E-10 | 1.84E-10 |
| *N*-linoleoyl ethanolamine | 6.94E-10 | 1.3E-10 | 5.32E-11 | | 8.98E-10 | 7.65E-11 | 3.12E-11 |
| *N*-arachidonoyl ethanolamine | 1.45E-10 | 2.89E-11 | 1.18E-11 | | 1.04E-10 | 2.1E-11 | 8.56E-12 |
| *N*-docosahexaenoyl ethanolamine | 1.4E-10 | 3.4E-11 | 1.39E-11 | | 1.38E-10 | 2.57E-11 | 1.05E-11 |
| ***N*-acyl GABA** |  |  |  | |  |  |  |
| *N*-palmitoyl GABA | 2.68E-11 | 9.17E-12 | 3.75E-12 | | 3.36E-11 | 1.29E-11 | 5.28E-12 |
| *N*-stearoyl GABA | PISSR |  |  | | PISSR |  |  |
| *N*-oleoyl GABA | PISSR |  |  | | PISSR |  |  |
| *N*-linoleoyl GABA | PISSR |  |  | | PISSR |  |  |
| *N*-arachidonoyl GABA | 2.53E-11 | 4.31E-12 | 1.76E-12 | | 2.05E-11 | 3.81E-12 | 1.56E-12 |
| *N*-docosahexaenoyl GABA | PISSR |  |  | | PISSR |  |  |
| ***N*-acyl glycine** |  |  |  | |  |  |  |
| *N*-palmitoyl glycine | 1.09E-09 | 3.74E-10 | 1.53E-10 | | 1.12E-09 | 2.37E-10 | 9.67E-11 |
| *N*-stearoyl glycine | 9.32E-10 | 3.31E-10 | 1.35E-10 | | 9.98E-10 | 1.57E-10 | 6.4E-11 |
| *N*-oleoyl glycine | 6.21E-10 | 1.26E-10 | 5.15E-11 | | 6.63E-10 | 5.25E-11 | 2.14E-11 |
| *N*-linoleoyl glycine | 1.72E-10 | 4.86E-11 | 1.98E-11 | | 2.08E-10 | 2.76E-11 | 1.13E-11 |
| *N*-arachidonoyl glycine | 2.81E-10 | 4.93E-11 | 2.01E-11 | | 3.14E-10 | 4.55E-11 | 1.86E-11 |
| *N*-docosahexaenoyl glycine | 4.09E-10 | 6.31E-11 | 2.58E-11 | | 4.49E-10 | 5.07E-11 | 2.07E-11 |
| ***N*-acyl leucine** |  |  |  | |  |  |  |
| *N*-palmitoyl leucine | 5.53E-11 | 3.81E-12 | 1.55E-12 | | 6.2E-11 | 3.97E-12 | 1.62E-12 |
| *N*-stearoyl leucine | 1.31E-11 | 2.66E-12 | 1.09E-12 | | 1.45E-11 | 2.33E-12 | 9.53E-13 |
| *N*-oleoyl leucine | 1.39E-11 | 1.63E-12 | 6.63E-13 | | 1.68E-11 | 2.7E-12 | 1.1E-12 |
| *N*-linoleoyl leucine | PISSR |  |  | | PISSR |  |  |
| *N*-docosahexaenoyl leucine | BDL |  |  | | BDL |  |  |
| ***N*-acyl methionine** |  |  |  | |  |  |  |
| *N*-palmitoyl methionine | 4.7E-10 | 2.03E-10 | 8.3E-11 | | 5.57E-10 | 2.09E-10 | 8.53E-11 |
| *N*-stearoyl methionine | PISSR |  |  | | PISSR |  |  |
| *N*-oleoyl methionine | PISSR |  |  | | PISSR |  |  |
| *N*-linoleoyl methionine | BDL |  |  | | BDL |  |  |
| *N*-arachidonoyl methionine | PISSR |  |  | | PISSR |  |  |
| *N*-docosahexaenoyl methionine | BDL |  |  | | BDL |  |  |
| ***N*-acyl phenylalanine** |  |  |  | |  |  |  |
| *N*-palmitoyl phenylalanine | 1.28E-10 | 1.84E-11 | 7.52E-12 | | 1.58E-10 | 2.56E-11 | 1.05E-11 |
| *N*-stearoyl phenylalanine | 6.08E-11 | 1.66E-11 | 6.76E-12 | | 6.97E-11 | 1.48E-11 | 6.06E-12 |
| *N*-oleoyl phenylalanine | 4.24E-11 | 7.65E-12 | 3.12E-12 | | 5.37E-11 | 8.62E-12 | 3.52E-12 |
| *N*-linoleoyl phenylalanine | 2.45E-11 | 8.91E-12 | 3.64E-12 | | 2.53E-11 | 8.06E-12 | 3.29E-12 |
| *N*-arachidonoyl phenylalanine | 5.26E-11 | 5.35E-12 | 2.18E-12 | | 7.1E-11 | 8.39E-12 | 3.42E-12 |
| *N*-docosahexaenoyl phenylalanine | PISSR |  |  | | PISSR |  |  |

**Supplemental Table 3: Continued**

|  | C6 | | | | | |
| --- | --- | --- | --- | --- | --- | --- |
|  | Vehicle | | | 1 µM THC | | |
| ***N*-acyl proline** | Mean | Std Dev | Std Error | Mean | Std Dev | Std Error |
| *N*-palmitoyl proline | 5.83E-11 | 1.16E-11 | 4.73E-12 | 5.46E-11 | 1.38E-11 | 5.64E-12 |
| *N*-stearoyl proline | 1.63E-11 | 3.17E-12 | 1.29E-12 | 1.45E-11 | 1.66E-12 | 6.78E-13 |
| *N-*oleoyl proline | 2.18E-11 | 4.59E-12 | 1.87E-12 | 2.04E-11 | 6.98E-12 | 2.85E-12 |
| *N*-linoleoyl proline | BDL |  |  | BDL |  |  |
| *N*-arachidonoyl proline | BDL |  |  | BDL |  |  |
| *N*-docosahexaenoyl proline | BDL |  |  | BDL |  |  |
| ***N*-acyl serine** |  |  |  |  |  |  |
| *N*-palmitoyl serine | 1.41E-09 | 1.18E-10 | 4.81E-11 | 1.48E-09 | 8.42E-11 | 3.44E-11 |
| *N*-stearoyl serine | 3.78E-09 | 4.11E-10 | 1.68E-10 | 3.9E-09 | 1.91E-10 | 7.8E-11 |
| *N*-oleoyl serine | 9.78E-09 | 7.88E-10 | 3.22E-10 | 1.01E-08 | 3.77E-10 | 1.54E-10 |
| *N*-linoleoyl serine | 5.39E-09 | 5.48E-10 | 2.24E-10 | 5.67E-09 | 3.08E-10 | 1.26E-10 |
| *N*-arachidonoyl serine | PISSR |  |  | PISSR |  |  |
| *N*-docosahexaenoyl serine | 7.8E-10 | 1.91E-10 | 7.81E-11 | 8.16E-10 | 2.73E-10 | 1.12E-10 |
| ***N*-acyl taurine** |  |  |  |  |  |  |
| *N*-palmitoyl taurine | 8.71E-10 | 1.4E-10 | 5.73E-11 | 7.28E-10 | 1.95E-10 | 7.97E-11 |
| *N*-stearoyl taurine | 1.11E-09 | 1.25E-10 | 5.11E-11 | 1.16E-09 | 1.63E-10 | 6.67E-11 |
| *N*-oleoyl taurine | 3.47E-10 | 6.92E-11 | 2.82E-11 | 2.5E-10 | 7.65E-11 | 3.12E-11 |
| *N*-arachidonoyl taurine | 5.64E-11 | 4.86E-12 | 1.98E-12 | 4.97E-11 | 5.89E-12 | 2.41E-12 |
| ***N*-acyl tryptophan** |  |  |  |  |  |  |
| *N*-palmitoyl tryptophan | 6.58E-11 | 1.07E-11 | 4.35E-12 | 6.74E-11 | 1.14E-11 | 4.66E-12 |
| *N*-stearoyl tryptophan | 5.04E-11 | 1.15E-11 | 4.68E-12 | 4.74E-11 | 1.48E-11 | 6.05E-12 |
| *N*-oleoyl tryptophan | PISSR |  |  | PISSR |  |  |
| *N*-linoleoyl tryptophan | BDL |  |  | BDL |  |  |
| *N*-arachidonoyl tryptophan | PISSR |  |  | PISSR |  |  |
| *N*-docosahexaenoyl tryptophan | PISSR |  |  | PISSR |  |  |
| ***N*-acyl tyrosine** |  |  |  |  |  |  |
| *N*-palmitoyl tyrosine | 3.82E-11 | 5.27E-12 | 2.15E-12 | 4.04E-11 | 5.34E-12 | 2.18E-12 |
| *N*-stearoyl tyrosine | 8.53E-12 | 3.76E-12 | 1.54E-12 | 9.33E-12 | 3.02E-12 | 1.23E-12 |
| *N-*oleoyl tyrosine | 2.06E-11 | 6.89E-12 | 2.81E-12 | 1.84E-11 | 6.36E-12 | 2.6E-12 |
| *N*-linoleoyl tyrosine | BDL |  |  | BDL |  |  |
| *N*-arachidonoyl tyrosine | PISSR |  |  | PISSR |  |  |
| *N*-docosahexaenoyl tyrosine | PISSR |  |  | PISSR |  |  |
| ***N*-acyl valine** |  |  |  |  |  |  |
| *N*-palmitoyl valine | 2.39E-11 | 5.93E-12 | 2.42E-12 | 2.33E-11 | 5.41E-12 | 2.21E-12 |
| *N*- stearoyl valine | 1.47E-11 | 1.41E-12 | 5.76E-13 | 1.22E-11 | 4.27E-12 | 1.75E-12 |
| *N*-oleoyl valine | 6.3E-12 | 1.79E-12 | 7.31E-13 | 4.6E-12 | 2.61E-12 | 1.06E-12 |
| *N*-nervonoyl valine | BDL |  |  | BDL |  |  |
| *N*-linoleoyl valine | PISSR |  |  | PISSR |  |  |
| *N*-docosahexaenoyl valine | PISSR |  |  | PISSR |  |  |
| **2-acyl glycerols** |  |  |  |  |  |  |
| 2-palmitoyl glycerol | 1.2E-07 | 3.09E-08 | 1.26E-08 | 8.82E-08 | 2.99E-08 | 1.22E-08 |
| 2-oleoyl glycerol | 2.45E-08 | 5.05E-09 | 2.06E-09 | 1.63E-08 | 2.44E-09 | 9.96E-10 |
| 2-linoleoyl glycerol | 3.5E-08 | 1.99E-09 | 8.11E-10 | 3.12E-08 | 9.49E-10 | 3.87E-10 |
| 2-arachidonoyl glycerol | 1.4E-08 | 1.5E-09 | 6.12E-10 | 1.1E-08 | 1.46E-09 | 5.95E-10 |
| **Free Fatty Acids** |  |  |  |  |  |  |
| Oleic acid | 1.54E-08 | 2.86E-09 | 1.17E-09 | 1.61E-08 | 3.3E-09 | 1.35E-09 |
| Linoleic acid | 1.24E-08 | 2.13E-09 | 8.7E-10 | 1.4E-08 | 1.89E-09 | 7.73E-10 |
| Arachidonic acid | 6.99E-09 | 8.4E-10 | 3.43E-10 | 8.14E-09 | 8.55E-10 | 3.49E-10 |
| **PhosphoLEA** |  |  |  |  |  |  |
| PhosphoLEA | BDL |  |  | BDL |  |  |
| **Prostaglandins** |  |  |  |  |  |  |
| PGE_2_ | BDL |  |  | BDL |  |  |
| PGF_2α_ | BDL |  |  | BDL |  |  |
| 6-ketoPGF_1α_ | PISSR |  |  | PISSR |  |  |
| **THC/CBD** |  |  |  |  |  |  |
| Cannabidiol | BDL |  |  | BDL |  |  |
| THC | BDL |  |  | 4.8E-08 | 7.34E-09 | 3E-09 |
| **THC/CBD** |  |  |  |  |  |  |
| 11-nor-9-carboxyTHC | BDL |  |  | 4.57E-10 | 1.35E-10 | 5.5E-11 |
| 11-OH-THC | BDL |  |  | 1.6E-09 | 2.12E-10 | 8.67E-11 |

**Supplemental Table 4: List of lipids in C6 glioma cells significantly affected by 1 µM THC**

| C6 THC Significant Differences in One-Way ANOVA | | | | |
| --- | --- | --- | --- | --- |
| Lipid | F | p | Direction (relative to Veh) | Magnitude (x Veh level) |
| *N*-linoleoyl ethanolamine | 10.94 | .008 | ↑ | 1.29 |
| *N*-arachidonoyl ethanolamine | 7.84 | .019 | ↓ | 0.72 |
| *N*-arachidonoyl GABA | 4.26 | .066 | ↓ | 0.81 |
| *N*-palmitoyl leucine | 8.75 | .014 | ↑ | 1.12 |
| *N*-oleoyl leucine | 4.92 | .051 | ↑ | 1.20 |
| *N*-palmitoyl phenylalanine | 5.31 | .044 | ↑ | 1.23 |
| *N*-oleoyl phenylalanine | 5.76 | .037 | ↑ | 1.27 |
| *N*-arachidonoyl phenylalanine | 20.36 | .001 | ↑ | 1.35 |
| *N*-oleoyl taurine | 5.38 | .043 | ↓ | 0.72 |
| *N*-arachidonoyl taurine | 4.69 | .056 | ↓ | 0.88 |
| 2-palmitoyl glycerol | 3.35 | .097 | ↓ | 0.73 |
| 2-oleoyl glycerol | 12.66 | .005 | ↓ | 0.66 |
| 2-linoleoyl glycerol | 17.72 | .002 | ↓ | 0.89 |
| 2-arachidonoyl glycerol | 12.02 | .006 | ↓ | 0.79 |
| Arachidonic acid | 5.56 | .040 | ↑ | 1.17 |
| THC | 256.49 | .000 | ↑ | infinite |
| 11-nor-9-carboxyTHC | 69.03 | .000 | ↑ | infinite |
| 11-OH-THC | 340.44 | .000 | ↑ | infinite |

**Supplemental Table 5: Lipid levels in N18 neuroblastoma cells treated with Vehicle or 1 µM THC for 2 hours**

|  | N18 | | | | | | |
| --- | --- | --- | --- | --- | --- | --- | --- |
|  | Vehicle | | | 1 µM THC | | | |
| ***N*-acyl alanine** | Mean | Std Dev | Std Error | | Mean | Std Dev | Std Error |
| *N*-palmitoyl alanine | 7.14E-11 | 7.09E-12 | 2.89E-12 | | 6.9E-11 | 1E-11 | 4.1E-12 |
| *N*-stearoyl alanine | 7.66E-11 | 2.17E-11 | 8.86E-12 | | 7.58E-11 | 1.22E-11 | 4.98E-12 |
| *N*-oleoyl alanine | 3.24E-11 | 3.96E-12 | 1.61E-12 | | 3.31E-11 | 6.11E-12 | 2.49E-12 |
| *N*-linoleoyl alanine | PISSR |  |  | | PISSR |  |  |
| *N*-arachidonoyl alanine | PISSR |  |  | | PISSR |  |  |
| *N*-docosahexaenoyl alanine | PISSR |  |  | | PISSR |  |  |
| ***N*-acyl dopamine** |  |  |  | |  |  |  |
| *N*-oleoyl dopamine | BDL |  |  | | BDL |  |  |
| *N*-arachidonoyl dopamine | BDL |  |  | | BDL |  |  |
| ***N*-acyl ethanolamine** |  |  |  | |  |  |  |
| *N*-palmitoyl ethanolamine | 1.16E-10 | 7.61E-12 | 3.11E-12 | | 1.26E-10 | 2.43E-11 | 9.93E-12 |
| *N*-stearoyl ethanolamine | 4.15E-11 | 1.88E-11 | 7.67E-12 | | 4.83E-11 | 1.75E-11 | 7.13E-12 |
| *N*-oleoyl ethanolamine | 2E-10 | 3.19E-11 | 1.3E-11 | | 2.81E-10 | 2.3E-11 | 9.4E-12 |
| *N*-linoleoyl ethanolamine | 6.76E-11 | 9.17E-12 | 3.74E-12 | | 7.12E-11 | 1.19E-11 | 4.87E-12 |
| *N*-arachidonoyl ethanolamine | 6.04E-12 | 6.34E-13 | 2.59E-13 | | 6.89E-12 | 1.35E-12 | 5.52E-13 |
| *N*-docosahexaenoyl ethanolamine | 6.18E-11 | 8E-12 | 3.26E-12 | | 4.09E-11 | 9.71E-12 | 3.96E-12 |
| ***N*-acyl GABA** |  |  |  | |  |  |  |
| *N*-palmitoyl GABA | 3.44E-11 | 1.14E-11 | 4.67E-12 | | 3.46E-11 | 4.11E-12 | 1.68E-12 |
| *N*-stearoyl GABA | 9.11E-11 | 1.62E-11 | 6.61E-12 | | 8.95E-11 | 1.1E-11 | 4.49E-12 |
| *N*-oleoyl GABA | 4.32E-11 | 6.83E-12 | 2.79E-12 | | 5.08E-11 | 9.38E-12 | 3.83E-12 |
| *N*-linoleoyl GABA | PISSR |  |  | | PISSR |  |  |
| *N*-arachidonoyl GABA | PISSR |  |  | | PISSR |  |  |
| *N*-docosahexaenoyl GABA | PISSR |  |  | | PISSR |  |  |
| ***N*-acyl glycine** |  |  |  | |  |  |  |
| *N*-palmitoyl glycine | 4.75E-10 | 3.06E-11 | 1.25E-11 | | 6E-10 | 8.24E-11 | 3.37E-11 |
| *N*-stearoyl glycine | 5.83E-10 | 4.78E-11 | 1.95E-11 | | 6.38E-10 | 6.51E-11 | 2.66E-11 |
| *N*-oleoyl glycine | 3.1E-10 | 7.93E-12 | 3.24E-12 | | 3.55E-10 | 2.8E-11 | 1.15E-11 |
| *N*-linoleoyl glycine | 5.45E-11 | 6.92E-12 | 2.82E-12 | | 6.89E-11 | 6.22E-12 | 2.54E-12 |
| *N*-arachidonoyl glycine | 5.21E-11 | 5.79E-12 | 2.36E-12 | | 6.18E-11 | 7.84E-12 | 3.2E-12 |
| *N*-docosahexaenoyl glycine | 1.66E-11 | 2.18E-12 | 8.89E-13 | | 1.83E-11 | 1.76E-12 | 7.17E-13 |
| ***N*-acyl leucine** |  |  |  | |  |  |  |
| *N*-palmitoyl leucine | 1.71E-11 | 2.11E-11 | 8.61E-12 | | 1.21E-11 | 5.62E-12 | 2.3E-12 |
| *N*-stearoyl leucine | 2.53E-11 | 1.18E-11 | 4.82E-12 | | 2.54E-11 | 9.16E-12 | 3.74E-12 |
| *N*-oleoyl leucine | 4.84E-12 | 2.55E-12 | 1.04E-12 | | 6.55E-12 | 2.75E-12 | 1.12E-12 |
| *N*-linoleoyl leucine | PISSR |  |  | | PISSR |  |  |
| *N*-docosahexaenoyl leucine | PISSR |  |  | | PISSR |  |  |
| ***N*-acyl methionine** |  |  |  | |  |  |  |
| *N*-palmitoyl methionine | 1.38E-11 | 4.44E-12 | 1.81E-12 | | 1.07E-11 | 4.16E-12 | 1.7E-12 |
| *N*-stearoyl methionine | PISSR |  |  | | PISSR |  |  |
| *N*-oleoyl methionine | PISSR |  |  | | PISSR |  |  |
| *N*-linoleoyl methionine | BDL |  |  | | BDL |  |  |
| *N*-arachidonoyl methionine | PISSR |  |  | | PISSR |  |  |
| *N*-docosahexaenoyl methionine | BDL |  |  | | BDL |  |  |
| ***N*-acyl phenylalanine** |  |  |  | |  |  |  |
| *N*-palmitoyl phenylalanine | 1.48E-11 | 2.97E-12 | 1.21E-12 | | 1.49E-11 | 5.06E-12 | 2.07E-12 |
| *N*-stearoyl phenylalanine | 1.72E-11 | 1.04E-11 | 4.26E-12 | | 2E-11 | 7.25E-12 | 2.96E-12 |
| *N*-oleoyl phenylalanine | 4.74E-12 | 2.3E-12 | 9.39E-13 | | 5.54E-12 | 2.51E-12 | 1.02E-12 |
| *N*-linoleoyl phenylalanine | PISSR |  |  | | PISSR |  |  |
| *N*-arachidonoyl phenylalanine | 2.77E-12 | 7E-13 | 2.86E-13 | | 4.38E-12 | 8.34E-13 | 3.41E-13 |
| *N*-docosahexaenoyl phenylalanine | PISSR |  |  | | PISSR |  |  |

**Supplemental Table 5: Continued**

|  | N18 | | | | | |
| --- | --- | --- | --- | --- | --- | --- |
|  | Vehicle | | | 1 µM THC | | |
| ***N*-acyl proline** | Mean | Std Dev | Std Error | Mean | Std Dev | Std Error |
| *N*-palmitoyl proline | 1.08E-12 | 4.08E-13 | 1.67E-13 | 1.35E-12 | 6.57E-13 | 2.68E-13 |
| *N*-stearoyl proline | 1.35E-12 | 3.86E-13 | 1.58E-13 | 1.4E-12 | 3.6E-13 | 1.47E-13 |
| *N-*oleoyl proline | PISSR |  |  | PISSR |  |  |
| *N*-linoleoyl proline | BDL |  |  | BDL |  |  |
| *N*-arachidonoyl proline | BDL |  |  | BDL |  |  |
| *N*-docosahexaenoyl proline | BDL |  |  | BDL |  |  |
| ***N*-acyl serine** |  |  |  |  |  |  |
| *N*-palmitoyl serine | 1.41E-10 | 2.24E-11 | 9.14E-12 | 1.53E-10 | 2.88E-11 | 1.18E-11 |
| *N*-stearoyl serine | 1.73E-10 | 3.22E-11 | 1.32E-11 | 1.8E-10 | 2.9E-11 | 1.19E-11 |
| *N*-oleoyl serine | 5.09E-09 | 8.92E-10 | 3.64E-10 | 5.14E-09 | 6.6E-10 | 2.7E-10 |
| *N*-linoleoyl serine | 1.05E-09 | 2.52E-10 | 1.03E-10 | 1.03E-09 | 1.93E-10 | 7.86E-11 |
| *N*-arachidonoyl serine | 4.04E-11 | 3.99E-12 | 1.63E-12 | 3.01E-11 | 3.3E-12 | 1.35E-12 |
| *N*-docosahexaenoyl serine | 4.65E-11 | 9.33E-12 | 3.81E-12 | 4.5E-11 | 8.5E-12 | 3.47E-12 |
| ***N*-acyl taurine** |  |  |  |  |  |  |
| *N*-palmitoyl taurine | 1.28E-09 | 8.09E-11 | 3.3E-11 | 1.57E-09 | 1.44E-10 | 5.87E-11 |
| *N*-stearoyl taurine | 2.55E-09 | 2.45E-10 | 1E-10 | 3.05E-09 | 4.86E-10 | 1.98E-10 |
| *N*-oleoyl taurine | 5.08E-10 | 6.03E-11 | 2.46E-11 | 6.24E-10 | 6.34E-11 | 2.59E-11 |
| *N*-arachidonoyl taurine | 1.2E-10 | 8.61E-12 | 3.51E-12 | 1.37E-10 | 1.6E-11 | 6.52E-12 |
| ***N*-acyl tryptophan** |  |  |  |  |  |  |
| *N*-palmitoyl tryptophan | BDL |  |  | BDL |  |  |
| *N*-stearoyl tryptophan | 1.54E-11 | 3.11E-12 | 1.27E-12 | 1.47E-11 | 4.6E-12 | 1.88E-12 |
| *N*-oleoyl tryptophan | BDL |  |  | BDL |  |  |
| *N*-linoleoyl tryptophan | BDL |  |  | BDL |  |  |
| *N*-arachidonoyl tryptophan | BDL |  |  | BDL |  |  |
| *N*-docosahexaenoyl tryptophan | BDL |  |  | BDL |  |  |
| ***N*-acyl tyrosine** |  |  |  |  |  |  |
| *N*-palmitoyl tyrosine | 8.63E-11 | 1.16E-11 | 4.72E-12 | 8.8E-11 | 1.08E-11 | 4.4E-12 |
| *N*-stearoyl tyrosine | 1.61E-11 | 1.26E-12 | 5.12E-13 | 1.77E-11 | 2.07E-12 | 8.45E-13 |
| *N-*oleoyl tyrosine | 5.96E-11 | 3.71E-12 | 1.52E-12 | 6.6E-11 | 5.97E-12 | 2.44E-12 |
| *N*-linoleoyl tyrosine | 6.8E-12 | 1.34E-12 | 5.45E-13 | 6.64E-12 | 1.43E-12 | 5.86E-13 |
| *N*-arachidonoyl tyrosine | 2.01E-11 | 1.57E-12 | 6.42E-13 | 2.51E-11 | 2.11E-12 | 8.61E-13 |
| *N*-docosahexaenoyl tyrosine | 1.2E-11 | 3.16E-12 | 1.29E-12 | 1.28E-11 | 2.5E-12 | 1.02E-12 |
| ***N*-acyl valine** |  |  |  |  |  |  |
| *N*-palmitoyl valine | 2.85E-12 | 2.55E-12 | 1.04E-12 | 3.11E-12 | 1.28E-12 | 5.21E-13 |
| *N*- stearoyl valine | 1.67E-11 | 9.35E-12 | 3.82E-12 | 1.99E-11 | 8.5E-12 | 3.47E-12 |
| *N*-oleoyl valine | 3.98E-12 | 6.38E-12 | 2.6E-12 | 2.22E-12 | 1.02E-12 | 4.16E-13 |
| *N*-nervonoyl valine | BDL |  |  | BDL |  |  |
| *N*-linoleoyl valine | BDL |  |  | BDL |  |  |
| *N*-docosahexaenoyl valine | BDL |  |  | BDL |  |  |
| **2-acyl glycerols** |  |  |  |  |  |  |
| 2-palmitoyl glycerol | 5.29E-10 | 8.92E-11 | 3.99E-11 | 6.24E-10 | 1.19E-10 | 4.88E-11 |
| 2-oleoyl glycerol | 2.33E-09 | 3.26E-10 | 1.46E-10 | 2.97E-09 | 3E-10 | 1.23E-10 |
| 2-linoleoyl glycerol | 2.62E-10 | 4.02E-11 | 1.64E-11 | 3.48E-10 | 8.45E-11 | 3.45E-11 |
| 2-arachidonoyl glycerol | 2.35E-09 | 4.3E-10 | 1.76E-10 | 1.94E-09 | 2.61E-10 | 1.06E-10 |
| **Free Fatty Acids** |  |  |  |  |  |  |
| Oleic acid | 1.47E-08 | 3.48E-09 | 1.42E-09 | 1.5E-08 | 4.31E-09 | 1.76E-09 |
| Linoleic acid | 7.19E-09 | 9.06E-10 | 3.7E-10 | 9.2E-09 | 9.68E-10 | 3.95E-10 |
| Arachidonic acid | 6.68E-09 | 6.52E-10 | 2.66E-10 | 8.16E-09 | 7.05E-10 | 2.88E-10 |
| **PhosphoLEA** |  |  |  |  |  |  |
| PhosphoLEA | 1.69E-09 | 2.31E-10 | 9.42E-11 | 2.37E-09 | 2.42E-10 | 9.87E-11 |
| **Prostaglandins** |  |  |  |  |  |  |
| PGE_2_ | 1.07E-10 | 2.09E-11 | 8.53E-12 | 1.59E-10 | 2.68E-11 | 1.09E-11 |
| PGF_2α_ | BDL |  |  | BDL |  |  |
| 6-ketoPGF_1α_ | 1.94E-10 | 3.79E-11 | 1.55E-11 | 2.63E-10 | 3.36E-11 | 1.37E-11 |
| **THC/CBD** |  |  |  |  |  |  |
| Cannabidiol | BDL |  |  | BDL |  |  |
| THC | BDL |  |  | 2.28E-08 | 2.02E-09 | 8.23E-10 |
| **THC/CBD** |  |  |  |  |  |  |
| 11-nor-9-carboxyTHC | BDL |  |  | 4.62E-10 | 9.89E-11 | 4.04E-11 |
| 11-OH-THC | BDL |  |  | 4.65E-10 | 5.67E-11 | 2.31E-11 |

**Supplemental Table 6: List of lipids in N18 neuroblastoma cells significantly affected by 1 µM THC**

| N18 THC Significant Differences in One-Way ANOVA | | | | |
| --- | --- | --- | --- | --- |
| Lipid | F | p | Direction (relative to Veh) | Magnitude (x Veh level) |
| *N*-oleoyl ethanolamine | 25.44 | .001 | ↑ | 1.41 |
| *N*-docosahexaenoyl ethanolamine | 16.56 | .002 | ↓ | 0.66 |
| *N*-palmitoyl glycine | 12.15 | .006 | ↑ | 1.26 |
| *N*-oleoyl glycine | 14.13 | .004 | ↑ | 1.15 |
| *N*-linoleoyl glycine | 14.40 | .004 | ↑ | 1.26 |
| *N*-arachidonoyl glycine | 5.95 | .035 | ↑ | 1.19 |
| *N*-arachidonoyl phenylalanine | 13.02 | .005 | ↑ | 1.58 |
| *N*-arachidonoyl serine | 24.12 | .001 | ↓ | 0.74 |
| *N*-palmitoyl taurine | 18.34 | .002 | ↑ | 1.23 |
| *N*-stearoyl taurine | 5.12 | .047 | ↑ | 1.20 |
| *N*-oleoyl taurine | 10.54 | .009 | ↑ | 1.23 |
| *N*-arachidonoyl taurine | 5.22 | .041 | ↑ | 1.14 |
| *N*-oleoyl tyrosine | 5.05 | .048 | ↑ | 1.11 |
| *N*-arachidonoyl tyrosine | 21.93 | .001 | ↑ | 1.26 |
| 2-oleoyl glycerol | 11.24 | .008 | ↑ | 1.27 |
| 2-linoleoyl glycerol | 5.03 | .049 | ↑ | 1.33 |
| 2-arachidonoyl glycerol | 3.93 | .075 | ↓ | 0.83 |
| Linoleic acid | 13.81 | .004 | ↑ | 1.28 |
| Arachidonic acid | 14.34 | .004 | ↑ | 1.22 |
| phosphoLEA | 25.05 | .001 | ↑ | 1.40 |
| PGE_2_ | 14.15 | .004 | ↑ | 1.49 |
| 6-ketoPGF_1α_ | 11.28 | .007 | ↑ | 1.36 |
| THC | 765.90 | .000 | ↑ | infinite |
| 11-nor-9-carboxyTHC | 130.76 | .000 | ↑ | infinite |
| 11-OH-THC | 403.80 | .000 | ↑ | infinite |

**Supplemental Table 7: Lipid levels in BV2 microglial cells treated with Vehicle or 1 µM CBD for 2 hours**

|  | BV2 | | | | | | |
| --- | --- | --- | --- | --- | --- | --- | --- |
|  | Vehicle | | | 1 µM CBD | | | |
| ***N*-acyl alanine** | Mean | Std Dev | Std Error | | Mean | Std Dev | Std Error |
| *N*-palmitoyl alanine | 1.07E-10 | 1.99E-11 | 8.13E-12 | | 9.89E-11 | 1.62E-11 | 6.6E-12 |
| *N*-stearoyl alanine | 1.33E-10 | 1.76E-11 | 7.2E-12 | | 1.24E-10 | 1.76E-11 | 7.2E-12 |
| *N*-oleoyl alanine | 7.04E-11 | 1.26E-11 | 5.13E-12 | | 6.75E-11 | 8.25E-12 | 3.37E-12 |
| *N*-linoleoyl alanine | PISSR |  |  | | PISSR |  |  |
| *N*-arachidonoyl alanine | PISSR |  |  | | PISSR |  |  |
| *N*-docosahexaenoyl alanine | BDL |  |  | | BDL |  |  |
| ***N*-acyl dopamine** |  |  |  | |  |  |  |
| *N*-oleoyl dopamine | BDL |  |  | | BDL |  |  |
| *N*-arachidonoyl dopamine | BDL |  |  | | BDL |  |  |
| ***N*-acyl ethanolamine** |  |  |  | |  |  |  |
| *N*-palmitoyl ethanolamine | 1.74E-11 | 2.7E-12 | 1.1E-12 | | 2.17E-11 | 3.78E-12 | 1.54E-12 |
| *N*-stearoyl ethanolamine | 1.47E-12 | 5.78E-13 | 2.36E-13 | | 1.62E-12 | 7.4E-13 | 3.02E-13 |
| *N*-oleoyl ethanolamine | 4.05E-11 | 6.77E-12 | 2.76E-12 | | 5.63E-11 | 1.24E-11 | 5.04E-12 |
| *N*-linoleoyl ethanolamine | 1.16E-11 | 1.9E-12 | 7.76E-13 | | 1.62E-11 | 2.23E-12 | 9.1E-13 |
| *N*-arachidonoyl ethanolamine | 1.13E-11 | 4.68E-13 | 1.91E-13 | | 1.33E-11 | 1.29E-12 | 5.28E-13 |
| *N*-docosahexaenoyl ethanolamine | 2.83E-11 | 1.12E-11 | 4.56E-12 | | 3.87E-11 | 5.64E-12 | 2.3E-12 |
| ***N*-acyl GABA** |  |  |  | |  |  |  |
| *N*-palmitoyl GABA | 1.86E-11 | 1.17E-12 | 4.78E-13 | | 2.18E-11 | 7.88E-12 | 3.22E-12 |
| *N*-stearoyl GABA | 2.31E-11 | 2.35E-12 | 9.6E-13 | | 2.19E-11 | 6.1E-12 | 2.49E-12 |
| *N*-oleoyl GABA | 7.59E-12 | 1.34E-12 | 5.48E-13 | | 7.68E-12 | 1.44E-12 | 5.89E-13 |
| *N*-linoleoyl GABA | PISSR |  |  | | PISSR |  |  |
| *N*-arachidonoyl GABA | PISSR |  |  | | PISSR |  |  |
| *N*-docosahexaenoyl GABA | PISSR |  |  | | PISSR |  |  |
| ***N*-acyl glycine** |  |  |  | |  |  |  |
| *N*-palmitoyl glycine | 1.25E-09 | 2.03E-10 | 8.3E-11 | | 1.23E-09 | 2.69E-10 | 1.1E-10 |
| *N*-stearoyl glycine | 6.18E-10 | 7.67E-11 | 3.13E-11 | | 6.6E-10 | 9.69E-11 | 3.96E-11 |
| *N*-oleoyl glycine | 5.39E-10 | 6.66E-11 | 2.72E-11 | | 5.78E-10 | 6.66E-11 | 2.72E-11 |
| *N*-linoleoyl glycine | 1.94E-10 | 1.98E-11 | 8.1E-12 | | 2.36E-10 | 2.62E-11 | 1.07E-11 |
| *N*-arachidonoyl glycine | 1.65E-10 | 9.41E-12 | 3.84E-12 | | 2.1E-10 | 2.55E-11 | 1.04E-11 |
| *N*-docosahexaenoyl glycine | 1.51E-10 | 1.47E-11 | 5.99E-12 | | 1.7E-10 | 4.2E-11 | 1.72E-11 |
| ***N*-acyl leucine** |  |  |  | |  |  |  |
| *N*-palmitoyl leucine | 3.11E-11 | 4.46E-12 | 1.82E-12 | | 3.08E-11 | 4.76E-12 | 1.94E-12 |
| *N*-stearoyl leucine | 2E-11 | 3E-12 | 1.23E-12 | | 2.66E-11 | 3.61E-12 | 1.48E-12 |
| *N*-oleoyl leucine | 3.43E-11 | 3.69E-12 | 1.5E-12 | | 4.14E-11 | 5.59E-12 | 2.28E-12 |
| *N*-linoleoyl leucine | 4.22E-12 | 1.2E-12 | 4.91E-13 | | 4.34E-12 | 6.49E-13 | 2.65E-13 |
| *N*-docosahexaenoyl leucine | 5.94E-12 | 1.22E-12 | 5E-13 | | 7.13E-12 | 1.26E-12 | 5.16E-13 |
| ***N*-acyl methionine** |  |  |  | |  |  |  |
| *N*-palmitoyl methionine | 1.39E-09 | 4.87E-10 | 1.99E-10 | | 1.29E-09 | 7.11E-10 | 2.9E-10 |
| *N*-stearoyl methionine | 2.86E-11 | 5.6E-12 | 2.29E-12 | | 2.54E-11 | 5.38E-12 | 2.2E-12 |
| *N*-oleoyl methionine | 3.09E-11 | 9.35E-12 | 3.82E-12 | | 2.83E-11 | 5.11E-12 | 2.09E-12 |
| *N*-linoleoyl methionine | PISSR |  |  | | PISSR |  |  |
| *N*-arachidonoyl methionine | 2.74E-11 | 7.45E-12 | 3.04E-12 | | 2.87E-11 | 5.47E-12 | 2.23E-12 |
| *N*-docosahexaenoyl methionine | PISSR |  |  | | PISSR |  |  |
| ***N*-acyl phenylalanine** |  |  |  | |  |  |  |
| *N*-palmitoyl phenylalanine | 1.07E-10 | 2.7E-11 | 1.1E-11 | | 1.07E-10 | 2.41E-11 | 9.84E-12 |
| *N*-stearoyl phenylalanine | 2.69E-11 | 5.87E-12 | 2.39E-12 | | 2.96E-11 | 5.29E-12 | 2.16E-12 |
| *N*-oleoyl phenylalanine | 1.97E-11 | 2.06E-12 | 8.41E-13 | | 1.91E-11 | 3.84E-12 | 1.57E-12 |
| *N*-linoleoyl phenylalanine | PISSR |  |  | | PISSR |  |  |
| *N*-arachidonoyl phenylalanine | 1.03E-10 | 1.6E-11 | 6.51E-12 | | 1.08E-10 | 1.18E-11 | 4.82E-12 |
| *N*-docosahexaenoyl phenylalanine | PISSR |  |  | | PISSR |  |  |

**Supplemental Table 7: Continued**

|  | BV2 | | | | | |
| --- | --- | --- | --- | --- | --- | --- |
|  | Vehicle | | | 1 µM CBD | | |
| ***N*-acyl proline** | Mean | Std Dev | Std Error | Mean | Std Dev | Std Error |
| *N*-palmitoyl proline | 2.79E-11 | 3.01E-12 | 1.23E-12 | 2.48E-11 | 4.64E-12 | 1.9E-12 |
| *N*-stearoyl proline | 1.51E-11 | 1.77E-12 | 7.23E-13 | 1.49E-11 | 2.74E-12 | 1.12E-12 |
| *N-*oleoyl proline | 1.44E-11 | 2.49E-12 | 1.02E-12 | 1.43E-11 | 2.3E-12 | 9.37E-13 |
| *N*-linoleoyl proline | PISSR |  |  | PISSR |  |  |
| *N*-arachidonoyl proline | PISSR |  |  | PISSR |  |  |
| *N*-docosahexaenoyl proline | PISSR |  |  | PISSR |  |  |
| ***N*-acyl serine** |  |  |  |  |  |  |
| *N*-palmitoyl serine | 6.18E-10 | 9.67E-11 | 3.95E-11 | 5.61E-10 | 1.04E-10 | 4.26E-11 |
| *N*-stearoyl serine | 3.61E-10 | 1.07E-10 | 4.38E-11 | 3.62E-10 | 9.58E-11 | 3.91E-11 |
| *N*-oleoyl serine | 4.34E-10 | 6.97E-11 | 2.84E-11 | 5.51E-10 | 8.78E-11 | 3.58E-11 |
| *N*-linoleoyl serine | 2.74E-10 | 5.61E-11 | 2.29E-11 | 3.18E-10 | 2.81E-11 | 1.15E-11 |
| *N*-arachidonoyl serine | 5.86E-11 | 1.83E-11 | 7.46E-12 | 6.47E-11 | 2.19E-11 | 8.92E-12 |
| *N*-docosahexaenoyl serine | 1.55E-10 | 8.35E-11 | 3.41E-11 | 2.27E-10 | 9.83E-11 | 4.01E-11 |
| ***N*-acyl taurine** |  |  |  |  |  |  |
| *N*-palmitoyl taurine | 1.37E-09 | 2.66E-10 | 1.08E-10 | 1.42E-09 | 3.14E-10 | 1.28E-10 |
| *N*-stearoyl taurine | 1.13E-09 | 1.83E-10 | 7.46E-11 | 1.17E-09 | 1.92E-10 | 7.85E-11 |
| *N*-oleoyl taurine | 1.51E-09 | 3.76E-10 | 1.54E-10 | 1.57E-09 | 3.01E-10 | 1.23E-10 |
| *N*-arachidonoyl taurine | 2.08E-10 | 3.77E-11 | 1.54E-11 | 2.91E-10 | 2.87E-11 | 1.17E-11 |
| ***N*-acyl tryptophan** |  |  |  |  |  |  |
| *N*-palmitoyl tryptophan | PISSR |  |  | PISSR |  |  |
| *N*-stearoyl tryptophan | PISSR |  |  | PISSR |  |  |
| *N*-oleoyl tryptophan | PISSR |  |  | PISSR |  |  |
| *N*-linoleoyl tryptophan | BDL |  |  | BDL |  |  |
| *N*-arachidonoyl tryptophan | BDL |  |  | BDL |  |  |
| *N*-docosahexaenoyl tryptophan | BDL |  |  | BDL |  |  |
| ***N*-acyl tyrosine** |  |  |  |  |  |  |
| *N*-palmitoyl tyrosine | 4.55E-11 | 6.84E-12 | 2.79E-12 | 4.54E-11 | 6.33E-12 | 2.58E-12 |
| *N*-stearoyl tyrosine | 8.65E-12 | 9.45E-13 | 3.86E-13 | 8.53E-12 | 1.02E-12 | 4.18E-13 |
| *N-*oleoyl tyrosine | 4.56E-11 | 7.67E-12 | 3.13E-12 | 4.74E-11 | 8.88E-12 | 3.62E-12 |
| *N*-linoleoyl tyrosine | PISSR |  |  | PISSR |  |  |
| *N*-arachidonoyl tyrosine | 1.59E-11 | 1.82E-12 | 7.44E-13 | 1.4E-11 | 2.2E-12 | 8.96E-13 |
| *N*-docosahexaenoyl tyrosine | PISSR |  |  | PISSR |  |  |
| ***N*-acyl valine** |  |  |  |  |  |  |
| *N*-palmitoyl valine | 1.14E-11 | 3.19E-12 | 1.3E-12 | 9.2E-12 | 2.96E-12 | 1.21E-12 |
| *N*- stearoyl valine | 8.64E-12 | 2.99E-12 | 1.22E-12 | 1.07E-11 | 1.88E-12 | 7.68E-13 |
| *N*-oleoyl valine | 6.94E-12 | 1.41E-12 | 5.77E-13 | 5.74E-12 | 1.8E-12 | 7.34E-13 |
| *N*-nervonoyl valine | BDL |  |  | BDL |  |  |
| *N*-linoleoyl valine | PISSR |  |  | PISSR |  |  |
| *N*-docosahexaenoyl valine | BDL |  |  | BDL |  |  |
| **2-acyl glycerols** |  |  |  |  |  |  |
| 2-palmitoyl glycerol | 1.7E-09 | 4.2E-10 | 1.71E-10 | 1.9E-09 | 6.85E-10 | 2.79E-10 |
| 2-oleoyl glycerol | 3.08E-09 | 4.97E-10 | 2.03E-10 | 3.52E-09 | 6.12E-10 | 2.5E-10 |
| 2-linoleoyl glycerol | 5.59E-10 | 4.97E-11 | 2.03E-11 | 6.34E-10 | 7.27E-11 | 2.97E-11 |
| 2-arachidonoyl glycerol | 9.82E-10 | 6.02E-11 | 2.46E-11 | 1.03E-09 | 1.27E-10 | 5.17E-11 |
| **Free Fatty Acids** |  |  |  |  |  |  |
| Oleic acid | 5.52E-09 | 1.13E-09 | 4.63E-10 | 4.81E-09 | 1.05E-09 | 4.28E-10 |
| Linoleic acid | 2.99E-09 | 4.15E-10 | 1.69E-10 | 3.02E-09 | 7.12E-10 | 2.91E-10 |
| Arachidonic acid | 3.47E-09 | 4.86E-10 | 1.99E-10 | 2.58E-09 | 3.4E-10 | 1.39E-10 |
| **PhosphoLEA** |  |  |  |  |  |  |
| PhosphoLEA | 5.92E-10 | 8.72E-11 | 3.56E-11 | 7.68E-10 | 1.1E-10 | 4.5E-11 |
| **Prostaglandins** |  |  |  |  |  |  |
| PGE_2_ | 1.03E-08 | 1.62E-09 | 6.61E-10 | 8.31E-09 | 1.14E-09 | 4.64E-10 |
| PGF_2α_ | 2.12E-09 | 3.11E-10 | 1.27E-10 | 1.37E-09 | 3.15E-10 | 1.29E-10 |
| 6-ketoPGF_1α_ | 4.4E-10 | 1.31E-10 | 5.33E-11 | 4.12E-10 | 1.08E-10 | 4.4E-11 |
| **THC/CBD** |  |  |  |  |  |  |
| Cannabidiol | BDL |  |  | 1.79E-07 | 4.18E-08 | 1.71E-08 |
| THC | BDL |  |  | BDL |  |  |
| **THC Metabolites** |  |  |  |  |  |  |
| 11-nor-9-carboxyTHC | BDL |  |  | BDL |  |  |
| 11-OH-THC | BDL |  |  | BDL |  |  |

**Supplemental Table 8: List of lipids in BV2 microglial cells significantly affected by 1 µM CBD**

| BV2 CBD Significant Differences in One-Way ANOVA | | | | |
| --- | --- | --- | --- | --- |
| Lipid | F | p | Direction (relative to Veh) | Magnitude (x Veh level) |
| *N*-palmitoyl ethanolamine | 5.10 | .047 | ↑ | 1.25 |
| *N*-oleoyl ethanolamine | 7.60 | .020 | ↑ | 1.39 |
| *N*-linoleoyl ethanolamine | 14.50 | .003 | ↑ | 1.40 |
| *N*-arachidonoyl ethanolamine | 12.91 | .005 | ↑ | 1.18 |
| *N*-docosahexaenoyl ethanolamine | 4.16 | .069 | ↑ | 1.37 |
| *N*-linoleoyl glycine | 9.94 | .010 | ↑ | 1.22 |
| *N*-arachidonoyl glycine | 16.96 | .002 | ↑ | 1.27 |
| *N*-stearoyl leucine | 11.84 | .006 | ↑ | 1.33 |
| *N*-oleoyl leucine | 6.87 | .027 | ↑ | 1.21 |
| *N*-oleoyl serine | 6.54 | .029 | ↑ | 1.27 |
| *N*-arachidonoyl taurine | 18.37 | .002 | ↑ | 1.40 |
| 2-linoleoyl glycerol | 4.38 | .063 | ↑ | 1.13 |
| Arachidonic acid | 13.53 | .004 | ↓ | 0.74 |
| phosphoLEA | 9.35 | .012 | ↑ | 1.30 |
| PGE_2_ | 5.99 | .034 | ↓ | 0.81 |
| PGF_2α_ | 17.39 | .002 | ↓ | 0.56 |
| CBD | 109.77 | .000 | ↑ | infinite |

**Supplemental Table 9: Lipid levels in C6 glioma cells treated with Vehicle or 1 µM CBD for 2 hours**

|  | C6 | | | | | | |
| --- | --- | --- | --- | --- | --- | --- | --- |
|  | Vehicle | | | 1 µM CBD | | | |
| ***N*-acyl alanine** | Mean | Std Dev | Std Error | | Mean | Std Dev | Std Error |
| *N*-palmitoyl alanine | 2.76E-10 | 5.9E-11 | 2.41E-11 | | 2.67E-10 | 4.75E-11 | 1.94E-11 |
| *N*-stearoyl alanine | 3.42E-10 | 5.07E-11 | 2.07E-11 | | 3.53E-10 | 5.86E-11 | 2.39E-11 |
| *N*-oleoyl alanine | 1.64E-10 | 2.08E-11 | 8.51E-12 | | 1.68E-10 | 2.53E-11 | 1.03E-11 |
| *N*-linoleoyl alanine | PISSR |  |  | | PISSR |  |  |
| *N*-arachidonoyl alanine | PISSR |  |  | | PISSR |  |  |
| *N*-docosahexaenoyl alanine | BDL |  |  | | BDL |  |  |
| ***N*-acyl dopamine** |  |  |  | |  |  |  |
| *N*-oleoyl dopamine | BDL |  |  | | BDL |  |  |
| *N*-arachidonoyl dopamine | BDL |  |  | | BDL |  |  |
| ***N*-acyl ethanolamine** |  |  |  | |  |  |  |
| *N*-palmitoyl ethanolamine | 4.19E-10 | 5.31E-11 | 2.17E-11 | | 5.35E-10 | 7.38E-11 | 3.01E-11 |
| *N*-stearoyl ethanolamine | 4.23E-11 | 1.22E-11 | 5E-12 | | 6.08E-11 | 1.17E-11 | 4.79E-12 |
| *N*-oleoyl ethanolamine | 4.73E-10 | 7.3E-11 | 2.98E-11 | | 7.42E-10 | 7.96E-11 | 3.25E-11 |
| *N*-linoleoyl ethanolamine | 1.92E-10 | 3.61E-11 | 1.47E-11 | | 2.5E-10 | 4.77E-11 | 1.95E-11 |
| *N*-arachidonoyl ethanolamine | 2.67E-11 | 4.63E-12 | 1.89E-12 | | 4.19E-11 | 6.12E-12 | 2.5E-12 |
| *N*-docosahexaenoyl ethanolamine | 8.13E-11 | 7.32E-12 | 2.99E-12 | | 1.11E-10 | 1.14E-11 | 4.65E-12 |
| ***N*-acyl GABA** |  |  |  | |  |  |  |
| *N*-palmitoyl GABA | 4.21E-11 | 1.25E-11 | 5.12E-12 | | 4.15E-11 | 7.82E-12 | 3.19E-12 |
| *N*-stearoyl GABA | 2.23E-10 | 4.66E-11 | 1.9E-11 | | 2.34E-10 | 2.48E-11 | 1.01E-11 |
| *N*-oleoyl GABA | 5.97E-11 | 1.23E-11 | 5.04E-12 | | 6.76E-11 | 5.02E-12 | 2.05E-12 |
| *N*-linoleoyl GABA | 2.95E-11 | 8.72E-12 | 3.56E-12 | | 3.86E-11 | 8.87E-12 | 3.62E-12 |
| *N*-arachidonoyl GABA | 2.23E-11 | 2.75E-12 | 1.12E-12 | | 2.78E-11 | 1.72E-12 | 7.01E-13 |
| *N*-docosahexaenoyl GABA | 3.97E-11 | 9.68E-12 | 3.95E-12 | | 4.37E-11 | 1.05E-11 | 4.3E-12 |
| ***N*-acyl glycine** |  |  |  | |  |  |  |
| *N*-palmitoyl glycine | 1.3E-09 | 1.82E-10 | 7.42E-11 | | 1.28E-09 | 2.27E-10 | 9.28E-11 |
| *N*-stearoyl glycine | 1.41E-09 | 2.27E-10 | 9.26E-11 | | 1.45E-09 | 2.2E-10 | 8.98E-11 |
| *N*-oleoyl glycine | 9.16E-10 | 1.61E-10 | 6.58E-11 | | 9.03E-10 | 1.54E-10 | 6.27E-11 |
| *N*-linoleoyl glycine | 3.64E-10 | 7.27E-11 | 2.97E-11 | | 3.3E-10 | 7.12E-11 | 2.91E-11 |
| *N*-arachidonoyl glycine | 3.65E-10 | 9.26E-11 | 3.78E-11 | | 3.21E-10 | 5.92E-11 | 2.42E-11 |
| *N*-docosahexaenoyl glycine | 3.68E-10 | 8.88E-11 | 3.63E-11 | | 2.97E-10 | 6.98E-11 | 2.85E-11 |
| ***N*-acyl leucine** |  |  |  | |  |  |  |
| *N*-palmitoyl leucine | 1.13E-10 | 1.33E-11 | 5.44E-12 | | 1.24E-10 | 2.15E-11 | 8.77E-12 |
| *N*-stearoyl leucine | 7.72E-11 | 1.32E-11 | 5.4E-12 | | 7.64E-11 | 1.15E-11 | 4.68E-12 |
| *N*-oleoyl leucine | 7.41E-11 | 7.47E-12 | 3.05E-12 | | 8.61E-11 | 9.08E-12 | 3.71E-12 |
| *N*-linoleoyl leucine | 1.29E-11 | 3.61E-12 | 1.47E-12 | | 1.56E-11 | 5.84E-12 | 2.38E-12 |
| *N*-docosahexaenoyl leucine | 1.86E-11 | 4.12E-12 | 1.68E-12 | | 2.78E-11 | 5.99E-12 | 2.44E-12 |
| ***N*-acyl methionine** |  |  |  | |  |  |  |
| *N*-palmitoyl methionine | 5.61E-11 | 9.41E-12 | 3.84E-12 | | 5.19E-11 | 2.07E-11 | 8.46E-12 |
| *N*-stearoyl methionine | PISSR |  |  | | PISSR |  |  |
| *N*-oleoyl methionine | PISSR |  |  | | PISSR |  |  |
| *N*-linoleoyl methionine | BDL |  |  | | BDL |  |  |
| *N*-arachidonoyl methionine | BDL |  |  | | BDL |  |  |
| *N*-docosahexaenoyl methionine | BDL |  |  | | BDL |  |  |
| ***N*-acyl phenylalanine** |  |  |  | |  |  |  |
| *N*-palmitoyl phenylalanine | 2.36E-11 | 3.64E-12 | 1.49E-12 | | 2.31E-11 | 8.39E-12 | 3.43E-12 |
| *N*-stearoyl phenylalanine | 3.98E-11 | 5.85E-12 | 2.39E-12 | | 4.61E-11 | 8.81E-12 | 3.6E-12 |
| *N*-oleoyl phenylalanine | 1.1E-11 | 2.69E-12 | 1.1E-12 | | 1.8E-11 | 4.84E-12 | 1.98E-12 |
| *N*-linoleoyl phenylalanine | PISSR |  |  | | PISSR |  |  |
| *N*-arachidonoyl phenylalanine | 8.83E-12 | 1.3E-12 | 5.32E-13 | | 1.37E-11 | 1.57E-12 | 6.39E-13 |
| *N*-docosahexaenoyl phenylalanine | PISSR |  |  | | PISSR |  |  |

**Supplemental Table 9: Continued**

|  | C6 | | | | | |
| --- | --- | --- | --- | --- | --- | --- |
|  | Vehicle | | | 1 µM CBD | | |
| ***N*-acyl proline** | Mean | Std Dev | Std Error | Mean | Std Dev | Std Error |
| *N*-palmitoyl proline | 4.68E-11 | 5.14E-12 | 2.1E-12 | 5.42E-11 | 5.78E-12 | 2.36E-12 |
| *N*-stearoyl proline | 3.02E-11 | 3.64E-12 | 1.48E-12 | 3.21E-11 | 3.29E-12 | 1.34E-12 |
| *N-*oleoyl proline | PISSR |  |  | PISSR |  |  |
| *N*-linoleoyl proline | BDL |  |  | BDL |  |  |
| *N*-arachidonoyl proline | PISSR |  |  | PISSR |  |  |
| *N*-docosahexaenoyl proline | BDL |  |  | BDL |  |  |
| ***N*-acyl serine** |  |  |  |  |  |  |
| *N*-palmitoyl serine | 3.17E-10 | 6.52E-11 | 2.66E-11 | 2.95E-10 | 5.63E-11 | 2.3E-11 |
| *N*-stearoyl serine | 2.16E-10 | 4.36E-11 | 1.78E-11 | 1.87E-10 | 5.27E-11 | 2.15E-11 |
| *N*-oleoyl serine | 2.65E-10 | 6.64E-11 | 2.71E-11 | 2.41E-10 | 6.21E-11 | 2.53E-11 |
| *N*-linoleoyl serine | 1.86E-10 | 5.52E-11 | 2.25E-11 | 1.73E-10 | 7E-11 | 2.86E-11 |
| *N*-arachidonoyl serine | PISSR |  |  | PISSR |  |  |
| *N*-docosahexaenoyl serine | PISSR |  |  | PISSR |  |  |
| ***N*-acyl taurine** |  |  |  |  |  |  |
| *N*-palmitoyl taurine | 1.02E-09 | 2.67E-10 | 1.09E-10 | 9.4E-10 | 1.28E-10 | 5.23E-11 |
| *N*-stearoyl taurine | 1.85E-09 | 4.74E-10 | 1.93E-10 | 2.07E-09 | 4.21E-10 | 1.72E-10 |
| *N*-oleoyl taurine | 1.08E-09 | 9.61E-11 | 3.92E-11 | 1.31E-09 | 1.32E-10 | 5.38E-11 |
| *N*-arachidonoyl taurine | 2.34E-10 | 5.88E-11 | 2.4E-11 | 3.62E-10 | 6.32E-11 | 2.58E-11 |
| ***N*-acyl tryptophan** |  |  |  |  |  |  |
| *N*-palmitoyl tryptophan | PISSR |  |  | PISSR |  |  |
| *N*-stearoyl tryptophan | 7.42E-12 | 8.27E-13 | 3.38E-13 | 5.67E-12 | 6.25E-13 | 2.55E-13 |
| *N*-oleoyl tryptophan | PISSR |  |  | PISSR |  |  |
| *N*-linoleoyl tryptophan | BDL |  |  | BDL |  |  |
| *N*-arachidonoyl tryptophan | BDL |  |  | BDL |  |  |
| *N*-docosahexaenoyl tryptophan | BDL |  |  | BDL |  |  |
| ***N*-acyl tyrosine** |  |  |  |  |  |  |
| *N*-palmitoyl tyrosine | 2.01E-11 | 4.17E-12 | 1.7E-12 | 2.04E-11 | 5.89E-12 | 2.41E-12 |
| *N*-stearoyl tyrosine | 5.79E-12 | 7.48E-13 | 3.05E-13 | 5.36E-12 | 1.61E-12 | 6.59E-13 |
| *N-*oleoyl tyrosine | 2.43E-11 | 3.74E-12 | 1.53E-12 | 2.19E-11 | 5.5E-12 | 2.25E-12 |
| *N*-linoleoyl tyrosine | PISSR |  |  | PISSR |  |  |
| *N*-arachidonoyl tyrosine | 1.21E-11 | 2.24E-12 | 9.15E-13 | 1.72E-11 | 2.23E-12 | 9.12E-13 |
| *N*-docosahexaenoyl tyrosine | PISSR |  |  | PISSR |  |  |
| ***N*-acyl valine** |  |  |  |  |  |  |
| *N*-palmitoyl valine | 1.29E-11 | 5.79E-12 | 2.36E-12 | 1.97E-11 | 7.75E-12 | 3.16E-12 |
| *N*- stearoyl valine | 2.45E-11 | 3.58E-12 | 1.46E-12 | 2.77E-11 | 6.52E-12 | 2.66E-12 |
| *N*-oleoyl valine | 9.71E-12 | 2.09E-12 | 8.52E-13 | 1.51E-11 | 3.64E-12 | 1.49E-12 |
| *N*-nervonoyl valine | BDL |  |  | BDL |  |  |
| *N*-linoleoyl valine | BDL |  |  | BDL |  |  |
| *N*-docosahexaenoyl valine | BDL |  |  | BDL |  |  |
| **2-acyl glycerols** |  |  |  |  |  |  |
| 2-palmitoyl glycerol | 3.13E-08 | 9.71E-09 | 3.96E-09 | 3.33E-08 | 5.07E-09 | 2.07E-09 |
| 2-oleoyl glycerol | 1.07E-08 | 2.62E-09 | 1.07E-09 | 1.16E-08 | 2.35E-09 | 9.6E-10 |
| 2-linoleoyl glycerol | 4.52E-09 | 5.76E-10 | 2.35E-10 | 4.95E-09 | 7.94E-10 | 3.24E-10 |
| 2-arachidonoyl glycerol | 6.05E-09 | 7.03E-10 | 2.87E-10 | 5.99E-09 | 9.25E-10 | 3.78E-10 |
| **Free Fatty Acids** |  |  |  |  |  |  |
| Oleic acid | 3.08E-08 | 6.35E-09 | 2.59E-09 | 3.04E-08 | 4.08E-09 | 1.67E-09 |
| Linoleic acid | 2.55E-08 | 6.59E-09 | 2.69E-09 | 2.35E-08 | 3.4E-09 | 1.39E-09 |
| Arachidonic acid | 1.34E-08 | 2.94E-09 | 1.2E-09 | 1.39E-08 | 2.35E-09 | 9.59E-10 |
| **PhosphoLEA** |  |  |  |  |  |  |
| PhosphoLEA | 1.13E-09 | 3.29E-10 | 1.34E-10 | 1.02E-09 | 3.69E-10 | 1.51E-10 |
| **Prostaglandins** |  |  |  |  |  |  |
| PGE_2_ | BDL |  |  | BDL |  |  |
| PGF_2α_ | BDL |  |  | BDL |  |  |
| 6-ketoPGF_1α_ | BDL |  |  | BDL |  |  |
| **THC/CBD** |  |  |  |  |  |  |
| Cannabidiol | BDL |  |  | 6.8E-08 | 9.46E-09 | 3.86E-09 |
| THC | BDL |  |  | BDL |  |  |
| **THC Metabolites** |  |  |  |  |  |  |
| 11-nor-9-carboxyTHC | BDL |  |  | BDL |  |  |
| 11-OH-THC | BDL |  |  | BDL |  |  |

**Supplemental Table 10: List of lipids in C6 glioma cells significantly affected by 1 µM CBD**

| C6 CBD Significant Differences in One-Way ANOVA | | | | |
| --- | --- | --- | --- | --- |
| Lipid | F | p | Direction (relative to Veh) | Magnitude (x Veh level) |
| *N*-palmitoyl ethanolamine | 9.81 | .001 | ↑ | 1.28 |
| *N*-stearoyl ethanolamine | 7.1 | .024 | ↑ | 1.44 |
| *N*-oleoyl ethanolamine | 37.31 | .000 | ↑ | 1.57 |
| *N*-linoleoyl ethanolamine | 5.63 | .039 | ↑ | 1.30 |
| *N*-arachidonoyl ethanolamine | 23.54 | .001 | ↑ | 1.57 |
| *N*-docosahexaenoyl ethanolamine | 28.03 | .000 | ↑ | 1.37 |
| *N*-arachidonoyl GABA | 17.49 | .002 | ↑ | 1.25 |
| *N*-oleoyl leucine | 6.29 | .031 | ↑ | 1.16 |
| *N*-docosahexaenoyl leucine | 9.67 | .011 | ↑ | 1.49 |
| *N*-oleoyl phenylalanine | 9.67 | .011 | ↑ | 1.64 |
| *N*-arachidonoyl phenylalanine | 33.89 | .000 | ↑ | 1.56 |
| *N*-palmitoyl proline | 5.37 | .043 | ↑ | 1.16 |
| *N*-oleoyl taurine | 12.76 | .005 | ↑ | 1.21 |
| *N*-arachidonoyl taurine | 13.28 | .005 | ↑ | 1.55 |
| *N*-stearoyl tryptophan | 17.18 | .002 | ↓ | 0.76 |
| *N*-arachidonoyl tyrosine | 15.71 | .003 | ↑ | 1.42 |
| *N*-oleoyl valine | 9.83 | .011 | ↑ | 1.56 |
| CBD | 310.54 | .000 | ↑ | infinite |

**Supplemental Table 11: Lipid levels in N18 neuroblastoma cells treated with Vehicle or 1 µM CBD for 2 hours**

|  | N18 | | | | | | |
| --- | --- | --- | --- | --- | --- | --- | --- |
|  | Vehicle | | | 1 µM CBD | | | |
| ***N*-acyl alanine** | Mean | Std Dev | Std Error | | Mean | Std Dev | Std Error |
| *N*-palmitoyl alanine | 1.51E-10 | 4.63E-11 | 1.89E-11 | | 1.59E-10 | 2.59E-11 | 1.06E-11 |
| *N*-stearoyl alanine | 9.3E-11 | 3.06E-11 | 1.25E-11 | | 1.05E-10 | 1.89E-11 | 7.71E-12 |
| *N*-oleoyl alanine | 1.07E-11 | 4.74E-12 | 1.94E-12 | | 1.23E-11 | 1.91E-12 | 7.78E-13 |
| *N*-linoleoyl alanine | PISSR |  |  | | PISSR |  |  |
| *N*-arachidonoyl alanine | PISSR |  |  | | PISSR |  |  |
| *N*-docosahexaenoyl alanine | BDL |  |  | | BDL |  |  |
| ***N*-acyl dopamine** |  |  |  | |  |  |  |
| *N*-oleoyl dopamine | BDL |  |  | | BDL |  |  |
| *N*-arachidonoyl dopamine | BDL |  |  | | BDL |  |  |
| ***N*-acyl ethanolamine** |  |  |  | |  |  |  |
| *N*-palmitoyl ethanolamine | 8.16E-10 | 9.14E-11 | 3.73E-11 | | 1E-09 | 1.3E-10 | 5.32E-11 |
| *N*-stearoyl ethanolamine | 3.39E-10 | 4.62E-11 | 1.89E-11 | | 4.33E-10 | 5.67E-11 | 2.31E-11 |
| *N*-oleoyl ethanolamine | 6.2E-10 | 6.98E-11 | 2.85E-11 | | 7.88E-10 | 7.14E-11 | 2.92E-11 |
| *N*-linoleoyl ethanolamine | 4.51E-11 | 8.64E-12 | 3.53E-12 | | 6.29E-11 | 1.45E-11 | 5.91E-12 |
| *N*-arachidonoyl ethanolamine | 1.01E-10 | 5.39E-12 | 2.2E-12 | | 1.16E-10 | 9.11E-12 | 3.72E-12 |
| *N*-eicosapentaenoyl ethanolamine | 1.88E-11 | 2.98E-12 | 1.21E-12 | | 2.59E-11 | 3.71E-12 | 1.51E-12 |
| *N*-docosahexaenoyl ethanolamine | 3.58E-11 | 6.76E-12 | 2.76E-12 | | 5.98E-11 | 1.21E-11 | 4.93E-12 |
| ***N*-acyl GABA** |  |  |  | |  |  |  |
| *N*-palmitoyl GABA | 3.15E-11 | 1.2E-11 | 4.88E-12 | | 4.06E-11 | 9.52E-12 | 3.89E-12 |
| *N*-stearoyl GABA | 2.9E-11 | 5.38E-12 | 2.2E-12 | | 4.29E-11 | 1.02E-11 | 4.17E-12 |
| *N*-oleoyl GABA | 1.64E-11 | 9.66E-12 | 3.94E-12 | | 1.99E-11 | 4.07E-12 | 1.66E-12 |
| *N*-linoleoyl GABA | 4.24E-12 | 2.17E-12 | 8.84E-13 | | 5.78E-12 | 2.08E-12 | 8.51E-13 |
| *N*-arachidonoyl GABA | PISSR |  |  | | PISSR |  |  |
| *N*-docosahexaenoyl GABA | 6.07E-12 | 1.72E-12 | 7.03E-13 | | 1.11E-11 | 2.79E-12 | 1.14E-12 |
| ***N*-acyl glycine** |  |  |  | |  |  |  |
| *N*-palmitoyl glycine | 9.93E-10 | 1.87E-10 | 7.63E-11 | | 1.09E-09 | 2.89E-10 | 1.18E-10 |
| *N*-stearoyl glycine | 8.01E-10 | 7.08E-11 | 2.89E-11 | | 1E-09 | 1.08E-10 | 4.41E-11 |
| *N*-oleoyl glycine | 3.16E-10 | 5.12E-11 | 2.09E-11 | | 4.03E-10 | 4.04E-11 | 1.65E-11 |
| *N*-linoleoyl glycine | 1.25E-10 | 3.03E-11 | 1.24E-11 | | 1.19E-10 | 2.45E-11 | 1E-11 |
| *N*-arachidonoyl glycine | 1.63E-11 | 3.92E-12 | 1.6E-12 | | 2.58E-11 | 6.35E-12 | 2.59E-12 |
| *N*-docosahexaenoyl glycine | 1.06E-11 | 3.77E-12 | 1.54E-12 | | 1.17E-11 | 2.74E-12 | 1.12E-12 |
| ***N*-acyl leucine** |  |  |  | |  |  |  |
| *N*-palmitoyl leucine | 9.24E-11 | 4.78E-11 | 1.95E-11 | | 1.08E-10 | 4.65E-11 | 1.9E-11 |
| *N*-stearoyl leucine | 1.53E-10 | 3.65E-11 | 1.49E-11 | | 1.62E-10 | 4.45E-11 | 1.82E-11 |
| *N*-oleoyl leucine | 6.94E-11 | 3.54E-11 | 1.45E-11 | | 6.99E-11 | 2.41E-11 | 9.83E-12 |
| *N*-linoleoyl leucine | 4.04E-12 | 2.55E-12 | 1.04E-12 | | 4.93E-12 | 1.45E-12 | 5.93E-13 |
| *N*-docosahexaenoyl leucine | 1.24E-11 | 4.18E-12 | 1.71E-12 | | 1.68E-11 | 3.92E-12 | 1.6E-12 |
| ***N*-acyl methionine** |  |  |  | |  |  |  |
| *N*-palmitoyl methionine | 4.51E-11 | 1.48E-11 | 6.04E-12 | | 3.85E-11 | 8.97E-12 | 3.66E-12 |
| *N*-stearoyl methionine | 2.35E-11 | 1.1E-11 | 4.51E-12 | | 2.87E-11 | 9.29E-12 | 3.79E-12 |
| *N*-oleoyl methionine | 9.73E-12 | 6.49E-12 | 2.65E-12 | | 8.71E-12 | 5.33E-12 | 2.18E-12 |
| *N*-linoleoyl methionine | PISSR |  |  | | PISSR |  |  |
| *N*-arachidonoyl methionine | BDL |  |  | | BDL |  |  |
| *N*-docosahexaenoyl methionine | BDL |  |  | | BDL |  |  |
| ***N*-acyl phenylalanine** |  |  |  | |  |  |  |
| *N*-palmitoyl phenylalanine | 5.16E-11 | 2.25E-11 | 9.17E-12 | | 6.13E-11 | 2.3E-11 | 9.4E-12 |
| *N*-stearoyl phenylalanine | 8.84E-11 | 2.58E-11 | 1.05E-11 | | 8.85E-11 | 1.61E-11 | 6.57E-12 |
| *N*-oleoyl phenylalanine | 4.03E-11 | 1.89E-11 | 7.72E-12 | | 4.05E-11 | 1.46E-11 | 5.94E-12 |
| *N*-linoleoyl phenylalanine | 2.74E-12 | 2.53E-13 | 1.03E-13 | | 2.72E-12 | 3.87E-13 | 1.58E-13 |
| *N*-arachidonoyl phenylalanine | 6.18E-12 | 1.07E-12 | 4.37E-13 | | 5.6E-12 | 2.14E-12 | 8.75E-13 |
| *N*-docosahexaenoyl phenylalanine | PISSR |  |  | | PISSR |  |  |

**Supplemental Table 11: Continued**

|  | N18 | | | | | |
| --- | --- | --- | --- | --- | --- | --- |
|  | Vehicle | | | 1 µM CBD | | |
| ***N*-acyl proline** | Mean | Std Dev | Std Error | Mean | Std Dev | Std Error |
| *N*-palmitoyl proline | 2.09E-11 | 1.17E-11 | 4.76E-12 | 1.74E-11 | 6.03E-12 | 2.46E-12 |
| *N*-stearoyl proline | 8.34E-12 | 2.87E-12 | 1.17E-12 | 8.3E-12 | 2.15E-12 | 8.77E-13 |
| *N-*oleoyl proline | 2.31E-12 | 8.41E-13 | 3.43E-13 | 3.39E-12 | 8.29E-13 | 3.38E-13 |
| *N*-linoleoyl proline | PISSR |  |  | PISSR |  |  |
| *N*-arachidonoyl proline | PISSR |  |  | PISSR |  |  |
| *N*-docosahexaenoyl proline | PISSR |  |  | PISSR |  |  |
| ***N*-acyl serine** |  |  |  |  |  |  |
| *N*-palmitoyl serine | 6.05E-10 | 9.28E-11 | 3.79E-11 | 5.77E-10 | 4.37E-11 | 1.78E-11 |
| *N*-stearoyl serine | 2.29E-10 | 5.39E-11 | 2.2E-11 | 2.2E-10 | 3.17E-11 | 1.29E-11 |
| *N*-oleoyl serine | 1.82E-10 | 6.24E-11 | 2.55E-11 | 1.77E-10 | 6.28E-12 | 2.57E-12 |
| *N*-linoleoyl serine | 1.15E-10 | 3.54E-11 | 1.44E-11 | 1.05E-10 | 3.74E-11 | 1.53E-11 |
| *N*-arachidonoyl serine | 1.63E-11 | 5.57E-12 | 2.27E-12 | 1.89E-11 | 5.89E-12 | 2.4E-12 |
| *N*-docosahexaenoyl serine | 1.1E-10 | 2.04E-11 | 8.31E-12 | 1.15E-10 | 3.89E-11 | 1.59E-11 |
| ***N*-acyl taurine** |  |  |  |  |  |  |
| *N*-palmitoyl taurine | 2.3E-09 | 4.43E-10 | 1.81E-10 | 2.29E-09 | 2.49E-10 | 1.02E-10 |
| *N*-stearoyl taurine | 5.95E-09 | 1.13E-09 | 4.59E-10 | 6.73E-09 | 5.01E-10 | 2.05E-10 |
| *N*-oleoyl taurine | 7.57E-10 | 1.6E-10 | 6.53E-11 | 8.36E-10 | 8.16E-11 | 3.33E-11 |
| *N*-arachidonoyl taurine | 3.05E-10 | 6.09E-11 | 2.49E-11 | 4.28E-10 | 5.31E-11 | 2.17E-11 |
| ***N*-acyl tryptophan** |  |  |  |  |  |  |
| *N*-palmitoyl tryptophan | 6.14E-11 | 1.67E-11 | 6.8E-12 | 6.11E-11 | 1.95E-11 | 7.95E-12 |
| *N*-stearoyl tryptophan | 1.2E-10 | 2.36E-11 | 9.62E-12 | 1.27E-10 | 2.89E-11 | 1.18E-11 |
| *N*-oleoyl tryptophan | PISSR |  |  | PISSR |  |  |
| *N*-linoleoyl tryptophan | BDL |  |  | BDL |  |  |
| *N*-arachidonoyl tryptophan | BDL |  |  | BDL |  |  |
| *N*-docosahexaenoyl tryptophan | PISSR |  |  | PISSR |  |  |
| ***N*-acyl tyrosine** |  |  |  |  |  |  |
| *N*-palmitoyl tyrosine | 2.79E-11 | 4.46E-12 | 1.82E-12 | 3.07E-11 | 4.18E-12 | 1.71E-12 |
| *N*-stearoyl tyrosine | 5.03E-12 | 1.26E-12 | 5.15E-13 | 5.14E-12 | 9.39E-13 | 3.83E-13 |
| *N-*oleoyl tyrosine | 2.13E-11 | 5.86E-12 | 2.39E-12 | 2.09E-11 | 2.96E-12 | 1.21E-12 |
| *N*-linoleoyl tyrosine | 4.15E-12 | 1.45E-12 | 5.92E-13 | 4.12E-12 | 1.58E-12 | 6.46E-13 |
| *N*-arachidonoyl tyrosine | 3.87E-12 | 1.05E-12 | 4.3E-13 | 5.09E-12 | 9.73E-13 | 3.97E-13 |
| *N*-docosahexaenoyl tyrosine | 5.92E-12 | 2.06E-12 | 8.42E-13 | 5.48E-12 | 1.3E-12 | 5.32E-13 |
| ***N*-acyl valine** |  |  |  |  |  |  |
| *N*-palmitoyl valine | 4.49E-11 | 2.92E-11 | 1.19E-11 | 4.54E-11 | 1.96E-11 | 8.01E-12 |
| *N*- stearoyl valine | 3.56E-11 | 7.97E-12 | 3.25E-12 | 4.82E-11 | 1.16E-11 | 4.73E-12 |
| *N*-oleoyl valine | 1.77E-11 | 1.05E-11 | 4.3E-12 | 1.5E-11 | 4.29E-12 | 1.75E-12 |
| *N*-nervonoyl valine | BDL |  |  | BDL |  |  |
| *N*-linoleoyl valine | PISSR |  |  | PISSR |  |  |
| *N*-docosahexaenoyl valine | PISSR |  |  | PISSR |  |  |
| **2-acyl glycerols** |  |  |  |  |  |  |
| 2-palmitoyl glycerol | 2.24E-08 | 4.25E-09 | 1.74E-09 | 3.29E-08 | 9.68E-09 | 3.95E-09 |
| 2-oleoyl glycerol | 1.25E-08 | 1.48E-09 | 6.06E-10 | 1.59E-08 | 2.09E-09 | 8.54E-10 |
| 2-linoleoyl glycerol | 1.96E-09 | 2.27E-10 | 9.25E-11 | 2.57E-09 | 3.03E-10 | 1.24E-10 |
| 2-arachidonoyl glycerol | 4.75E-09 | 4.71E-10 | 1.92E-10 | 4.54E-09 | 5.29E-10 | 2.16E-10 |
| **Free Fatty Acids** |  |  |  |  |  |  |
| Oleic acid | 3.48E-09 | 9.22E-10 | 3.77E-10 | 4.37E-09 | 7.62E-10 | 3.11E-10 |
| Linoleic acid | 1.09E-08 | 3.11E-09 | 1.27E-09 | 1.07E-08 | 7.75E-10 | 3.16E-10 |
| Arachidonic acid | 1E-08 | 2.81E-09 | 1.15E-09 | 1.01E-08 | 1.21E-09 | 4.95E-10 |
| **PhosphoLEA** |  |  |  |  |  |  |
| PhosphoLEA | 4.38E-09 | 6.16E-10 | 2.52E-10 | 5.7E-09 | 8.56E-10 | 3.49E-10 |
| **Prostaglandins** |  |  |  |  |  |  |
| PGE_2_ | 2.56E-10 | 5.22E-11 | 2.13E-11 | 1.72E-10 | 4.27E-11 | 1.75E-11 |
| PGF_2α_ | BDL |  |  | BDL |  |  |
| 6-ketoPGF_1α_ | BDL |  |  | BDL |  |  |
| **THC/CBD** |  |  |  |  |  |  |
| Cannabidiol | BDL |  |  | 1.92E-07 | 2.38E-08 | 9.71E-09 |
| THC | BDL |  |  | BDL |  |  |
| **THC Metabolites** |  |  |  |  |  |  |
| 11-nor-9-carboxyTHC | BDL |  |  | BDL |  |  |
| 11-OH-THC | BDL |  |  | BDL |  |  |

**Supplemental Table 12: List of lipids in N18 neuroblastoma cells significantly affected by 1 µM CBD**

| N18 CBD Significant Differences in One-Way ANOVA | | | | |
| --- | --- | --- | --- | --- |
| Lipid | F | p | Direction (relative to Veh) | Magnitude (x Veh level) |
| *N*-palmitoyl ethanolamine | 8.06 | .018 | ↑ | 1.23 |
| *N*-stearoyl ethanolamine | 9.87 | .010 | ↑ | 1.28 |
| *N*-oleoyl ethanolamine | 16.88 | .002 | ↑ | 1.27 |
| *N*-linoleoyl ethanolamine | 6.66 | .027 | ↑ | 1.39 |
| *N*-arachidonoyl ethanolamine | 11.95 | .006 | ↑ | 1.16 |
| *N*-docosahexaenoyl ethanolamine | 18.11 | .002 | ↑ | 1.67 |
| *N*-stearoyl GABA | 8.70 | .015 | ↑ | 1.48 |
| *N*-docosahexaenoyl GABA | 14.1 | .004 | ↑ | 1.81 |
| *N*-stearoyl glycine | 14.60 | .003 | ↑ | 1.25 |
| *N*-oleoyl glycine | 10.67 | .008 | ↑ | 1.28 |
| *N*-arachidonoyl glycine | 9.79 | .011 | ↑ | 1.58 |
| *N*-docosahexaenoyl leucine | 3.52 | .090 | ↑ | 1.35 |
| *N*-oleoyl proline | 5.03 | .049 | ↑ | 1.47 |
| *N*-arachidonoyl taurine | 13.75 | .004 | ↑ | 1.40 |
| *N*-arachidonoyl tyrosine | 4.33 | .064 | ↑ | 1.32 |
| *N*-stearoyl valine | 4.86 | .052 | ↑ | 1.35 |
| 2-palmitoyl glycerol | 5.93 | .035 | ↑ | 1.47 |
| 2-oleoyl glycerol | 10.46 | .009 | ↑ | 1.27 |
| 2-linoleoyl glycerol | 15.85 | .003 | ↑ | 1.31 |
| Oleic acid | 3.36 | .097 | ↑ | 1.26 |
| phosphoLEA | 9.34 | .012 | ↑ | 1.30 |
| PGE_2_ | 9.40 | .012 | ↓ | 0.67 |
| CBD | 390.14 | .000 | ↑ | infinite |

**Supplemental Table 13: Lipid levels in BV2 microglial cells treated with Vehicle or 1 µM THC:CBD for 2 hours**

|  | BV2 | | | | | | |
| --- | --- | --- | --- | --- | --- | --- | --- |
|  | Vehicle | | | 1 µM THC:CBD | | | |
| ***N*-acyl alanine** | Mean | Std Dev | Std Error | | Mean | Std Dev | Std Error |
| *N*-palmitoyl alanine | 3.08E-10 | 4.71E-11 | 1.92E-11 | | 2.82E-10 | 4.24E-11 | 1.73E-11 |
| *N*-stearoyl alanine | 1.79E-10 | 2.96E-11 | 1.21E-11 | | 1.94E-10 | 2.54E-11 | 1.04E-11 |
| *N*-oleoyl alanine | 2.29E-11 | 3.3E-12 | 1.35E-12 | | 3.01E-11 | 6.83E-12 | 2.79E-12 |
| *N*-linoleoyl alanine | PISSR |  |  | | PISSR |  |  |
| *N*-arachidonoyl alanine | 2.87E-11 | 5.13E-12 | 2.1E-12 | | 2.88E-11 | 4.67E-12 | 1.91E-12 |
| *N*-docosahexaenoyl alanine | BDL |  |  | | BDL |  |  |
| ***N*-acyl dopamine** |  |  |  | |  |  |  |
| *N*-oleoyl dopamine | BDL |  |  | | BDL |  |  |
| *N*-arachidonoyl dopamine | BDL |  |  | | BDL |  |  |
| ***N*-acyl ethanolamine** |  |  |  | |  |  |  |
| *N*-palmitoyl ethanolamine | 1.33E-09 | 2.07E-10 | 8.46E-11 | | 1.89E-09 | 2.36E-10 | 9.63E-11 |
| *N*-stearoyl ethanolamine | 6.41E-10 | 1.21E-10 | 4.95E-11 | | 9.52E-10 | 1.65E-10 | 6.73E-11 |
| *N*-oleoyl ethanolamine | 1.63E-09 | 2.49E-10 | 1.02E-10 | | 2.69E-09 | 4.75E-10 | 1.94E-10 |
| *N*-linoleoyl ethanolamine | 8.9E-11 | 1.89E-11 | 7.72E-12 | | 1.55E-10 | 3.54E-11 | 1.45E-11 |
| *N*-arachidonoyl ethanolamine | 5.54E-11 | 8.47E-12 | 3.46E-12 | | 8.77E-11 | 6.04E-12 | 2.47E-12 |
| *N*-docosahexaenoyl ethanolamine | 1.75E-10 | 2.05E-11 | 8.38E-12 | | 2.06E-10 | 1.28E-11 | 5.23E-12 |
| ***N*-acyl GABA** |  |  |  | |  |  |  |
| *N*-palmitoyl GABA | 8.25E-11 | 1.82E-11 | 7.43E-12 | | 8.04E-11 | 1.44E-11 | 5.87E-12 |
| *N*-stearoyl GABA | 7.8E-11 | 1.49E-11 | 6.09E-12 | | 1.03E-10 | 1.55E-11 | 6.34E-12 |
| *N*-oleoyl GABA | 7.68E-11 | 2.29E-11 | 9.36E-12 | | 7.74E-11 | 2.16E-11 | 8.84E-12 |
| *N*-linoleoyl GABA | 3.73E-11 | 1.1E-11 | 4.48E-12 | | 4.81E-11 | 1.99E-11 | 8.13E-12 |
| *N*-arachidonoyl GABA | PISSR |  |  | | PISSR |  |  |
| *N*-docosahexaenoyl GABA | 2.52E-11 | 8.95E-12 | 3.65E-12 | | 2.42E-11 | 4.66E-12 | 1.9E-12 |
| ***N*-acyl glycine** |  |  |  | |  |  |  |
| *N*-palmitoyl glycine | 4.39E-10 | 5E-11 | 2.04E-11 | | 5.55E-10 | 9.45E-11 | 3.86E-11 |
| *N*-stearoyl glycine | 6.26E-10 | 6.51E-11 | 2.66E-11 | | 7.62E-10 | 7.85E-11 | 3.21E-11 |
| *N*-oleoyl glycine | 3.9E-10 | 4.67E-11 | 1.91E-11 | | 5.5E-10 | 6.86E-11 | 2.8E-11 |
| *N*-linoleoyl glycine | 1.83E-10 | 4.17E-11 | 1.7E-11 | | 2.69E-10 | 4.7E-11 | 1.92E-11 |
| *N*-arachidonoyl glycine | 1.56E-11 | 5.6E-12 | 2.29E-12 | | 1.82E-11 | 3.23E-12 | 1.32E-12 |
| *N*-docosahexaenoyl glycine | 1.96E-11 | 2.31E-12 | 9.41E-13 | | 2.45E-11 | 3.8E-12 | 1.55E-12 |
| ***N*-acyl leucine** |  |  |  | |  |  |  |
| *N*-palmitoyl leucine | 1.29E-10 | 2.13E-11 | 8.68E-12 | | 1.17E-10 | 2.1E-11 | 8.58E-12 |
| *N*-stearoyl leucine | 1.65E-10 | 1.57E-11 | 6.41E-12 | | 1.36E-10 | 1.62E-11 | 6.62E-12 |
| *N*-oleoyl leucine | 1.44E-10 | 2.27E-11 | 9.26E-12 | | 1.3E-10 | 1.51E-11 | 6.16E-12 |
| *N*-linoleoyl leucine | 1.6E-11 | 4.25E-12 | 1.73E-12 | | 1.1E-11 | 2.58E-12 | 1.05E-12 |
| *N*-docosahexaenoyl leucine | 4.1E-11 | 9.1E-12 | 3.71E-12 | | 3.38E-11 | 6.74E-12 | 2.75E-12 |
| ***N*-acyl methionine** |  |  |  | |  |  |  |
| *N*-palmitoyl methionine | 1.32E-10 | 3.02E-11 | 1.23E-11 | | 9.87E-11 | 1.09E-11 | 4.43E-12 |
| *N*-stearoyl methionine | 5.67E-11 | 6.44E-12 | 2.63E-12 | | 4.21E-11 | 9.03E-12 | 3.68E-12 |
| *N*-oleoyl methionine | 7.74E-11 | 2.88E-11 | 1.18E-11 | | 7.23E-11 | 2.44E-11 | 9.95E-12 |
| *N*-linoleoyl methionine | PISSR |  |  | | PISSR |  |  |
| *N*-arachidonoyl methionine | PISSR |  |  | | PISSR |  |  |
| *N*-docosahexaenoyl methionine | PISSR |  |  | | PISSR |  |  |
| ***N*-acyl phenylalanine** |  |  |  | |  |  |  |
| *N*-palmitoyl phenylalanine | 6.78E-11 | 1.11E-11 | 4.51E-12 | | 6.93E-11 | 1.29E-11 | 5.28E-12 |
| *N*-stearoyl phenylalanine | 8.39E-11 | 1.17E-11 | 4.79E-12 | | 7.26E-11 | 1.15E-11 | 4.68E-12 |
| *N*-oleoyl phenylalanine | 6.89E-11 | 9.23E-12 | 3.77E-12 | | 5.91E-11 | 6.97E-12 | 2.85E-12 |
| *N*-linoleoyl phenylalanine | 7.62E-12 | 1.81E-12 | 7.41E-13 | | 6.9E-12 | 2.43E-12 | 9.91E-13 |
| *N*-arachidonoyl phenylalanine | 1.47E-11 | 2.07E-12 | 8.45E-13 | | 1.96E-11 | 3.37E-12 | 1.38E-12 |
| *N*-docosahexaenoyl phenylalanine | PISSR |  |  | | PISSR |  |  |

**Supplemental Table 13: Continued**

|  | BV2 | | | | | |
| --- | --- | --- | --- | --- | --- | --- |
|  | Vehicle | | | 1 µM THC:CBD | | |
| ***N*-acyl proline** | Mean | Std Dev | Std Error | Mean | Std Dev | Std Error |
| *N*-palmitoyl proline | 4.32E-11 | 7.7E-12 | 3.15E-12 | 2.84E-11 | 8.01E-12 | 3.27E-12 |
| *N*-stearoyl proline | 1.53E-11 | 2.88E-12 | 1.17E-12 | 1.47E-11 | 1.29E-12 | 5.28E-13 |
| *N-*oleoyl proline | 8.06E-12 | 1.88E-12 | 7.69E-13 | 6.67E-12 | 2.78E-12 | 1.14E-12 |
| *N*-linoleoyl proline | PISSR |  |  | PISSR |  |  |
| *N*-arachidonoyl proline | PISSR |  |  | PISSR |  |  |
| *N*-docosahexaenoyl proline | PISSR |  |  | PISSR |  |  |
| ***N*-acyl serine** |  |  |  |  |  |  |
| *N*-palmitoyl serine | 1.49E-09 | 1.51E-10 | 6.16E-11 | 1.53E-09 | 2.46E-10 | 1E-10 |
| *N*-stearoyl serine | 3.71E-10 | 3.44E-11 | 1.4E-11 | 4.36E-10 | 5.31E-11 | 2.17E-11 |
| *N*-oleoyl serine | 4.28E-10 | 6.54E-11 | 2.67E-11 | 6.04E-10 | 7.13E-11 | 2.91E-11 |
| *N*-linoleoyl serine | 3.5E-10 | 1.29E-10 | 5.26E-11 | 3.86E-10 | 9.86E-11 | 4.02E-11 |
| *N*-arachidonoyl serine | PISSR |  |  | PISSR |  |  |
| *N*-docosahexaenoyl serine | 3.23E-10 | 1.41E-10 | 5.77E-11 | 2.92E-10 | 9.77E-11 | 3.99E-11 |
| ***N*-acyl taurine** |  |  |  |  |  |  |
| *N*-palmitoyl taurine | 2.41E-09 | 3.93E-10 | 1.6E-10 | 3.37E-09 | 6.87E-10 | 2.8E-10 |
| *N*-stearoyl taurine | 3.88E-09 | 5.01E-10 | 2.05E-10 | 5.24E-09 | 4.31E-10 | 1.76E-10 |
| *N*-oleoyl taurine | 1.72E-09 | 3.36E-10 | 1.37E-10 | 2.22E-09 | 2.76E-10 | 1.13E-10 |
| *N*-arachidonoyl taurine | 3.38E-10 | 8.94E-11 | 3.65E-11 | 6.25E-10 | 7.43E-11 | 3.03E-11 |
| ***N*-acyl tryptophan** |  |  |  |  |  |  |
| *N*-palmitoyl tryptophan | PISSR |  |  | PISSR |  |  |
| *N*-stearoyl tryptophan | 4.07E-10 | 1.06E-10 | 4.31E-11 | 4.09E-10 | 1.13E-10 | 4.63E-11 |
| *N*-oleoyl tryptophan | PISSR |  |  | PISSR |  |  |
| *N*-linoleoyl tryptophan | PISSR |  |  | PISSR |  |  |
| *N*-arachidonoyl tryptophan | BDL |  |  | BDL |  |  |
| *N*-docosahexaenoyl tryptophan | PISSR |  |  | PISSR |  |  |
| ***N*-acyl tyrosine** |  |  |  |  |  |  |
| *N*-palmitoyl tyrosine | 1.08E-10 | 1.04E-11 | 4.26E-12 | 1.02E-10 | 1.65E-11 | 6.73E-12 |
| *N*-stearoyl tyrosine | 1.73E-11 | 1.91E-12 | 7.82E-13 | 1.71E-11 | 3.77E-12 | 1.54E-12 |
| *N-*oleoyl tyrosine | 1.53E-10 | 2.27E-11 | 9.26E-12 | 1.41E-10 | 1.72E-11 | 7.01E-12 |
| *N*-linoleoyl tyrosine | 2.15E-11 | 3.96E-12 | 1.62E-12 | 2.32E-11 | 5.36E-12 | 2.19E-12 |
| *N*-arachidonoyl tyrosine | 1.31E-11 | 1.48E-12 | 6.04E-13 | 1.58E-11 | 3.43E-12 | 1.4E-12 |
| *N*-docosahexaenoyl tyrosine | 1.79E-11 | 3.55E-12 | 1.45E-12 | 1.58E-11 | 3.46E-12 | 1.41E-12 |
| ***N*-acyl valine** |  |  |  |  |  |  |
| *N*-palmitoyl valine | 5.92E-11 | 7.93E-12 | 3.24E-12 | 5.37E-11 | 1.68E-11 | 6.84E-12 |
| *N*- stearoyl valine | 4.41E-11 | 9.45E-12 | 3.86E-12 | 4.31E-11 | 9.12E-12 | 3.72E-12 |
| *N*-oleoyl valine | 2.49E-11 | 7.62E-12 | 3.11E-12 | 2.17E-11 | 7.65E-12 | 3.12E-12 |
| *N*-nervonoyl valine | BDL |  |  | BDL |  |  |
| *N*-linoleoyl valine | PISSR |  |  | PISSR |  |  |
| *N*-docosahexaenoyl valine | PISSR |  |  | PISSR |  |  |
| **2-acyl glycerols** |  |  |  |  |  |  |
| 2-palmitoyl glycerol | 3.2E-08 | 8.53E-09 | 3.48E-09 | 3.46E-08 | 8.74E-09 | 3.57E-09 |
| 2-oleoyl glycerol | 2.68E-08 | 4.47E-09 | 1.82E-09 | 3.03E-08 | 4.68E-09 | 1.91E-09 |
| 2-linoleoyl glycerol | 4.58E-09 | 4.83E-10 | 1.97E-10 | 3.5E-09 | 4.78E-10 | 1.95E-10 |
| 2-arachidonoyl glycerol | 1.1E-08 | 2.38E-09 | 9.7E-10 | 1.12E-08 | 2.11E-09 | 8.63E-10 |
| **Free Fatty Acids** |  |  |  |  |  |  |
| Oleic acid | 5.53E-09 | 9.85E-10 | 4.02E-10 | 5.12E-09 | 1.07E-09 | 4.38E-10 |
| Linoleic acid | 1.38E-08 | 2.43E-09 | 9.91E-10 | 1.5E-08 | 2E-09 | 8.15E-10 |
| Arachidonic acid | 1.37E-08 | 9.42E-10 | 3.85E-10 | 1.19E-08 | 1.04E-09 | 4.25E-10 |
| **PhosphoLEA** |  |  |  |  |  |  |
| PhosphoLEA | 1.28E-08 | 8.19E-10 | 3.34E-10 | 7.95E-09 | 1.48E-09 | 6.06E-10 |
| **Prostaglandins** |  |  |  |  |  |  |
| PGE_2_ | 1.97E-08 | 4.94E-09 | 2.02E-09 | 2.04E-08 | 3.77E-09 | 1.54E-09 |
| PGF_2α_ | 5.27E-09 | 7.29E-10 | 2.98E-10 | 6.72E-09 | 6.47E-10 | 2.64E-10 |
| 6-ketoPGF_1α_ | 3.98E-10 | 8.5E-11 | 3.47E-11 | 4.63E-10 | 5.21E-11 | 2.13E-11 |
| **THC/CBD** |  |  |  |  |  |  |
| Cannabidiol | BDL |  |  | 4.74E-07 | 5.92E-08 | 2.42E-08 |
| THC | BDL |  |  | 5.14E-07 | 6.51E-08 | 2.66E-08 |
| **THC Metabolites** |  |  |  |  |  |  |
| 11-nor-9-carboxyTHC | BDL |  |  | 2.51E-10 | 9.32E-11 | 3.8E-11 |
| 11-OH-THC | BDL |  |  | 2.97E-09 | 3.86E-10 | 1.58E-10 |

**Supplemental Table 14: List of lipids in BV2 microglial cells significantly affected by 1 µM THC:CBD**

| BV2 THC:CBD Significant Differences in One-Way ANOVA | | | | |
| --- | --- | --- | --- | --- |
| Lipid | F | p | Direction (relative to Veh) | Magnitude (x Veh level) |
| *N*-oleoyl alanine | 5.39 | .043 | ↑ | 1.31 |
| *N*-palmitoyl ethanolamine | 19.52 | .001 | ↑ | 1.42 |
| *N*-stearoyl ethanolamine | 13.87 | .004 | ↑ | 1.49 |
| *N*-oleoyl ethanolamine | 23.25 | .001 | ↑ | 1.65 |
| *N*-linoleoyl ethanolamine | 16.29 | .002 | ↑ | 1.74 |
| *N*-arachidonoyl ethanolamine | 57.55 | .000 | ↑ | 1.58 |
| *N*-docosahexaenoyl ethanolamine | 9.88 | .010 | ↑ | 1.18 |
| *N*-stearoyl GABA | 8.32 | .016 | ↑ | 1.29 |
| *N*-palmitoyl glycine | 7.05 | .024 | ↑ | 1.26 |
| *N*-stearoyl glycine | 10.70 | .008 | ↑ | 1.22 |
| *N*-oleoyl glycine | 22.27 | .001 | ↑ | 1.41 |
| *N*-linoleoyl glycine | 11.43 | .007 | ↑ | 1.47 |
| *N*-docosahexaenoyl glycine | 7.32 | .022 | ↑ | 1.25 |
| *N*-stearoyl leucine | 9.47 | .012 | ↓ | 0.82 |
| *N*-linoleoyl leucine | 6.13 | .033 | ↓ | 0.69 |
| *N*-palmitoyl methionine | 6.47 | .029 | ↓ | 0.75 |
| *N*-stearoyl methionine | 10.28 | .009 | ↓ | 0.74 |
| *N*-oleoyl phenylalanine | 4.28 | .065 | ↓ | 0.86 |
| *N*-arachidonoyl phenylalanine | 9.20 | .013 | ↑ | 1.33 |
| *N*-palmitoyl proline | 10.64 | .009 | ↓ | 0.66 |
| *N*-stearoyl serine | 6.32 | .031 | ↑ | 1.18 |
| *N*-oleoyl serine | 19.71 | .001 | ↑ | 1.41 |
| *N*-palmitoyl taurine | 8.86 | .014 | ↑ | 1.40 |
| *N*-stearoyl taurine | 25.12 | .001 | ↑ | 1.35 |
| *N*-oleoyl taurine | 7.78 | .019 | ↑ | 1.28 |
| *N*-arachidonoyl taurine | 36.56 | .000 | ↑ | 1.85 |
| 2-linoleoyl glycerol | 15.31 | .003 | ↓ | 0.76 |
| Arachidonic acid | 9.50 | .012 | ↓ | 0.87 |
| phosphoLEA | 48.89 | .000 | ↓ | 0.62 |
| PGF_2α_ | 13.21 | .005 | ↑ | 1.28 |
| CBD | 385.21 | .000 | ↑ | infinite |
| THC | 373.54 | .000 | ↑ | infinite |
| (±)-11-nor-9-carboxy-THC | 43.7 | .000 | ↑ | infinite |
| 11-OH-THC | 355.63 | .000 | ↑ | infinite |

**Supplemental Table 15: Lipid levels in C6 glioma cells treated with Vehicle or 1 µM THC:CBD for 2 hours**

|  | C6 | | | | | | |
| --- | --- | --- | --- | --- | --- | --- | --- |
|  | Vehicle | | | 1 µM THC:CBD | | | |
| ***N*-acyl alanine** | Mean | Std Dev | Std Error | | Mean | Std Dev | Std Error |
| *N*-palmitoyl alanine | 2.38E-10 | 5.68E-11 | 2.32E-11 | | 2.28E-10 | 3.07E-11 | 1.25E-11 |
| *N*-stearoyl alanine | 2.71E-10 | 9.23E-11 | 3.77E-11 | | 2.34E-10 | 3.33E-11 | 1.36E-11 |
| *N*-oleoyl alanine | 1.15E-10 | 2.78E-11 | 1.14E-11 | | 1.08E-10 | 1.42E-11 | 5.78E-12 |
| *N*-linoleoyl alanine | PISSR |  |  | | PISSR |  |  |
| *N*-arachidonoyl alanine | BDL |  |  | | BDL |  |  |
| *N*-docosahexaenoyl alanine | BDL |  |  | | BDL |  |  |
| ***N*-acyl dopamine** |  |  |  | |  |  |  |
| *N*-oleoyl dopamine | BDL |  |  | | BDL |  |  |
| *N*-arachidonoyl dopamine | BDL |  |  | | BDL |  |  |
| ***N*-acyl ethanolamine** |  |  |  | |  |  |  |
| *N*-palmitoyl ethanolamine | 3.27E-10 | 1.02E-10 | 4.16E-11 | | 3.3E-10 | 5.32E-11 | 2.17E-11 |
| *N*-stearoyl ethanolamine | 2.64E-11 | 1.06E-11 | 4.34E-12 | | 2.32E-11 | 1.1E-11 | 4.47E-12 |
| *N*-oleoyl ethanolamine | 3.56E-10 | 7.18E-11 | 2.93E-11 | | 4.28E-10 | 7.15E-11 | 2.92E-11 |
| *N*-linoleoyl ethanolamine | 1.65E-10 | 5.48E-11 | 2.24E-11 | | 2.18E-10 | 5.42E-11 | 2.21E-11 |
| *N*-arachidonoyl ethanolamine | 1.86E-11 | 4.37E-12 | 1.78E-12 | | 2.51E-11 | 3.72E-12 | 1.52E-12 |
| *N*-docosahexaenoyl ethanolamine | 7.48E-11 | 2.09E-11 | 8.53E-12 | | 8.37E-11 | 1.13E-11 | 4.62E-12 |
| ***N*-acyl GABA** |  |  |  | |  |  |  |
| *N*-palmitoyl GABA | 2.94E-11 | 8.42E-12 | 3.44E-12 | | 1.7E-11 | 6.13E-12 | 2.5E-12 |
| *N*-stearoyl GABA | 1.23E-10 | 3.01E-11 | 1.23E-11 | | 1.08E-10 | 9.12E-12 | 3.73E-12 |
| *N*-oleoyl GABA | 2.55E-11 | 8.62E-12 | 3.52E-12 | | 2.13E-11 | 4.28E-12 | 1.75E-12 |
| *N*-linoleoyl GABA | 1.04E-11 | 1.35E-12 | 5.52E-13 | | 8.3E-12 | 3.37E-12 | 1.38E-12 |
| *N*-arachidonoyl GABA | 9.94E-12 | 4.27E-12 | 1.74E-12 | | 7.66E-12 | 1.89E-12 | 7.72E-13 |
| *N*-docosahexaenoyl GABA | 1.24E-11 | 3.61E-12 | 1.47E-12 | | 1.07E-11 | 2.67E-12 | 1.09E-12 |
| ***N*-acyl glycine** |  |  |  | |  |  |  |
| *N*-palmitoyl glycine | 1.6E-09 | 8.75E-10 | 3.57E-10 | | 9.91E-10 | 1.37E-10 | 5.61E-11 |
| *N*-stearoyl glycine | 1.34E-09 | 6.06E-10 | 2.47E-10 | | 1.05E-09 | 1.37E-10 | 5.58E-11 |
| *N*-oleoyl glycine | 6.53E-10 | 1.2E-10 | 4.92E-11 | | 6.49E-10 | 7.55E-11 | 3.08E-11 |
| *N*-linoleoyl glycine | 2.6E-10 | 5.09E-11 | 2.08E-11 | | 2.35E-10 | 4.53E-11 | 1.85E-11 |
| *N*-arachidonoyl glycine | 2.77E-10 | 4.94E-11 | 2.01E-11 | | 2.5E-10 | 3.28E-11 | 1.34E-11 |
| *N*-docosahexaenoyl glycine | 2.27E-10 | 4.13E-11 | 1.69E-11 | | 2.16E-10 | 2.68E-11 | 1.09E-11 |
| ***N*-acyl leucine** |  |  |  | |  |  |  |
| *N*-palmitoyl leucine | 7.22E-11 | 1.48E-11 | 6.05E-12 | | 6.11E-11 | 1.27E-11 | 5.18E-12 |
| *N*-stearoyl leucine | 4.96E-11 | 1.13E-11 | 4.6E-12 | | 4.69E-11 | 7.2E-12 | 2.94E-12 |
| *N*-oleoyl leucine | 4.98E-11 | 1.21E-11 | 4.94E-12 | | 4.11E-11 | 8.62E-12 | 3.52E-12 |
| *N*-linoleoyl leucine | 4.32E-12 | 8.19E-13 | 3.34E-13 | | 3.41E-12 | 8.7E-13 | 3.55E-13 |
| *N*-docosahexaenoyl leucine | 6.14E-12 | 1.71E-12 | 6.97E-13 | | 6.1E-12 | 1.84E-12 | 7.51E-13 |
| ***N*-acyl methionine** |  |  |  | |  |  |  |
| *N*-palmitoyl methionine | 4.78E-11 | 1.88E-11 | 7.69E-12 | | 4.21E-11 | 2.22E-11 | 9.06E-12 |
| *N*-stearoyl methionine | PISSR |  |  | | PISSR |  |  |
| *N*-oleoyl methionine | PISSR |  |  | | PISSR |  |  |
| *N*-linoleoyl methionine | BDL |  |  | | BDL |  |  |
| *N*-arachidonoyl methionine | BDL |  |  | | BDL |  |  |
| *N*-docosahexaenoyl methionine | BDL |  |  | | BDL |  |  |
| ***N*-acyl phenylalanine** |  |  |  | |  |  |  |
| *N*-palmitoyl phenylalanine | 2.09E-11 | 8.58E-12 | 3.5E-12 | | 1.19E-11 | 2.16E-12 | 8.82E-13 |
| *N*-stearoyl phenylalanine | 2.35E-11 | 1.04E-11 | 4.25E-12 | | 2.03E-11 | 3.8E-12 | 1.55E-12 |
| *N*-oleoyl phenylalanine | 5.93E-12 | 1.11E-12 | 4.54E-13 | | 5.33E-12 | 6.58E-13 | 2.69E-13 |
| *N*-linoleoyl phenylalanine | PISSR |  |  | | PISSR |  |  |
| *N*-arachidonoyl phenylalanine | 4.78E-12 | 6.35E-13 | 2.59E-13 | | 6.55E-12 | 9.69E-13 | 3.96E-13 |
| *N*-docosahexaenoyl phenylalanine | BDL |  |  | | BDL |  |  |

**Supplemental Table 15: Continued**

|  | C6 | | | | | |
| --- | --- | --- | --- | --- | --- | --- |
|  | Vehicle | | | 1 µM THC:CBD | | |
| ***N*-acyl proline** | Mean | Std Dev | Std Error | Mean | Std Dev | Std Error |
| *N*-palmitoyl proline | 3.78E-11 | 1.05E-11 | 4.28E-12 | 3.11E-11 | 7.51E-12 | 3.07E-12 |
| *N*-stearoyl proline | 1.91E-11 | 5.67E-12 | 2.31E-12 | 1.77E-11 | 2.08E-12 | 8.51E-13 |
| *N-*oleoyl proline | 1.44E-11 | 4.09E-12 | 1.67E-12 | 1.5E-11 | 3.88E-12 | 1.58E-12 |
| *N*-linoleoyl proline | BDL |  |  | BDL |  |  |
| *N*-arachidonoyl proline | PISSR |  |  | PISSR |  |  |
| *N*-docosahexaenoyl proline | BDL |  |  | BDL |  |  |
| ***N*-acyl serine** |  |  |  |  |  |  |
| *N*-palmitoyl serine | 2.2E-10 | 5.24E-11 | 2.14E-11 | 2.22E-10 | 1.75E-11 | 7.13E-12 |
| *N*-stearoyl serine | 2.17E-10 | 8.89E-11 | 3.63E-11 | 1.95E-10 | 5.21E-11 | 2.13E-11 |
| *N*-oleoyl serine | 1.64E-10 | 3.85E-11 | 1.57E-11 | 1.49E-10 | 2.06E-11 | 8.4E-12 |
| *N*-linoleoyl serine | 1E-10 | 2.75E-11 | 1.12E-11 | 9.68E-11 | 2.26E-11 | 9.21E-12 |
| *N*-arachidonoyl serine | 3.56E-11 | 7.95E-12 | 3.24E-12 | 3.28E-11 | 1.52E-11 | 6.2E-12 |
| *N*-docosahexaenoyl serine | PISSR |  |  | PISSR |  |  |
| ***N*-acyl taurine** |  |  |  |  |  |  |
| *N*-palmitoyl taurine | 8.79E-10 | 2.21E-10 | 9.01E-11 | 8.75E-10 | 1.41E-10 | 5.76E-11 |
| *N*-stearoyl taurine | 1.33E-09 | 2.99E-10 | 1.22E-10 | 1.49E-09 | 3.92E-10 | 1.6E-10 |
| *N*-oleoyl taurine | 1.06E-09 | 1.86E-10 | 7.61E-11 | 1.07E-09 | 1.26E-10 | 5.13E-11 |
| *N*-arachidonoyl taurine | 2.73E-10 | 7.78E-11 | 3.18E-11 | 2.96E-10 | 4.96E-11 | 2.02E-11 |
| ***N*-acyl tryptophan** |  |  |  |  |  |  |
| *N*-palmitoyl tryptophan | PISSR |  |  | PISSR |  |  |
| *N*-stearoyl tryptophan | PISSR |  |  | PISSR |  |  |
| *N*-oleoyl tryptophan | PISSR |  |  | PISSR |  |  |
| *N*-linoleoyl tryptophan | BDL |  |  | BDL |  |  |
| *N*-arachidonoyl tryptophan | PISSR |  |  | PISSR |  |  |
| *N*-docosahexaenoyl tryptophan | PISSR |  |  | PISSR |  |  |
| ***N*-acyl tyrosine** |  |  |  |  |  |  |
| *N*-palmitoyl tyrosine | 1.51E-11 | 4.31E-12 | 1.76E-12 | 1.02E-11 | 1.77E-12 | 7.21E-13 |
| *N*-stearoyl tyrosine | 4.48E-12 | 1.05E-12 | 4.29E-13 | 2.8E-12 | 7.36E-13 | 3E-13 |
| *N-*oleoyl tyrosine | 1.27E-11 | 4.3E-12 | 1.75E-12 | 1.27E-11 | 2.66E-12 | 1.09E-12 |
| *N*-linoleoyl tyrosine | PISSR |  |  | PISSR |  |  |
| *N*-arachidonoyl tyrosine | 1.44E-11 | 4.74E-12 | 1.93E-12 | 1.05E-11 | 3.3E-12 | 1.35E-12 |
| *N*-docosahexaenoyl tyrosine | PISSR |  |  | PISSR |  |  |
| ***N*-acyl valine** |  |  |  |  |  |  |
| *N*-palmitoyl valine | 6.6E-12 | 1.51E-12 | 6.17E-13 | 4.69E-12 | 1.87E-12 | 7.65E-13 |
| *N*- stearoyl valine | 1.29E-11 | 3.88E-12 | 1.58E-12 | 1.17E-11 | 2.24E-12 | 9.13E-13 |
| *N*-oleoyl valine | 5E-12 | 1.72E-12 | 7.01E-13 | 4.52E-12 | 1.87E-12 | 7.62E-13 |
| *N*-nervonoyl valine | BDL |  |  | BDL |  |  |
| *N*-linoleoyl valine | BDL |  |  | BDL |  |  |
| *N*-docosahexaenoyl valine | BDL |  |  | BDL |  |  |
| **2-acyl glycerols** |  |  |  |  |  |  |
| 2-palmitoyl glycerol | 2.03E-08 | 3.66E-09 | 1.49E-09 | 1.51E-08 | 4.67E-09 | 1.91E-09 |
| 2-oleoyl glycerol | 6.37E-09 | 8.13E-10 | 3.32E-10 | 4.42E-09 | 1.11E-09 | 4.51E-10 |
| 2-linoleoyl glycerol | 2.62E-09 | 6.66E-10 | 2.72E-10 | 1.82E-09 | 3.26E-10 | 1.33E-10 |
| 2-arachidonoyl glycerol | 5.68E-09 | 1.01E-09 | 4.12E-10 | 4.67E-09 | 3.86E-10 | 1.58E-10 |
| **Free Fatty Acids** |  |  |  |  |  |  |
| Oleic acid | 1.5E-08 | 3.3E-09 | 1.35E-09 | 1.14E-08 | 7.85E-10 | 3.21E-10 |
| Linoleic acid | 1.27E-08 | 2.33E-09 | 9.51E-10 | 9.74E-09 | 7.47E-10 | 3.05E-10 |
| Arachidonic acid | 7.78E-09 | 1.01E-09 | 4.14E-10 | 5.52E-09 | 7.52E-10 | 3.07E-10 |
| **PhosphoLEA** |  |  |  |  |  |  |
| PhosphoLEA | 5.31E-10 | 9.21E-11 | 3.76E-11 | 7.88E-10 | 5.53E-11 | 2.26E-11 |
| **Prostaglandins** |  |  |  |  |  |  |
| PGE_2_ | BDL |  |  | BDL |  |  |
| PGF_2α_ | BDL |  |  | BDL |  |  |
| 6-ketoPGF_1α_ | BDL |  |  | BDL |  |  |
| **THC/CBD** |  |  |  |  |  |  |
| Cannabidiol | BDL |  |  | 5.28E-08 | 4.73E-09 | 1.93E-09 |
| THC | BDL |  |  | 9.53E-08 | 1.24E-08 | 5.05E-09 |
| **THC Metabolites** |  |  |  |  |  |  |
| 11-nor-9-carboxyTHC | BDL |  |  | 8.19E-10 | 9.29E-11 | 3.79E-11 |
| 11-OH-THC | BDL |  |  | 1.2E-09 | 1.38E-10 | 5.65E-11 |

**Supplemental Table 16: List of lipids in C6 glioma cells significantly affected by 1 µM THC:CBD**

| C6 THC:CBD Significant Differences in One-Way ANOVA | | | | |
| --- | --- | --- | --- | --- |
| Lipid | F | p | Direction (relative to Veh) | Magnitude (x Veh level) |
| *N*-arachidonoyl ethanolamine | 7.65 | .020 | ↑ | 1.35 |
| *N*-palmitoyl GABA | 8.41 | .016 | ↓ | 0.58 |
| *N*-linoleoyl leucine | 3.48 | .092 | ↓ | 0.79 |
| *N*-palmitoyl phenylalanine | 6.17 | .032 | ↓ | 0.57 |
| *N*-arachidonoyl phenylalanine | 14.03 | .004 | ↑ | 1.37 |
| *N*-palmitoyl tyrosine | 6.77 | .026 | ↓ | 0.68 |
| *N*-stearoyl tyrosine | 10.30 | .009 | ↓ | 0.63 |
| *N*-palmitoyl valine | 3.76 | .081 | ↓ | 0.71 |
| 2-palmitoyl glycerol | 4.59 | .058 | ↓ | 0.74 |
| 2-oleoyl glycerol | 12.20 | .006 | ↓ | 0.69 |
| 2-linoleoyl glycerol | 7.06 | .024 | ↓ | 0.69 |
| 2-arachidonoyl glycerol | 5.24 | .045 | ↓ | 0.82 |
| Oleic acid | 6.68 | .027 | ↓ | 0.76 |
| Linoleic acid | 8.51 | .015 | ↓ | 0.76 |
| Arachidonic acid | 19.27 | .001 | ↓ | 0.71 |
| phosphoLEA | 34.37 | .000 | ↑ | 1.48 |
| CBD | 745.78 | .000 | ↑ | infinite |
| THC | 355.75 | .000 | ↑ | infinite |
| (±)-11-nor-9-carboxy-THC | 465.85 | .000 | ↑ | infinite |
| 11-OH-THC | 450.65 | .000 | ↑ | infinite |

**Supplemental Table 17: Lipid levels in N18 neuroblastoma cells treated with Vehicle or 1 µM THC:CBD for 2 hours**

|  | N18 | | | | | | |
| --- | --- | --- | --- | --- | --- | --- | --- |
|  | Vehicle | | | 1 µM THC:CBD | | | |
| ***N*-acyl alanine** | Mean | Std Dev | Std Error | | Mean | Std Dev | Std Error |
| *N*-palmitoyl alanine | 1.22E-10 | 1.4E-11 | 5.73E-12 | | 1.28E-10 | 1.41E-11 | 5.76E-12 |
| *N*-stearoyl alanine | 6.88E-11 | 4.91E-12 | 2.01E-12 | | 8.51E-11 | 9.68E-12 | 3.95E-12 |
| *N*-oleoyl alanine | 6.71E-12 | 8.73E-13 | 3.56E-13 | | 7.11E-12 | 1.1E-12 | 4.51E-13 |
| *N*-linoleoyl alanine | 1.38E-12 | 4.46E-13 | 1.82E-13 | | 1.59E-12 | 3.69E-13 | 1.51E-13 |
| *N*-arachidonoyl alanine | PISSR |  |  | | PISSR |  |  |
| *N*-docosahexaenoyl alanine | BDL |  |  | | BDL |  |  |
| ***N*-acyl dopamine** |  |  |  | |  |  |  |
| *N*-oleoyl dopamine | BDL |  |  | | BDL |  |  |
| *N*-arachidonoyl dopamine | BDL |  |  | | BDL |  |  |
| ***N*-acyl ethanolamine** |  |  |  | |  |  |  |
| *N*-palmitoyl ethanolamine | 8.91E-10 | 6.09E-11 | 2.49E-11 | | 1.01E-09 | 5.21E-11 | 2.13E-11 |
| *N*-stearoyl ethanolamine | 3.59E-10 | 6.37E-11 | 2.6E-11 | | 3.69E-10 | 6.82E-11 | 2.78E-11 |
| *N*-oleoyl ethanolamine | 5.41E-10 | 5.59E-11 | 2.28E-11 | | 6.18E-10 | 6.94E-11 | 2.83E-11 |
| *N*-linoleoyl ethanolamine | 3.27E-11 | 6.26E-12 | 2.56E-12 | | 3.73E-11 | 3.74E-12 | 1.53E-12 |
| *N*-arachidonoyl ethanolamine | 1.03E-10 | 1.36E-11 | 5.55E-12 | | 1.03E-10 | 1.1E-11 | 4.5E-12 |
| *N*-eicosapentaenoyl ethanolamine | 2.45E-11 | 6.2E-12 | 2.53E-12 | | 3.32E-11 | 7.09E-12 | 2.89E-12 |
| *N*-docosahexaenoyl ethanolamine | 4.27E-11 | 8.76E-12 | 3.58E-12 | | 6.23E-11 | 9.34E-12 | 3.81E-12 |
| ***N*-acyl GABA** |  |  |  | |  |  |  |
| *N*-palmitoyl GABA | 1.57E-11 | 2.62E-12 | 1.07E-12 | | 1.95E-11 | 2.51E-12 | 1.02E-12 |
| *N*-stearoyl GABA | 2E-11 | 2.6E-12 | 1.06E-12 | | 2.61E-11 | 1.56E-12 | 6.37E-13 |
| *N*-oleoyl GABA | 6.58E-12 | 1.53E-12 | 6.24E-13 | | 9.74E-12 | 2.01E-12 | 8.19E-13 |
| *N*-linoleoyl GABA | PISSR |  |  | | PISSR |  |  |
| *N*-arachidonoyl GABA | PISSR |  |  | | PISSR |  |  |
| *N*-docosahexaenoyl GABA | 5.5E-12 | 1.06E-12 | 4.34E-13 | | 5.35E-12 | 1.82E-12 | 7.43E-13 |
| ***N*-acyl glycine** |  |  |  | |  |  |  |
| *N*-palmitoyl glycine | 8.6E-10 | 1.01E-10 | 4.13E-11 | | 9.59E-10 | 6.92E-11 | 2.82E-11 |
| *N*-stearoyl glycine | 8.1E-10 | 4.3E-11 | 1.76E-11 | | 9.3E-10 | 6.21E-11 | 2.53E-11 |
| *N*-oleoyl glycine | 4.04E-10 | 3.01E-11 | 1.23E-11 | | 4.56E-10 | 3.28E-11 | 1.34E-11 |
| *N*-linoleoyl glycine | 6.43E-10 | 8.11E-11 | 3.31E-11 | | 6.8E-10 | 8.42E-11 | 3.44E-11 |
| *N*-arachidonoyl glycine | 2.7E-11 | 5.52E-12 | 2.25E-12 | | 3.31E-11 | 7.22E-12 | 2.95E-12 |
| *N*-docosahexaenoyl glycine | 1.58E-11 | 3.25E-12 | 1.33E-12 | | 2.2E-11 | 5.26E-12 | 2.15E-12 |
| ***N*-acyl leucine** |  |  |  | |  |  |  |
| *N*-palmitoyl leucine | 5.31E-11 | 1.22E-11 | 5E-12 | | 5.95E-11 | 1.22E-11 | 4.98E-12 |
| *N*-stearoyl leucine | 1.22E-10 | 1.83E-11 | 7.48E-12 | | 1.46E-10 | 1.2E-11 | 4.92E-12 |
| *N*-oleoyl leucine | 4.12E-11 | 5.22E-12 | 2.13E-12 | | 5.21E-11 | 8.13E-12 | 3.32E-12 |
| *N*-linoleoyl leucine | 1.83E-12 | 6.26E-13 | 2.56E-13 | | 2.77E-12 | 7.86E-13 | 3.21E-13 |
| *N*-docosahexaenoyl leucine | 4.91E-12 | 1.1E-12 | 4.49E-13 | | 7.29E-12 | 1.75E-12 | 7.16E-13 |
| ***N*-acyl methionine** |  |  |  | |  |  |  |
| *N*-palmitoyl methionine | 4.23E-11 | 9.21E-12 | 3.76E-12 | | 3.09E-11 | 4.48E-12 | 1.83E-12 |
| *N*-stearoyl methionine | 9.99E-12 | 3.11E-12 | 1.27E-12 | | 1.66E-11 | 4.58E-12 | 1.87E-12 |
| *N*-oleoyl methionine | PISSR |  |  | | PISSR |  |  |
| *N*-linoleoyl methionine | BDL |  |  | | BDL |  |  |
| *N*-arachidonoyl methionine | BDL |  |  | | BDL |  |  |
| *N*-docosahexaenoyl methionine | BDL |  |  | | BDL |  |  |
| ***N*-acyl phenylalanine** |  |  |  | |  |  |  |
| *N*-palmitoyl phenylalanine | 3.65E-11 | 8.28E-12 | 3.38E-12 | | 4.17E-11 | 7.23E-12 | 2.95E-12 |
| *N*-stearoyl phenylalanine | 7.35E-11 | 1.03E-11 | 4.19E-12 | | 9E-11 | 1.22E-11 | 4.98E-12 |
| *N*-oleoyl phenylalanine | 2.48E-11 | 4.22E-12 | 1.72E-12 | | 3.12E-11 | 6.38E-12 | 2.61E-12 |
| *N*-linoleoyl phenylalanine | 1.46E-12 | 4.09E-13 | 1.67E-13 | | 1.26E-12 | 4.17E-13 | 1.7E-13 |
| *N*-arachidonoyl phenylalanine | 2.59E-12 | 5.7E-13 | 2.33E-13 | | 4.32E-12 | 7.2E-13 | 2.94E-13 |
| *N*-docosahexaenoyl phenylalanine | PISSR |  |  | | PISSR |  |  |

**Supplemental Table 17: Continued**

|  | N18 | | | | | |
| --- | --- | --- | --- | --- | --- | --- |
|  | Vehicle | | | 1 µM THC:CBD | | |
| ***N*-acyl proline** | Mean | Std Dev | Std Error | Mean | Std Dev | Std Error |
| *N*-palmitoyl proline | 1.02E-11 | 2.12E-12 | 8.66E-13 | 9.6E-12 | 2.41E-12 | 9.86E-13 |
| *N*-stearoyl proline | 5.97E-12 | 5.32E-13 | 2.17E-13 | 6.53E-12 | 9.31E-13 | 3.8E-13 |
| *N-*oleoyl proline | 1.84E-12 | 6.06E-13 | 2.47E-13 | 2.22E-12 | 9.14E-13 | 3.73E-13 |
| *N*-linoleoyl proline | PISSR |  |  | PISSR |  |  |
| *N*-arachidonoyl proline | PISSR |  |  | PISSR |  |  |
| *N*-docosahexaenoyl proline | PISSR |  |  | PISSR |  |  |
| ***N*-acyl serine** |  |  |  |  |  |  |
| *N*-palmitoyl serine | 4.52E-10 | 2.63E-11 | 1.07E-11 | 5.06E-10 | 2.66E-11 | 1.09E-11 |
| *N*-stearoyl serine | 1.83E-10 | 2.32E-11 | 9.46E-12 | 2.13E-10 | 2.95E-11 | 1.2E-11 |
| *N*-oleoyl serine | 1.89E-10 | 1.3E-11 | 5.29E-12 | 2.2E-10 | 1.52E-11 | 6.19E-12 |
| *N*-linoleoyl serine | 9.29E-11 | 1.23E-11 | 5.03E-12 | 1.26E-10 | 1.69E-11 | 6.89E-12 |
| *N*-arachidonoyl serine | 2.77E-11 | 8.04E-12 | 3.28E-12 | 3.85E-11 | 8.11E-12 | 3.31E-12 |
| *N*-docosahexaenoyl serine | 1.13E-10 | 3.05E-11 | 1.25E-11 | 1.29E-10 | 2.49E-11 | 1.02E-11 |
| ***N*-acyl taurine** |  |  |  |  |  |  |
| *N*-palmitoyl taurine | 2.43E-09 | 1.91E-10 | 7.8E-11 | 2.73E-09 | 3.63E-10 | 1.48E-10 |
| *N*-stearoyl taurine | 6.3E-09 | 4.05E-10 | 1.65E-10 | 7.55E-09 | 8.03E-10 | 3.28E-10 |
| *N*-oleoyl taurine | 1.15E-09 | 1.02E-10 | 4.17E-11 | 1.29E-09 | 2.23E-10 | 9.11E-11 |
| *N*-arachidonoyl taurine | 4.99E-10 | 7.26E-11 | 2.97E-11 | 6.68E-10 | 1.6E-10 | 6.54E-11 |
| ***N*-acyl tryptophan** |  |  |  |  |  |  |
| *N*-palmitoyl tryptophan | PISSR |  |  | PISSR |  |  |
| *N*-stearoyl tryptophan | 7.79E-11 | 1.54E-11 | 6.29E-12 | 9.04E-11 | 9.94E-12 | 4.06E-12 |
| *N*-oleoyl tryptophan | PISSR |  |  | PISSR |  |  |
| *N*-linoleoyl tryptophan | BDL |  |  | BDL |  |  |
| *N*-arachidonoyl tryptophan | BDL |  |  | BDL |  |  |
| *N*-docosahexaenoyl tryptophan | BDL |  |  | BDL |  |  |
| ***N*-acyl tyrosine** |  |  |  |  |  |  |
| *N*-palmitoyl tyrosine | 2.61E-11 | 2.55E-12 | 1.04E-12 | 2.61E-11 | 2.56E-12 | 1.05E-12 |
| *N*-stearoyl tyrosine | 4.87E-12 | 3.78E-13 | 1.54E-13 | 5.57E-12 | 3.78E-13 | 1.54E-13 |
| *N-*oleoyl tyrosine | 2.23E-11 | 2.3E-12 | 9.37E-13 | 2.47E-11 | 2.78E-12 | 1.13E-12 |
| *N*-linoleoyl tyrosine | 1.99E-12 | 9.28E-13 | 3.79E-13 | 2.59E-12 | 8.08E-13 | 3.3E-13 |
| *N*-arachidonoyl tyrosine | 1.76E-12 | 3.61E-13 | 1.47E-13 | 2.76E-12 | 5.35E-13 | 2.18E-13 |
| *N*-docosahexaenoyl tyrosine | 3.48E-12 | 7.94E-13 | 3.24E-13 | 4.84E-12 | 8.24E-13 | 3.37E-13 |
| ***N*-acyl valine** |  |  |  |  |  |  |
| *N*-palmitoyl valine | 2.08E-11 | 3.59E-12 | 1.47E-12 | 2.44E-11 | 6.15E-12 | 2.51E-12 |
| *N*- stearoyl valine | 4.02E-11 | 6.86E-12 | 2.8E-12 | 4.93E-11 | 6.47E-12 | 2.64E-12 |
| *N*-oleoyl valine | 9.9E-12 | 2.94E-12 | 1.2E-12 | 1.18E-11 | 3.84E-12 | 1.57E-12 |
| *N*-nervonoyl valine | BDL |  |  | BDL |  |  |
| *N*-linoleoyl valine | PISSR |  |  | PISSR |  |  |
| *N*-docosahexaenoyl valine | PISSR |  |  | PISSR |  |  |
| **2-acyl glycerols** |  |  |  |  |  |  |
| 2-palmitoyl glycerol | 9.04E-09 | 1.79E-09 | 7.3E-10 | 1.01E-08 | 1.11E-09 | 4.51E-10 |
| 2-oleoyl glycerol | 1.18E-08 | 1.28E-09 | 5.24E-10 | 1.35E-08 | 1.29E-09 | 5.27E-10 |
| 2-linoleoyl glycerol | 1.97E-09 | 2.32E-10 | 9.48E-11 | 2.29E-09 | 3.39E-10 | 1.39E-10 |
| 2-arachidonoyl glycerol | 5.88E-09 | 2.54E-09 | 1.04E-09 | 5.23E-09 | 2.5E-09 | 1.02E-09 |
| **Free Fatty Acids** |  |  |  |  |  |  |
| Oleic acid | 3.58E-09 | 9.28E-10 | 3.79E-10 | 4.11E-09 | 9.67E-10 | 3.95E-10 |
| Linoleic acid | 1.12E-08 | 1.96E-09 | 8E-10 | 1.36E-08 | 4.74E-09 | 1.94E-09 |
| Arachidonic acid | 8.36E-09 | 1.36E-09 | 5.53E-10 | 9.13E-09 | 1.52E-09 | 6.22E-10 |
| **PhosphoLEA** |  |  |  |  |  |  |
| PhosphoLEA | 3.08E-10 | 7.05E-11 | 2.88E-11 | 3.3E-10 | 7.18E-11 | 2.93E-11 |
| **Prostaglandins** |  |  |  |  |  |  |
| PGE_2_ | 2.11E-10 | 1.45E-11 | 5.9E-12 | 3.35E-10 | 4.3E-11 | 1.76E-11 |
| PGF_2α_ | 9.01E-10 | 1.92E-10 | 7.83E-11 | 1.4E-09 | 3.19E-10 | 1.3E-10 |
| 6-ketoPGF_1α_ | PISSR |  |  | PISSR |  |  |
| **THC/CBD** |  |  |  |  |  |  |
| Cannabidiol | BDL |  |  | 1.86E-07 | 6.08E-09 | 2.48E-09 |
| THC | BDL |  |  | 1.48E-07 | 7.91E-09 | 3.23E-09 |
| **THC Metabolites** |  |  |  |  |  |  |
| 11-nor-9-carboxyTHC | BDL |  |  | 2.52E-10 | 1.91E-11 | 7.79E-12 |
| 11-OH-THC | BDL |  |  | 1.07E-09 | 1.17E-10 | 4.77E-11 |

Supplemental Table 18: List of lipids in N18 neuroblastoma cells significantly affected by 1 µM THC:CBD

| N18 THC:CBD Significant Differences in One-Way ANOVA | | | | |
| --- | --- | --- | --- | --- |
| Lipid | F | p | Direction (relative to Veh) | Magnitude (x Veh level) |
| *N*-stearoyl alanine | 13.57 | .004 | ↑ | 1.24 |
| *N*-palmitoyl ethanolamine | 12.98 | .005 | ↑ | 1.13 |
| *N*-oleoyl ethanolamine | 4.45 | .061 | ↑ | 1.14 |
| *N*-docosahexaenoyl ethanolamine | 14.07 | .004 | ↑ | 1.46 |
| *N*-palmitoyl GABA | 6.70 | .027 | ↑ | 1.24 |
| *N*-stearoyl GABA | 24.31 | .001 | ↑ | 1.31 |
| *N*-oleoyl GABA | 9.45 | .012 | ↑ | 1.48 |
| *N*-palmitoyl glycine | 3.91 | .076 | ↑ | 1.12 |
| *N*-stearoyl glycine | 15.15 | .003 | ↑ | 1.15 |
| *N*-oleoyl glycine | 8.39 | .016 | ↑ | 1.13 |
| *N*-docosahexaenoyl glycine | 6.12 | .033 | ↑ | 1.39 |
| *N*-stearoyl leucine | 7.46 | .021 | ↑ | 1.20 |
| *N*-oleoyl leucine | 7.58 | .020 | ↑ | 1.26 |
| *N*-linoleoyl leucine | 5.24 | .025 | ↑ | 1.51 |
| *N*-docosahexaenoyl leucine | 7.88 | .019 | ↑ | 1.48 |
| *N*-palmitoyl methionine | 7.37 | .022 | ↓ | 0.73 |
| *N*-stearoyl methionine | 8.52 | .015 | ↑ | 1.66 |
| *N*-stearoyl phenylalanine | 6.39 | .030 | ↑ | 1.22 |
| *N*-oleoyl phenylalanine | 4.25 | .066 | ↑ | 1.26 |
| *N*-arachidonoyl phenylalanine | 21.32 | .001 | ↑ | 1.67 |
| *N*-palmitoyl serine | 12.19 | .006 | ↑ | 1.12 |
| *N*-stearoyl serine | 3.74 | .082 | ↑ | 1.16 |
| *N*-oleoyl serine | 14.72 | .003 | ↑ | 1.16 |
| *N*-linoleoyl serine | 15.12 | .003 | ↑ | 1.35 |
| *N*-arachidonoyl serine | 5.33 | .044 | ↑ | 1.39 |
| *N*-palmitoyl taurine | 3.36 | .097 | ↑ | 1.12 |
| *N*-stearoyl taurine | 11.46 | .007 | ↑ | 1.18 |
| *N*-arachidonoyl taurine | 5.52 | .041 | ↑ | 1.34 |
| *N*-stearoyl tyrosine | 10.49 | .009 | ↑ | 1.14 |
| *N*-arachidonoyl tyrosine | 14.55 | .003 | ↑ | 1.57 |
| *N*-docosahexaenoyl tyrosine | 8.49 | .015 | ↑ | 1.39 |
| *N*-stearoyl valine | 5.53 | .040 | ↑ | 1.23 |
| 2-oleoyl glycerol | 5.75 | .037 | ↑ | 1.14 |
| 2-linoleoyl glycerol | 3.50 | .091 | ↑ | 1.16 |
| PGE_2_ | 44.94 | .000 | ↑ | 1.59 |
| PGF_2α_ | 10.61 | .009 | ↑ | 1.56 |
| CBD | 5596.58 | .000 | ↑ | infinite |
| THC | 2109.06 | .000 | ↑ | infinite |
| (±)-11-nor-9-carboxy-THC | 501.75 | .000 | ↑ | infinite |
| 11-OH-THC | 1051.42 | .000 | ↑ | infinite |

**Supplemental Table 19: Continued Supplemental Table 1: Lipid levels in BV2 microglial cells treated with Vehicle or 1 µM URB597 for 2 hours**

|  | BV2 | | | | | | |
| --- | --- | --- | --- | --- | --- | --- | --- |
|  | Vehicle | | | 1 µM URB597 | | | |
| ***N*-acyl alanine** | Mean | Std Dev | Std Error | | Mean | Std Dev | Std Error |
| *N*-palmitoyl alanine | 6.96E-11 | 5.38E-12 | 2.2E-12 | | 6.79E-11 | 5.82E-12 | 2.38E-12 |
| *N*-stearoyl alanine | 6.63E-11 | 7.38E-12 | 3.01E-12 | | 6.46E-11 | 6.7E-12 | 2.73E-12 |
| *N*-oleoyl alanine | 1.08E-11 | 2.08E-13 | 8.48E-14 | | 1.41E-11 | 1.01E-12 | 4.14E-13 |
| *N*-linoleoyl alanine | 1.48E-12 | 6.81E-13 | 2.78E-13 | | 3.25E-12 | 1.13E-12 | 4.62E-13 |
| *N*-arachidonoyl alanine | 1.61E-11 | 3.41E-12 | 1.39E-12 | | 1.08E-11 | 1.5E-12 | 6.12E-13 |
| *N*-docosahexaenoyl alanine | PISSR |  |  | | PISSR |  |  |
| ***N*-acyl dopamine** |  |  |  | |  |  |  |
| *N*-oleoyl dopamine | BDL |  |  | | BDL |  |  |
| *N*-arachidonoyl dopamine | BDL |  |  | | BDL |  |  |
| ***N*-acyl ethanolamine** |  |  |  | |  |  |  |
| *N*-palmitoyl ethanolamine | 1.5E-10 | 5.44E-11 | 2.22E-11 | | 1.06E-09 | 1.67E-10 | 6.84E-11 |
| *N*-stearoyl ethanolamine | 2.42E-11 | 9.67E-12 | 3.95E-12 | | 1.79E-10 | 5.26E-11 | 2.15E-11 |
| *N*-oleoyl ethanolamine | 3.38E-10 | 7.38E-11 | 3.01E-11 | | 2.95E-08 | 4.87E-09 | 1.99E-09 |
| *N*-linoleoyl ethanolamine | 6.14E-12 | 1.14E-12 | 4.67E-13 | | 1.29E-10 | 2.94E-11 | 1.2E-11 |
| *N*-arachidonoyl ethanolamine | 3.66E-11 | 8.27E-12 | 3.37E-12 | | 1.4E-10 | 2.1E-11 | 8.56E-12 |
| *N*-docosahexaenoyl ethanolamine | 5.96E-12 | 1.6E-12 | 6.54E-13 | | 3.88E-11 | 5.48E-12 | 2.24E-12 |
| ***N*-acyl GABA** |  |  |  | |  |  |  |
| *N*-palmitoyl GABA | 3.85E-11 | 6.82E-12 | 2.78E-12 | | 8.31E-11 | 1.06E-11 | 4.33E-12 |
| *N*-stearoyl GABA | 2.77E-11 | 3.69E-12 | 1.51E-12 | | 4.96E-11 | 7.62E-12 | 3.11E-12 |
| *N*-oleoyl GABA | 2.23E-11 | 5.34E-12 | 2.18E-12 | | 3.77E-11 | 6.55E-12 | 2.68E-12 |
| *N*-linoleoyl GABA | 1.83E-11 | 4.57E-12 | 1.86E-12 | | 3.55E-11 | 1.3E-11 | 5.29E-12 |
| *N*-arachidonoyl GABA | 4.27E-12 | 1.14E-12 | 4.66E-13 | | 2.47E-12 | 8.09E-13 | 3.3E-13 |
| *N*-docosahexaenoyl GABA | 9.14E-12 | 2.17E-12 | 8.85E-13 | | 1.38E-11 | 2.27E-12 | 9.27E-13 |
| ***N*-acyl glycine** |  |  |  | |  |  |  |
| *N*-palmitoyl glycine | 3.01E-10 | 5.15E-11 | 2.1E-11 | | 4.07E-10 | 4.83E-11 | 1.97E-11 |
| *N*-stearoyl glycine | 2.28E-10 | 2.8E-11 | 1.14E-11 | | 4.15E-10 | 4.26E-11 | 1.74E-11 |
| *N*-oleoyl glycine | 1.34E-10 | 9.91E-12 | 4.05E-12 | | 3.33E-10 | 3.82E-11 | 1.56E-11 |
| *N*-linoleoyl glycine | 4.16E-11 | 7.18E-12 | 2.93E-12 | | 9.02E-11 | 1.47E-11 | 5.99E-12 |
| *N*-arachidonoyl glycine | 3.93E-11 | 5.51E-12 | 2.25E-12 | | 2.59E-11 | 3.47E-12 | 1.42E-12 |
| *N*-docosahexaenoyl glycine | 1.14E-11 | 2.18E-12 | 8.92E-13 | | 1.24E-11 | 4.02E-12 | 1.64E-12 |
| ***N*-acyl leucine** |  |  |  | |  |  |  |
| *N*-palmitoyl leucine | 4.05E-11 | 8.61E-12 | 3.52E-12 | | 3.62E-11 | 5.75E-12 | 2.35E-12 |
| *N*-stearoyl leucine | 6.19E-11 | 1.06E-11 | 4.34E-12 | | 5.1E-11 | 3.56E-12 | 1.45E-12 |
| *N*-oleoyl leucine | 4.15E-11 | 1.1E-11 | 4.47E-12 | | 3.59E-11 | 6.76E-12 | 2.76E-12 |
| *N*-linoleoyl leucine | 5.4E-12 | 5.86E-13 | 2.39E-13 | | 5.21E-12 | 1.36E-12 | 5.56E-13 |
| *N*-docosahexaenoyl leucine | 1.9E-11 | 3.64E-12 | 1.49E-12 | | 2.02E-11 | 2.03E-12 | 8.27E-13 |
| ***N*-acyl methionine** |  |  |  | |  |  |  |
| *N*-palmitoyl methionine | 2.7E-10 | 4.34E-11 | 1.77E-11 | | 2.65E-10 | 4.33E-11 | 1.77E-11 |
| *N*-stearoyl methionine | 3.76E-11 | 1.19E-11 | 4.86E-12 | | 3.96E-11 | 6.54E-12 | 2.67E-12 |
| *N*-oleoyl methionine | 3.81E-11 | 1.58E-11 | 6.43E-12 | | 3.73E-11 | 1.42E-11 | 5.81E-12 |
| *N*-linoleoyl methionine | PISSR |  |  | | PISSR |  |  |
| *N*-arachidonoyl methionine | 1.37E-11 | 1.45E-12 | 5.93E-13 | | 6.83E-12 | 1.88E-12 | 7.67E-13 |
| *N*-docosahexaenoyl methionine | PISSR |  |  | | PISSR |  |  |
| ***N*-acyl phenylalanine** |  |  |  | |  |  |  |
| *N*-palmitoyl phenylalanine | 1.14E-10 | 1.87E-11 | 7.62E-12 | | 1.1E-10 | 1.46E-11 | 5.98E-12 |
| *N*-stearoyl phenylalanine | 4.72E-11 | 9.26E-12 | 3.78E-12 | | 4.53E-11 | 5.34E-12 | 2.18E-12 |
| *N*-oleoyl phenylalanine | 4.12E-11 | 9.61E-12 | 3.92E-12 | | 3.94E-11 | 7.35E-12 | 3E-12 |
| *N*-linoleoyl phenylalanine | 1.21E-11 | 3.46E-12 | 1.41E-12 | | 1.03E-11 | 2.28E-12 | 9.3E-13 |
| *N*-arachidonoyl phenylalanine | 4.96E-11 | 5.93E-12 | 2.42E-12 | | 3.7E-11 | 3.56E-12 | 1.45E-12 |
| *N*-docosahexaenoyl phenylalanine | 7.36E-12 | 1.84E-12 | 7.53E-13 | | 6.53E-12 | 1.38E-12 | 5.65E-13 |

**Supplemental Table 19: Continued**

|  | BV2 | | | | | |
| --- | --- | --- | --- | --- | --- | --- |
|  | Vehicle | | | 1 µM URB597 | | |
| ***N*-acyl proline** | Mean | Std Dev | Std Error | Mean | Std Dev | Std Error |
| *N*-palmitoyl proline | 1.88E-11 | 2.64E-12 | 1.08E-12 | 1.66E-11 | 2.63E-12 | 1.07E-12 |
| *N*-stearoyl proline | 7.01E-12 | 1.5E-12 | 6.12E-13 | 6.47E-12 | 6.25E-13 | 2.55E-13 |
| *N-*oleoyl proline | 3.54E-12 | 8.11E-13 | 3.31E-13 | 2.85E-12 | 6.11E-13 | 2.49E-13 |
| *N*-linoleoyl proline | 1.07E-12 | 1.96E-13 | 8.02E-14 | 1.09E-12 | 2.52E-13 | 1.03E-13 |
| *N*-arachidonoyl proline | 3.63E-12 | 5.37E-13 | 2.19E-13 | 2.04E-12 | 5.27E-13 | 2.15E-13 |
| *N*-docosahexaenoyl proline | PISSR |  |  | PISSR |  |  |
| ***N*-acyl serine** |  |  |  |  |  |  |
| *N*-palmitoyl serine | 2.94E-10 | 5.19E-11 | 2.12E-11 | 3.03E-10 | 2.98E-11 | 1.22E-11 |
| *N*-stearoyl serine | 1.5E-10 | 1.86E-11 | 7.58E-12 | 1.49E-10 | 3.89E-11 | 1.59E-11 |
| *N*-oleoyl serine | 1.06E-10 | 1.6E-11 | 6.55E-12 | 2.14E-10 | 4.06E-11 | 1.66E-11 |
| *N*-linoleoyl serine | 1.36E-10 | 3.48E-11 | 1.42E-11 | 2.92E-10 | 3.23E-11 | 1.32E-11 |
| *N*-arachidonoyl serine | 2.31E-11 | 7.02E-12 | 2.87E-12 | 5.48E-11 | 1.36E-11 | 5.55E-12 |
| *N*-docosahexaenoyl serine | 7.43E-11 | 2.76E-11 | 1.13E-11 | 8.91E-11 | 4.14E-11 | 1.69E-11 |
| ***N*-acyl taurine** |  |  |  |  |  |  |
| *N*-palmitoyl taurine | 1.21E-09 | 1.53E-10 | 6.24E-11 | 2.32E-09 | 3.72E-10 | 1.52E-10 |
| *N*-stearoyl taurine | 1.37E-09 | 2.36E-10 | 9.63E-11 | 3.3E-09 | 6.59E-10 | 2.69E-10 |
| *N*-oleoyl taurine | 6.14E-10 | 1.52E-10 | 6.2E-11 | 1.79E-09 | 3.42E-10 | 1.4E-10 |
| *N*-arachidonoyl taurine | 1.93E-10 | 1.8E-11 | 7.34E-12 | 1.95E-10 | 5.19E-11 | 2.12E-11 |
| ***N*-acyl tryptophan** |  |  |  |  |  |  |
| *N*-palmitoyl tryptophan | 1.21E-10 | 1.91E-11 | 7.78E-12 | 1.12E-10 | 1.75E-11 | 7.14E-12 |
| *N*-stearoyl tryptophan | 2.7E-10 | 5.42E-11 | 2.21E-11 | 2.6E-10 | 2.61E-11 | 1.07E-11 |
| *N*-oleoyl tryptophan | 8.32E-11 | 1.2E-11 | 4.88E-12 | 8.49E-11 | 1.26E-11 | 5.16E-12 |
| *N*-linoleoyl tryptophan | BDL |  |  | BDL |  |  |
| *N*-arachidonoyl tryptophan | PISSR |  |  | PISSR |  |  |
| *N*-docosahexaenoyl tryptophan | PISSR |  |  | PISSR |  |  |
| ***N*-acyl tyrosine** |  |  |  |  |  |  |
| *N*-palmitoyl tyrosine | 2.24E-11 | 4.74E-12 | 1.93E-12 | 2.1E-11 | 5.29E-12 | 2.16E-12 |
| *N*-stearoyl tyrosine | 4.22E-12 | 9.62E-13 | 3.93E-13 | 4.03E-12 | 4.9E-13 | 2E-13 |
| *N-*oleoyl tyrosine | 2.67E-11 | 7.28E-12 | 2.97E-12 | 2.54E-11 | 5.76E-12 | 2.35E-12 |
| *N*-linoleoyl tyrosine | 7.65E-12 | 1.76E-12 | 7.2E-13 | 9.65E-12 | 2.33E-12 | 9.5E-13 |
| *N*-arachidonoyl tyrosine | 5.38E-12 | 1.12E-12 | 4.56E-13 | 3.88E-12 | 7.35E-13 | 3E-13 |
| *N*-docosahexaenoyl tyrosine | 4.21E-12 | 6.53E-13 | 2.67E-13 | 4.49E-12 | 1.08E-12 | 4.39E-13 |
| ***N*-acyl valine** |  |  |  |  |  |  |
| *N*-palmitoyl valine | 1.91E-11 | 4.1E-12 | 1.68E-12 | 1.65E-11 | 8.27E-13 | 3.38E-13 |
| *N*- stearoyl valine | 1.68E-11 | 4.14E-12 | 1.69E-12 | 1.5E-11 | 2.03E-12 | 8.28E-13 |
| *N*-oleoyl valine | 7.98E-12 | 2.52E-12 | 1.03E-12 | 7.9E-12 | 3.25E-12 | 1.33E-12 |
| *N*-nervonoyl valine | BDL |  |  | BDL |  |  |
| *N*-linoleoyl valine | PISSR |  |  | PISSR |  |  |
| *N*-docosahexaenoyl valine | 1.44E-12 | 3.64E-13 | 1.49E-13 | 1.99E-12 | 7.5E-13 | 3.06E-13 |
| **2-acyl glycerols** |  |  |  |  |  |  |
| 2-palmitoyl glycerol | 5.92E-10 | 2.23E-10 | 9.1E-11 | 2.05E-09 | 3.62E-10 | 1.48E-10 |
| 2-oleoyl glycerol | 4.22E-09 | 1.22E-09 | 4.97E-10 | 1.02E-08 | 8.12E-10 | 3.31E-10 |
| 2-linoleoyl glycerol | 1.06E-09 | 2.3E-10 | 9.39E-11 | 1.47E-09 | 2E-10 | 8.17E-11 |
| 2-arachidonoyl glycerol | 1.32E-09 | 3.9E-10 | 1.59E-10 | 1.57E-09 | 2.96E-10 | 1.21E-10 |
| **Free Fatty Acids** |  |  |  |  |  |  |
| Oleic acid | 3.08E-10 | 6.93E-11 | 2.83E-11 | 3.3E-10 | 6.41E-11 | 2.61E-11 |
| Linoleic acid | 1.94E-09 | 5.36E-10 | 2.19E-10 | 1.97E-09 | 4.12E-10 | 1.68E-10 |
| Arachidonic acid | 1.9E-09 | 3.5E-10 | 1.43E-10 | 1.8E-09 | 3.4E-10 | 1.39E-10 |
| **PhosphoLEA** |  |  |  |  |  |  |
| PhosphoLEA | BDL |  |  | BDL |  |  |
| **Prostaglandins** |  |  |  |  |  |  |
| PGE_2_ | 5.88E-09 | 2.24E-09 | 9.13E-10 | 5.13E-09 | 1.19E-09 | 4.86E-10 |
| PGF_2α_ | 2.33E-09 | 6.29E-10 | 2.57E-10 | 2.08E-09 | 2.2E-10 | 8.99E-11 |
| 6-ketoPGF_1α_ | 6.02E-10 | 8.93E-11 | 3.65E-11 | 6.12E-10 | 4.34E-11 | 1.77E-11 |
| **THC/CBD** |  |  |  |  |  |  |
| Cannabidiol | BDL |  |  | BDL |  |  |
| THC | BDL |  |  | BDL |  |  |
| **THC Metabolites** |  |  |  |  |  |  |
| 11-nor-9-carboxyTHC | BDL |  |  | BDL |  |  |
| 11-OH-THC | BDL |  |  | BDL |  |  |

**Supplemental Table 20: List of lipids in BV2 microglial cells significantly affected by 1 µM URB597**

| BV2 URB597 Significant Differences in One-Way ANOVA | | | | |
| --- | --- | --- | --- | --- |
| Lipid | F | p | Direction (relative to Veh) | Magnitude (x Veh level) |
| *N*-oleoyl alanine | 58.93 | .000 | ↑ | 1.31 |
| *N*-linoleoyl alanine | 10.81 | .008 | ↑ | 2.20 |
| *N*-arachidonoyl alanine | 12.06 | .006 | ↓ | 0.67 |
| *N*-palmitoyl ethanolamine | 159.18 | .000 | ↑ | 7.06 |
| *N*-stearoyl ethanolamine | 50.48 | .000 | ↑ | 7.37 |
| *N*-oleoyl ethanolamine | 214.25 | .000 | ↑ | 87.27 |
| *N*-linoleoyl ethanolamine | 104.05 | .000 | ↑ | 20.97 |
| *N*-arachidonoyl ethanolamine | 125.39 | .000 | ↑ | 3.83 |
| *N*-docosahexaenoyl ethanolamine | 198.69 | .000 | ↑ | 6.51 |
| *N*-palmitoyl GABA | 75.16 | .000 | ↑ | 2.16 |
| *N*-stearoyl GABA | 40.44 | .000 | ↑ | 1.79 |
| *N*-oleoyl GABA | 19.96 | .001 | ↑ | 1.69 |
| *N*-linoleoyl GABA | 9.42 | .012 | ↑ | 1.94 |
| *N*-arachidonoyl GABA | 9.86 | .011 | ↓ | 0.58 |
| *N*-docosahexaenoyl GABA | 13.25 | .005 | ↑ | 1.52 |
| *N*-palmitoyl glycine | 13.69 | .004 | ↑ | 1.35 |
| *N*-stearoyl glycine | 80.37 | .000 | ↑ | 1.82 |
| *N*-oleoyl glycine | 152.16 | .000 | ↑ | 2.49 |
| *N*-linoleoyl glycine | 53.07 | .000 | ↑ | 2.17 |
| *N*-arachidonoyl glycine | 25.52 | .000 | ↓ | 0.66 |
| *N*-stearoyl leucine | 5.61 | .039 | ↓ | 0.82 |
| *N*-arachidonoyl methionine | 50.21 | .000 | ↓ | 0.50 |
| *N*-arachidonoyl phenylalanine | 19.97 | .001 | ↓ | 0.75 |
| *N*-arachidonoyl proline | 26.87 | .000 | ↓ | 0.56 |
| *N*-oleoyl serine | 36.50 | .000 | ↑ | 2.02 |
| *N*-linoleoyl serine | 64.86 | .000 | ↑ | 2.15 |
| *N*-arachidonoyl serine | 25.78 | .000 | ↑ | 2.37 |
| *N*-palmitoyl taurine | 45.66 | .000 | ↑ | 1.92 |
| *N*-stearoyl taurine | 46.03 | .000 | ↑ | 2.41 |
| *N*-oleoyl taurine | 59.15 | .000 | ↑ | 2.92 |
| *N*-arachidonoyl tyrosine | 7.60 | .020 | ↓ | 0.72 |
| 2-palmitoyl glycerol | 70.97 | .000 | ↑ | 3.47 |
| 2-oleoyl glycerol | 101.28 | .000 | ↑ | 2.43 |
| 2-linoleoyl glycerol | 11.14 | .008 | ↑ | 1.39 |

Supplemental Table 21: Levels of THC, CBD, and THC metabolites between BV2 cells treated with either drug alone or in combination

Descriptive statistics

|  | | N | Mean | Std. Deviation | Std. Error | 95% Confidence Interval for Mean | | Minimum | Maximum |
| --- | --- | --- | --- | --- | --- | --- | --- | --- | --- |
|  |  |  |  |  |  | Lower Bound | Upper Bound |  |  |
| % Original THC incorporated at 2 hours | 1uM THC | 6 | 4.7785 | .92461 | .37747 | 3.8082 | 5.7488 | 3.71 | 5.96 |
|  | 1uM THC:CBD | 6 | 18.0731 | .96197 | .39272 | 17.0636 | 19.0826 | 16.99 | 19.46 |
|  | Total | 12 | 11.4258 | 7.00091 | 2.02099 | 6.9776 | 15.8740 | 3.71 | 19.46 |
| Ratio of 11-OH-THC to THC incorporated at 2 hours | 1uM THC | 6 | .2173 | .03817 | .01558 | .1772 | .2573 | .17 | .27 |
|  | 1uM THC:CBD | 6 | .5792 | .03560 | .01453 | .5419 | .6166 | .52 | .63 |
|  | Total | 12 | .3982 | .19226 | .05550 | .2761 | .5204 | .17 | .63 |
| Ratio of THC-COOH to THC incorporated at 2 hours | 1uM THC | 6 | .0934 | .02098 | .00856 | .0714 | .1154 | .08 | .14 |
|  | 1uM THC:CBD | 6 | .0484 | .01415 | .00578 | .0336 | .0633 | .03 | .07 |
|  | Total | 12 | .0709 | .02905 | .00838 | .0525 | .0894 | .03 | .14 |
| Ratio of THC-COOH to 11-OH-THC at 2 hours | 1uM THC | 6 | 43.3545 | 7.58623 | 3.09707 | 35.3932 | 51.3158 | 34.07 | 54.31 |
|  | 1uM THC:CBD | 6 | 8.3737 | 2.43389 | .99363 | 5.8194 | 10.9279 | 5.57 | 11.75 |
|  | Total | 12 | 25.8641 | 19.04147 | 5.49680 | 13.7657 | 37.9624 | 5.57 | 54.31 |

| Percent of CBD incorporated after 2 hours | | | | | | | | |
| --- | --- | --- | --- | --- | --- | --- | --- | --- |
|  | N | Mean | Std. Deviation | Std. Error | 95% Confidence Interval for Mean | | Minimum | Maximum |
|  |  |  |  |  | Lower Bound | Upper Bound |  |  |
| 1uM CBD | 6 | 10.9414 | 2.02332 | .82602 | 8.8181 | 13.0647 | 9.05 | 13.65 |
| 1uM THC:CBD | 6 | 16.7780 | 2.16617 | .88434 | 14.5047 | 19.0512 | 14.26 | 19.46 |
| Total | 12 | 13.8597 | 3.64476 | 1.05215 | 11.5439 | 16.1754 | 9.05 | 19.46 |

Supplemental Table 22: ANOVA Comparing levels of THC, CBD and THC metabolites between BV2 cells treated with either drug alone or in combination

ANOVA

|  | | Sum of Squares | df | Mean Square | F | Sig. |
| --- | --- | --- | --- | --- | --- | --- |
| % Original THC incorporated at 2 hours | Between Groups | 530.239 | 1 | 530.239 | 595.679 | .000 |
|  | Within Groups | 8.901 | 10 | .890 |  |  |
|  | Total | 539.140 | 11 |  |  |  |
| Ratio of 11-OH-THC to THC incorporated at 2 hours | Between Groups | .393 | 1 | .393 | 288.526 | .000 |
|  | Within Groups | .014 | 10 | .001 |  |  |
|  | Total | .407 | 11 |  |  |  |
| Ratio of THC-COOH to THC incorporated at 2 hours | Between Groups | .006 | 1 | .006 | 18.989 | .001 |
|  | Within Groups | .003 | 10 | .000 |  |  |
|  | Total | .009 | 11 |  |  |  |
| Ratio of THC-COOH to 11-OH-THC at 2 hours | Between Groups | 3670.978 | 1 | 3670.978 | 115.667 | .000 |
|  | Within Groups | 317.374 | 10 | 31.737 |  |  |
|  | Total | 3988.351 | 11 |  |  |  |

| Percent of CBD incorporated after 2 hours | | | | | |
| --- | --- | --- | --- | --- | --- |
|  | Sum of Squares | df | Mean Square | F | Sig. |
| Between Groups | 102.197 | 1 | 102.197 | 23.263 | .001 |
| Within Groups | 43.931 | 10 | 4.393 |  |  |
| Total | 146.127 | 11 |  |  |  |

Supplemental Table 23: Levels of THC, CBD, and THC metabolites between C6 cells treated with either drug alone or in combination

|  | | N | Mean | Std. Deviation | Std. Error | 95% Confidence Interval for Mean | | Minimum | Maximum |
| --- | --- | --- | --- | --- | --- | --- | --- | --- | --- |
|  |  |  |  |  |  | Lower Bound | Upper Bound |  |  |
| % Original THC incorporated at 2 hours | 1uM THC | 6 | 1.3630 | .19722 | .08052 | 1.1560 | 1.5699 | 1.00 | 1.58 |
|  | 1uM THC:CBD | 6 | 4.0901 | .69932 | .28549 | 3.3563 | 4.8240 | 3.33 | 5.25 |
|  | Total | 12 | 2.7266 | 1.50612 | .43478 | 1.7696 | 3.6835 | 1.00 | 5.25 |
| Ratio of 11-OH-THC to THC incorporated at 2 hours | 1uM THC | 6 | 3.3801 | .55795 | .22778 | 2.7946 | 3.9657 | 2.85 | 4.45 |
|  | 1uM THC:CBD | 6 | 1.2696 | .17049 | .06960 | 1.0907 | 1.4485 | 1.02 | 1.46 |
|  | Total | 12 | 2.3249 | 1.17027 | .33783 | 1.5813 | 3.0684 | 1.02 | 4.45 |
| Ratio of THC-COOH to THC incorporated at 2 hours | 1uM THC | 6 | .9514 | .23250 | .09492 | .7074 | 1.1954 | .58 | 1.24 |
|  | 1uM THC:CBD | 6 | .8761 | .17941 | .07324 | .6878 | 1.0644 | .66 | 1.13 |
|  | Total | 12 | .9138 | .20186 | .05827 | .7855 | 1.0420 | .58 | 1.24 |
| Ratio of THC-COOH to 11-OH-THC at 2 hours | 1uM THC | 6 | 28.6380 | 8.06591 | 3.29289 | 20.1734 | 37.1027 | 18.16 | 38.14 |
|  | 1uM THC:CBD | 6 | 68.8328 | 9.60993 | 3.92324 | 58.7478 | 78.9178 | 62.39 | 87.86 |
|  | Total | 12 | 48.7354 | 22.63125 | 6.53308 | 34.3562 | 63.1147 | 18.16 | 87.86 |

| Percent of CBD incorporated after 2 hours | | | | | | | | |
| --- | --- | --- | --- | --- | --- | --- | --- | --- |
|  | N | Mean | Std. Deviation | Std. Error | 95% Confidence Interval for Mean | | Minimum | Maximum |
|  |  |  |  |  | Lower Bound | Upper Bound |  |  |
| 1uM CBD | 6 | 1.9441 | .34766 | .14193 | 1.5792 | 2.3089 | 1.55 | 2.34 |
| 1uM THC:CBD | 6 | 1.5002 | .14725 | .06011 | 1.3457 | 1.6548 | 1.30 | 1.76 |
| Total | 12 | 1.7221 | .34427 | .09938 | 1.5034 | 1.9409 | 1.30 | 2.34 |

Supplemental Table 24: ANOVA Comparing levels of THC, CBD and THC metabolites between C6 cells treated with either drug alone or in combination

ANOVA

|  | | Sum of Squares | df | Mean Square | F | Sig. |
| --- | --- | --- | --- | --- | --- | --- |
| % Original THC incorporated at 2 hours | Between Groups | 22.313 | 1 | 22.313 | 84.527 | .000 |
|  | Within Groups | 2.640 | 10 | .264 |  |  |
|  | Total | 24.952 | 11 |  |  |  |
| Ratio of 11-OH-THC to THC incorporated at 2 hours | Between Groups | 13.363 | 1 | 13.363 | 78.519 | .000 |
|  | Within Groups | 1.702 | 10 | .170 |  |  |
|  | Total | 15.065 | 11 |  |  |  |
| Ratio of THC-COOH to THC incorporated at 2 hours | Between Groups | .017 | 1 | .017 | .394 | .544 |
|  | Within Groups | .431 | 10 | .043 |  |  |
|  | Total | .448 | 11 |  |  |  |
| Ratio of THC-COOH to 11-OH-THC at 2 hours | Between Groups | 4846.862 | 1 | 4846.862 | 61.583 | .000 |
|  | Within Groups | 787.048 | 10 | 78.705 |  |  |
|  | Total | 5633.910 | 11 |  |  |  |

| Percent of CBD incorporated after 2 hours | | | | | |
| --- | --- | --- | --- | --- | --- |
|  | Sum of Squares | df | Mean Square | F | Sig. |
| Between Groups | .591 | 1 | .591 | 8.291 | .016 |
| Within Groups | .713 | 10 | .071 |  |  |
| Total | 1.304 | 11 |  |  |  |

Supplemental Table 25: Levels of THC, CBD, and THC metabolites between N18 cells treated with either drug alone or in combination

|  | | N | Mean | Std. Deviation | Std. Error | 95% Confidence Interval for Mean | | Minimum | Maximum |
| --- | --- | --- | --- | --- | --- | --- | --- | --- | --- |
|  |  |  |  |  |  | Lower Bound | Upper Bound |  |  |
| % Original THC incorporated at 2 hours | 1uM THC | 6 | 1.0864 | .12060 | .04924 | .9598 | 1.2129 | .85 | 1.15 |
|  | 1uM THC:CBD | 6 | 14.8357 | .71311 | .29113 | 14.0874 | 15.5841 | 13.56 | 15.57 |
|  | Total | 12 | 7.9611 | 7.19691 | 2.07757 | 3.3884 | 12.5338 | .85 | 15.57 |
| Ratio of 11-OH-THC to THC incorporated at 2 hours | 1uM THC | 6 | 2.0552 | .31887 | .13018 | 1.7206 | 2.3899 | 1.74 | 2.67 |
|  | 1uM THC:CBD | 6 | .7212 | .08032 | .03279 | .6369 | .8055 | .64 | .83 |
|  | Total | 12 | 1.3882 | .73109 | .21105 | .9237 | 1.8528 | .64 | 2.67 |
| Ratio of THC-COOH to THC incorporated at 2 hours | 1uM THC | 6 | 2.0379 | .46674 | .19054 | 1.5481 | 2.5277 | 1.54 | 2.80 |
|  | 1uM THC:CBD | 6 | .1705 | .01313 | .00536 | .1567 | .1842 | .15 | .19 |
|  | Total | 12 | 1.1042 | 1.02478 | .29583 | .4531 | 1.7553 | .15 | 2.80 |
| Ratio of THC-COOH to 11-OH-THC at 2 hours | 1uM THC | 6 | 98.8260 | 14.32301 | 5.84734 | 83.7950 | 113.8571 | 85.21 | 124.34 |
|  | 1uM THC:CBD | 6 | 23.8080 | 2.52337 | 1.03016 | 21.1599 | 26.4561 | 20.50 | 27.69 |
|  | Total | 12 | 61.3170 | 40.38530 | 11.65823 | 35.6574 | 86.9766 | 20.50 | 124.34 |

| Percent of CBD incorporated after 2 hours | | | | | | | | |
| --- | --- | --- | --- | --- | --- | --- | --- | --- |
|  | N | Mean | Std. Deviation | Std. Error | 95% Confidence Interval for Mean | | Minimum | Maximum |
|  |  |  |  |  | Lower Bound | Upper Bound |  |  |
| 1uM CBD | 6 | 17.4325 | 1.89187 | .77235 | 15.4471 | 19.4179 | 14.78 | 19.95 |
| 1uM THC:CBD | 6 | 18.6386 | 1.72298 | .70340 | 16.8305 | 20.4468 | 17.10 | 21.70 |
| Total | 12 | 18.0356 | 1.83659 | .53018 | 16.8687 | 19.2025 | 14.78 | 21.70 |

Supplemental Table 26: ANOVA Comparing levels of THC, CBD and THC metabolites between N18 cells treated with either drug alone or in combination

|  | | Sum of Squares | df | Mean Square | F | Sig. |
| --- | --- | --- | --- | --- | --- | --- |
| % Original THC incorporated at 2 hours | Between Groups | 567.135 | 1 | 567.135 | 2168.480 | .000 |
|  | Within Groups | 2.615 | 10 | .262 |  |  |
|  | Total | 569.750 | 11 |  |  |  |
| Ratio of 11-OH-THC to THC incorporated at 2 hours | Between Groups | 5.339 | 1 | 5.339 | 98.749 | .000 |
|  | Within Groups | .541 | 10 | .054 |  |  |
|  | Total | 5.879 | 11 |  |  |  |
| Ratio of THC-COOH to THC incorporated at 2 hours | Between Groups | 10.462 | 1 | 10.462 | 95.973 | .000 |
|  | Within Groups | 1.090 | 10 | .109 |  |  |
|  | Total | 11.552 | 11 |  |  |  |
| Ratio of THC-COOH to 11-OH-THC at 2 hours | Between Groups | 16883.121 | 1 | 16883.121 | 159.639 | .000 |
|  | Within Groups | 1057.580 | 10 | 105.758 |  |  |
|  | Total | 17940.701 | 11 |  |  |  |

| Percent of CBD incorporated after 2 hours | | | | | |
| --- | --- | --- | --- | --- | --- |
|  | Sum of Squares | df | Mean Square | F | Sig. |
| Between Groups | 4.364 | 1 | 4.364 | 1.333 | .275 |
| Within Groups | 32.739 | 10 | 3.274 |  |  |
| Total | 37.104 | 11 |  |  |  |

**Supplemental Table 27: Lipid levels in the striatum of C57 WT female mice treated with Vehicle or 3 mg/kg CBD (CBD_9 outlier)**

|  | Striatum | | | | | | |
| --- | --- | --- | --- | --- | --- | --- | --- |
|  | Vehicle | | | 3mg/kg CBD | | | |
| ***N*-acyl alanine** | Mean | Std Dev | Std Error | | Mean | Std Dev | Std Error |
| *N*-palmitoyl alanine | 1.98E-11 | 4.98E-12 | 1.66E-12 | | 1.75E-11 | 2.18E-12 | 7.7E-13 |
| *N*-stearoyl alanine | 5.24E-12 | 7.09E-13 | 2.36E-13 | | 6.87E-12 | 7.76E-13 | 2.74E-13 |
| *N*-oleoyl alanine | 5.89E-12 | 6.12E-13 | 2.04E-13 | | 6.9E-12 | 8.78E-13 | 3.1E-13 |
| *N*-linoleoyl alanine | 9.73E-13 | 1.57E-13 | 5.25E-14 | | 1.09E-12 | 1.61E-13 | 5.68E-14 |
| *N*-arachidonoyl alanine | 3.35E-12 | 5.22E-13 | 1.74E-13 | | 4.59E-12 | 6.99E-13 | 2.47E-13 |
| *N*-docosahexaenoyl alanine | PISSR |  |  | | PISSR |  |  |
| ***N*-acyl dopamine** |  |  |  | |  |  |  |
| *N*-oleoyl dopamine | PISSR |  |  | | PISSR |  |  |
| *N*-arachidonoyl dopamine | PISSR |  |  | | PISSR |  |  |
| ***N*-acyl ethanolamine** |  |  |  | |  |  |  |
| *N*-palmitoyl ethanolamine | 2.84E-10 | 4.27E-11 | 1.42E-11 | | 2.69E-10 | 6.92E-11 | 2.45E-11 |
| *N*-stearoyl ethanolamine | 7.28E-11 | 1E-11 | 3.35E-12 | | 6.91E-11 | 1.38E-11 | 4.88E-12 |
| *N*-oleoyl ethanolamine | 4.18E-10 | 4.64E-11 | 1.55E-11 | | 4.74E-10 | 8E-11 | 2.83E-11 |
| *N*-linoleoyl ethanolamine | 2.2E-10 | 5.55E-11 | 1.85E-11 | | 2.22E-10 | 6.64E-11 | 2.35E-11 |
| *N*-arachidonoyl ethanolamine | 3.12E-11 | 5.51E-12 | 1.84E-12 | | 3.61E-11 | 7.65E-12 | 2.71E-12 |
| *N*-docosahexaenoyl ethanolamine | 5.21E-11 | 1.02E-11 | 3.39E-12 | | 5.66E-11 | 8.85E-12 | 3.13E-12 |
| ***N*-acyl GABA** |  |  |  | |  |  |  |
| *N*-palmitoyl GABA | 4.86E-12 | 1.09E-12 | 3.62E-13 | | 4.41E-12 | 6.68E-13 | 2.36E-13 |
| *N*-stearoyl GABA | 4.06E-12 | 2.93E-13 | 9.78E-14 | | 5.06E-12 | 3.83E-13 | 1.36E-13 |
| *N*-oleoyl GABA | 1.83E-12 | 2.4E-13 | 8E-14 | | 2.44E-12 | 3.35E-13 | 1.19E-13 |
| *N*-linoleoyl GABA | PISSR |  |  | | PISSR |  |  |
| *N*-arachidonoyl GABA | 7.4E-12 | 6.11E-13 | 2.04E-13 | | 9.38E-12 | 6.58E-13 | 2.33E-13 |
| *N*-docosahexaenoyl GABA | 1.01E-12 | 1.65E-13 | 5.51E-14 | | 9E-13 | 1.23E-13 | 4.35E-14 |
| ***N*-acyl glycine** |  |  |  | |  |  |  |
| *N*-palmitoyl glycine | 1.3E-11 | 2.65E-12 | 8.83E-13 | | 1.45E-11 | 3.51E-12 | 1.24E-12 |
| *N*-stearoyl glycine | 2.78E-12 | 7.37E-13 | 2.46E-13 | | 2.76E-12 | 1.02E-12 | 3.6E-13 |
| *N*-oleoyl glycine | 4.17E-12 | 6.36E-13 | 2.12E-13 | | 5.15E-12 | 5.74E-13 | 2.03E-13 |
| *N*-linoleoyl glycine | 6.02E-13 | 1.7E-13 | 5.67E-14 | | 5.44E-13 | 1.51E-13 | 5.33E-14 |
| *N*-arachidonoyl glycine | 1.35E-11 | 9.46E-13 | 3.15E-13 | | 1.7E-11 | 2.54E-12 | 8.96E-13 |
| *N*-docosahexaenoyl glycine | 9.73E-13 | 1.38E-13 | 4.61E-14 | | 1.39E-12 | 2.05E-13 | 7.26E-14 |
| ***N*-acyl leucine** |  |  |  | |  |  |  |
| *N*-palmitoyl leucine | 4.47E-12 | 5.6E-13 | 1.87E-13 | | 5.33E-12 | 5.38E-13 | 1.9E-13 |
| *N*-stearoyl leucine | 5.77E-12 | 1.01E-12 | 3.36E-13 | | 4.96E-12 | 1.31E-12 | 4.64E-13 |
| *N*-oleoyl leucine | 5.51E-12 | 9.69E-13 | 3.23E-13 | | 4.5E-12 | 9.25E-13 | 3.27E-13 |
| *N*-linoleoyl leucine | 1.63E-12 | 5.56E-13 | 1.85E-13 | | 1.13E-12 | 2.9E-13 | 1.02E-13 |
| *N*-docosahexaenoyl leucine | PISSR |  |  | | PISSR |  |  |
| ***N*-acyl methionine** |  |  |  | |  |  |  |
| *N*-palmitoyl methionine | 3.87E-12 | 1.06E-12 | 3.53E-13 | | 4.93E-12 | 1.18E-12 | 4.18E-13 |
| *N*-stearoyl methionine | 1.53E-12 | 5.26E-13 | 1.75E-13 | | 1.63E-12 | 3.4E-13 | 1.2E-13 |
| *N*-oleoyl methionine | 1.87E-12 | 5.18E-13 | 1.73E-13 | | 1.58E-12 | 9.59E-13 | 3.39E-13 |
| *N*-linoleoyl methionine | PISSR |  |  | | PISSR |  |  |
| *N*-arachidonoyl methionine | PISSR |  |  | | PISSR |  |  |
| *N*-docosahexaenoyl methionine | BDL |  |  | | BDL |  |  |
| ***N*-acyl phenylalanine** |  |  |  | |  |  |  |
| *N*-palmitoyl phenylalanine | 2.93E-12 | 8.26E-13 | 2.75E-13 | | 2.65E-12 | 9.88E-13 | 3.49E-13 |
| *N*-stearoyl phenylalanine | 1.04E-12 | 4.01E-13 | 1.34E-13 | | 8.8E-13 | 4.83E-13 | 1.71E-13 |
| *N*-oleoyl phenylalanine | 7.93E-13 | 9.58E-14 | 3.19E-14 | | 1.13E-12 | 3.52E-13 | 1.24E-13 |
| *N*-linoleoyl phenylalanine | PISSR |  |  | | PISSR |  |  |
| *N*-arachidonoyl phenylalanine | 7.37E-13 | 8.06E-14 | 2.69E-14 | | 1.3E-12 | 3.48E-13 | 1.23E-13 |
| *N*-docosahexaenoyl phenylalanine | 1.28E-12 | 5.63E-13 | 1.88E-13 | | 1.71E-12 | 8.11E-13 | 2.87E-13 |

**Supplemental Table 27: Continued**

|  | Striatum | | | | | |
| --- | --- | --- | --- | --- | --- | --- |
|  | Vehicle | | | 3mg/kg CBD | | |
| ***N*-acyl proline** | Mean | Std Dev | Std Error | Mean | Std Dev | Std Error |
| *N*-palmitoyl proline | PISSR |  |  | PISSR |  |  |
| *N*-stearoyl proline | PISSR |  |  | PISSR |  |  |
| *N-*oleoyl proline | PISSR |  |  | PISSR |  |  |
| *N*-linoleoyl proline | BDL |  |  | BDL |  |  |
| *N*-arachidonoyl proline | BDL |  |  | BDL |  |  |
| *N*-docosahexaenoyl proline | BDL |  |  | BDL |  |  |
| ***N*-acyl serine** |  |  |  |  |  |  |
| *N*-palmitoyl serine | 1.25E-11 | 4.17E-12 | 1.39E-12 | 1.24E-11 | 2.83E-12 | 1E-12 |
| *N*-stearoyl serine | 1.96E-11 | 4.56E-12 | 1.52E-12 | 1.9E-11 | 3.73E-12 | 1.32E-12 |
| *N*-oleoyl serine | 3.42E-10 | 6.29E-11 | 2.1E-11 | 3.19E-10 | 8.19E-11 | 2.9E-11 |
| *N*-linoleoyl serine | 8.64E-11 | 1.11E-11 | 3.68E-12 | 7.67E-11 | 1.51E-11 | 5.33E-12 |
| *N*-arachidonoyl serine | 5.23E-13 | 1.01E-13 | 3.37E-14 | 8.11E-13 | 1.55E-13 | 5.49E-14 |
| *N*-docosahexaenoyl serine | 1.84E-12 | 9.47E-13 | 3.16E-13 | 1.54E-12 | 5.82E-13 | 2.06E-13 |
| ***N*-acyl taurine** |  |  |  |  |  |  |
| *N*-arachidonoyl taurine | 2.77E-11 | 4.16E-12 | 1.39E-12 | 2.78E-11 | 5.36E-12 | 1.89E-12 |
| ***N*-acyl tryptophan** |  |  |  |  |  |  |
| *N*-palmitoyl tryptophan | 1.14E-12 | 5.13E-13 | 1.71E-13 | 1.2E-12 | 4.62E-13 | 1.63E-13 |
| *N*-stearoyl tryptophan | 7.44E-12 | 3.2E-12 | 1.07E-12 | 6.82E-12 | 1.16E-12 | 4.1E-13 |
| *N*-oleoyl tryptophan | BDL |  |  | BDL |  |  |
| *N*-linoleoyl tryptophan | BDL |  |  | BDL |  |  |
| *N*-arachidonoyl tryptophan | BDL |  |  | BDL |  |  |
| *N*-docosahexaenoyl tryptophan | BDL |  |  | BDL |  |  |
| ***N*-acyl tyrosine** |  |  |  |  |  |  |
| *N*-palmitoyl tyrosine | 2.94E-13 | 1.11E-13 | 3.7E-14 | 2.76E-13 | 1.28E-13 | 4.53E-14 |
| *N*-stearoyl tyrosine | 5.42E-14 | 3.33E-14 | 1.11E-14 | 5.05E-14 | 2.72E-14 | 9.63E-15 |
| *N-*oleoyl tyrosine | 2.54E-13 | 1.5E-13 | 4.99E-14 | 2.23E-13 | 8.67E-14 | 3.06E-14 |
| *N*-linoleoyl tyrosine | PISSR |  |  | PISSR |  |  |
| *N*-arachidonoyl tyrosine | PISSR |  |  | PISSR |  |  |
| *N*-docosahexaenoyl tyrosine | PISSR |  |  | PISSR |  |  |
| ***N*-acyl valine** |  |  |  |  |  |  |
| *N*-palmitoyl valine | 2.87E-13 | 1.67E-13 | 5.56E-14 | 4.04E-13 | 1.8E-13 | 6.37E-14 |
| *N*- stearoyl valine | 2.76E-13 | 9.83E-14 | 3.28E-14 | 2.2E-13 | 8.02E-14 | 2.83E-14 |
| *N*-oleoyl valine | 1.83E-13 | 8.7E-14 | 2.9E-14 | 1.47E-13 | 6.12E-14 | 2.16E-14 |
| *N*-nervonoyl valine | BDL |  |  | BDL |  |  |
| *N*-linoleoyl valine | BDL |  |  | BDL |  |  |
| *N*-docosahexaenoyl valine | PISSR |  |  | PISSR |  |  |
| **2-acyl glycerols** |  |  |  |  |  |  |
| 2-palmitoyl glycerol | 3.67E-10 | 9.24E-11 | 3.08E-11 | 4.85E-10 | 7E-11 | 2.47E-11 |
| 2-oleoyl glycerol | 5.21E-09 | 1.41E-09 | 4.7E-10 | 6.52E-09 | 1.55E-09 | 5.47E-10 |
| 2-linoleoyl glycerol | 3.04E-11 | 5.99E-12 | 2E-12 | 4.06E-11 | 8.08E-12 | 2.86E-12 |
| 2-arachidonoyl glycerol | 4.69E-10 | 4.57E-11 | 1.52E-11 | 5.83E-10 | 1.27E-10 | 4.5E-11 |
| **Free Fatty Acids** |  |  |  |  |  |  |
| Oleic acid | 2.37E-08 | 4.07E-09 | 1.36E-09 | 3.98E-08 | 4.96E-09 | 1.76E-09 |
| Linoleic acid | 8.6E-10 | 3.06E-11 | 1.02E-11 | 1.11E-09 | 1.1E-10 | 3.87E-11 |
| Arachidonic acid | 1.21E-08 | 7.41E-10 | 2.47E-10 | 1.49E-08 | 3.03E-09 | 1.07E-09 |
| **PhosphoLEA** |  |  |  |  |  |  |
| PhosphoLEA | 2.17E-11 | 6.27E-12 | 2.09E-12 | 3.49E-11 | 8.15E-12 | 2.88E-12 |
| **Prostaglandins** |  |  |  |  |  |  |
| PGE_2_ | 1.96E-10 | 1.79E-11 | 5.97E-12 | 1.56E-10 | 1.36E-11 | 4.82E-12 |
| PGF_2α_ | 2.01E-10 | 1.66E-11 | 5.54E-12 | 1.45E-10 | 1.74E-11 | 6.14E-12 |
| 6-ketoPGF_1α_ | 4.76E-12 | 7.21E-13 | 2.4E-13 | 2.34E-12 | 7.83E-13 | 2.77E-13 |
| **THC/CBD** |  |  |  |  |  |  |
| Cannabidiol | BDL |  |  | 3.92E-11 | 9.7E-12 | 3.43E-12 |
| THC | BDL |  |  | BDL |  |  |
| **THC Metabolites** |  |  |  |  |  |  |
| 11-nor-9-carboxyTHC | BDL |  |  | BDL |  |  |
| 11-OH-THC | BDL |  |  | BDL |  |  |

**Supplemental Table 28: List of lipids in the WT striatum significantly affected by 3 mg/kg CBD**

| WT CBD Striatum Significant Differences in One-Way ANOVA | | | | |
| --- | --- | --- | --- | --- |
| Lipid | F | p | Direction (relative to Veh) | Magnitude (x Veh level) |
| *N*-stearoyl alanine | 20.50 | .000 | ↑ | 1.31 |
| *N*-oleoyl alanine | 7.76 | .014 | ↑ | 1.17 |
| *N*-arachidonoyl alanine | 17.49 | .001 | ↑ | 1.37 |
| *N*-oleoyl ethanolamine | 3.24 | .092 | ↑ | 1.13 |
| *N*-stearoyl GABA | 37.05 | .000 | ↑ | 1.25 |
| *N*-oleoyl GABA | 19.13 | .001 | ↑ | 1.33 |
| *N*-arachidonoyl GABA | 41.79 | .000 | ↑ | 1.27 |
| *N*-oleoyl glycine | 10.96 | .005 | ↑ | 1.24 |
| *N*-arachidonoyl glycine | 15.05 | .001 | ↑ | 1.26 |
| *N*-docosahexaenoyl glycine | 24.23 | .000 | ↑ | 1.43 |
| *N*-palmitoyl leucine | 10.49 | .006 | ↑ | 1.19 |
| *N*-oleoyl leucine | 4.87 | .043 | ↓ | 0.82 |
| *N*-linoleoyl leucine | 5.24 | .037 | ↓ | 0.69 |
| *N*-palmitoyl methionine | 3.85 | .069 | ↑ | 1.27 |
| *N*-oleoyl phenylalanine | 7.47 | .015 | ↑ | 1.43 |
| *N*-arachidonoyl phenylalanine | 22.66 | .000 | ↑ | 1.76 |
| *N*-arachidonoyl serine | 20.94 | .000 | ↑ | 1.55 |
| 2-palmitoyl glycerol | 8.77 | .010 | ↑ | 1.32 |
| 2-oleoyl glycerol | 3.30 | .090 | ↑ | 1.25 |
| 2-linleoyl glycerol | 8.85 | .009 | ↑ | 1.34 |
| 2-arachidonoyl glycerol | 6.35 | .024 | ↑ | 1.24 |
| Oleic acid | 54.04 | .000 | ↑ | 1.68 |
| Linoleic acid | 42.75 | .000 | ↑ | 1.29 |
| Arachidonic acid | 6.89 | .019 | ↑ | 1.23 |
| phosphoLEA | 14.25 | .002 | ↑ | 1.61 |
| PGE_2_ | 26.47 | .000 | ↓ | 0.80 |
| PGF_2α_ | 45.98 | .000 | ↓ | 0.72 |
| 6-ketoPGF_1α_ | 44.29 | .000 | ↓ | 0.49 |
| CBD | 148.56 | .000 | ↑ | infinite |
| Sample Mass | 6.40 | .023 | ↑ | 1.13 |

**Supplemental Table 29: Lipid levels in the hippocampus of C57 WT female mice treated with Vehicle or 3 mg/kg CBD**

|  | Hippocampus | | | | | | |
| --- | --- | --- | --- | --- | --- | --- | --- |
|  | Vehicle | | | 3mg/kg CBD | | | |
| ***N*-acyl alanine** | Mean | Std Dev | Std Error | | Mean | Std Dev | Std Error |
| *N*-palmitoyl alanine | 2.53E-11 | 1.94E-12 | 6.45E-13 | | 2.65E-11 | 2.55E-12 | 8.51E-13 |
| *N*-stearoyl alanine | 8.6E-12 | 1.16E-12 | 3.88E-13 | | 1.02E-11 | 1.11E-12 | 3.71E-13 |
| *N*-oleoyl alanine | 1.99E-11 | 1.52E-12 | 5.08E-13 | | 1.84E-11 | 1.79E-12 | 5.98E-13 |
| *N*-linoleoyl alanine | 5.31E-12 | 1.05E-12 | 3.51E-13 | | 5.28E-12 | 9.49E-13 | 3.16E-13 |
| *N*-arachidonoyl alanine | 1.27E-11 | 2.07E-12 | 6.92E-13 | | 1.31E-11 | 2.5E-12 | 8.32E-13 |
| *N*-docosahexaenoyl alanine | 1.74E-12 | 6.77E-13 | 2.26E-13 | | 1.96E-12 | 6.66E-13 | 2.22E-13 |
| ***N*-acyl dopamine** |  |  |  | |  |  |  |
| *N*-oleoyl dopamine | BDL |  |  | | BDL |  |  |
| *N*-arachidonoyl dopamine | BDL |  |  | | BDL |  |  |
| ***N*-acyl ethanolamine** |  |  |  | |  |  |  |
| *N*-palmitoyl ethanolamine | 3.11E-10 | 9.67E-11 | 3.22E-11 | | 3.63E-10 | 1.17E-10 | 3.91E-11 |
| *N*-stearoyl ethanolamine | 6.56E-11 | 2.53E-11 | 8.43E-12 | | 7.38E-11 | 2.03E-11 | 6.75E-12 |
| *N*-oleoyl ethanolamine | 5.72E-10 | 7.19E-11 | 2.4E-11 | | 7.04E-10 | 1.61E-10 | 5.37E-11 |
| *N*-linoleoyl ethanolamine | 1.53E-10 | 2.75E-11 | 9.16E-12 | | 1.87E-10 | 4.24E-11 | 1.41E-11 |
| *N*-arachidonoyl ethanolamine | 7.36E-11 | 1.48E-11 | 4.94E-12 | | 9.82E-11 | 2.61E-11 | 8.71E-12 |
| *N*-docosahexaenoyl ethanolamine | 6.79E-11 | 1.96E-11 | 6.55E-12 | | 8.82E-11 | 1.92E-11 | 6.39E-12 |
| ***N*-acyl GABA** |  |  |  | |  |  |  |
| *N*-palmitoyl GABA | 8.67E-12 | 1.19E-12 | 3.97E-13 | | 9.72E-12 | 1.41E-12 | 4.7E-13 |
| *N*-stearoyl GABA | 8.25E-12 | 1.79E-12 | 5.95E-13 | | 7.95E-12 | 1.49E-12 | 4.98E-13 |
| *N*-oleoyl GABA | 6.67E-12 | 1.07E-12 | 3.56E-13 | | 7.98E-12 | 1.69E-12 | 5.62E-13 |
| *N*-linoleoyl GABA | 1.11E-12 | 3.73E-13 | 1.24E-13 | | 1.67E-12 | 5.88E-13 | 1.96E-13 |
| *N*-arachidonoyl GABA | 4.64E-11 | 1.8E-12 | 6E-13 | | 5.24E-11 | 6.39E-12 | 2.13E-12 |
| *N*-docosahexaenoyl GABA | 1.99E-12 | 2.95E-13 | 9.83E-14 | | 2.6E-12 | 5.73E-13 | 1.91E-13 |
| ***N*-acyl glycine** |  |  |  | |  |  |  |
| *N*-palmitoyl glycine | 1.85E-11 | 3.67E-12 | 1.22E-12 | | 1.88E-11 | 3.88E-12 | 1.29E-12 |
| *N*-stearoyl glycine | 6.19E-12 | 6.97E-13 | 2.32E-13 | | 6.45E-12 | 9.21E-13 | 3.07E-13 |
| *N*-oleoyl glycine | 9.91E-12 | 1.31E-12 | 4.38E-13 | | 1.18E-11 | 1.75E-12 | 5.83E-13 |
| *N*-linoleoyl glycine | 1.51E-12 | 1.49E-13 | 4.97E-14 | | 2.12E-12 | 3.11E-13 | 1.04E-13 |
| *N*-arachidonoyl glycine | 5.91E-11 | 2.32E-12 | 7.74E-13 | | 7.1E-11 | 8E-12 | 2.67E-12 |
| *N*-docosahexaenoyl glycine | 3.11E-12 | 3.98E-13 | 1.33E-13 | | 3.92E-12 | 4.95E-13 | 1.65E-13 |
| ***N*-acyl leucine** |  |  |  | |  |  |  |
| *N*-palmitoyl leucine | 3.69E-12 | 6.66E-13 | 2.22E-13 | | 4.17E-12 | 8.12E-13 | 2.71E-13 |
| *N*-stearoyl leucine | 5.03E-12 | 9.62E-13 | 3.21E-13 | | 5E-12 | 7.07E-13 | 2.36E-13 |
| *N*-oleoyl leucine | 5.26E-12 | 1.27E-12 | 4.25E-13 | | 5.77E-12 | 9.14E-13 | 3.05E-13 |
| *N*-linoleoyl leucine | 1.34E-12 | 2.74E-13 | 9.15E-14 | | 1.32E-12 | 4.23E-13 | 1.41E-13 |
| *N*-docosahexaenoyl leucine | 6.06E-13 | 1.5E-13 | 4.99E-14 | | 5.68E-13 | 1.68E-13 | 5.61E-14 |
| ***N*-acyl methionine** |  |  |  | |  |  |  |
| *N*-palmitoyl methionine | 7.75E-12 | 3.84E-12 | 1.28E-12 | | 7.95E-12 | 2.35E-12 | 7.85E-13 |
| *N*-stearoyl methionine | 1.6E-12 | 3.94E-13 | 1.31E-13 | | 1.53E-12 | 6.25E-13 | 2.08E-13 |
| *N*-oleoyl methionine | 1.25E-12 | 4.88E-13 | 1.63E-13 | | 1.29E-12 | 2.9E-13 | 9.66E-14 |
| *N*-linoleoyl methionine | BDL |  |  | | BDL |  |  |
| *N*-arachidonoyl methionine | 1.06E-12 | 2.49E-13 | 8.29E-14 | | 1.52E-12 | 1.39E-13 | 4.64E-14 |
| *N*-docosahexaenoyl methionine | PISSR |  |  | | PISSR |  |  |
| ***N*-acyl phenylalanine** |  |  |  | |  |  |  |
| *N*-palmitoyl phenylalanine | 2.62E-12 | 7.44E-13 | 2.48E-13 | | 2.57E-12 | 1.05E-12 | 3.51E-13 |
| *N*-stearoyl phenylalanine | 9.97E-13 | 3.59E-13 | 1.2E-13 | | 9.55E-13 | 5.1E-13 | 1.7E-13 |
| *N*-oleoyl phenylalanine | 1.09E-12 | 4.73E-13 | 1.58E-13 | | 1.02E-12 | 5.33E-13 | 1.78E-13 |
| *N*-linoleoyl phenylalanine | 2.23E-13 | 8.06E-14 | 2.69E-14 | | 3.05E-13 | 7.72E-14 | 2.57E-14 |
| *N*-arachidonoyl phenylalanine | 1.55E-12 | 2.8E-13 | 9.32E-14 | | 1.9E-12 | 4.96E-13 | 1.65E-13 |
| *N*-docosahexaenoyl phenylalanine | 1.41E-12 | 6.32E-13 | 2.11E-13 | | 1.85E-12 | 5.81E-13 | 1.94E-13 |

**Supplemental Table 29: Continued**

|  | Hippocampus | | | | | |
| --- | --- | --- | --- | --- | --- | --- |
|  | Vehicle | | | 3mg/kg CBD | | |
| ***N*-acyl proline** | Mean | Std Dev | Std Error | Mean | Std Dev | Std Error |
| *N*-palmitoyl proline | PISSR |  |  | PISSR |  |  |
| *N*-stearoyl proline | PISSR |  |  | PISSR |  |  |
| *N-*oleoyl proline | PISSR |  |  | PISSR |  |  |
| *N*-linoleoyl proline | BDL |  |  | BDL |  |  |
| *N*-arachidonoyl proline | BDL |  |  | BDL |  |  |
| *N*-docosahexaenoyl proline | BDL |  |  | BDL |  |  |
| ***N*-acyl serine** |  |  |  |  |  |  |
| *N*-palmitoyl serine | 3.85E-11 | 5.54E-12 | 1.85E-12 | 3.74E-11 | 6.05E-12 | 2.02E-12 |
| *N*-stearoyl serine | 3.29E-12 | 9.61E-13 | 3.2E-13 | 3.6E-12 | 8.48E-13 | 2.83E-13 |
| *N*-oleoyl serine | 4.15E-10 | 5.57E-11 | 1.86E-11 | 4.17E-10 | 3.43E-11 | 1.14E-11 |
| *N*-linoleoyl serine | 9.18E-11 | 1.13E-11 | 3.77E-12 | 8.94E-11 | 5.48E-12 | 1.83E-12 |
| *N*-arachidonoyl serine | 1.67E-12 | 2.62E-13 | 8.73E-14 | 2.58E-12 | 2.7E-13 | 9.01E-14 |
| *N*-docosahexaenoyl serine | 4.33E-12 | 9.6E-13 | 3.2E-13 | 5.83E-12 | 7.98E-13 | 2.66E-13 |
| ***N*-acyl taurine** |  |  |  |  |  |  |
| *N*-arachidonoyl taurine | 1.18E-10 | 1.96E-11 | 6.55E-12 | 1.16E-10 | 2.34E-11 | 7.8E-12 |
| ***N*-acyl tryptophan** |  |  |  |  |  |  |
| *N*-palmitoyl tryptophan | 9.64E-13 | 1.96E-13 | 6.53E-14 | 1.33E-12 | 2.48E-13 | 8.26E-14 |
| *N*-stearoyl tryptophan | 5.45E-12 | 2.17E-12 | 7.22E-13 | 6.17E-12 | 1.47E-12 | 4.89E-13 |
| *N*-oleoyl tryptophan | PISSR |  |  | PISSR |  |  |
| *N*-linoleoyl tryptophan | BDL |  |  | BDL |  |  |
| *N*-arachidonoyl tryptophan | BDL |  |  | BDL |  |  |
| *N*-docosahexaenoyl tryptophan | BDL |  |  | BDL |  |  |
| ***N*-acyl tyrosine** |  |  |  |  |  |  |
| *N*-palmitoyl tyrosine | 5.59E-13 | 3.29E-13 | 1.1E-13 | 6.44E-13 | 4.26E-13 | 1.42E-13 |
| *N*-stearoyl tyrosine | 6.9E-14 | 5.27E-14 | 1.76E-14 | 9.55E-14 | 4.13E-14 | 1.38E-14 |
| *N-*oleoyl tyrosine | 4.14E-13 | 2.34E-13 | 7.79E-14 | 3.49E-13 | 2.31E-13 | 7.68E-14 |
| *N*-linoleoyl tyrosine | PISSR |  |  | PISSR |  |  |
| *N*-arachidonoyl tyrosine | 1.39E-13 | 5.7E-14 | 1.9E-14 | 1.97E-13 | 5.6E-14 | 1.87E-14 |
| *N*-docosahexaenoyl tyrosine | PISSR |  |  | PISSR |  |  |
| ***N*-acyl valine** |  |  |  |  |  |  |
| *N*-palmitoyl valine | 3.09E-13 | 1.29E-13 | 4.3E-14 | 4.18E-13 | 1.03E-13 | 3.45E-14 |
| *N*- stearoyl valine | 1.84E-13 | 1.16E-13 | 3.87E-14 | 1.7E-13 | 8.4E-14 | 2.8E-14 |
| *N*-oleoyl valine | 1.76E-13 | 6.95E-14 | 2.32E-14 | 1.72E-13 | 5.89E-14 | 1.96E-14 |
| *N*-nervonoyl valine | BDL |  |  | BDL |  |  |
| *N*-linoleoyl valine | PISSR |  |  | PISSR |  |  |
| *N*-docosahexaenoyl valine | PISSR |  |  | PISSR |  |  |
| **2-acyl glycerols** |  |  |  |  |  |  |
| 2-palmitoyl glycerol | 6.77E-10 | 9.05E-11 | 3.02E-11 | 6.3E-10 | 1.59E-10 | 5.31E-11 |
| 2-oleoyl glycerol | 9.85E-09 | 3.54E-09 | 1.18E-09 | 1.01E-08 | 3.98E-09 | 1.33E-09 |
| 2-linoleoyl glycerol | 4.05E-11 | 7.01E-12 | 2.34E-12 | 4.81E-11 | 8.29E-12 | 2.76E-12 |
| 2-arachidonoyl glycerol | 2.61E-09 | 1.71E-10 | 5.68E-11 | 2.72E-09 | 4.33E-10 | 1.44E-10 |
| **Free Fatty Acids** |  |  |  |  |  |  |
| Oleic acid | 1.23E-07 | 5.61E-08 | 1.87E-08 | 2.14E-07 | 7.69E-08 | 2.56E-08 |
| Linoleic acid | 1.24E-09 | 2.27E-10 | 7.58E-11 | 1.88E-09 | 4.07E-10 | 1.36E-10 |
| Arachidonic acid | 2E-08 | 2.36E-09 | 7.85E-10 | 2.42E-08 | 3.2E-09 | 1.07E-09 |
| **PhosphoLEA** |  |  |  |  |  |  |
| PhosphoLEA | 2.76E-11 | 6.4E-12 | 2.13E-12 | 2.71E-11 | 4.41E-12 | 1.47E-12 |
| **Prostaglandins** |  |  |  |  |  |  |
| PGE_2_ | 4.61E-10 | 5.02E-11 | 1.67E-11 | 4.14E-10 | 3.05E-11 | 1.02E-11 |
| PGF_2α_ | 4.29E-10 | 3.89E-11 | 1.3E-11 | 3.61E-10 | 2.75E-11 | 9.16E-12 |
| 6-ketoPGF_1α_ | 3.53E-12 | 4.7E-13 | 1.57E-13 | 2.94E-12 | 2.62E-13 | 8.74E-14 |
| **THC/CBD** |  |  |  |  |  |  |
| Cannabidiol | BDL |  |  | 3.56E-11 | 1E-11 | 3.34E-12 |
| THC | BDL |  |  | BDL |  |  |
| **THC Metabolites** |  |  |  |  |  |  |
| 11-nor-9-carboxyTHC | BDL |  |  | BDL |  |  |
| 11-OH-THC | BDL |  |  | BDL |  |  |

**Supplemental Table 30: List of lipids in the WT hippocampus significantly affected by 3 mg/kg CBD**

| WT CBD Hippocampus Significant Differences in One-Way ANOVA | | | | |
| --- | --- | --- | --- | --- |
| Lipid | F | p | Direction (relative to Veh) | Magnitude (x Veh level) |
| *N*-stearoyl alanine | 8.54 | .010 | ↑ | 1.19 |
| *N*-oleoyl alanine | 4.08 | .060 | ↓ | 0.92 |
| *N*-oleoyl ethanolamine | 4.97 | .041 | ↑ | 1.23 |
| *N*-linoleoyl ethanolamine | 3.93 | .065 | ↑ | 1.22 |
| *N*-arachidonoyl ethanolamine | 6.03 | .026 | ↑ | 1.33 |
| *N*-docosahexaenoyl ethanolamine | 4.92 | .041 | ↑ | 1.30 |
| *N*-oleoyl GABA | 3.84 | .068 | ↑ | 1.20 |
| *N*-linoleoyl GABA | 5.76 | .029 | ↑ | 1.51 |
| *N*-arachidonoyl GABA | 7.37 | .015 | ↑ | 1.13 |
| *N*-docosahexaenoyl GABA | 8.15 | .011 | ↑ | 1.31 |
| *N*-oleoyl glycine | 6.86 | .019 | ↑ | 1.19 |
| *N*-linoleoyl glycine | 28.93 | .000 | ↑ | 1.40 |
| *N*-arachidonoyl glycine | 18.22 | .001 | ↑ | 1.20 |
| *N*-docosahexaenoyl glycine | 14.76 | .001 | ↑ | 1.26 |
| *N*-arachidonoyl methionine | 24.09 | .000 | ↑ | 1.43 |
| *N*-linoleoyl phenylalanine | 4.84 | .043 | ↑ | 1.37 |
| *N*-arachidonoyl phenylalanine | 3.44 | .082 | ↑ | 1.23 |
| *N*-arachidonoyl serine | 52.88 | .000 | ↑ | 1.54 |
| *N*-docosahexaenoyl serine | 12.93 | .002 | ↑ | 1.35 |
| *N*-palmitoyl tryptophan | 12.04 | .003 | ↑ | 1.39 |
| *N*-arachidonoyl tyrosine | 4.70 | .046 | ↑ | 1.42 |
| *N*-palmitoyl valine | 3.91 | .065 | ↑ | 1.35 |
| 2-linleoyl glycerol | 4.33 | .054 | ↑ | 1.19 |
| Oleic acid | 8.20 | .011 | ↑ | 1.74 |
| Linoleic acid | 16.91 | .001 | ↑ | 1.52 |
| Arachidonic acid | 9.95 | .006 | ↑ | 1.21 |
| PGE_2_ | 5.92 | .027 | ↓ | 0.90 |
| PGF_2α_ | 18.75 | .001 | ↓ | 0.84 |
| 6-ketoPGF_1α_ | 10.91 | .004 | ↓ | 0.83 |
| CBD | 113.45 | .000 | ↑ | infinite |

**Supplemental Table 31: Lipid levels in the cerebellum of C57 WT female mice treated with Vehicle or 3 mg/kg CBD**

|  | Cerebellum | | | | | | |
| --- | --- | --- | --- | --- | --- | --- | --- |
|  | Vehicle | | | 3mg/kg CBD | | | |
| ***N*-acyl alanine** | Mean | Std Dev | Std Error | | Mean | Std Dev | Std Error |
| *N*-palmitoyl alanine | 1.51E-11 | 1.91E-12 | 6.36E-13 | | 1.54E-11 | 2.86E-12 | 9.52E-13 |
| *N*-stearoyl alanine | 1.97E-11 | 2.52E-12 | 8.4E-13 | | 2.11E-11 | 5.33E-12 | 1.78E-12 |
| *N*-oleoyl alanine | 1.64E-11 | 3.07E-12 | 1.02E-12 | | 1.35E-11 | 3.71E-12 | 1.24E-12 |
| *N*-linoleoyl alanine | 5.21E-12 | 1.79E-12 | 5.98E-13 | | 4.58E-12 | 1.46E-12 | 4.87E-13 |
| *N*-arachidonoyl alanine | 7.33E-12 | 9.36E-13 | 3.12E-13 | | 7.26E-12 | 1.44E-12 | 4.81E-13 |
| *N*-docosahexaenoyl alanine | 4.14E-12 | 8.22E-13 | 2.74E-13 | | 4.51E-12 | 9.91E-13 | 3.3E-13 |
| ***N*-acyl dopamine** |  |  |  | |  |  |  |
| *N*-oleoyl dopamine | BDL |  |  | | BDL |  |  |
| *N*-arachidonoyl dopamine | BDL |  |  | | BDL |  |  |
| ***N*-acyl ethanolamine** |  |  |  | |  |  |  |
| *N*-palmitoyl ethanolamine | 4.04E-10 | 1.09E-10 | 3.85E-11 | | 4.54E-10 | 7.99E-11 | 2.82E-11 |
| *N*-stearoyl ethanolamine | 9.91E-11 | 2.09E-11 | 7.37E-12 | | 1.04E-10 | 2.15E-11 | 7.59E-12 |
| *N*-oleoyl ethanolamine | 7E-10 | 1.37E-10 | 4.85E-11 | | 7.05E-10 | 1.83E-10 | 6.09E-11 |
| *N*-linoleoyl ethanolamine | 1.24E-10 | 2.19E-11 | 7.29E-12 | | 1.54E-10 | 2.7E-11 | 9.54E-12 |
| *N*-arachidonoyl ethanolamine | 3.84E-11 | 6.1E-12 | 2.03E-12 | | 4.83E-11 | 6.56E-12 | 2.32E-12 |
| *N*-docosahexaenoyl ethanolamine | 1.54E-10 | 3.48E-11 | 1.16E-11 | | 1.97E-10 | 5.42E-11 | 1.92E-11 |
| ***N*-acyl GABA** |  |  |  | |  |  |  |
| *N*-palmitoyl GABA | 2E-11 | 2.58E-12 | 8.61E-13 | | 2.07E-11 | 3.43E-12 | 1.14E-12 |
| *N*-stearoyl GABA | 2.48E-11 | 2.73E-12 | 9.1E-13 | | 2.59E-11 | 3.42E-12 | 1.14E-12 |
| *N*-oleoyl GABA | 1.77E-11 | 2.27E-12 | 7.55E-13 | | 1.89E-11 | 2.72E-12 | 9.08E-13 |
| *N*-linoleoyl GABA | 6.47E-12 | 1.09E-12 | 3.62E-13 | | 6.87E-12 | 1.39E-12 | 4.64E-13 |
| *N*-arachidonoyl GABA | 2.4E-11 | 2.18E-12 | 7.27E-13 | | 2.43E-11 | 3.46E-12 | 1.15E-12 |
| *N*-docosahexaenoyl GABA | 8.11E-12 | 6.65E-13 | 2.22E-13 | | 8.84E-12 | 8.61E-13 | 2.87E-13 |
| ***N*-acyl glycine** |  |  |  | |  |  |  |
| *N*-palmitoyl glycine | 1.86E-11 | 3.12E-12 | 1.04E-12 | | 1.81E-11 | 4.2E-12 | 1.4E-12 |
| *N*-stearoyl glycine | 9.86E-12 | 1.69E-12 | 5.63E-13 | | 1.14E-11 | 2.1E-12 | 6.99E-13 |
| *N*-oleoyl glycine | 7.89E-12 | 1.29E-12 | 4.31E-13 | | 7.99E-12 | 1.22E-12 | 4.08E-13 |
| *N*-linoleoyl glycine | 9.32E-13 | 1.11E-13 | 3.7E-14 | | 1.09E-12 | 1.82E-13 | 6.06E-14 |
| *N*-arachidonoyl glycine | 1.38E-11 | 2.01E-12 | 6.71E-13 | | 1.38E-11 | 2.24E-12 | 7.46E-13 |
| *N*-docosahexaenoyl glycine | 3.45E-12 | 3.74E-13 | 1.25E-13 | | 3.75E-12 | 6.46E-13 | 2.15E-13 |
| ***N*-acyl leucine** |  |  |  | |  |  |  |
| *N*-palmitoyl leucine | 8.73E-13 | 6.86E-14 | 2.29E-14 | | 9.32E-13 | 1.47E-13 | 4.89E-14 |
| *N*-stearoyl leucine | 1.08E-12 | 1.29E-13 | 4.3E-14 | | 1.11E-12 | 1.97E-13 | 6.57E-14 |
| *N*-oleoyl leucine | 3.83E-13 | 3.45E-14 | 1.15E-14 | | 4.37E-13 | 7.87E-14 | 2.62E-14 |
| *N*-linoleoyl leucine | 2.09E-13 | 6.04E-14 | 2.01E-14 | | 2.24E-13 | 8.62E-14 | 2.87E-14 |
| *N*-docosahexaenoyl leucine | 6.18E-13 | 9.51E-14 | 3.17E-14 | | 6.25E-13 | 9.32E-14 | 3.11E-14 |
| ***N*-acyl methionine** |  |  |  | |  |  |  |
| *N*-palmitoyl methionine | 7.35E-12 | 2.17E-12 | 7.23E-13 | | 7.2E-12 | 1.44E-12 | 4.81E-13 |
| *N*-stearoyl methionine | 1.97E-12 | 3.23E-13 | 1.08E-13 | | 2.14E-12 | 3.6E-13 | 1.2E-13 |
| *N*-oleoyl methionine | 1.13E-12 | 1.32E-13 | 4.4E-14 | | 1.25E-12 | 3.46E-13 | 1.15E-13 |
| *N*-linoleoyl methionine | BDL |  |  | | BDL |  |  |
| *N*-arachidonoyl methionine | 8.37E-13 | 9.62E-14 | 3.21E-14 | | 1.13E-12 | 2.01E-13 | 6.69E-14 |
| *N*-docosahexaenoyl methionine | PISSR |  |  | | PISSR |  |  |
| ***N*-acyl phenylalanine** |  |  |  | |  |  |  |
| *N*-palmitoyl phenylalanine | 3.58E-12 | 1E-12 | 3.35E-13 | | 3.46E-12 | 9.15E-13 | 3.05E-13 |
| *N*-stearoyl phenylalanine | 2.16E-12 | 6.71E-13 | 2.24E-13 | | 2.16E-12 | 6.06E-13 | 2.02E-13 |
| *N*-oleoyl phenylalanine | 2.23E-12 | 6.86E-13 | 2.29E-13 | | 2.38E-12 | 1.14E-12 | 3.81E-13 |
| *N*-linoleoyl phenylalanine | 3.26E-13 | 1.67E-13 | 5.57E-14 | | 3.33E-13 | 1.55E-13 | 5.18E-14 |
| *N*-arachidonoyl phenylalanine | 9.97E-13 | 2.63E-13 | 8.75E-14 | | 1.29E-12 | 5.34E-13 | 1.78E-13 |
| *N*-docosahexaenoyl phenylalanine | 1.04E-12 | 4.15E-13 | 1.38E-13 | | 9.74E-13 | 1.78E-13 | 5.95E-14 |

**Supplemental Table 31: Continued**

|  | Cerebellum | | | | | |
| --- | --- | --- | --- | --- | --- | --- |
|  | Vehicle | | | 3mg/kg CBD | | |
| ***N*-acyl proline** | Mean | Std Dev | Std Error | Mean | Std Dev | Std Error |
| *N*-palmitoyl proline | 3.77E-13 | 8.55E-14 | 2.85E-14 | 5.34E-13 | 1.14E-13 | 3.8E-14 |
| *N*-stearoyl proline | 1.71E-13 | 2.09E-14 | 6.98E-15 | 1.87E-13 | 4.24E-14 | 1.41E-14 |
| *N-*oleoyl proline | 1.33E-13 | 3E-14 | 1E-14 | 1.41E-13 | 4.9E-14 | 1.63E-14 |
| *N*-linoleoyl proline | PISSR |  |  | PISSR |  |  |
| *N*-arachidonoyl proline | PISSR |  |  | PISSR |  |  |
| *N*-docosahexaenoyl proline | PISSR |  |  | PISSR |  |  |
| ***N*-acyl serine** |  |  |  |  |  |  |
| *N*-palmitoyl serine | 9.83E-11 | 1.28E-11 | 4.25E-12 | 1.09E-10 | 1.24E-11 | 4.13E-12 |
| *N*-stearoyl serine | 4.52E-11 | 6.73E-12 | 2.24E-12 | 4.62E-11 | 8.35E-12 | 2.78E-12 |
| *N*-oleoyl serine | 4.41E-10 | 5.96E-11 | 1.99E-11 | 5.09E-10 | 6.41E-11 | 2.14E-11 |
| *N*-linoleoyl serine | 1.06E-10 | 1.69E-11 | 5.65E-12 | 1.13E-10 | 1.83E-11 | 6.11E-12 |
| *N*-arachidonoyl serine | 9.04E-12 | 8.23E-13 | 2.74E-13 | 1.25E-11 | 1.54E-12 | 5.12E-13 |
| *N*-docosahexaenoyl serine | 3.82E-11 | 5.48E-12 | 1.83E-12 | 4.98E-11 | 9.71E-12 | 3.24E-12 |
| ***N*-acyl taurine** |  |  |  |  |  |  |
| *N*-arachidonoyl taurine | 4.61E-11 | 2.98E-12 | 9.94E-13 | 3.91E-11 | 3.35E-12 | 1.12E-12 |
| ***N*-acyl tryptophan** |  |  |  |  |  |  |
| *N*-palmitoyl tryptophan | 2.06E-12 | 6.07E-13 | 2.02E-13 | 2.11E-12 | 6.02E-13 | 2.01E-13 |
| *N*-stearoyl tryptophan | 4.52E-12 | 5.69E-13 | 1.9E-13 | 4.86E-12 | 8.84E-13 | 2.95E-13 |
| *N*-oleoyl tryptophan | 9.18E-13 | 3.36E-13 | 1.12E-13 | 9.7E-13 | 2.24E-13 | 7.47E-14 |
| *N*-linoleoyl tryptophan | PISSR |  |  | PISSR |  |  |
| *N*-arachidonoyl tryptophan | PISSR |  |  | PISSR |  |  |
| *N*-docosahexaenoyl tryptophan | PISSR |  |  | PISSR |  |  |
| ***N*-acyl tyrosine** |  |  |  |  |  |  |
| *N*-palmitoyl tyrosine | 1.59E-12 | 6.88E-13 | 2.29E-13 | 1.84E-12 | 6.96E-13 | 2.32E-13 |
| *N*-stearoyl tyrosine | 1.48E-13 | 4.23E-14 | 1.41E-14 | 1.69E-13 | 5.29E-14 | 1.76E-14 |
| *N-*oleoyl tyrosine | 1.17E-12 | 4.68E-13 | 1.56E-13 | 1.09E-12 | 3.85E-13 | 1.28E-13 |
| *N*-linoleoyl tyrosine | PISSR |  |  | PISSR |  |  |
| *N*-arachidonoyl tyrosine | 1.56E-12 | 6.24E-13 | 2.08E-13 | 1.58E-12 | 7.69E-13 | 2.56E-13 |
| *N*-docosahexaenoyl tyrosine | 1.79E-12 | 5.55E-13 | 1.85E-13 | 1.97E-12 | 9.43E-13 | 3.14E-13 |
| ***N*-acyl valine** |  |  |  |  |  |  |
| *N*-palmitoyl valine | 6.17E-13 | 1.04E-13 | 3.48E-14 | 6.88E-13 | 1.8E-13 | 6E-14 |
| *N*- stearoyl valine | 7.37E-13 | 1.46E-13 | 4.86E-14 | 7.91E-13 | 2.22E-13 | 7.41E-14 |
| *N*-oleoyl valine | 2.93E-13 | 7.85E-14 | 2.62E-14 | 3.09E-13 | 4.12E-14 | 1.37E-14 |
| *N*-nervonoyl valine | BDL |  |  | BDL |  |  |
| *N*-linoleoyl valine | PISSR |  |  | PISSR |  |  |
| *N*-docosahexaenoyl valine | PISSR |  |  | PISSR |  |  |
| **2-acyl glycerols** |  |  |  |  |  |  |
| 2-palmitoyl glycerol | 1.67E-08 | 8.27E-09 | 2.76E-09 | 1.44E-08 | 7.74E-09 | 2.58E-09 |
| 2-oleoyl glycerol | 1.64E-08 | 7.39E-09 | 2.46E-09 | 1.59E-08 | 6.95E-09 | 2.32E-09 |
| 2-linoleoyl glycerol | 2.18E-10 | 6.75E-11 | 2.25E-11 | 1.97E-10 | 5.18E-11 | 1.73E-11 |
| 2-arachidonoyl glycerol | 1.33E-09 | 5.11E-10 | 1.81E-10 | 1.26E-09 | 6E-10 | 2E-10 |
| **Free Fatty Acids** |  |  |  |  |  |  |
| Oleic acid | 1.54E-09 | 2.03E-10 | 6.78E-11 | 2.06E-09 | 2.94E-10 | 9.81E-11 |
| Linoleic acid | 2.09E-10 | 2.64E-11 | 8.8E-12 | 2.28E-10 | 3.88E-11 | 1.29E-11 |
| Arachidonic acid | 1.49E-09 | 1.11E-10 | 3.69E-11 | 1.56E-09 | 1.87E-10 | 6.25E-11 |
| **PhosphoLEA** |  |  |  |  |  |  |
| PhosphoLEA | 4.02E-11 | 6.91E-12 | 2.3E-12 | 5.33E-11 | 7.84E-12 | 2.61E-12 |
| **Prostaglandins** |  |  |  |  |  |  |
| PGE_2_ | 2.73E-10 | 4.18E-11 | 1.39E-11 | 2.75E-10 | 5.64E-11 | 1.88E-11 |
| PGF_2α_ | 2.95E-10 | 2.09E-11 | 6.97E-12 | 2.35E-10 | 3.55E-11 | 1.18E-11 |
| 6-ketoPGF_1α_ | 1.16E-11 | 1.57E-12 | 5.24E-13 | 8.14E-12 | 1.22E-12 | 4.06E-13 |
| **THC/CBD** |  |  |  |  |  |  |
| Cannabidiol | BDL |  |  | 3.46E-11 | 9.78E-12 | 3.26E-12 |
| THC | BDL |  |  | BDL |  |  |
| **THC Metabolites** |  |  |  |  |  |  |
| 11-nor-9-carboxyTHC | BDL |  |  | BDL |  |  |
| 11-OH-THC | BDL |  |  | BDL |  |  |

**Supplemental Table 32: List of lipids in the WT cerebellum significantly affected by 3 mg/kg CBD**

| WT CBD Cerebellum Significant Differences in One-Way ANOVA | | | | |
| --- | --- | --- | --- | --- |
| Lipid | F | p | Direction (relative to Veh) | Magnitude (x Veh level) |
| *N*-oleoyl alanine | 3.11 | .097 | ↓ | 0.82 |
| *N*-linoleoyl ethanolamine | 6.70 | .021 | ↑ | 1.24 |
| *N*-arachidonoyl ethanolamine | 10.47 | .006 | ↑ | 1.26 |
| *N*-docosahexaenoyl ethanolamine | 3.88 | .068 | ↑ | 1.28 |
| *N*-docosahexaenoyl GABA | 4.02 | .062 | ↑ | 1.09 |
| *N*-linoleoyl glycine | 4.83 | .043 | ↑ | 1.17 |
| *N*-oleoyl leucine | 3.59 | .076 | ↑ | 1.14 |
| *N*-arachidonoyl methionine | 15.17 | .001 | ↑ | 1.35 |
| *N*-palmitoyl proline | 10.96 | .004 | ↑ | 1.42 |
| *N*-palmitoyl serine | 3.19 | .093 | ↑ | 1.11 |
| *N*-oleoyl serine | 5.47 | .033 | ↑ | 1.16 |
| *N*-arachidonoyl serine | 35.83 | .000 | ↑ | 1.39 |
| *N*-docosahexaenoyl serine | 9.77 | .007 | ↑ | 1.30 |
| *N*-arachidonoyl taurine | 21.71 | .000 | ↓ | 0.85 |
| Oleic acid | 19.15 | .000 | ↑ | 1.34 |
| phosphoLEA | 14.07 | .002 | ↑ | 1.33 |
| PGF_2α_ | 19.05 | .000 | ↓ | 0.80 |
| 6-ketoPGF_1α_ | 27.06 | .000 | ↓ | 0.70 |
| CBD | 112.71 | .000 | ↑ | infinite |

**Supplemental Table 33: Lipid levels in the thalamus of C57 WT female mice treated with Vehicle or 3 mg/kg CBD**

|  | Thalamus | | | | | | |
| --- | --- | --- | --- | --- | --- | --- | --- |
|  | Vehicle | | | 3mg/kg CBD | | | |
| ***N*-acyl alanine** | Mean | Std Dev | Std Error | | Mean | Std Dev | Std Error |
| *N*-palmitoyl alanine | 1.37E-11 | 2.29E-12 | 7.64E-13 | | 1.39E-11 | 1.49E-12 | 4.96E-13 |
| *N*-stearoyl alanine | 1.09E-11 | 1.16E-12 | 3.86E-13 | | 1.07E-11 | 9.94E-13 | 3.31E-13 |
| *N*-oleoyl alanine | 1.23E-11 | 2.97E-12 | 9.89E-13 | | 9.71E-12 | 3.84E-12 | 1.28E-12 |
| *N*-linoleoyl alanine | 4.87E-12 | 1.42E-12 | 4.74E-13 | | 3.53E-12 | 1.33E-12 | 4.44E-13 |
| *N*-arachidonoyl alanine | 6.18E-12 | 3.01E-13 | 1E-13 | | 6.9E-12 | 7.44E-13 | 2.48E-13 |
| *N*-docosahexaenoyl alanine | 2.24E-12 | 4.73E-13 | 1.58E-13 | | 2.71E-12 | 3.59E-13 | 1.2E-13 |
| ***N*-acyl dopamine** |  |  |  | |  |  |  |
| *N*-oleoyl dopamine | BDL |  |  | | BDL |  |  |
| *N*-arachidonoyl dopamine | BDL |  |  | | BDL |  |  |
| ***N*-acyl ethanolamine** |  |  |  | |  |  |  |
| *N*-palmitoyl ethanolamine | 3.05E-10 | 4.57E-11 | 1.52E-11 | | 3.67E-10 | 5.75E-11 | 1.92E-11 |
| *N*-stearoyl ethanolamine | 5.24E-11 | 7.03E-12 | 2.34E-12 | | 6.32E-11 | 4.82E-12 | 1.61E-12 |
| *N*-oleoyl ethanolamine | 6.29E-10 | 1.07E-10 | 3.58E-11 | | 1.02E-09 | 2.24E-10 | 7.47E-11 |
| *N*-linoleoyl ethanolamine | 6.83E-11 | 1.46E-11 | 4.88E-12 | | 8.42E-11 | 1.8E-11 | 6E-12 |
| *N*-arachidonoyl ethanolamine | 3.35E-11 | 6.13E-12 | 2.04E-12 | | 4.05E-11 | 6.78E-12 | 2.26E-12 |
| *N*-docosahexaenoyl ethanolamine | 8.07E-11 | 1.33E-11 | 4.42E-12 | | 1.08E-10 | 2.57E-11 | 8.56E-12 |
| ***N*-acyl GABA** |  |  |  | |  |  |  |
| *N*-palmitoyl GABA | 2.16E-11 | 2.27E-12 | 7.56E-13 | | 2.19E-11 | 1.97E-12 | 6.55E-13 |
| *N*-stearoyl GABA | 2.86E-11 | 2.89E-12 | 9.63E-13 | | 2.8E-11 | 1.87E-12 | 6.24E-13 |
| *N*-oleoyl GABA | 1.65E-11 | 1.62E-12 | 5.39E-13 | | 1.74E-11 | 1.35E-12 | 4.49E-13 |
| *N*-linoleoyl GABA | 6.4E-12 | 8.38E-13 | 2.79E-13 | | 6.38E-12 | 9.47E-13 | 3.16E-13 |
| *N*-arachidonoyl GABA | 2.94E-11 | 1.51E-12 | 5.04E-13 | | 3.17E-11 | 2.04E-12 | 6.8E-13 |
| *N*-docosahexaenoyl GABA | 6.87E-12 | 9.63E-13 | 3.21E-13 | | 6.94E-12 | 7.36E-13 | 2.45E-13 |
| ***N*-acyl glycine** |  |  |  | |  |  |  |
| *N*-palmitoyl glycine | 1.77E-11 | 9.97E-13 | 3.32E-13 | | 1.9E-11 | 3.8E-12 | 1.27E-12 |
| *N*-stearoyl glycine | 8.06E-12 | 1.76E-12 | 5.85E-13 | | 7.73E-12 | 1.11E-12 | 3.7E-13 |
| *N*-oleoyl glycine | 6.01E-12 | 7.46E-13 | 2.49E-13 | | 7.09E-12 | 6.44E-13 | 2.15E-13 |
| *N*-linoleoyl glycine | 5.31E-13 | 7.07E-14 | 2.36E-14 | | 6.77E-13 | 8.44E-14 | 2.81E-14 |
| *N*-arachidonoyl glycine | 1.04E-11 | 7.91E-13 | 2.64E-13 | | 1.22E-11 | 1.64E-12 | 5.47E-13 |
| *N*-docosahexaenoyl glycine | 1.49E-12 | 2.15E-13 | 7.17E-14 | | 1.85E-12 | 1.86E-13 | 6.19E-14 |
| ***N*-acyl leucine** |  |  |  | |  |  |  |
| *N*-palmitoyl leucine | 1.11E-12 | 9.17E-14 | 3.06E-14 | | 1.21E-12 | 8.36E-14 | 2.79E-14 |
| *N*-stearoyl leucine | 1.33E-12 | 8.87E-14 | 2.96E-14 | | 1.41E-12 | 1.1E-13 | 3.65E-14 |
| *N*-oleoyl leucine | 4.54E-13 | 3.74E-14 | 1.25E-14 | | 4.69E-13 | 4.58E-14 | 1.53E-14 |
| *N*-linoleoyl leucine | 2.28E-13 | 6.41E-14 | 2.14E-14 | | 2.39E-13 | 9.42E-14 | 3.14E-14 |
| *N*-docosahexaenoyl leucine | 5.62E-13 | 1.25E-13 | 4.15E-14 | | 6.41E-13 | 6.82E-14 | 2.27E-14 |
| ***N*-acyl methionine** |  |  |  | |  |  |  |
| *N*-palmitoyl methionine | 1.14E-11 | 3.14E-12 | 1.05E-12 | | 1.04E-11 | 1.93E-12 | 6.45E-13 |
| *N*-stearoyl methionine | 3.22E-12 | 4.72E-13 | 1.57E-13 | | 3.41E-12 | 3.9E-13 | 1.3E-13 |
| *N*-oleoyl methionine | 1.6E-12 | 4.35E-13 | 1.45E-13 | | 1.53E-12 | 3.89E-13 | 1.3E-13 |
| *N*-linoleoyl methionine | PISSR |  |  | | PISSR |  |  |
| *N*-arachidonoyl methionine | 7.64E-13 | 1.03E-13 | 3.45E-14 | | 8.92E-13 | 1.37E-13 | 4.55E-14 |
| *N*-docosahexaenoyl methionine | PISSR |  |  | | PISSR |  |  |
| ***N*-acyl phenylalanine** |  |  |  | |  |  |  |
| *N*-palmitoyl phenylalanine | 4.13E-12 | 1.23E-12 | 4.09E-13 | | 4.23E-12 | 1.55E-12 | 5.16E-13 |
| *N*-stearoyl phenylalanine | 2.73E-12 | 6.51E-13 | 2.17E-13 | | 2.87E-12 | 8.98E-13 | 2.99E-13 |
| *N*-oleoyl phenylalanine | 2.53E-12 | 9.29E-13 | 3.1E-13 | | 2.58E-12 | 7.1E-13 | 2.37E-13 |
| *N*-linoleoyl phenylalanine | 2.22E-13 | 1.01E-13 | 3.38E-14 | | 2.81E-13 | 1.22E-13 | 4.05E-14 |
| *N*-arachidonoyl phenylalanine | 9.79E-13 | 8.41E-14 | 2.8E-14 | | 1.34E-12 | 2.4E-13 | 8.01E-14 |
| *N*-docosahexaenoyl phenylalanine | 9.19E-13 | 3.19E-13 | 1.06E-13 | | 9.23E-13 | 5.14E-13 | 1.71E-13 |

**Supplemental Table 33: Continued**

|  | Thalamus | | | | | |
| --- | --- | --- | --- | --- | --- | --- |
|  | Vehicle | | | 3mg/kg CBD | | |
| ***N*-acyl proline** | Mean | Std Dev | Std Error | Mean | Std Dev | Std Error |
| *N*-palmitoyl proline | 4.13E-13 | 1.01E-13 | 3.38E-14 | 3.78E-13 | 7.63E-14 | 2.54E-14 |
| *N*-stearoyl proline | PISSR |  |  | PISSR |  |  |
| *N-*oleoyl proline | PISSR |  |  | PISSR |  |  |
| *N*-linoleoyl proline | BDL |  |  | BDL |  |  |
| *N*-arachidonoyl proline | BDL |  |  | BDL |  |  |
| *N*-docosahexaenoyl proline | BDL |  |  | BDL |  |  |
| ***N*-acyl serine** |  |  |  |  |  |  |
| *N*-palmitoyl serine | 1.69E-10 | 2E-11 | 6.65E-12 | 1.9E-10 | 2.03E-11 | 6.76E-12 |
| *N*-stearoyl serine | 4.23E-11 | 4.94E-12 | 1.65E-12 | 4.23E-11 | 5.14E-12 | 1.71E-12 |
| *N*-oleoyl serine | 4.56E-10 | 4.1E-11 | 1.37E-11 | 5.24E-10 | 4.66E-11 | 1.55E-11 |
| *N*-linoleoyl serine | 1.07E-10 | 1.32E-11 | 4.39E-12 | 1.2E-10 | 1.28E-11 | 4.25E-12 |
| *N*-arachidonoyl serine | 8.78E-12 | 7.56E-13 | 2.52E-13 | 1.16E-11 | 1.25E-12 | 4.17E-13 |
| *N*-docosahexaenoyl serine | 2.7E-11 | 4.09E-12 | 1.36E-12 | 3.52E-11 | 7.19E-12 | 2.4E-12 |
| ***N*-acyl taurine** |  |  |  |  |  |  |
| *N*-arachidonoyl taurine | 3.46E-11 | 2.37E-12 | 7.9E-13 | 3.56E-11 | 3.19E-12 | 1.06E-12 |
| ***N*-acyl tryptophan** |  |  |  |  |  |  |
| *N*-palmitoyl tryptophan | 3.93E-12 | 9.41E-13 | 3.14E-13 | 4E-12 | 1.63E-12 | 5.42E-13 |
| *N*-stearoyl tryptophan | 7.25E-12 | 1.15E-12 | 3.82E-13 | 7.19E-12 | 1.26E-12 | 4.2E-13 |
| *N*-oleoyl tryptophan | PISSR |  |  | PISSR |  |  |
| *N*-linoleoyl tryptophan | BDL |  |  | BDL |  |  |
| *N*-arachidonoyl tryptophan | PISSR |  |  | PISSR |  |  |
| *N*-docosahexaenoyl tryptophan | PISSR |  |  | PISSR |  |  |
| ***N*-acyl tyrosine** |  |  |  |  |  |  |
| *N*-palmitoyl tyrosine | 1.45E-12 | 6.18E-13 | 2.06E-13 | 1.58E-12 | 6.54E-13 | 2.18E-13 |
| *N*-stearoyl tyrosine | 2.37E-13 | 8.74E-14 | 2.91E-14 | 2.38E-13 | 7.71E-14 | 2.57E-14 |
| *N-*oleoyl tyrosine | 8.42E-13 | 2.92E-13 | 9.73E-14 | 9.2E-13 | 4.02E-13 | 1.34E-13 |
| *N*-linoleoyl tyrosine | PISSR |  |  | PISSR |  |  |
| *N*-arachidonoyl tyrosine | 8.53E-13 | 1.67E-13 | 5.57E-14 | 9.98E-13 | 1.75E-13 | 5.83E-14 |
| *N*-docosahexaenoyl tyrosine | PISSR |  |  | PISSR |  |  |
| ***N*-acyl valine** |  |  |  |  |  |  |
| *N*-palmitoyl valine | 7.28E-13 | 9.18E-14 | 3.06E-14 | 8.39E-13 | 1.57E-13 | 5.22E-14 |
| *N*- stearoyl valine | 7.1E-13 | 1.25E-13 | 4.16E-14 | 8.61E-13 | 1.85E-13 | 6.15E-14 |
| *N*-oleoyl valine | 3.29E-13 | 8.64E-14 | 2.88E-14 | 3.13E-13 | 8.14E-14 | 2.71E-14 |
| *N*-nervonoyl valine | BDL |  |  | BDL |  |  |
| *N*-linoleoyl valine | PISSR |  |  | PISSR |  |  |
| *N*-docosahexaenoyl valine | PISSR |  |  | PISSR |  |  |
| **2-acyl glycerols** |  |  |  |  |  |  |
| 2-palmitoyl glycerol | 2.42E-09 | 3.97E-10 | 1.32E-10 | 2.91E-09 | 7.18E-10 | 2.39E-10 |
| 2-oleoyl glycerol | 3.25E-08 | 7.41E-09 | 2.47E-09 | 4.01E-08 | 9.87E-09 | 3.29E-09 |
| 2-linoleoyl glycerol | 2.09E-10 | 4.75E-11 | 1.58E-11 | 2.55E-10 | 6.93E-11 | 2.31E-11 |
| 2-arachidonoyl glycerol | 2.19E-09 | 5.4E-10 | 1.8E-10 | 2.25E-09 | 8.5E-10 | 2.83E-10 |
| **Free Fatty Acids** |  |  |  |  |  |  |
| Oleic acid | 3.54E-09 | 5.79E-10 | 1.93E-10 | 4.17E-09 | 6.31E-10 | 2.1E-10 |
| Linoleic acid | 5.71E-10 | 7.94E-11 | 2.65E-11 | 6.71E-10 | 1.02E-10 | 3.38E-11 |
| Arachidonic acid | 2.94E-09 | 1.79E-10 | 5.96E-11 | 3.2E-09 | 1.59E-10 | 5.3E-11 |
| **PhosphoLEA** |  |  |  |  |  |  |
| PhosphoLEA | 6.47E-11 | 9.27E-12 | 3.09E-12 | 8.17E-11 | 9.17E-12 | 3.06E-12 |
| **Prostaglandins** |  |  |  |  |  |  |
| PGE_2_ | 4.67E-10 | 7.32E-11 | 2.44E-11 | 4.41E-10 | 2.89E-11 | 9.64E-12 |
| PGF_2α_ | 5.96E-10 | 2.37E-11 | 7.88E-12 | 4.91E-10 | 3.74E-11 | 1.25E-11 |
| 6-ketoPGF_1α_ | 1.45E-11 | 1.46E-12 | 4.86E-13 | 9.75E-12 | 1.25E-12 | 4.17E-13 |
| **THC/CBD** |  |  |  |  |  |  |
| Cannabidiol | BDL |  |  | 3.55E-11 | 9.76E-12 | 3.25E-12 |
| THC | BDL |  |  | BDL |  |  |
| **THC Metabolites** |  |  |  |  |  |  |
| 11-nor-9-carboxyTHC | BDL |  |  | BDL |  |  |
| 11-OH-THC | BDL |  |  | BDL |  |  |

**Supplemental Table 34: List of lipids in the WT thalamus significantly affected by 3 mg/kg CBD**

| WT CBD Thalamus Significant Differences in One-Way ANOVA | | | | |
| --- | --- | --- | --- | --- |
| Lipid | F | p | Direction (relative to Veh) | Magnitude (x Veh level) |
| *N*-linoleoyl alanine | 4.29 | .055 | ↓ | 0.72 |
| *N*-arachidonoyl alanine | 7.19 | .016 | ↑ | 1.12 |
| *N*-docosahexaenoyl alanine | 5.70 | .030 | ↑ | 1.21 |
| *N*-palmitoyl ethanolamine | 6.55 | .021 | ↑ | 1.20 |
| *N*-stearoyl ethanolamine | 14.69 | .001 | ↑ | 1.21 |
| *N*-oleoyl ethanolamine | 22.09 | .000 | ↑ | 1.62 |
| *N*-linoleoyl ethanolamine | 4.21 | .057 | ↑ | 1.23 |
| *N*-arachidonoyl ethanolamine | 5.33 | .035 | ↑ | 1.21 |
| *N*-docosahexaenoyl ethanolamine | 8.08 | .012 | ↑ | 1.33 |
| *N*-arachidonoyl GABA | 7.54 | .014 | ↑ | 1.08 |
| *N*-oleoyl glycine | 10.71 | .005 | ↑ | 1.18 |
| *N*-linoleoyl glycine | 15.99 | .001 | ↑ | 1.27 |
| *N*-arachidonoyl glycine | 8.77 | .009 | ↑ | 1.17 |
| *N*-docosahexaenoyl glycine | 14.05 | .002 | ↑ | 1.24 |
| *N*-palmitoyl leucine | 5.15 | .037 | ↑ | 1.09 |
| *N*-arachidonoyl methionine | 4.98 | .040 | ↑ | 1.17 |
| *N*-arachidonoyl phenylalanine | 17.89 | .001 | ↑ | 1.37 |
| *N*-palmitoyl serine | 4.98 | .040 | ↑ | 1.12 |
| *N*-oleoyl serine | 10.92 | .004 | ↑ | 1.15 |
| *N*-linoleoyl serine | 4.20 | .057 | ↑ | 1.12 |
| *N*-arachidonoyl serine | 33.35 | .000 | ↑ | 1.32 |
| *N*-docosahexaenoyl serine | 8.75 | .009 | ↑ | 1.30 |
| *N*-arachidonoyl tyrosine | 3.23 | .091 | ↑ | 1.17 |
| 2-palmitoyl glycerol | 3.09 | .098 | ↑ | 1.20 |
| 2-oleoyl glycerol | 3.37 | .085 | ↑ | 1.23 |
| Oleic acid | 4.83 | .043 | ↑ | 1.18 |
| Linoleic acid | 5.46 | .033 | ↑ | 1.18 |
| Arachidonic acid | 9.97 | .006 | ↑ | 1.09 |
| phosphoLEA | 15.18 | .001 | ↑ | 1.26 |
| PGF_2α_ | 50.00 | .000 | ↓ | 0.82 |
| 6-ketoPGF_1α_ | 55.86 | .000 | ↓ | 0.67 |
| CBD | 118.81 | .000 | ↑ | infinite |

**Supplemental Table 35: Lipid levels in the cortex of C57 WT female mice treated with Vehicle or 3 mg/kg CBD**

|  | Cortex | | | | | | |
| --- | --- | --- | --- | --- | --- | --- | --- |
|  | Vehicle | | | 3mg/kg CBD | | | |
| ***N*-acyl alanine** | Mean | Std Dev | Std Error | | Mean | Std Dev | Std Error |
| *N*-palmitoyl alanine | 4.61E-12 | 1.26E-12 | 4.2E-13 | | 4.51E-12 | 1.11E-12 | 3.69E-13 |
| *N*-stearoyl alanine | 6.97E-12 | 1.06E-12 | 3.53E-13 | | 6.85E-12 | 1.32E-12 | 4.39E-13 |
| *N*-oleoyl alanine | 6.69E-12 | 1.14E-12 | 3.81E-13 | | 4.56E-12 | 1.15E-12 | 3.85E-13 |
| *N*-linoleoyl alanine | 1.57E-12 | 6.02E-13 | 2.01E-13 | | 1.05E-12 | 2.3E-13 | 7.67E-14 |
| *N*-arachidonoyl alanine | 2.62E-12 | 3.3E-13 | 1.1E-13 | | 3.2E-12 | 5.34E-13 | 1.78E-13 |
| *N*-docosahexaenoyl alanine | 1.18E-12 | 5.23E-13 | 1.74E-13 | | 1.11E-12 | 3.75E-13 | 1.25E-13 |
| ***N*-acyl dopamine** |  |  |  | |  |  |  |
| *N*-oleoyl dopamine | BDL |  |  | | BDL |  |  |
| *N*-arachidonoyl dopamine | BDL |  |  | | BDL |  |  |
| ***N*-acyl ethanolamine** |  |  |  | |  |  |  |
| *N*-palmitoyl ethanolamine | 7.33E-11 | 8.82E-12 | 3.12E-12 | | 9.24E-11 | 9.7E-12 | 3.23E-12 |
| *N*-stearoyl ethanolamine | 2.01E-11 | 2.4E-12 | 8.47E-13 | | 2.55E-11 | 4.87E-12 | 1.72E-12 |
| *N*-oleoyl ethanolamine | 2.72E-10 | 7.19E-11 | 2.54E-11 | | 4.19E-10 | 6.91E-11 | 2.3E-11 |
| *N*-linoleoyl ethanolamine | 8.04E-11 | 1.01E-11 | 3.37E-12 | | 1.15E-10 | 2.29E-11 | 7.64E-12 |
| *N*-arachidonoyl ethanolamine | 2.87E-11 | 3.94E-12 | 1.31E-12 | | 3.82E-11 | 6.26E-12 | 2.09E-12 |
| *N*-docosahexaenoyl ethanolamine | 5.26E-11 | 7.35E-12 | 2.45E-12 | | 7.03E-11 | 1.14E-11 | 3.79E-12 |
| ***N*-acyl GABA** |  |  |  | |  |  |  |
| *N*-palmitoyl GABA | 8.28E-12 | 1.12E-12 | 3.72E-13 | | 8.18E-12 | 1.2E-12 | 3.99E-13 |
| *N*-stearoyl GABA | 1.25E-11 | 1.28E-12 | 4.28E-13 | | 1.21E-11 | 1.02E-12 | 3.41E-13 |
| *N*-oleoyl GABA | 7.98E-12 | 9.96E-13 | 3.32E-13 | | 7.88E-12 | 8.62E-13 | 2.87E-13 |
| *N*-linoleoyl GABA | 3.13E-12 | 4.46E-13 | 1.49E-13 | | 2.96E-12 | 5.29E-13 | 1.76E-13 |
| *N*-arachidonoyl GABA | 1.6E-11 | 1.72E-12 | 5.72E-13 | | 1.73E-11 | 2.49E-12 | 8.3E-13 |
| *N*-docosahexaenoyl GABA | 4.19E-12 | 8.17E-13 | 2.72E-13 | | 4.1E-12 | 7.52E-13 | 2.51E-13 |
| ***N*-acyl glycine** |  |  |  | |  |  |  |
| *N*-palmitoyl glycine | 8.17E-12 | 9.95E-13 | 3.32E-13 | | 8.72E-12 | 1.04E-12 | 3.46E-13 |
| *N*-stearoyl glycine | 6.22E-12 | 1.18E-12 | 3.92E-13 | | 6.03E-12 | 1.59E-12 | 5.3E-13 |
| *N*-oleoyl glycine | 4.88E-12 | 4.43E-13 | 1.48E-13 | | 4.9E-12 | 9.67E-13 | 3.22E-13 |
| *N*-linoleoyl glycine | 4.94E-13 | 6.52E-14 | 2.17E-14 | | 5.92E-13 | 8.27E-14 | 2.76E-14 |
| *N*-arachidonoyl glycine | 1.54E-11 | 1.34E-12 | 4.46E-13 | | 1.91E-11 | 1.8E-12 | 5.99E-13 |
| *N*-docosahexaenoyl glycine | 1.34E-12 | 1.28E-13 | 4.26E-14 | | 1.57E-12 | 1.44E-13 | 4.8E-14 |
| ***N*-acyl leucine** |  |  |  | |  |  |  |
| *N*-palmitoyl leucine | 6.16E-13 | 4.88E-14 | 1.63E-14 | | 7.02E-13 | 5.37E-14 | 1.79E-14 |
| *N*-stearoyl leucine | 1.24E-12 | 1.16E-13 | 3.86E-14 | | 1.27E-12 | 1.85E-13 | 6.15E-14 |
| *N*-oleoyl leucine | 2.41E-13 | 2.91E-14 | 9.69E-15 | | 2.5E-13 | 4.12E-14 | 1.37E-14 |
| *N*-linoleoyl leucine | 1.14E-13 | 2.49E-14 | 8.31E-15 | | 1.11E-13 | 3.36E-14 | 1.12E-14 |
| *N*-docosahexaenoyl leucine | 4.19E-13 | 4.73E-14 | 1.58E-14 | | 4.1E-13 | 4.34E-14 | 1.45E-14 |
| ***N*-acyl methionine** |  |  |  | |  |  |  |
| *N*-palmitoyl methionine | 3.19E-12 | 8.83E-13 | 2.94E-13 | | 3.52E-12 | 5.25E-13 | 1.75E-13 |
| *N*-stearoyl methionine | 1.43E-12 | 2.94E-13 | 9.79E-14 | | 1.66E-12 | 1.79E-13 | 5.96E-14 |
| *N*-oleoyl methionine | 6.74E-13 | 8.12E-14 | 2.71E-14 | | 6.67E-13 | 1.7E-13 | 5.65E-14 |
| *N*-linoleoyl methionine | BDL |  |  | | BDL |  |  |
| *N*-arachidonoyl methionine | 2.42E-13 | 5.2E-14 | 1.73E-14 | | 3.4E-13 | 5.27E-14 | 1.76E-14 |
| *N*-docosahexaenoyl methionine | PISSR |  |  | | PISSR |  |  |
| ***N*-acyl phenylalanine** |  |  |  | |  |  |  |
| *N*-palmitoyl phenylalanine | 2.07E-12 | 5.55E-13 | 1.85E-13 | | 2.12E-12 | 5.09E-13 | 1.7E-13 |
| *N*-stearoyl phenylalanine | 2.17E-12 | 5.14E-13 | 1.71E-13 | | 2.25E-12 | 3.87E-13 | 1.29E-13 |
| *N*-oleoyl phenylalanine | 1.27E-12 | 3.78E-13 | 1.26E-13 | | 1.21E-12 | 3.58E-13 | 1.19E-13 |
| *N*-linoleoyl phenylalanine | 1.15E-13 | 5.01E-14 | 1.67E-14 | | 1.25E-13 | 3.19E-14 | 1.06E-14 |
| *N*-arachidonoyl phenylalanine | 4.47E-13 | 5.9E-14 | 1.97E-14 | | 5.85E-13 | 4.91E-14 | 1.64E-14 |
| *N*-docosahexaenoyl phenylalanine | 4.45E-13 | 1.3E-13 | 4.33E-14 | | 4.68E-13 | 1.21E-13 | 4.04E-14 |

**Supplemental Table 35: Continued**

|  | Cortex | | | | | |
| --- | --- | --- | --- | --- | --- | --- |
|  | Vehicle | | | 3mg/kg CBD | | |
| ***N*-acyl proline** | Mean | Std Dev | Std Error | Mean | Std Dev | Std Error |
| *N*-palmitoyl proline | 1.1E-13 | 3.13E-14 | 1.04E-14 | 1.77E-13 | 5.38E-14 | 1.79E-14 |
| *N*-stearoyl proline | 5.32E-14 | 1.29E-14 | 4.29E-15 | 6.32E-14 | 2.09E-14 | 6.96E-15 |
| *N-*oleoyl proline | 3.21E-14 | 1.38E-14 | 4.6E-15 | 3.38E-14 | 1.7E-14 | 5.68E-15 |
| *N*-linoleoyl proline | PISSR |  |  | PISSR |  |  |
| *N*-arachidonoyl proline | PISSR |  |  | PISSR |  |  |
| *N*-docosahexaenoyl proline | PISSR |  |  | PISSR |  |  |
| ***N*-acyl serine** |  |  |  |  |  |  |
| *N*-palmitoyl serine | 5.82E-11 | 1.54E-11 | 5.15E-12 | 6.59E-11 | 1.07E-11 | 3.56E-12 |
| *N*-stearoyl serine | 1.85E-11 | 4.16E-12 | 1.39E-12 | 1.85E-11 | 2.66E-12 | 8.85E-13 |
| *N*-oleoyl serine | 2.13E-10 | 3.76E-11 | 1.25E-11 | 2.52E-10 | 3.35E-11 | 1.12E-11 |
| *N*-linoleoyl serine | 4.97E-11 | 9.47E-12 | 3.16E-12 | 5.35E-11 | 7.94E-12 | 2.65E-12 |
| *N*-arachidonoyl serine | 3.73E-12 | 7.41E-13 | 2.47E-13 | 5.18E-12 | 6.9E-13 | 2.3E-13 |
| *N*-docosahexaenoyl serine | 1.87E-11 | 4.45E-12 | 1.48E-12 | 2.71E-11 | 5.16E-12 | 1.72E-12 |
| ***N*-acyl taurine** |  |  |  |  |  |  |
| *N*-arachidonoyl taurine | 1.13E-10 | 2.26E-11 | 7.55E-12 | 1.1E-10 | 3.08E-11 | 1.03E-11 |
| ***N*-acyl tryptophan** |  |  |  |  |  |  |
| *N*-palmitoyl tryptophan | 1.52E-12 | 4.88E-13 | 1.63E-13 | 1.65E-12 | 3.09E-13 | 1.03E-13 |
| *N*-stearoyl tryptophan | 3.69E-12 | 2.68E-13 | 8.92E-14 | 4.46E-12 | 3.31E-13 | 1.1E-13 |
| *N*-oleoyl tryptophan | 3.51E-13 | 7.09E-14 | 2.36E-14 | 5.03E-13 | 1.16E-13 | 3.87E-14 |
| *N*-linoleoyl tryptophan | BDL |  |  | PISSR |  |  |
| *N*-arachidonoyl tryptophan | PISSR |  |  | PISSR |  |  |
| *N*-docosahexaenoyl tryptophan | PISSR |  |  | PISSR |  |  |
| ***N*-acyl tyrosine** |  |  |  |  |  |  |
| *N*-palmitoyl tyrosine | 9.71E-13 | 2.01E-13 | 6.71E-14 | 1.02E-12 | 3.51E-13 | 1.17E-13 |
| *N*-stearoyl tyrosine | 1.52E-13 | 5.08E-14 | 1.69E-14 | 1.65E-13 | 8.09E-14 | 2.7E-14 |
| *N-*oleoyl tyrosine | 4.99E-13 | 1.15E-13 | 3.83E-14 | 5.56E-13 | 2.02E-13 | 6.73E-14 |
| *N*-linoleoyl tyrosine | BDL |  |  | BDL |  |  |
| *N*-arachidonoyl tyrosine | 4.06E-13 | 5.67E-14 | 1.89E-14 | 5.2E-13 | 6.03E-14 | 2.01E-14 |
| *N*-docosahexaenoyl tyrosine | 4.48E-13 | 6.23E-14 | 2.08E-14 | 5.67E-13 | 9.01E-14 | 3E-14 |
| ***N*-acyl valine** |  |  |  |  |  |  |
| *N*-palmitoyl valine | 4.12E-13 | 6.73E-14 | 2.24E-14 | 4.27E-13 | 7.86E-14 | 2.62E-14 |
| *N*- stearoyl valine | 5.29E-13 | 4.96E-14 | 1.65E-14 | 5.87E-13 | 9.35E-14 | 3.12E-14 |
| *N*-oleoyl valine | 1.52E-13 | 1.15E-14 | 3.84E-15 | 2.02E-13 | 2.9E-14 | 9.67E-15 |
| *N*-nervonoyl valine | BDL |  |  | BDL |  |  |
| *N*-linoleoyl valine | PISSR |  |  | PISSR |  |  |
| *N*-docosahexaenoyl valine | 6.83E-14 | 2.2E-14 | 7.33E-15 | 1.12E-13 | 3.93E-14 | 1.31E-14 |
| **2-acyl glycerols** |  |  |  |  |  |  |
| 2-palmitoyl glycerol | 4.55E-09 | 1.9E-09 | 6.32E-10 | 4.63E-09 | 1.51E-09 | 5.05E-10 |
| 2-oleoyl glycerol | 1.21E-08 | 6.41E-09 | 2.14E-09 | 1.22E-08 | 4.08E-09 | 1.36E-09 |
| 2-linoleoyl glycerol | 6.71E-11 | 1.9E-11 | 6.35E-12 | 6.68E-11 | 2.16E-11 | 7.2E-12 |
| 2-arachidonoyl glycerol | 7.68E-10 | 1.77E-10 | 5.92E-11 | 8.28E-10 | 1.58E-10 | 5.28E-11 |
| **Free Fatty Acids** |  |  |  |  |  |  |
| Oleic acid | 2.37E-09 | 3.62E-10 | 1.21E-10 | 2.94E-09 | 5.63E-10 | 1.88E-10 |
| Linoleic acid | 2.9E-10 | 4.7E-11 | 1.57E-11 | 3.37E-10 | 5.46E-11 | 1.82E-11 |
| Arachidonic acid | 1.92E-09 | 1.88E-10 | 6.26E-11 | 1.91E-09 | 2.09E-10 | 6.97E-11 |
| **PhosphoLEA** |  |  |  |  |  |  |
| PhosphoLEA | 1.91E-11 | 3.74E-12 | 1.25E-12 | 2.69E-11 | 2.66E-12 | 8.87E-13 |
| **Prostaglandins** |  |  |  |  |  |  |
| PGE_2_ | 4.57E-10 | 4.8E-11 | 1.6E-11 | 4.71E-10 | 7.52E-11 | 2.51E-11 |
| PGF_2α_ | 3.38E-10 | 2.03E-11 | 6.75E-12 | 2.85E-10 | 1.55E-11 | 5.17E-12 |
| 6-ketoPGF_1α_ | 4.48E-12 | 4.25E-13 | 1.42E-13 | 3.42E-12 | 5.08E-13 | 1.69E-13 |
| **THC/CBD** |  |  |  |  |  |  |
| Cannabidiol | BDL |  |  | 2.58E-11 | 8.02E-12 | 2.67E-12 |
| THC | BDL |  |  | BDL |  |  |
| **THC Metabolites** |  |  |  |  |  |  |
| 11-nor-9-carboxyTHC | BDL |  |  | BDL |  |  |
| 11-OH-THC | BDL |  |  | BDL |  |  |

**Supplemental Table 36: List of lipids in the WT cortex significantly affected by 3 mg/kg CBD**

| WT CBD Cortex Significant Differences in One-Way ANOVA | | | | |
| --- | --- | --- | --- | --- |
| Lipid | F | p | Direction (rel to Veh) | Magnitude (x Veh level) |
| *N*-oleoyl alanine | 15.43 | .001 | ↓ | 0.68 |
| *N*-linoleoyl alanine | 5.81 | .028 | ↓ | 0.67 |
| *N*-arachidonoyl alanine | 7.66 | .014 | ↑ | 1.22 |
| *N*-palmitoyl ethanolamine | 17.94 | .001 | ↑ | 1.26 |
| *N*-stearoyl ethanolamine | 31.61 | .000 | ↑ | 1.27 |
| *N*-oleoyl ethanolamine | 18.39 | .001 | ↑ | 1.54 |
| *N*-linoleoyl ethanolamine | 16.84 | .001 | ↑ | 1.44 |
| *N*-arachidonoyl ethanolamine | 14.83 | .001 | ↑ | 1.33 |
| *N*-docosahexaenoyl ethanolamine | 15.45 | .001 | ↑ | 1.34 |
| *N*-linoleoyl glycine | 7.77 | .013 | ↑ | 1.20 |
| *N*-arachidonoyl glycine | 24.68 | .000 | ↑ | 1.24 |
| *N*-docosahexaenoyl glycine | 13.26 | .002 | ↑ | 1.17 |
| *N*-palmitoyl leucine | 12.57 | .003 | ↑ | 1.14 |
| *N*-stearoyl methionine | 3.80 | .069 | ↑ | 1.16 |
| *N*-arachidonoyl methionine | 15.69 | .001 | ↑ | 1.40 |
| *N*-arachidonoyl phenylalanine | 29.12 | .000 | ↑ | 1.31 |
| *N*-palmitoyl proline | 10.32 | .005 | ↑ | 1.61 |
| *N*-oleoyl serine | 5.62 | .031 | ↑ | 1.18 |
| *N*-arachidonoyl serine | 18.60 | .001 | ↑ | 1.39 |
| *N*-docosahexaenoyl serine | 13.53 | .002 | ↑ | 1.45 |
| *N*-stearoyl tryptophan | 28.88 | .000 | ↑ | 1.21 |
| *N*-oleoyl tryptophan | 11.25 | .004 | ↑ | 1.43 |
| *N*-arachidonoyl tyrosine | 17.21 | .001 | ↑ | 1.28 |
| *N*-docosahexaenoyl tyrosine | 10.68 | .005 | ↑ | 1.27 |
| *N*-oleoyl valine | 22.65 | .000 | ↑ | 1.33 |
| *N*-docosahexaenoyl valine | 8.39 | .001 | ↑ | 1.64 |
| Oleic acid | 6.63 | .020 | ↑ | 1.24 |
| Linoleic acid | 3.89 | .066 | ↑ | 1.16 |
| phosphoLEA | 26.41 | .000 | ↑ | 1.42 |
| PGF_2α_ | 38.60 | .000 | ↓ | 0.84 |
| 6-ketoPGF_1α_ | 23.11 | .000 | ↓ | 0.76 |
| CBD | 93.37 | .000 | ↑ | infinite |

**Supplemental Table 37: Lipid levels in the hypothalamus of C57 WT female mice treated with Vehicle or 3 mg/kg CBD (CBD 9 an outlier)**

|  | Hypothalamus | | | | | | |
| --- | --- | --- | --- | --- | --- | --- | --- |
|  | Vehicle | | | 3mg/kg CBD | | | |
| ***N*-acyl alanine** | Mean | Std Dev | Std Error | | Mean | Std Dev | Std Error |
| *N*-palmitoyl alanine | 2.79E-11 | 3.11E-12 | 1.04E-12 | | 2.71E-11 | 4.09E-12 | 1.44E-12 |
| *N*-stearoyl alanine | 2.58E-11 | 6.78E-12 | 2.26E-12 | | 2.36E-11 | 5.52E-12 | 1.95E-12 |
| *N*-oleoyl alanine | 2.72E-11 | 6.33E-12 | 2.11E-12 | | 1.92E-11 | 5.2E-12 | 1.84E-12 |
| *N*-linoleoyl alanine | 1.65E-11 | 6.71E-12 | 2.24E-12 | | 1.24E-11 | 6.53E-12 | 2.31E-12 |
| *N*-arachidonoyl alanine | 1.32E-11 | 6.74E-12 | 2.25E-12 | | 1.08E-11 | 5.45E-12 | 1.93E-12 |
| *N*-docosahexaenoyl alanine | 9.47E-12 | 7.87E-12 | 2.62E-12 | | 8.02E-12 | 6.51E-12 | 2.3E-12 |
| ***N*-acyl dopamine** |  |  |  | |  |  |  |
| *N*-oleoyl dopamine | BDL |  |  | | BDL |  |  |
| *N*-arachidonoyl dopamine | BDL |  |  | | BDL |  |  |
| ***N*-acyl ethanolamine** |  |  |  | |  |  |  |
| *N*-palmitoyl ethanolamine | 8.82E-12 | 3.74E-12 | 1.25E-12 | | 8.47E-12 | 5.25E-12 | 1.86E-12 |
| *N*-stearoyl ethanolamine | 7.44E-11 | 1.06E-11 | 3.53E-12 | | 6.92E-11 | 1.08E-11 | 3.82E-12 |
| *N*-oleoyl ethanolamine | 4.51E-12 | 4.93E-12 | 1.64E-12 | | 6.21E-12 | 1.12E-11 | 3.95E-12 |
| *N*-linoleoyl ethanolamine | 2.62E-12 | 1.32E-12 | 4.38E-13 | | 2.36E-12 | 1.77E-12 | 6.27E-13 |
| *N*-arachidonoyl ethanolamine | 2.47E-12 | 1.29E-12 | 4.29E-13 | | 1.97E-12 | 1.44E-12 | 5.08E-13 |
| *N*-docosahexaenoyl ethanolamine | 4.35E-12 | 1.72E-12 | 5.73E-13 | | 3.57E-12 | 1.75E-12 | 6.19E-13 |
| ***N*-acyl GABA** |  |  |  | |  |  |  |
| *N*-palmitoyl GABA | 2.83E-11 | 4.39E-12 | 1.46E-12 | | 2.53E-11 | 5.11E-12 | 1.81E-12 |
| *N*-stearoyl GABA | 3.63E-11 | 6.38E-12 | 2.13E-12 | | 3.23E-11 | 5.39E-12 | 1.91E-12 |
| *N*-oleoyl GABA | 2.11E-11 | 7.8E-12 | 2.6E-12 | | 1.85E-11 | 6.21E-12 | 2.2E-12 |
| *N*-linoleoyl GABA | PISSR |  |  | | PISSR |  |  |
| *N*-arachidonoyl GABA | 3.31E-11 | 4.95E-12 | 1.65E-12 | | 2.87E-11 | 6.34E-12 | 2.24E-12 |
| *N*-docosahexaenoyl GABA | 9.98E-12 | 6.23E-12 | 2.08E-12 | | 9.1E-12 | 5.5E-12 | 1.94E-12 |
| ***N*-acyl glycine** |  |  |  | |  |  |  |
| *N*-palmitoyl glycine | 1.28E-11 | 5.46E-12 | 1.82E-12 | | 9.77E-12 | 1.46E-12 | 5.15E-13 |
| *N*-stearoyl glycine | 8.18E-12 | 3.56E-12 | 1.19E-12 | | 5.93E-12 | 6.39E-13 | 2.26E-13 |
| *N*-oleoyl glycine | 2.97E-12 | 5.07E-13 | 1.69E-13 | | 3.12E-12 | 6.36E-13 | 2.25E-13 |
| *N*-linoleoyl glycine | 3.67E-13 | 1.04E-13 | 3.46E-14 | | 4.15E-13 | 1.25E-13 | 4.41E-14 |
| *N*-arachidonoyl glycine | 3.22E-12 | 7.52E-13 | 2.51E-13 | | 2.38E-12 | 4.91E-13 | 1.74E-13 |
| *N*-docosahexaenoyl glycine | 7.09E-13 | 9.74E-14 | 3.25E-14 | | 9.08E-13 | 1E-13 | 3.54E-14 |
| ***N*-acyl leucine** |  |  |  | |  |  |  |
| *N*-palmitoyl leucine | 2.61E-12 | 1.87E-12 | 6.24E-13 | | 2.18E-12 | 1.35E-12 | 4.77E-13 |
| *N*-stearoyl leucine | 2.67E-12 | 1.76E-12 | 5.88E-13 | | 2.36E-12 | 1.61E-12 | 5.68E-13 |
| *N*-oleoyl leucine | 1.59E-12 | 1.33E-12 | 4.45E-13 | | 1.43E-12 | 1.55E-12 | 5.48E-13 |
| *N*-linoleoyl leucine | 2.13E-12 | 2.15E-12 | 7.17E-13 | | 1.83E-12 | 1.75E-12 | 6.18E-13 |
| *N*-docosahexaenoyl leucine | 1.91E-12 | 1.39E-12 | 4.64E-13 | | 1.73E-12 | 1.43E-12 | 5.04E-13 |
| ***N*-acyl methionine** |  |  |  | |  |  |  |
| *N*-palmitoyl methionine | 2.71E-11 | 1.07E-11 | 3.58E-12 | | 2.62E-11 | 6.7E-12 | 2.37E-12 |
| *N*-stearoyl methionine | 2.38E-12 | 2.01E-12 | 6.7E-13 | | 1.95E-12 | 1.52E-12 | 5.37E-13 |
| *N*-oleoyl methionine | 1.89E-12 | 2.46E-12 | 8.2E-13 | | 1.52E-12 | 1.73E-12 | 6.13E-13 |
| *N*-linoleoyl methionine | PISSR |  |  | | PISSR |  |  |
| *N*-arachidonoyl methionine | PISSR |  |  | | PISSR |  |  |
| *N*-docosahexaenoyl methionine | PISSR |  |  | | PISSR |  |  |
| ***N*-acyl phenylalanine** |  |  |  | |  |  |  |
| *N*-palmitoyl phenylalanine | 2.82E-12 | 1.52E-12 | 5.07E-13 | | 2.61E-12 | 1.09E-12 | 3.85E-13 |
| *N*-stearoyl phenylalanine | 2.18E-12 | 1.78E-12 | 5.93E-13 | | 2.11E-12 | 1.61E-12 | 5.69E-13 |
| *N*-oleoyl phenylalanine | 2.34E-12 | 1.53E-12 | 5.1E-13 | | 2.04E-12 | 1.45E-12 | 5.14E-13 |
| *N*-linoleoyl phenylalanine | PISSR |  |  | | PISSR |  |  |
| *N*-arachidonoyl phenylalanine | 1.28E-12 | 7.92E-13 | 2.64E-13 | | 1.12E-12 | 1.03E-12 | 3.65E-13 |
| *N*-docosahexaenoyl phenylalanine | PISSR |  |  | | PISSR |  |  |

**Supplemental Table 37: Continued**

|  | Hypothalamus | | | | | |
| --- | --- | --- | --- | --- | --- | --- |
|  | Vehicle | | | 3mg/kg CBD | | |
| ***N*-acyl proline** | Mean | Std Dev | Std Error | Mean | Std Dev | Std Error |
| *N*-palmitoyl proline | 2.76E-12 | 1.63E-12 | 5.45E-13 | 2.42E-12 | 9.79E-13 | 3.46E-13 |
| *N*-stearoyl proline | 3.67E-12 | 3.53E-12 | 1.18E-12 | 3.33E-12 | 2.25E-12 | 7.95E-13 |
| *N-*oleoyl proline | 1.91E-12 | 1.55E-12 | 5.17E-13 | 1.5E-12 | 7.5E-13 | 2.65E-13 |
| *N*-linoleoyl proline | 1.45E-12 | 1.01E-12 | 3.36E-13 | 1.28E-12 | 8.32E-13 | 2.94E-13 |
| *N*-arachidonoyl proline | 1.5E-11 | 6.65E-12 | 2.22E-12 | 1.39E-11 | 5.52E-12 | 1.95E-12 |
| *N*-docosahexaenoyl proline | PISSR |  |  | PISSR |  |  |
| ***N*-acyl serine** |  |  |  |  |  |  |
| *N*-palmitoyl serine | 3.99E-11 | 6.94E-12 | 2.31E-12 | 3.42E-11 | 3.74E-12 | 1.32E-12 |
| *N*-stearoyl serine | 1.73E-11 | 2.88E-12 | 9.6E-13 | 1.56E-11 | 3.66E-12 | 1.3E-12 |
| *N*-oleoyl serine | 6.85E-10 | 1.23E-10 | 4.09E-11 | 6.54E-10 | 1.03E-10 | 3.63E-11 |
| *N*-linoleoyl serine | 1.62E-10 | 2.46E-11 | 8.21E-12 | 1.56E-10 | 2.25E-11 | 7.97E-12 |
| *N*-arachidonoyl serine | PISSR |  |  | PISSR |  |  |
| *N*-docosahexaenoyl serine | PISSR |  |  | PISSR |  |  |
| ***N*-acyl taurine** |  |  |  |  |  |  |
| *N*-palmitoyl taurine | 6.69E-10 | 1.25E-10 | 4.18E-11 | 5.26E-10 | 1.24E-10 | 4.39E-11 |
| *N*-stearoyl taurine | 5.86E-10 | 8.37E-11 | 2.79E-11 | 4.48E-10 | 5.9E-11 | 2.09E-11 |
| *N*-oleoyl taurine | 1.02E-10 | 2.1E-11 | 6.99E-12 | 5.75E-11 | 1.44E-11 | 5.1E-12 |
| *N*-arachidonoyl taurine | 4.61E-12 | 7.66E-13 | 2.55E-13 | 3.34E-12 | 8.24E-13 | 2.91E-13 |
| ***N*-acyl tryptophan** |  |  |  |  |  |  |
| *N*-palmitoyl tryptophan | PISSR |  |  | PISSR |  |  |
| *N*-stearoyl tryptophan | PISSR |  |  | PISSR |  |  |
| *N*-oleoyl tryptophan | BDL |  |  | BDL |  |  |
| *N*-linoleoyl tryptophan | BDL |  |  | BDL |  |  |
| *N*-arachidonoyl tryptophan | BDL |  |  | BDL |  |  |
| *N*-docosahexaenoyl tryptophan | BDL |  |  | BDL |  |  |
| ***N*-acyl tyrosine** |  |  |  |  |  |  |
| *N*-palmitoyl tyrosine | 5.18E-13 | 2.08E-13 | 6.92E-14 | 6.46E-13 | 3.21E-13 | 1.13E-13 |
| *N*-stearoyl tyrosine | PISSR |  |  | PISSR |  |  |
| *N-*oleoyl tyrosine | PISSR |  |  | PISSR |  |  |
| *N*-linoleoyl tyrosine | BDL |  |  | BDL |  |  |
| *N*-arachidonoyl tyrosine | BDL |  |  | BDL |  |  |
| *N*-docosahexaenoyl tyrosine | BDL |  |  | BDL |  |  |
| ***N*-acyl valine** |  |  |  |  |  |  |
| *N*-palmitoyl valine | 6.71E-13 | 2.65E-13 | 8.85E-14 | 7.25E-13 | 2.88E-13 | 1.02E-13 |
| *N*- stearoyl valine | PISSR |  |  | PISSR |  |  |
| *N*-oleoyl valine | PISSR |  |  | PISSR |  |  |
| *N*-nervonoyl valine | BDL |  |  | BDL |  |  |
| *N*-linoleoyl valine | BDL |  |  | BDL |  |  |
| *N*-docosahexaenoyl valine | BDL |  |  | BDL |  |  |
| **2-acyl glycerols** |  |  |  |  |  |  |
| 2-palmitoyl glycerol | 4.85E-09 | 4.17E-09 | 1.39E-09 | 7.01E-09 | 7.06E-09 | 2.5E-09 |
| 2-oleoyl glycerol | 1.04E-08 | 1.08E-08 | 3.59E-09 | 1.05E-08 | 1.52E-08 | 5.38E-09 |
| 2-linoleoyl glycerol | 2.79E-11 | 1.48E-11 | 4.93E-12 | 2.37E-11 | 1.15E-11 | 4.06E-12 |
| 2-arachidonoyl glycerol | 2.56E-09 | 1.01E-09 | 3.36E-10 | 1.31E-09 | 6.92E-10 | 2.45E-10 |
| **Free Fatty Acids** |  |  |  |  |  |  |
| Oleic acid | 6.26E-10 | 1.87E-10 | 6.24E-11 | 9.58E-10 | 2.56E-10 | 9.04E-11 |
| Linoleic acid | 2.37E-10 | 7.43E-11 | 2.48E-11 | 2.43E-10 | 1.05E-10 | 3.72E-11 |
| Arachidonic acid | 2.38E-09 | 4.01E-10 | 1.34E-10 | 2.21E-09 | 3.86E-10 | 1.36E-10 |
| **PhosphoLEA** |  |  |  |  |  |  |
| PhosphoLEA | 1.21E-10 | 3.31E-11 | 1.1E-11 | 1.72E-10 | 4.35E-11 | 1.54E-11 |
| **Prostaglandins** |  |  |  |  |  |  |
| PGE_2_ | 2.25E-10 | 4.14E-11 | 1.38E-11 | 1.88E-10 | 2.74E-11 | 9.68E-12 |
| PGF_2α_ | 3.09E-10 | 4.34E-11 | 1.45E-11 | 2.14E-10 | 2.27E-11 | 8.02E-12 |
| 6-ketoPGF_1α_ | 7.03E-12 | 1.29E-12 | 4.29E-13 | 5.33E-12 | 1.86E-12 | 6.57E-13 |
| **THC/CBD** |  |  |  |  |  |  |
| Cannabidiol | BDL |  |  | 7.64E-12 | 1.22E-12 | 4.31E-13 |
| THC | BDL |  |  | BDL |  |  |
| **THC Metabolites** |  |  |  |  |  |  |
| 11-nor-9-carboxyTHC | BDL |  |  | BDL |  |  |
| 11-OH-THC | BDL |  |  | BDL |  |  |

**Supplemental Table 38: List of lipids in the WT hypothalamus significantly affected by 3mg/kg CBD**

| WT CBD Hypothalamus Significant Differences in One-Way ANOVA | | | | |
| --- | --- | --- | --- | --- |
| Lipid | F | p | Direction (relative to Veh) | Magnitude (x Veh level) |
| *N*-oleoyl alanine | 8.11 | .012 | ↓ | 0.70 |
| *N*-stearoyl glycine | 3.10 | .099 | ↓ | 0.72 |
| *N*-arachidonoyl glycine | 7.22 | .017 | ↓ | 0.74 |
| *N*-docosahexaenoyl glycine | 17.29 | .001 | ↑ | 1.28 |
| *N*-palmitoyl serine | 4.37 | .054 | ↓ | 0.86 |
| *N*-palmitoyl taurine | 5.53 | .033 | ↓ | 0.79 |
| *N*-stearoyl taurine | 14.95 | .002 | ↓ | 0.76 |
| *N*-oleoyl taurine | 24.90 | .000 | ↓ | 0.56 |
| *N*-arachidonoyl taurine | 10.79 | .005 | ↓ | 0.72 |
| 2-arachidonoyl glycerol | 8.64 | .010 | ↓ | 0.51 |
| Oleic acid | 9.51 | .008 | ↑ | 1.53 |
| phosphoLEA | 7.50 | .015 | ↑ | 1.42 |
| PGF_2α_ | 7.44 | .016 | ↑ | 1.24 |
| CBD | 355.33 | .000 | ↑ | infinite |

**Supplemental Table 39: Lipid levels in the midbrain of C57 WT female mice treated with Vehicle or 3 mg/kg CBD**

|  | Midbrain | | | | | | |
| --- | --- | --- | --- | --- | --- | --- | --- |
|  | Vehicle | | | 3mg/kg CBD | | | |
| ***N*-acyl alanine** | Mean | Std Dev | Std Error | | Mean | Std Dev | Std Error |
| *N*-palmitoyl alanine | 1.1E-11 | 1.87E-12 | 6.24E-13 | | 1.07E-11 | 2.35E-12 | 7.82E-13 |
| *N*-stearoyl alanine | 1.03E-11 | 1.16E-12 | 3.86E-13 | | 1E-11 | 1.71E-12 | 5.71E-13 |
| *N*-oleoyl alanine | 1.07E-11 | 2.37E-12 | 7.88E-13 | | 7.66E-12 | 1.23E-12 | 4.12E-13 |
| *N*-linoleoyl alanine | PISSR |  |  | | PISSR |  |  |
| *N*-arachidonoyl alanine | 4.49E-12 | 9.38E-13 | 3.13E-13 | | 5.67E-12 | 8.37E-13 | 2.79E-13 |
| *N*-docosahexaenoyl alanine | PISSR |  |  | | PISSR |  |  |
| ***N*-acyl dopamine** |  |  |  | |  |  |  |
| *N*-oleoyl dopamine | BDL |  |  | | BDL |  |  |
| *N*-arachidonoyl dopamine | BDL |  |  | | BDL |  |  |
| ***N*-acyl ethanolamine** |  |  |  | |  |  |  |
| *N*-palmitoyl ethanolamine | 3.9E-10 | 7.54E-11 | 2.67E-11 | | 4.45E-10 | 9.24E-11 | 3.08E-11 |
| *N*-stearoyl ethanolamine | 4.35E-11 | 1.34E-11 | 4.72E-12 | | 4.17E-11 | 1.23E-11 | 4.11E-12 |
| *N*-oleoyl ethanolamine | 1.28E-09 | 1.96E-10 | 6.94E-11 | | 1.7E-09 | 4E-10 | 1.33E-10 |
| *N*-linoleoyl ethanolamine | 6.59E-11 | 1.1E-11 | 3.88E-12 | | 8.1E-11 | 1.59E-11 | 5.31E-12 |
| *N*-arachidonoyl ethanolamine | 3.56E-11 | 6.51E-12 | 2.17E-12 | | 4.76E-11 | 1.05E-11 | 3.5E-12 |
| *N*-docosahexaenoyl ethanolamine | 1.07E-10 | 1.22E-11 | 4.07E-12 | | 1.59E-10 | 3.09E-11 | 1.03E-11 |
| ***N*-acyl GABA** |  |  |  | |  |  |  |
| *N*-palmitoyl GABA | 2.4E-11 | 2.92E-12 | 9.74E-13 | | 2.29E-11 | 2.9E-12 | 9.65E-13 |
| *N*-stearoyl GABA | 2.8E-11 | 4.08E-12 | 1.36E-12 | | 2.67E-11 | 2.94E-12 | 9.81E-13 |
| *N*-oleoyl GABA | 1.82E-11 | 2.48E-12 | 8.26E-13 | | 1.72E-11 | 2.22E-12 | 7.4E-13 |
| *N*-linoleoyl GABA | 4.83E-12 | 7.31E-13 | 2.44E-13 | | 6E-12 | 9.93E-13 | 3.31E-13 |
| *N*-arachidonoyl GABA | 2.75E-11 | 1.65E-12 | 5.51E-13 | | 3.03E-11 | 2.66E-12 | 8.86E-13 |
| *N*-docosahexaenoyl GABA | 7.11E-12 | 1.01E-12 | 3.36E-13 | | 6.83E-12 | 1.13E-12 | 3.76E-13 |
| ***N*-acyl glycine** |  |  |  | |  |  |  |
| *N*-palmitoyl glycine | 1.55E-11 | 2.84E-12 | 9.48E-13 | | 1.64E-11 | 3.69E-12 | 1.23E-12 |
| *N*-stearoyl glycine | 1.1E-11 | 1.84E-12 | 6.12E-13 | | 1.03E-11 | 2.75E-12 | 9.17E-13 |
| *N*-oleoyl glycine | 6.45E-12 | 1.05E-12 | 3.51E-13 | | 6.31E-12 | 1.58E-12 | 5.25E-13 |
| *N*-linoleoyl glycine | 4.51E-13 | 7.17E-14 | 2.39E-14 | | 6.18E-13 | 6.46E-14 | 2.15E-14 |
| *N*-arachidonoyl glycine | 1.14E-11 | 2.17E-12 | 7.25E-13 | | 1.32E-11 | 1.35E-12 | 4.51E-13 |
| *N*-docosahexaenoyl glycine | 1.84E-12 | 5.24E-13 | 1.75E-13 | | 2.13E-12 | 3.22E-13 | 1.07E-13 |
| ***N*-acyl leucine** |  |  |  | |  |  |  |
| *N*-palmitoyl leucine | 8.06E-13 | 8.26E-14 | 2.75E-14 | | 7.9E-13 | 7.63E-14 | 2.54E-14 |
| *N*-stearoyl leucine | 8.72E-13 | 1.06E-13 | 3.54E-14 | | 8.6E-13 | 1.24E-13 | 4.14E-14 |
| *N*-oleoyl leucine | 3.14E-13 | 5.48E-14 | 1.83E-14 | | 3.04E-13 | 5.44E-14 | 1.81E-14 |
| *N*-linoleoyl leucine | PISSR |  |  | | PISSR |  |  |
| *N*-docosahexaenoyl leucine | 4.36E-13 | 6.53E-14 | 2.18E-14 | | 4.16E-13 | 5.69E-14 | 1.9E-14 |
| ***N*-acyl methionine** |  |  |  | |  |  |  |
| *N*-palmitoyl methionine | 9.86E-12 | 2.92E-12 | 9.75E-13 | | 1.12E-11 | 3.39E-12 | 1.13E-12 |
| *N*-stearoyl methionine | 1.62E-12 | 3.57E-13 | 1.19E-13 | | 1.95E-12 | 5.88E-13 | 1.96E-13 |
| *N*-oleoyl methionine | 8.65E-13 | 3.42E-13 | 1.14E-13 | | 9.37E-13 | 4.9E-13 | 1.63E-13 |
| *N*-linoleoyl methionine | BDL |  |  | | BDL |  |  |
| *N*-arachidonoyl methionine | 4.94E-13 | 1.08E-13 | 3.61E-14 | | 8.3E-13 | 1.05E-13 | 3.5E-14 |
| *N*-docosahexaenoyl methionine | PISSR |  |  | | PISSR |  |  |
| ***N*-acyl phenylalanine** |  |  |  | |  |  |  |
| *N*-palmitoyl phenylalanine | 3.06E-12 | 3.22E-13 | 1.07E-13 | | 3.25E-12 | 9.39E-13 | 3.13E-13 |
| *N*-stearoyl phenylalanine | 2.14E-12 | 7.2E-13 | 2.4E-13 | | 1.87E-12 | 4.03E-13 | 1.34E-13 |
| *N*-oleoyl phenylalanine | 1.61E-12 | 1.46E-13 | 4.88E-14 | | 2E-12 | 1.59E-13 | 5.3E-14 |
| *N*-linoleoyl phenylalanine | PISSR |  |  | | PISSR |  |  |
| *N*-arachidonoyl phenylalanine | 6.29E-13 | 9.67E-14 | 3.22E-14 | | 1.01E-12 | 1.5E-13 | 5E-14 |
| *N*-docosahexaenoyl phenylalanine | 5.13E-13 | 1.15E-13 | 3.82E-14 | | 6.41E-13 | 1.38E-13 | 4.59E-14 |

**Supplemental Table 39: Continued**

|  | Midbrain | | | | | |
| --- | --- | --- | --- | --- | --- | --- |
|  | Vehicle | | | 3mg/kg CBD | | |
| ***N*-acyl proline** | Mean | Std Dev | Std Error | Mean | Std Dev | Std Error |
| *N*-palmitoyl proline | 1.34E-13 | 5.18E-14 | 1.73E-14 | 1.23E-13 | 2.7E-14 | 9E-15 |
| *N*-stearoyl proline | PISSR |  |  | PISSR |  |  |
| *N-*oleoyl proline | PISSR |  |  | PISSR |  |  |
| *N*-linoleoyl proline | BDL |  |  | BDL |  |  |
| *N*-arachidonoyl proline | PISSR |  |  | PISSR |  |  |
| *N*-docosahexaenoyl proline | BDL |  |  | BDL |  |  |
| ***N*-acyl serine** |  |  |  |  |  |  |
| *N*-palmitoyl serine | 7.34E-11 | 1.77E-11 | 5.89E-12 | 8.18E-11 | 1.11E-11 | 3.72E-12 |
| *N*-stearoyl serine | 1.93E-11 | 4.74E-12 | 1.58E-12 | 2.18E-11 | 3.2E-12 | 1.07E-12 |
| *N*-oleoyl serine | 4.73E-10 | 1.08E-10 | 3.6E-11 | 5.57E-10 | 3.54E-11 | 1.18E-11 |
| *N*-linoleoyl serine | 1.05E-10 | 2.13E-11 | 7.11E-12 | 1.26E-10 | 7.11E-12 | 2.37E-12 |
| *N*-arachidonoyl serine | 3.73E-12 | 5.12E-13 | 1.71E-13 | 5.73E-12 | 7.83E-13 | 2.61E-13 |
| *N*-docosahexaenoyl serine | 9.52E-12 | 4.2E-12 | 1.4E-12 | 1.41E-11 | 5.25E-12 | 1.75E-12 |
| ***N*-acyl taurine** |  |  |  |  |  |  |
| *N*-arachidonoyl taurine | 1.12E-11 | 1.02E-12 | 3.4E-13 | 1.23E-11 | 1.62E-12 | 5.4E-13 |
| ***N*-acyl tryptophan** |  |  |  |  |  |  |
| *N*-palmitoyl tryptophan | 2.3E-12 | 5.82E-13 | 1.94E-13 | 2.43E-12 | 8.73E-13 | 2.91E-13 |
| *N*-stearoyl tryptophan | 2.97E-12 | 8.04E-13 | 2.68E-13 | 3.39E-12 | 1.11E-12 | 3.7E-13 |
| *N*-oleoyl tryptophan | PISSR |  |  | PISSR |  |  |
| *N*-linoleoyl tryptophan | BDL |  |  | BDL |  |  |
| *N*-arachidonoyl tryptophan | BDL |  |  | BDL |  |  |
| *N*-docosahexaenoyl tryptophan | BDL |  |  | BDL |  |  |
| ***N*-acyl tyrosine** |  |  |  |  |  |  |
| *N*-palmitoyl tyrosine | 1.01E-12 | 1.28E-13 | 4.26E-14 | 1.23E-12 | 2.33E-13 | 7.78E-14 |
| *N*-stearoyl tyrosine | 1.65E-13 | 1.22E-13 | 4.06E-14 | 1.39E-13 | 7.05E-14 | 2.35E-14 |
| *N-*oleoyl tyrosine | 5.53E-13 | 1.9E-13 | 6.34E-14 | 6.85E-13 | 2.16E-13 | 7.19E-14 |
| *N*-linoleoyl tyrosine | PISSR |  |  | PISSR |  |  |
| *N*-arachidonoyl tyrosine | 8.32E-13 | 8.74E-14 | 2.91E-14 | 8.11E-13 | 1.22E-13 | 4.06E-14 |
| *N*-docosahexaenoyl tyrosine | 5.13E-13 | 2.31E-13 | 7.71E-14 | 5.56E-13 | 1.24E-13 | 4.12E-14 |
| ***N*-acyl valine** |  |  |  |  |  |  |
| *N*-palmitoyl valine | 5.39E-13 | 1.23E-13 | 4.09E-14 | 5.79E-13 | 1.28E-13 | 4.27E-14 |
| *N*- stearoyl valine | 5.74E-13 | 1.95E-13 | 6.51E-14 | 6.82E-13 | 1.58E-13 | 5.27E-14 |
| *N*-oleoyl valine | 2.06E-13 | 6.29E-14 | 2.1E-14 | 2.38E-13 | 6.73E-14 | 2.24E-14 |
| *N*-nervonoyl valine | BDL |  |  | BDL |  |  |
| *N*-linoleoyl valine | PISSR |  |  | PISSR |  |  |
| *N*-docosahexaenoyl valine | PISSR |  |  | PISSR |  |  |
| **2-acyl glycerols** |  |  |  |  |  |  |
| 2-palmitoyl glycerol | 1.45E-09 | 2.32E-10 | 7.74E-11 | 2.13E-09 | 5.28E-10 | 1.76E-10 |
| 2-oleoyl glycerol | 5.21E-08 | 1.25E-08 | 4.15E-09 | 6.11E-08 | 1.4E-08 | 4.67E-09 |
| 2-linoleoyl glycerol | 3.04E-10 | 8.6E-11 | 2.87E-11 | 3.88E-10 | 1.23E-10 | 4.1E-11 |
| 2-arachidonoyl glycerol | 3.34E-09 | 4.95E-10 | 1.75E-10 | 3.8E-09 | 7.12E-10 | 2.37E-10 |
| **Free Fatty Acids** |  |  |  |  |  |  |
| Oleic acid | 3.05E-09 | 5.45E-10 | 1.82E-10 | 3.94E-09 | 4.78E-10 | 1.59E-10 |
| Linoleic acid | 4.96E-10 | 5.19E-11 | 1.73E-11 | 6.08E-10 | 7.77E-11 | 2.59E-11 |
| Arachidonic acid | 3.14E-09 | 4.42E-10 | 1.47E-10 | 3.13E-09 | 4.13E-10 | 1.38E-10 |
| **PhosphoLEA** |  |  |  |  |  |  |
| PhosphoLEA | 5.32E-11 | 9.34E-12 | 3.11E-12 | 6.98E-11 | 6.77E-12 | 2.26E-12 |
| **Prostaglandins** |  |  |  |  |  |  |
| PGE_2_ | 2.51E-10 | 3.41E-11 | 1.14E-11 | 2.49E-10 | 2.65E-11 | 8.82E-12 |
| PGF_2α_ | 3.07E-10 | 2.34E-11 | 7.81E-12 | 2.45E-10 | 1.63E-11 | 5.43E-12 |
| 6-ketoPGF_1α_ | 9.04E-12 | 5.78E-13 | 1.93E-13 | 5.88E-12 | 9.13E-13 | 3.04E-13 |
| **THC/CBD** |  |  |  |  |  |  |
| Cannabidiol | BDL |  |  | 3.36E-11 | 7.94E-12 | 2.65E-12 |
| THC | BDL |  |  | BDL |  |  |
| **THC Metabolites** |  |  |  |  |  |  |
| 11-nor-9-carboxyTHC | BDL |  |  | BDL |  |  |
| 11-OH-THC | BDL |  |  | BDL |  |  |

**Supplemental Table 40: List of lipids in the WT midbrain significantly affected by 3mg/kg CBD**

| WT CBD Midbrain Significant Differences in One-Way ANOVA | | | | |
| --- | --- | --- | --- | --- |
| Lipid | F | p | Direction (rel to Veh) | Magnitude (x Veh level) |
| *N*-oleoyl alanine | 11.38 | .004 | ↓ | 0.72 |
| *N*-arachidonoyl alanine | 7.97 | .012 | ↑ | 1.26 |
| *N*-oleoyl ethanolamine | 7.28 | .017 | ↑ | 1.33 |
| *N*-linoleoyl ethanolamine | 5.04 | .040 | ↑ | 1.23 |
| *N*-arachidonoyl ethanolamine | 8.44 | .010 | ↑ | 1.34 |
| *N*-docosahexaenoyl ethanolamine | 22.08 | .000 | ↑ | 1.49 |
| *N*-linoleoyl GABA | 8.06 | .012 | ↑ | 1.24 |
| *N*-arachidonoyl GABA | 7.28 | .016 | ↑ | 1.10 |
| *N*-linoleoyl glycine | 27.08 | .000 | ↑ | 1.37 |
| *N*-arachidonoyl glycine | 4.50 | .050 | ↑ | 1.16 |
| *N*-arachidonoyl methionine | 44.64 | .000 | ↑ | 1.68 |
| *N*-oleoyl phenylalanine | 29.14 | .000 | ↑ | 1.24 |
| *N*-arachidonoyl phenylalanine | 41.22 | .000 | ↑ | 1.60 |
| *N*-docosahexaenoyl phenylalanine | 4.64 | .047 | ↑ | 1.25 |
| *N*-oleoyl serine | 4.92 | .041 | ↑ | 1.18 |
| *N*-linoleoyl serine | 7.24 | .016 | ↑ | 1.20 |
| *N*-arachidonoyl serine | 40.91 | .000 | ↑ | 1.54 |
| *N*-docosahexaenoyl serine | 4.13 | .059 | ↑ | 1.48 |
| *N*-palmitoyl tyrosine | 5.96 | .027 | ↑ | 1.22 |
| 2-palmitoyl glycerol | 12.47 | .003 | ↑ | 1.47 |
| Oleic acid | 13.48 | .002 | ↑ | 1.29 |
| Linoleic acid | 12.98 | .002 | ↑ | 1.23 |
| phosphoLEA | 18.68 | .001 | ↑ | 1.31 |
| PGF_2α_ | 41.59 | .000 | ↓ | 0.83 |
| 6-ketoPGF_1α_ | 76.70 | .000 | ↓ | 0.65 |
| CBD | 161.22 | .000 | ↑ | infinite |

**Supplemental Table 41: Lipid levels in the brainstem of C57 WT female mice treated with Vehicle or 3 mg/kg CBD**

|  | Brainstem | | | | | | |
| --- | --- | --- | --- | --- | --- | --- | --- |
|  | Vehicle | | | 3mg/kg CBD | | | |
| ***N*-acyl alanine** | Mean | Std Dev | Std Error | | Mean | Std Dev | Std Error |
| *N*-palmitoyl alanine | 1.61E-11 | 4.24E-12 | 1.41E-12 | | 1.46E-11 | 3.56E-12 | 1.19E-12 |
| *N*-stearoyl alanine | 1.59E-11 | 2.95E-12 | 9.84E-13 | | 1.52E-11 | 3.74E-12 | 1.25E-12 |
| *N*-oleoyl alanine | 1.73E-11 | 4.2E-12 | 1.4E-12 | | 1.05E-11 | 2.12E-12 | 7.06E-13 |
| *N*-linoleoyl alanine | 5.68E-12 | 1.26E-12 | 4.19E-13 | | 3.65E-12 | 1.07E-12 | 3.58E-13 |
| *N*-arachidonoyl alanine | 5.86E-12 | 1.09E-12 | 3.64E-13 | | 5.55E-12 | 9.04E-13 | 3.01E-13 |
| *N*-docosahexaenoyl alanine | 3.02E-12 | 6.35E-13 | 2.12E-13 | | 3.18E-12 | 7.7E-13 | 2.57E-13 |
| ***N*-acyl dopamine** |  |  |  | |  |  |  |
| *N*-oleoyl dopamine | BDL |  |  | | BDL |  |  |
| *N*-arachidonoyl dopamine | BDL |  |  | | BDL |  |  |
| ***N*-acyl ethanolamine** |  |  |  | |  |  |  |
| *N*-palmitoyl ethanolamine | 4.37E-10 | 7.53E-11 | 2.51E-11 | | 5.58E-10 | 8.24E-11 | 2.75E-11 |
| *N*-stearoyl ethanolamine | 5.1E-11 | 1.32E-11 | 4.4E-12 | | 5.29E-11 | 1.41E-11 | 4.72E-12 |
| *N*-oleoyl ethanolamine | 1.31E-09 | 3.66E-10 | 1.22E-10 | | 2.26E-09 | 5.19E-10 | 1.73E-10 |
| *N*-linoleoyl ethanolamine | 4.61E-11 | 4.31E-12 | 1.44E-12 | | 6.2E-11 | 1.05E-11 | 3.48E-12 |
| *N*-arachidonoyl ethanolamine | 3.21E-11 | 5.33E-12 | 1.78E-12 | | 4.32E-11 | 9.5E-12 | 3.17E-12 |
| *N*-docosahexaenoyl ethanolamine | 1.22E-10 | 1.89E-11 | 6.29E-12 | | 1.65E-10 | 3.18E-11 | 1.06E-11 |
| ***N*-acyl GABA** |  |  |  | |  |  |  |
| *N*-palmitoyl GABA | 1.77E-11 | 3.81E-12 | 1.27E-12 | | 1.75E-11 | 3.91E-12 | 1.3E-12 |
| *N*-stearoyl GABA | 2.34E-11 | 4.55E-12 | 1.52E-12 | | 2.32E-11 | 4.88E-12 | 1.63E-12 |
| *N*-oleoyl GABA | 1.83E-11 | 4.73E-12 | 1.58E-12 | | 1.7E-11 | 3.74E-12 | 1.25E-12 |
| *N*-linoleoyl GABA | 6.13E-12 | 1.41E-12 | 4.71E-13 | | 5.92E-12 | 1.71E-12 | 5.72E-13 |
| *N*-arachidonoyl GABA | 1.76E-11 | 2.99E-12 | 9.95E-13 | | 1.69E-11 | 3.14E-12 | 1.05E-12 |
| *N*-docosahexaenoyl GABA | 6.42E-12 | 1.78E-12 | 5.94E-13 | | 6.2E-12 | 1.71E-12 | 5.71E-13 |
| ***N*-acyl glycine** |  |  |  | |  |  |  |
| *N*-palmitoyl glycine | 3.25E-11 | 6.07E-12 | 2.02E-12 | | 3.25E-11 | 5.91E-12 | 1.97E-12 |
| *N*-stearoyl glycine | 2.09E-11 | 6.78E-12 | 2.26E-12 | | 1.95E-11 | 5.76E-12 | 1.92E-12 |
| *N*-oleoyl glycine | 1.68E-11 | 4.89E-12 | 1.63E-12 | | 1.63E-11 | 4.23E-12 | 1.41E-12 |
| *N*-linoleoyl glycine | 1.59E-12 | 3.42E-13 | 1.14E-13 | | 1.56E-12 | 3.68E-13 | 1.23E-13 |
| *N*-arachidonoyl glycine | 2.36E-11 | 3.33E-12 | 1.11E-12 | | 2.22E-11 | 1.96E-12 | 6.53E-13 |
| *N*-docosahexaenoyl glycine | 5.52E-12 | 1.05E-12 | 3.51E-13 | | 5.23E-12 | 9.05E-13 | 3.02E-13 |
| ***N*-acyl leucine** |  |  |  | |  |  |  |
| *N*-palmitoyl leucine | 7.02E-13 | 3.3E-14 | 1.1E-14 | | 7.85E-13 | 5.4E-14 | 1.8E-14 |
| *N*-stearoyl leucine | 8.29E-13 | 1.77E-13 | 5.91E-14 | | 8.05E-13 | 1.83E-13 | 6.11E-14 |
| *N*-oleoyl leucine | 3.7E-13 | 5.72E-14 | 1.91E-14 | | 3.71E-13 | 8.25E-14 | 2.75E-14 |
| *N*-linoleoyl leucine | 1.38E-13 | 5.57E-14 | 1.86E-14 | | 1.31E-13 | 6.63E-14 | 2.21E-14 |
| *N*-docosahexaenoyl leucine | 3.22E-13 | 7.34E-14 | 2.45E-14 | | 4E-13 | 5.66E-14 | 1.89E-14 |
| ***N*-acyl methionine** |  |  |  | |  |  |  |
| *N*-palmitoyl methionine | 9.02E-12 | 3.36E-12 | 1.12E-12 | | 8.12E-12 | 1.82E-12 | 6.06E-13 |
| *N*-stearoyl methionine | 2.58E-12 | 5.76E-13 | 1.92E-13 | | 2.75E-12 | 5.2E-13 | 1.73E-13 |
| *N*-oleoyl methionine | 1.24E-12 | 3.76E-13 | 1.25E-13 | | 1.35E-12 | 3.73E-13 | 1.24E-13 |
| *N*-linoleoyl methionine | BDL |  |  | | BDL |  |  |
| *N*-arachidonoyl methionine | 3.33E-13 | 8.34E-14 | 2.78E-14 | | 6.33E-13 | 1.14E-13 | 3.79E-14 |
| *N*-docosahexaenoyl methionine | PISSR |  |  | | PISSR |  |  |
| ***N*-acyl phenylalanine** |  |  |  | |  |  |  |
| *N*-palmitoyl phenylalanine | 2.44E-12 | 3.19E-13 | 1.06E-13 | | 2.79E-12 | 2.9E-13 | 9.65E-14 |
| *N*-stearoyl phenylalanine | 1.82E-12 | 5.76E-13 | 1.92E-13 | | 1.82E-12 | 5.8E-13 | 1.93E-13 |
| *N*-oleoyl phenylalanine | 2.05E-12 | 7.79E-13 | 2.6E-13 | | 2.13E-12 | 8.98E-13 | 2.99E-13 |
| *N*-linoleoyl phenylalanine | 2.2E-13 | 1.26E-13 | 4.18E-14 | | 2.45E-13 | 9.03E-14 | 3.01E-14 |
| *N*-arachidonoyl phenylalanine | 7.51E-13 | 1.34E-13 | 4.48E-14 | | 7.5E-13 | 1.35E-13 | 4.51E-14 |
| *N*-docosahexaenoyl phenylalanine | 6.19E-13 | 1.27E-13 | 4.24E-14 | | 6.26E-13 | 1.64E-13 | 5.46E-14 |

**Supplemental Table 41: Continued**

|  | Brainstem | | | | | |
| --- | --- | --- | --- | --- | --- | --- |
|  | Vehicle | | | 3mg/kg CBD | | |
| ***N*-acyl proline** | Mean | Std Dev | Std Error | Mean | Std Dev | Std Error |
| *N*-palmitoyl proline | 4.66E-13 | 1.09E-13 | 3.63E-14 | 4.34E-13 | 1.27E-13 | 4.23E-14 |
| *N*-stearoyl proline | 1.35E-13 | 4.2E-14 | 1.4E-14 | 1.49E-13 | 3.81E-14 | 1.27E-14 |
| *N-*oleoyl proline | 1.07E-13 | 3.95E-14 | 1.32E-14 | 1.26E-13 | 3.83E-14 | 1.28E-14 |
| *N*-linoleoyl proline | PISSR |  |  | PISSR |  |  |
| *N*-arachidonoyl proline | BDL |  |  | BDL |  |  |
| *N*-docosahexaenoyl proline | BDL |  |  | BDL |  |  |
| ***N*-acyl serine** |  |  |  |  |  |  |
| *N*-palmitoyl serine | 1.32E-10 | 2.2E-11 | 7.32E-12 | 1.47E-10 | 2.09E-11 | 6.95E-12 |
| *N*-stearoyl serine | 3.21E-11 | 7.5E-12 | 2.5E-12 | 3.4E-11 | 8.04E-12 | 2.68E-12 |
| *N*-oleoyl serine | 4.1E-10 | 4.89E-11 | 1.63E-11 | 4.2E-10 | 8.58E-11 | 2.86E-11 |
| *N*-linoleoyl serine | 8.91E-11 | 1.32E-11 | 4.39E-12 | 8.94E-11 | 1.91E-11 | 6.36E-12 |
| *N*-arachidonoyl serine | 7.62E-12 | 7.12E-13 | 2.37E-13 | 9.23E-12 | 7.74E-13 | 2.58E-13 |
| *N*-docosahexaenoyl serine | 3.25E-11 | 4.47E-12 | 1.49E-12 | 3.15E-11 | 7.38E-12 | 2.46E-12 |
| ***N*-acyl taurine** |  |  |  |  |  |  |
| *N*-arachidonoyl taurine | 1.09E-11 | 9.3E-13 | 3.1E-13 | 1.11E-11 | 1.24E-12 | 4.13E-13 |
| ***N*-acyl tryptophan** |  |  |  |  |  |  |
| *N*-palmitoyl tryptophan | PISSR |  |  | PISSR |  |  |
| *N*-stearoyl tryptophan | PISSR |  |  | PISSR |  |  |
| *N*-oleoyl tryptophan | PISSR |  |  | PISSR |  |  |
| *N*-linoleoyl tryptophan | BDL |  |  | BDL |  |  |
| *N*-arachidonoyl tryptophan | BDL |  |  | BDL |  |  |
| *N*-docosahexaenoyl tryptophan | BDL |  |  | BDL |  |  |
| ***N*-acyl tyrosine** |  |  |  |  |  |  |
| *N*-palmitoyl tyrosine | 1.15E-12 | 2.24E-13 | 7.46E-14 | 1.25E-12 | 6.5E-13 | 2.17E-13 |
| *N*-stearoyl tyrosine | PISSR |  |  | PISSR |  |  |
| *N-*oleoyl tyrosine | 4.24E-13 | 2.32E-13 | 7.72E-14 | 5.45E-13 | 2.33E-13 | 7.76E-14 |
| *N*-linoleoyl tyrosine | PISSR |  |  | PISSR |  |  |
| *N*-arachidonoyl tyrosine | 6.72E-13 | 6.51E-14 | 2.17E-14 | 7.84E-13 | 8.76E-14 | 2.92E-14 |
| *N*-docosahexaenoyl tyrosine | 6.75E-13 | 6.84E-14 | 2.28E-14 | 8.27E-13 | 1.05E-13 | 3.5E-14 |
| ***N*-acyl valine** |  |  |  |  |  |  |
| *N*-palmitoyl valine | 3.47E-13 | 4.84E-14 | 1.61E-14 | 4.11E-13 | 6.42E-14 | 2.14E-14 |
| *N*- stearoyl valine | 3.92E-13 | 1.55E-13 | 5.16E-14 | 5.04E-13 | 1.98E-13 | 6.61E-14 |
| *N*-oleoyl valine | 1.83E-13 | 7.44E-14 | 2.48E-14 | 2.03E-13 | 7.71E-14 | 2.57E-14 |
| *N*-nervonoyl valine | BDL |  |  | BDL |  |  |
| *N*-linoleoyl valine | PISSR |  |  | PISSR |  |  |
| *N*-docosahexaenoyl valine | PISSR |  |  | PISSR |  |  |
| **2-acyl glycerols** |  |  |  |  |  |  |
| 2-palmitoyl glycerol | 2.59E-10 | 9.64E-11 | 3.21E-11 | 2.6E-10 | 1.23E-10 | 4.11E-11 |
| 2-oleoyl glycerol | 1.28E-08 | 1.98E-09 | 6.6E-10 | 1.65E-08 | 3.79E-09 | 1.26E-09 |
| 2-linoleoyl glycerol | 1.14E-10 | 1.33E-11 | 4.44E-12 | 1.55E-10 | 4.07E-11 | 1.36E-11 |
| 2-arachidonoyl glycerol | 3.11E-09 | 7.05E-10 | 2.35E-10 | 3.48E-09 | 8.97E-10 | 2.99E-10 |
| **Free Fatty Acids** |  |  |  |  |  |  |
| Oleic acid | 2.28E-09 | 3.96E-10 | 1.32E-10 | 2.94E-09 | 3.89E-10 | 1.3E-10 |
| Linoleic acid | 4.56E-10 | 4.56E-11 | 1.52E-11 | 5.43E-10 | 6.31E-11 | 2.1E-11 |
| Arachidonic acid | 2.27E-09 | 3.42E-10 | 1.14E-10 | 2.34E-09 | 4.32E-10 | 1.44E-10 |
| **PhosphoLEA** |  |  |  |  |  |  |
| PhosphoLEA | 3.99E-11 | 5E-12 | 1.67E-12 | 5.43E-11 | 5.49E-12 | 1.83E-12 |
| **Prostaglandins** |  |  |  |  |  |  |
| PGE_2_ | 2.75E-10 | 4.25E-11 | 1.42E-11 | 2.4E-10 | 3.15E-11 | 1.05E-11 |
| PGF_2α_ | 2.81E-10 | 2.32E-11 | 7.75E-12 | 2.29E-10 | 2.64E-11 | 8.81E-12 |
| 6-ketoPGF_1α_ | 1.29E-11 | 2.13E-12 | 7.1E-13 | 7.87E-12 | 9.5E-13 | 3.17E-13 |
| **THC/CBD** |  |  |  |  |  |  |
| Cannabidiol | BDL |  |  | 2.46E-11 | 4.4E-12 | 1.47E-12 |
| THC | BDL |  |  | BDL |  |  |
| **THC/CBD** |  |  |  |  |  |  |
| 11-nor-9-carboxyTHC | BDL |  |  | BDL |  |  |
| 11-OH-THC | BDL |  |  | BDL |  |  |

**Supplemental Table 42: List of lipids in the WT brainstem significantly affected by 3 mg/kg CBD**

| WT CBD Brainstem Significant Differences in One-Way ANOVA | | | | |
| --- | --- | --- | --- | --- |
| Lipid | F | p | Direction (relative to Veh) | Magnitude (x Veh level) |
| *N*-oleoyl alanine | 18.66 | .001 | ↓ | 0.60 |
| *N*-linoleoyl alanine | 13.58 | .002 | ↓ | 0.64 |
| *N*-palmitoyl ethanolamine | 10.56 | .005 | ↑ | 1.28 |
| *N*-oleoyl ethanolamine | 20.18 | .000 | ↑ | 1.73 |
| *N*-linoleoyl ethanolamine | 17.61 | .001 | ↑ | 1.34 |
| *N*-arachidonoyl ethanolamine | 9.55 | .008 | ↑ | 1.35 |
| *N*-docosahexaenoyl ethanolamine | 12.33 | .003 | ↑ | 1.35 |
| *N*-palmitoyl leucine | 15.50 | .001 | ↑ | 1.12 |
| *N*-docosahexaenoyl leucine | 6.35 | .023 | ↑ | 1.24 |
| *N*-arachidonoyl methionine | 40.49 | .000 | ↑ | 1.90 |
| *N*-palmitoyl phenylalanine | 6.04 | .026 | ↑ | 1.14 |
| *N*-arachidonoyl serine | 21.07 | .000 | ↑ | 1.21 |
| *N*-arachidonoyl tyrosine | 9.46 | .007 | ↑ | 1.17 |
| *N*-docosahexaenoyl tyrosine | 13.23 | .002 | ↑ | 1.23 |
| *N*-palmitoyl valine | 5.83 | .028 | ↑ | 1.18 |
| 2-oleoyl glycerol | 6.82 | .019 | ↑ | 1.29 |
| 2-linoleoyl glycerol | 8.26 | .011 | ↑ | 1.36 |
| Oleic acid | 12.61 | .003 | ↑ | 1.29 |
| Linoleic acid | 11.39 | .004 | ↑ | 1.19 |
| phosphoLEA | 33.77 | .000 | ↑ | 1.36 |
| PGE_2_ | 4.00 | .063 | ↓ | 0.87 |
| PGF_2α_ | 19.56 | .000 | ↓ | 0.81 |
| 6-ketoPGF_1α_ | 42.38 | .000 | ↓ | 0.61 |
| CBD | 282.86 | .000 | ↑ | infinite |

**Supplemental Table 43: Lipid levels in the striatum of NAPE-PLD KO female mice treated with Vehicle or 3 mg/kg CBD**

|  | Striatum | | | | | | |
| --- | --- | --- | --- | --- | --- | --- | --- |
|  | Vehicle | | | 3mg/kg CBD | | | |
| ***N*-acyl alanine** | Mean | Std Dev | Std Error | | Mean | Std Dev | Std Error |
| *N*-palmitoyl alanine | 8.63E-12 | 1.91E-12 | 6.75E-13 | | 9.67E-12 | 1.86E-12 | 6.59E-13 |
| *N*-stearoyl alanine | 7.09E-13 | 2.47E-13 | 8.74E-14 | | 9.43E-13 | 4.14E-13 | 1.46E-13 |
| *N*-oleoyl alanine | 1.76E-12 | 5.13E-13 | 1.81E-13 | | 1.87E-12 | 2.18E-13 | 7.7E-14 |
| *N*-linoleoyl alanine | PISSR |  |  | | PISSR |  |  |
| *N*-arachidonoyl alanine | 3.11E-13 | 1.17E-13 | 4.13E-14 | | 4.69E-13 | 9.84E-14 | 3.48E-14 |
| *N*-docosahexaenoyl alanine | PISSR |  |  | | PISSR |  |  |
| ***N*-acyl dopamine** |  |  |  | |  |  |  |
| *N*-oleoyl dopamine | PISSR |  |  | | PISSR |  |  |
| *N*-arachidonoyl dopamine | PISSR |  |  | | PISSR |  |  |
| ***N*-acyl ethanolamine** |  |  |  | |  |  |  |
| *N*-palmitoyl ethanolamine | 8.64E-10 | 2.47E-10 | 8.72E-11 | | 8.24E-10 | 1.52E-10 | 5.37E-11 |
| *N*-stearoyl ethanolamine | 2.24E-10 | 9.82E-11 | 3.47E-11 | | 2.18E-10 | 1.01E-10 | 3.56E-11 |
| *N*-oleoyl ethanolamine | 6.22E-10 | 1.81E-10 | 6.41E-11 | | 6.68E-10 | 1.57E-10 | 5.56E-11 |
| *N*-linoleoyl ethanolamine | 5.69E-11 | 1.11E-11 | 3.92E-12 | | 6.2E-11 | 1.21E-11 | 4.26E-12 |
| *N*-arachidonoyl ethanolamine | 4.74E-11 | 1.19E-11 | 4.21E-12 | | 5.02E-11 | 1.03E-11 | 3.65E-12 |
| *N*-docosahexaenoyl ethanolamine | 8.1E-11 | 2.02E-11 | 7.13E-12 | | 7.9E-11 | 1.33E-11 | 4.69E-12 |
| ***N*-acyl GABA** |  |  |  | |  |  |  |
| *N*-palmitoyl GABA | 3.58E-12 | 9.34E-13 | 3.3E-13 | | 4.29E-12 | 9.37E-13 | 3.31E-13 |
| *N*-stearoyl GABA | 6.31E-12 | 1.88E-12 | 6.65E-13 | | 7.37E-12 | 1.17E-12 | 4.14E-13 |
| *N*-oleoyl GABA | 1.32E-12 | 4.96E-13 | 1.75E-13 | | 1.36E-12 | 3.69E-13 | 1.3E-13 |
| *N*-linoleoyl GABA | PISSR |  |  | | PISSR |  |  |
| *N*-arachidonoyl GABA | 6.05E-12 | 1.16E-12 | 4.12E-13 | | 7.2E-12 | 1.08E-12 | 3.81E-13 |
| *N*-docosahexaenoyl GABA | 5.85E-13 | 2.72E-13 | 9.63E-14 | | 6.03E-13 | 1.15E-13 | 4.06E-14 |
| ***N*-acyl glycine** |  |  |  | |  |  |  |
| *N*-palmitoyl glycine | 2.87E-11 | 4.14E-12 | 1.46E-12 | | 3.22E-11 | 4.95E-12 | 1.75E-12 |
| *N*-stearoyl glycine | 2.18E-11 | 6.84E-12 | 2.42E-12 | | 2.48E-11 | 5.92E-12 | 2.09E-12 |
| *N*-oleoyl glycine | 2.47E-11 | 2.58E-12 | 9.13E-13 | | 2.61E-11 | 4.54E-12 | 1.6E-12 |
| *N*-linoleoyl glycine | 2.68E-12 | 8.02E-13 | 2.83E-13 | | 3.14E-12 | 5.94E-13 | 2.1E-13 |
| *N*-arachidonoyl glycine | 4.36E-11 | 2.08E-12 | 7.34E-13 | | 4.84E-11 | 5.26E-12 | 1.86E-12 |
| *N*-docosahexaenoyl glycine | 1.7E-11 | 1.93E-12 | 6.84E-13 | | 1.75E-11 | 3.33E-12 | 1.18E-12 |
| ***N*-acyl leucine** |  |  |  | |  |  |  |
| *N*-palmitoyl leucine | 2.51E-12 | 5.94E-13 | 2.1E-13 | | 2.77E-12 | 5.04E-13 | 1.78E-13 |
| *N*-stearoyl leucine | 1.93E-12 | 4.8E-13 | 1.7E-13 | | 2.21E-12 | 4.98E-13 | 1.76E-13 |
| *N*-oleoyl leucine | 7.76E-13 | 1.6E-13 | 5.64E-14 | | 7.85E-13 | 1.53E-13 | 5.4E-14 |
| *N*-linoleoyl leucine | 5.29E-14 | 2.07E-14 | 7.3E-15 | | 5.22E-14 | 1.6E-14 | 5.65E-15 |
| *N*-docosahexaenoyl leucine | PISSR |  |  | | PISSR |  |  |
| ***N*-acyl methionine** |  |  |  | |  |  |  |
| *N*-palmitoyl methionine | 4.06E-12 | 1.03E-12 | 3.63E-13 | | 4.69E-12 | 8.84E-13 | 3.12E-13 |
| *N*-stearoyl methionine | 2.03E-12 | 8.26E-13 | 2.92E-13 | | 2.83E-12 | 6.95E-13 | 2.46E-13 |
| *N*-oleoyl methionine | 1.21E-12 | 4.88E-13 | 1.73E-13 | | 1.59E-12 | 5.29E-13 | 1.87E-13 |
| *N*-linoleoyl methionine | BDL |  |  | | BDL |  |  |
| *N*-arachidonoyl methionine | PISSR |  |  | | PISSR |  |  |
| *N*-docosahexaenoyl methionine | BDL |  |  | | BDL |  |  |
| ***N*-acyl phenylalanine** |  |  |  | |  |  |  |
| *N*-palmitoyl phenylalanine | 4.73E-12 | 6.3E-13 | 2.23E-13 | | 5.08E-12 | 7.04E-13 | 2.49E-13 |
| *N*-stearoyl phenylalanine | 3.21E-12 | 7.04E-13 | 2.49E-13 | | 3.37E-12 | 7.17E-13 | 2.54E-13 |
| *N*-oleoyl phenylalanine | 1.67E-12 | 2.23E-13 | 7.88E-14 | | 1.7E-12 | 2.72E-13 | 9.63E-14 |
| *N*-linoleoyl phenylalanine | PISSR |  |  | | PISSR |  |  |
| *N*-arachidonoyl phenylalanine | 2.19E-12 | 4.87E-13 | 1.72E-13 | | 2.52E-12 | 3.86E-13 | 1.37E-13 |
| *N*-docosahexaenoyl phenylalanine | 2.67E-12 | 7.05E-13 | 2.49E-13 | | 2.05E-12 | 2.62E-13 | 9.25E-14 |

**Supplemental Table 43: Continued**

|  | Striatum | | | | | |
| --- | --- | --- | --- | --- | --- | --- |
|  | Vehicle | | | 3mg/kg CBD | | |
| ***N*-acyl proline** | Mean | Std Dev | Std Error | Mean | Std Dev | Std Error |
| *N*-palmitoyl proline | 2.07E-12 | 6.17E-13 | 2.18E-13 | 2.29E-12 | 5.84E-13 | 2.06E-13 |
| *N*-stearoyl proline | PISSR |  |  | PISSR |  |  |
| *N-*oleoyl proline | PISSR |  |  | PISSR |  |  |
| *N*-linoleoyl proline | BDL |  |  | BDL |  |  |
| *N*-arachidonoyl proline | BDL |  |  | BDL |  |  |
| *N*-docosahexaenoyl proline | BDL |  |  | BDL |  |  |
| ***N*-acyl serine** |  |  |  |  |  |  |
| *N*-palmitoyl serine | 9.97E-11 | 8.71E-12 | 3.08E-12 | 1.06E-10 | 9.34E-12 | 3.3E-12 |
| *N*-stearoyl serine | 4.51E-11 | 5.85E-12 | 2.07E-12 | 5.17E-11 | 5.57E-12 | 1.97E-12 |
| *N*-oleoyl serine | 2.9E-10 | 2.6E-11 | 9.2E-12 | 3.01E-10 | 3.24E-11 | 1.15E-11 |
| *N*-linoleoyl serine | 1.53E-10 | 1.57E-11 | 5.53E-12 | 1.59E-10 | 1.79E-11 | 6.32E-12 |
| *N*-arachidonoyl serine | 1.96E-11 | 8.86E-12 | 3.13E-12 | 2.58E-11 | 7.29E-12 | 2.58E-12 |
| *N*-docosahexaenoyl serine | 3.47E-11 | 1.17E-11 | 4.14E-12 | 3.33E-11 | 1.09E-11 | 3.85E-12 |
| ***N*-acyl taurine** |  |  |  |  |  |  |
| *N*-arachidonoyl taurine | 3.54E-11 | 7.32E-12 | 2.59E-12 | 3.41E-11 | 7.48E-12 | 2.64E-12 |
| ***N*-acyl tryptophan** |  |  |  |  |  |  |
| *N*-palmitoyl tryptophan | 4.15E-12 | 1.06E-12 | 3.74E-13 | 4.27E-12 | 1.23E-12 | 4.37E-13 |
| *N*-stearoyl tryptophan | 2.64E-12 | 8.21E-13 | 2.9E-13 | 2.69E-12 | 7.86E-13 | 2.78E-13 |
| *N*-oleoyl tryptophan | PISSR |  |  | PISSR |  |  |
| *N*-linoleoyl tryptophan | BDL |  |  | BDL |  |  |
| *N*-arachidonoyl tryptophan | PISSR |  |  | PISSR |  |  |
| *N*-docosahexaenoyl tryptophan | BDL |  |  | BDL |  |  |
| ***N*-acyl tyrosine** |  |  |  |  |  |  |
| *N*-palmitoyl tyrosine | 6.1E-13 | 1.16E-13 | 4.09E-14 | 7.74E-13 | 2.77E-13 | 9.79E-14 |
| *N*-stearoyl tyrosine | 1.65E-13 | 6.92E-14 | 2.45E-14 | 1.64E-13 | 7.37E-14 | 2.61E-14 |
| *N-*oleoyl tyrosine | 3.75E-13 | 1.86E-13 | 6.58E-14 | 3.6E-13 | 1.44E-13 | 5.09E-14 |
| *N*-linoleoyl tyrosine | BDL |  |  | BDL |  |  |
| *N*-arachidonoyl tyrosine | PISSR |  |  | PISSR |  |  |
| *N*-docosahexaenoyl tyrosine | PISSR |  |  | PISSR |  |  |
| ***N*-acyl valine** |  |  |  |  |  |  |
| *N*-palmitoyl valine | 1.3E-12 | 4.49E-13 | 1.59E-13 | 1.06E-12 | 3.79E-13 | 1.34E-13 |
| *N*- stearoyl valine | 3.73E-13 | 1.49E-13 | 5.26E-14 | 3.02E-13 | 8.69E-14 | 3.07E-14 |
| *N*-oleoyl valine | 2.24E-13 | 1.88E-14 | 6.66E-15 | 3.18E-13 | 1.07E-13 | 3.77E-14 |
| *N*-nervonoyl valine | BDL |  |  | BDL |  |  |
| *N*-linoleoyl valine | PISSR |  |  | PISSR |  |  |
| *N*-docosahexaenoyl valine | PISSR |  |  | PISSR |  |  |
| **2-acyl glycerols** |  |  |  |  |  |  |
| 2-palmitoyl glycerol | 7.6E-08 | 4.25E-08 | 1.5E-08 | 6.22E-08 | 2.41E-08 | 8.53E-09 |
| 2-oleoyl glycerol | 3.35E-08 | 1.74E-08 | 6.17E-09 | 2.61E-08 | 1.09E-08 | 3.87E-09 |
| 2-linoleoyl glycerol | 7.56E-10 | 3.13E-10 | 1.11E-10 | 6.77E-10 | 1.69E-10 | 5.99E-11 |
| 2-arachidonoyl glycerol | 3.97E-09 | 9.64E-10 | 3.41E-10 | 3.94E-09 | 6.13E-10 | 2.17E-10 |
| **Free Fatty Acids** |  |  |  |  |  |  |
| Oleic acid | 1.65E-09 | 9.15E-11 | 3.24E-11 | 1.87E-09 | 2.4E-10 | 8.5E-11 |
| Linoleic acid | 7.91E-10 | 5.26E-11 | 1.86E-11 | 9.09E-10 | 9.21E-11 | 3.26E-11 |
| Arachidonic acid | 2.3E-09 | 9.92E-11 | 3.51E-11 | 2.59E-09 | 2.71E-10 | 9.58E-11 |
| **PhosphoLEA** |  |  |  |  |  |  |
| PhosphoLEA | 4.26E-11 | 1.35E-11 | 4.76E-12 | 4.26E-11 | 1.91E-11 | 6.74E-12 |
| **Prostaglandins** |  |  |  |  |  |  |
| PGE_2_ | 2.97E-10 | 7.19E-11 | 2.54E-11 | 2.55E-10 | 4.46E-11 | 1.58E-11 |
| PGF_2α_ | 3.72E-10 | 8.02E-11 | 2.84E-11 | 2.99E-10 | 7.13E-11 | 2.52E-11 |
| 6-ketoPGF_1α_ | PISSR |  |  | PISSR |  |  |
| **THC/CBD** |  |  |  |  |  |  |
| Cannabidiol | BDL |  |  | 4.73E-11 | 1.65E-11 | 5.83E-12 |
| THC | BDL |  |  | BDL |  |  |
| **THC Metabolites** |  |  |  |  |  |  |
| 11-nor-9-carboxyTHC | BDL |  |  | BDL |  |  |
| 11-OH-THC | BDL |  |  | BDL |  |  |

**Supplemental Table 44: List of lipids in the NAPE-PLD KO striatum significantly affected by 3mg/kg CBD**

| NAPE-PLD KO Striatum Significant Differences in One-Way ANOVA | | | | |
| --- | --- | --- | --- | --- |
| Lipid | F | p | Direction (relative to Veh) | Magnitude (x Veh level) |
| *N*-arachidonoyl alanine | 8.58 | .011 | ↑ | 1.51 |
| *N*-arachidonoyl GABA | 4.24 | .059 | ↑ | 1.19 |
| *N*-arachidonoyl glycine | 5.84 | .030 | ↑ | 1.11 |
| *N*-stearoyl methionine | 4.35 | .056 | ↑ | 1.39 |
| *N*-docosahexaenoyl phenylalanine | 5.29 | .037 | ↓ | 0.77 |
| *N*-stearoyl serine | 5.35 | .036 | ↑ | 1.15 |
| *N*-oleoyl valine | 5.96 | .029 | ↑ | 1.42 |
| Oleic acid | 5.89 | .029 | ↑ | 1.13 |
| Linoleic acid | 9.92 | .007 | ↑ | 1.15 |
| Arachidonic acid | 8.08 | .013 | ↑ | 1.13 |
| PGF_2α_ | 3.64 | .077 | ↓ | 0.81 |
| CBD | 65.80 | .000 | ↑ | infinite |

**Supplemental Table 45: Lipid levels in the hippocampus of NAPE-PLD KO female mice treated with Vehicle or 3 mg/kg CBD**

|  | Hippocampus | | | | | | |
| --- | --- | --- | --- | --- | --- | --- | --- |
|  | Vehicle | | | 3mg/kg CBD | | | |
| ***N*-acyl alanine** | Mean | Std Dev | Std Error | | Mean | Std Dev | Std Error |
| *N*-palmitoyl alanine | 1.36E-11 | 1.25E-11 | 4.4E-12 | | 1.07E-11 | 2.73E-12 | 9.66E-13 |
| *N*-stearoyl alanine | 2.41E-12 | 3.31E-12 | 1.17E-12 | | 1.18E-12 | 4.76E-13 | 1.68E-13 |
| *N*-oleoyl alanine | 2.27E-12 | 5.78E-13 | 2.04E-13 | | 2.14E-12 | 5.55E-13 | 1.96E-13 |
| *N*-linoleoyl alanine | 4.28E-13 | 1.21E-13 | 4.29E-14 | | 4.9E-13 | 1.96E-13 | 6.94E-14 |
| *N*-arachidonoyl alanine | 1.37E-12 | 2.12E-13 | 7.5E-14 | | 1.89E-12 | 5.66E-13 | 2E-13 |
| *N*-docosahexaenoyl alanine | PISSR |  |  | | PISSR |  |  |
| ***N*-acyl dopamine** |  |  |  | |  |  |  |
| *N*-oleoyl dopamine | BDL |  |  | | BDL |  |  |
| *N*-arachidonoyl dopamine | BDL |  |  | | BDL |  |  |
| ***N*-acyl ethanolamine** |  |  |  | |  |  |  |
| *N*-palmitoyl ethanolamine | 4.18E-10 | 9.49E-11 | 3.35E-11 | | 3.29E-10 | 1.04E-10 | 3.66E-11 |
| *N*-stearoyl ethanolamine | 8.94E-11 | 6.87E-11 | 2.43E-11 | | 5.38E-11 | 2.1E-11 | 7.44E-12 |
| *N*-oleoyl ethanolamine | 3.99E-10 | 1.79E-10 | 6.31E-11 | | 3.51E-10 | 1.41E-10 | 4.99E-11 |
| *N*-linoleoyl ethanolamine | 4.34E-11 | 1.52E-11 | 5.38E-12 | | 4.26E-11 | 1.32E-11 | 4.66E-12 |
| *N*-arachidonoyl ethanolamine | 5.66E-11 | 1.75E-11 | 6.18E-12 | | 5.72E-11 | 1.99E-11 | 7.04E-12 |
| *N*-docosahexaenoyl ethanolamine | 6.62E-11 | 2.91E-11 | 1.03E-11 | | 6.37E-11 | 2.64E-11 | 9.32E-12 |
| ***N*-acyl GABA** |  |  |  | |  |  |  |
| *N*-palmitoyl GABA | 6.6E-12 | 7.47E-12 | 2.64E-12 | | 4.57E-12 | 1.32E-12 | 4.66E-13 |
| *N*-stearoyl GABA | 9.59E-12 | 9.89E-12 | 3.5E-12 | | 6.8E-12 | 2.08E-12 | 7.37E-13 |
| *N*-oleoyl GABA | 2.29E-12 | 3.04E-13 | 1.07E-13 | | 2.33E-12 | 7E-13 | 2.47E-13 |
| *N*-linoleoyl GABA | 6.41E-13 | 2.87E-13 | 1.01E-13 | | 7.77E-13 | 1.84E-13 | 6.49E-14 |
| *N*-arachidonoyl GABA | 1.95E-11 | 2.88E-12 | 1.02E-12 | | 2.3E-11 | 5.42E-12 | 1.92E-12 |
| *N*-docosahexaenoyl GABA | 7.13E-13 | 2.87E-13 | 1.01E-13 | | 7.47E-13 | 3.59E-13 | 1.27E-13 |
| ***N*-acyl glycine** |  |  |  | |  |  |  |
| *N*-palmitoyl glycine | 3.17E-11 | 1.9E-11 | 6.72E-12 | | 2.69E-11 | 3.86E-12 | 1.36E-12 |
| *N*-stearoyl glycine | 2.53E-11 | 4.72E-11 | 1.67E-11 | | 1.06E-11 | 4.48E-12 | 1.58E-12 |
| *N*-oleoyl glycine | 6.98E-12 | 1.49E-12 | 5.25E-13 | | 6.46E-12 | 1.06E-12 | 3.75E-13 |
| *N*-linoleoyl glycine | 1.3E-12 | 2.54E-13 | 8.99E-14 | | 1.34E-12 | 3.57E-13 | 1.26E-13 |
| *N*-arachidonoyl glycine | 2.98E-11 | 2.65E-12 | 9.37E-13 | | 3.34E-11 | 4.88E-12 | 1.73E-12 |
| *N*-docosahexaenoyl glycine | 2.15E-12 | 7.66E-13 | 2.71E-13 | | 2.09E-12 | 3.44E-13 | 1.22E-13 |
| ***N*-acyl leucine** |  |  |  | |  |  |  |
| *N*-palmitoyl leucine | 2.72E-12 | 2.33E-12 | 8.23E-13 | | 2.03E-12 | 2.44E-13 | 8.64E-14 |
| *N*-stearoyl leucine | 1.65E-12 | 8.75E-13 | 3.09E-13 | | 1.43E-12 | 2.92E-13 | 1.03E-13 |
| *N*-oleoyl leucine | 5.41E-13 | 1.2E-13 | 4.23E-14 | | 5.53E-13 | 1.2E-13 | 4.24E-14 |
| *N*-linoleoyl leucine | 3.88E-14 | 1.54E-14 | 5.43E-15 | | 5.48E-14 | 1.49E-14 | 5.28E-15 |
| *N*-docosahexaenoyl leucine | PISSR |  |  | | PISSR |  |  |
| ***N*-acyl methionine** |  |  |  | |  |  |  |
| *N*-palmitoyl methionine | 4.09E-12 | 1.1E-12 | 3.89E-13 | | 3.86E-12 | 6.91E-13 | 2.44E-13 |
| *N*-stearoyl methionine | 1.59E-12 | 5.79E-13 | 2.05E-13 | | 1.37E-12 | 6.01E-13 | 2.12E-13 |
| *N*-oleoyl methionine | 8.65E-13 | 4.21E-13 | 1.49E-13 | | 7.51E-13 | 3.25E-13 | 1.15E-13 |
| *N*-linoleoyl methionine | BDL |  |  | | BDL |  |  |
| *N*-arachidonoyl methionine | PISSR |  |  | | PISSR |  |  |
| *N*-docosahexaenoyl methionine | PISSR |  |  | | PISSR |  |  |
| ***N*-acyl phenylalanine** |  |  |  | |  |  |  |
| *N*-palmitoyl phenylalanine | 5.36E-12 | 1.04E-12 | 3.66E-13 | | 5.13E-12 | 4.29E-13 | 1.51E-13 |
| *N*-stearoyl phenylalanine | 3E-12 | 6.84E-13 | 2.42E-13 | | 2.73E-12 | 3.56E-13 | 1.26E-13 |
| *N*-oleoyl phenylalanine | 1.62E-12 | 3.3E-13 | 1.17E-13 | | 1.63E-12 | 3.77E-13 | 1.33E-13 |
| *N*-linoleoyl phenylalanine | 7.03E-13 | 3.23E-13 | 1.14E-13 | | 6.76E-13 | 1.9E-13 | 6.73E-14 |
| *N*-arachidonoyl phenylalanine | 2.93E-12 | 5.75E-13 | 2.03E-13 | | 2.94E-12 | 4.69E-13 | 1.66E-13 |
| *N*-docosahexaenoyl phenylalanine | 2.71E-12 | 4.17E-13 | 1.47E-13 | | 2.97E-12 | 6.11E-13 | 2.16E-13 |

**Supplemental Table 45: Continued**

|  | Hippocampus | | | | | |
| --- | --- | --- | --- | --- | --- | --- |
|  | Vehicle | | | 3mg/kg CBD | | |
| ***N*-acyl proline** | Mean | Std Dev | Std Error | Mean | Std Dev | Std Error |
| *N*-palmitoyl proline | 1.26E-12 | 1.86E-12 | 6.57E-13 | 6.86E-13 | 3E-13 | 1.06E-13 |
| *N*-stearoyl proline | 1.2E-13 | 1.26E-13 | 4.44E-14 | 9.01E-14 | 4.09E-14 | 1.45E-14 |
| *N-*oleoyl proline | 2.32E-13 | 1.7E-13 | 6.03E-14 | 1.75E-13 | 6.95E-14 | 2.46E-14 |
| *N*-linoleoyl proline | BDL |  |  | BDL |  |  |
| *N*-arachidonoyl proline | PISSR |  |  | PISSR |  |  |
| *N*-docosahexaenoyl proline | PISSR |  |  | PISSR |  |  |
| ***N*-acyl serine** |  |  |  |  |  |  |
| *N*-palmitoyl serine | 6.64E-11 | 1.84E-11 | 6.5E-12 | 6.19E-11 | 6.27E-12 | 2.22E-12 |
| *N*-stearoyl serine | 2.87E-11 | 2.46E-11 | 8.69E-12 | 2.02E-11 | 4.77E-12 | 1.69E-12 |
| *N*-oleoyl serine | 5.14E-10 | 4.68E-11 | 1.65E-11 | 6.2E-10 | 1.01E-10 | 3.56E-11 |
| *N*-linoleoyl serine | 1.08E-10 | 1.56E-11 | 5.5E-12 | 1.24E-10 | 2.3E-11 | 8.12E-12 |
| *N*-arachidonoyl serine | 3.76E-12 | 5.3E-13 | 1.87E-13 | 3.53E-12 | 7.41E-13 | 2.62E-13 |
| *N*-docosahexaenoyl serine | 9.67E-12 | 1.88E-12 | 6.66E-13 | 1.06E-11 | 1.22E-12 | 4.33E-13 |
| ***N*-acyl taurine** |  |  |  |  |  |  |
| *N*-arachidonoyl taurine | 1.2E-10 | 1.1E-11 | 3.89E-12 | 1.08E-10 | 1.53E-11 | 5.41E-12 |
| ***N*-acyl tryptophan** |  |  |  |  |  |  |
| *N*-palmitoyl tryptophan | 4.68E-12 | 2.96E-12 | 1.05E-12 | 3.55E-12 | 8.22E-13 | 2.91E-13 |
| *N*-stearoyl tryptophan | 9.55E-12 | 3.96E-12 | 1.4E-12 | 9.15E-12 | 2.44E-12 | 8.62E-13 |
| *N*-oleoyl tryptophan | PISSR |  |  | PISSR |  |  |
| *N*-linoleoyl tryptophan | BDL |  |  | BDL |  |  |
| *N*-arachidonoyl tryptophan | BDL |  |  | BDL |  |  |
| *N*-docosahexaenoyl tryptophan | PISSR |  |  | PISSR |  |  |
| ***N*-acyl tyrosine** |  |  |  |  |  |  |
| *N*-palmitoyl tyrosine | 8.02E-13 | 2.35E-13 | 8.31E-14 | 7.29E-13 | 1.99E-13 | 7.03E-14 |
| *N*-stearoyl tyrosine | 1.89E-13 | 1.3E-13 | 4.61E-14 | 1.06E-13 | 4.73E-14 | 1.67E-14 |
| *N-*oleoyl tyrosine | PISSR |  |  | PISSR |  |  |
| *N*-linoleoyl tyrosine | BDL |  |  | BDL |  |  |
| *N*-arachidonoyl tyrosine | 3.31E-13 | 2.37E-13 | 8.37E-14 | 3.04E-13 | 1.16E-13 | 4.11E-14 |
| *N*-docosahexaenoyl tyrosine | PISSR |  |  | PISSR |  |  |
| ***N*-acyl valine** |  |  |  |  |  |  |
| *N*-palmitoyl valine | 2.39E-12 | 4.03E-12 | 1.43E-12 | 1.15E-12 | 4.67E-13 | 1.65E-13 |
| *N*- stearoyl valine | 1.52E-12 | 2.95E-12 | 1.04E-12 | 5.87E-13 | 2.78E-13 | 9.83E-14 |
| *N*-oleoyl valine | 5.97E-13 | 2.47E-13 | 8.73E-14 | 5.16E-13 | 2.08E-13 | 7.35E-14 |
| *N*-nervonoyl valine | BDL |  |  | BDL |  |  |
| *N*-linoleoyl valine | PISSR |  |  | PISSR |  |  |
| *N*-docosahexaenoyl valine | PISSR |  |  | PISSR |  |  |
| **2-acyl glycerols** |  |  |  |  |  |  |
| 2-palmitoyl glycerol | 3.03E-08 | 3.22E-09 | 1.14E-09 | 4.07E-08 | 5.45E-09 | 1.93E-09 |
| 2-oleoyl glycerol | 2.2E-08 | 6.58E-09 | 2.33E-09 | 1.96E-08 | 6.62E-09 | 2.34E-09 |
| 2-linoleoyl glycerol | 4.24E-10 | 4.89E-11 | 1.73E-11 | 4.86E-10 | 3.32E-11 | 1.17E-11 |
| 2-arachidonoyl glycerol | 2.3E-09 | 5.44E-10 | 1.92E-10 | 2.81E-09 | 6.25E-10 | 2.21E-10 |
| **Free Fatty Acids** |  |  |  |  |  |  |
| Oleic acid | 1.47E-09 | 3.43E-10 | 1.21E-10 | 1.7E-09 | 3.04E-10 | 1.07E-10 |
| Linoleic acid | 7.5E-10 | 8.77E-11 | 3.1E-11 | 8.12E-10 | 1.25E-10 | 4.42E-11 |
| Arachidonic acid | 2.28E-09 | 3.44E-10 | 1.22E-10 | 2.58E-09 | 4.14E-10 | 1.46E-10 |
| **PhosphoLEA** |  |  |  |  |  |  |
| PhosphoLEA | 1.51E-11 | 8.66E-12 | 3.06E-12 | 1.83E-11 | 6.33E-12 | 2.24E-12 |
| **Prostaglandins** |  |  |  |  |  |  |
| PGE_2_ | 5.45E-10 | 5.51E-11 | 1.95E-11 | 4.43E-10 | 4.8E-11 | 1.7E-11 |
| PGF_2α_ | 5.76E-10 | 3.02E-11 | 1.07E-11 | 4.42E-10 | 5.82E-11 | 2.06E-11 |
| 6-ketoPGF_1α_ | 1.22E-11 | 2.89E-12 | 1.02E-12 | 9.16E-12 | 2.25E-12 | 7.95E-13 |
| **THC/CBD** |  |  |  |  |  |  |
| Cannabidiol | BDL |  |  | 3.88E-11 | 1.23E-11 | 4.35E-12 |
| THC | BDL |  |  | BDL |  |  |
| **THC Metabolites** |  |  |  |  |  |  |
| 11-nor-9-carboxyTHC | BDL |  |  | BDL |  |  |
| 11-OH-THC | BDL |  |  | BDL |  |  |

**Supplemental Table 46: List of lipids in the NAPE-PLD KO hippocampus significantly affected by 3mg/kg CBD**

| NAPE-PLD KO Hippocampus Significant Differences in One-Way ANOVA | | | | |
| --- | --- | --- | --- | --- |
| Lipid | F | p | Direction (relative to Veh) | Magnitude (x Veh level) |
| *N*-arachidonoyl alanine | 6.05 | .028 | ↑ | 1.38 |
| *N*-palmitoyl ethanolamine | 3.20 | .095 | ↓ | 0.79 |
| *N*-arachidonoyl glycine | 3.26 | .093 | ↑ | 1.12 |
| *N*-linoleoyl leucine | 4.49 | .052 | ↑ | 1.41 |
| *N*-oleoyl serine | 7.27 | .017 | ↑ | 1.21 |
| *N*-arachidonoyl taurine | 3.32 | .090 | ↓ | 0.90 |
| 2-palmitoyl glycerol | 21.61 | .000 | ↑ | 1.34 |
| 2-linoleoyl glycerol | 8.78 | .010 | ↑ | 1.15 |
| PGE_2_ | 15.49 | .001 | ↓ | 0.81 |
| PGF_2α_ | 33.22 | .000 | ↓ | 0.77 |
| 6-ketoPGF_1α_ | 5.54 | .034 | ↓ | 0.75 |
| CBD | 79.40 | .000 | ↑ | infinite |

**Supplemental Table 47: Lipid levels in the cerebellum of NAPE-PLD KO female mice treated with Vehicle or 3 mg/kg CBD**

|  | Cerebellum | | | | | | |
| --- | --- | --- | --- | --- | --- | --- | --- |
|  | Vehicle | | | 3mg/kg CBD | | | |
| ***N*-acyl alanine** | Mean | Std Dev | Std Error | | Mean | Std Dev | Std Error |
| *N*-palmitoyl alanine | 1.57E-11 | 1.44E-12 | 5.1E-13 | | 1.54E-11 | 2.74E-12 | 9.68E-13 |
| *N*-stearoyl alanine | 1.59E-11 | 2.68E-12 | 9.48E-13 | | 1.78E-11 | 5.91E-12 | 2.09E-12 |
| *N*-oleoyl alanine | 1.22E-11 | 1.75E-12 | 6.19E-13 | | 7.36E-12 | 9.78E-13 | 3.46E-13 |
| *N*-linoleoyl alanine | 1.83E-12 | 1.98E-13 | 7E-14 | | 7.9E-13 | 1.65E-13 | 5.82E-14 |
| *N*-arachidonoyl alanine | 4.69E-12 | 6.03E-13 | 2.13E-13 | | 3.82E-12 | 9.37E-13 | 3.31E-13 |
| *N*-docosahexaenoyl alanine | 2.29E-12 | 5.76E-13 | 2.04E-13 | | 1.91E-12 | 5.34E-13 | 1.89E-13 |
| ***N*-acyl dopamine** |  |  |  | |  |  |  |
| *N*-oleoyl dopamine | BDL |  |  | | BDL |  |  |
| *N*-arachidonoyl dopamine | BDL |  |  | | BDL |  |  |
| ***N*-acyl ethanolamine** |  |  |  | |  |  |  |
| *N*-palmitoyl ethanolamine | 4.25E-10 | 9.98E-11 | 3.53E-11 | | 3.47E-10 | 6.74E-11 | 2.38E-11 |
| *N*-stearoyl ethanolamine | 1.05E-10 | 3.35E-11 | 1.18E-11 | | 8.38E-11 | 2.12E-11 | 7.49E-12 |
| *N*-oleoyl ethanolamine | 9.78E-10 | 3.21E-10 | 1.13E-10 | | 8.58E-10 | 2.12E-10 | 7.51E-11 |
| *N*-linoleoyl ethanolamine | 3.92E-11 | 1.8E-11 | 6.37E-12 | | 3.73E-11 | 1.41E-11 | 4.98E-12 |
| *N*-arachidonoyl ethanolamine | 2.52E-11 | 8.81E-12 | 3.12E-12 | | 2.33E-11 | 6.61E-12 | 2.34E-12 |
| *N*-docosahexaenoyl ethanolamine | 5.94E-11 | 2.03E-11 | 7.18E-12 | | 5.55E-11 | 1.85E-11 | 6.53E-12 |
| ***N*-acyl GABA** |  |  |  | |  |  |  |
| *N*-palmitoyl GABA | 1.42E-11 | 1.49E-12 | 5.28E-13 | | 1.47E-11 | 3.51E-12 | 1.24E-12 |
| *N*-stearoyl GABA | 1.29E-11 | 1.25E-12 | 4.4E-13 | | 1.28E-11 | 1.84E-12 | 6.51E-13 |
| *N*-oleoyl GABA | 1E-11 | 1.36E-12 | 4.81E-13 | | 9.57E-12 | 1.44E-12 | 5.1E-13 |
| *N*-linoleoyl GABA | 1.65E-12 | 4.08E-13 | 1.44E-13 | | 1.59E-12 | 2.91E-13 | 1.03E-13 |
| *N*-arachidonoyl GABA | 1.4E-11 | 1.38E-12 | 4.88E-13 | | 1.57E-11 | 1.7E-12 | 6.03E-13 |
| *N*-docosahexaenoyl GABA | 3.6E-12 | 4.09E-13 | 1.45E-13 | | 3.28E-12 | 3.65E-13 | 1.29E-13 |
| ***N*-acyl glycine** |  |  |  | |  |  |  |
| *N*-palmitoyl glycine | 2.15E-11 | 2.96E-12 | 1.05E-12 | | 2.21E-11 | 5.45E-12 | 1.93E-12 |
| *N*-stearoyl glycine | 1.03E-11 | 7.88E-13 | 2.79E-13 | | 1.08E-11 | 2.95E-12 | 1.04E-12 |
| *N*-oleoyl glycine | 1.08E-11 | 9.93E-13 | 3.51E-13 | | 1.07E-11 | 1.89E-12 | 6.67E-13 |
| *N*-linoleoyl glycine | 1.97E-12 | 3.22E-13 | 1.14E-13 | | 1.61E-12 | 2.48E-13 | 8.76E-14 |
| *N*-arachidonoyl glycine | 2.24E-11 | 2.33E-12 | 8.23E-13 | | 2.65E-11 | 4.34E-12 | 1.53E-12 |
| *N*-docosahexaenoyl glycine | 4.53E-12 | 9.42E-13 | 3.33E-13 | | 4.26E-12 | 6.34E-13 | 2.24E-13 |
| ***N*-acyl leucine** |  |  |  | |  |  |  |
| *N*-palmitoyl leucine | 3.95E-12 | 7.45E-13 | 2.63E-13 | | 3.88E-12 | 4.54E-13 | 1.61E-13 |
| *N*-stearoyl leucine | 4.51E-12 | 4.43E-13 | 1.57E-13 | | 4.44E-12 | 8.02E-13 | 2.84E-13 |
| *N*-oleoyl leucine | 3.61E-12 | 2.99E-13 | 1.06E-13 | | 2.88E-12 | 4.13E-13 | 1.46E-13 |
| *N*-linoleoyl leucine | 1.05E-12 | 1.97E-13 | 6.97E-14 | | 9.41E-13 | 1.72E-13 | 6.1E-14 |
| *N*-docosahexaenoyl leucine | 5.17E-13 | 1.33E-13 | 4.7E-14 | | 5.73E-13 | 8.1E-14 | 2.86E-14 |
| ***N*-acyl methionine** |  |  |  | |  |  |  |
| *N*-palmitoyl methionine | 3.78E-12 | 5.1E-13 | 1.8E-13 | | 2.89E-12 | 3.67E-13 | 1.3E-13 |
| *N*-stearoyl methionine | 1.89E-12 | 3.23E-13 | 1.14E-13 | | 1.51E-12 | 2.1E-13 | 7.43E-14 |
| *N*-oleoyl methionine | 1.63E-12 | 3.11E-13 | 1.1E-13 | | 1.18E-12 | 1.92E-13 | 6.79E-14 |
| *N*-linoleoyl methionine | PISSR |  |  | | PISSR |  |  |
| *N*-arachidonoyl methionine | 1.56E-12 | 3.6E-13 | 1.27E-13 | | 1.15E-12 | 1.14E-13 | 4.02E-14 |
| *N*-docosahexaenoyl methionine | PISSR |  |  | | PISSR |  |  |
| ***N*-acyl phenylalanine** |  |  |  | |  |  |  |
| *N*-palmitoyl phenylalanine | 5.71E-12 | 8.01E-13 | 2.83E-13 | | 5.17E-12 | 5.43E-13 | 1.92E-13 |
| *N*-stearoyl phenylalanine | 2.68E-12 | 4.5E-13 | 1.59E-13 | | 2.39E-12 | 3.76E-13 | 1.33E-13 |
| *N*-oleoyl phenylalanine | 3.47E-12 | 4.06E-13 | 1.44E-13 | | 2.96E-12 | 3.77E-13 | 1.33E-13 |
| *N*-linoleoyl phenylalanine | 9.4E-13 | 3.04E-13 | 1.07E-13 | | 6.69E-13 | 2.39E-13 | 8.44E-14 |
| *N*-arachidonoyl phenylalanine | 2.57E-12 | 3.77E-13 | 1.33E-13 | | 2.27E-12 | 4.35E-13 | 1.54E-13 |
| *N*-docosahexaenoyl phenylalanine | 2.55E-12 | 5.21E-13 | 1.84E-13 | | 2.34E-12 | 4.45E-13 | 1.57E-13 |

**Supplemental Table 47: Continued**

|  | Cerebellum | | | | | |
| --- | --- | --- | --- | --- | --- | --- |
|  | Vehicle | | | 3mg/kg CBD | | |
| ***N*-acyl proline** | Mean | Std Dev | Std Error | Mean | Std Dev | Std Error |
| *N*-palmitoyl proline | 2.19E-13 | 7.25E-14 | 2.56E-14 | 1.75E-13 | 4.38E-14 | 1.55E-14 |
| *N*-stearoyl proline | 3.17E-14 | 9.64E-15 | 3.41E-15 | 3.29E-14 | 1E-14 | 3.54E-15 |
| *N-*oleoyl proline | PISSR |  |  | PISSR |  |  |
| *N*-linoleoyl proline | BDL |  |  | BDL |  |  |
| *N*-arachidonoyl proline | PISSR |  |  | PISSR |  |  |
| *N*-docosahexaenoyl proline | BDL |  |  | BDL |  |  |
| ***N*-acyl serine** |  |  |  |  |  |  |
| *N*-palmitoyl serine | 1.88E-11 | 3.51E-12 | 1.24E-12 | 1.93E-11 | 3.33E-12 | 1.18E-12 |
| *N*-stearoyl serine | 3.87E-12 | 6.39E-13 | 2.26E-13 | 3.54E-12 | 7.8E-13 | 2.76E-13 |
| *N*-oleoyl serine | 2.84E-10 | 3.25E-11 | 1.15E-11 | 2.87E-10 | 3.22E-11 | 1.14E-11 |
| *N*-linoleoyl serine | 5.49E-11 | 5.46E-12 | 1.93E-12 | 5.39E-11 | 5.97E-12 | 2.11E-12 |
| *N*-arachidonoyl serine | 1.48E-12 | 3.46E-13 | 1.22E-13 | 1.16E-12 | 2.49E-13 | 8.8E-14 |
| *N*-docosahexaenoyl serine | 6.88E-12 | 1.03E-12 | 3.63E-13 | 6.79E-12 | 1.14E-12 | 4.01E-13 |
| ***N*-acyl taurine** |  |  |  |  |  |  |
| *N*-arachidonoyl taurine | 5.11E-11 | 6.31E-12 | 2.23E-12 | 4.95E-11 | 3.38E-12 | 1.2E-12 |
| ***N*-acyl tryptophan** |  |  |  |  |  |  |
| *N*-palmitoyl tryptophan | 1.71E-12 | 3.83E-13 | 1.35E-13 | 1.45E-12 | 5.33E-13 | 1.89E-13 |
| *N*-stearoyl tryptophan | 4.7E-12 | 1.22E-12 | 4.31E-13 | 4.22E-12 | 5.27E-13 | 1.86E-13 |
| *N*-oleoyl tryptophan | PISSR |  |  | PISSR |  |  |
| *N*-linoleoyl tryptophan | PISSR |  |  | PISSR |  |  |
| *N*-arachidonoyl tryptophan | PISSR |  |  | PISSR |  |  |
| *N*-docosahexaenoyl tryptophan | PISSR |  |  | PISSR |  |  |
| ***N*-acyl tyrosine** |  |  |  |  |  |  |
| *N*-palmitoyl tyrosine | 7.31E-13 | 1.06E-13 | 3.74E-14 | 6.38E-13 | 8.5E-14 | 3E-14 |
| *N*-stearoyl tyrosine | 5.88E-14 | 2.06E-14 | 7.27E-15 | 6.34E-14 | 2.32E-14 | 8.21E-15 |
| *N-*oleoyl tyrosine | 6.46E-13 | 9.68E-14 | 3.42E-14 | 6.09E-13 | 1.67E-13 | 5.9E-14 |
| *N*-linoleoyl tyrosine | PISSR |  |  | PISSR |  |  |
| *N*-arachidonoyl tyrosine | 1.85E-13 | 3.93E-14 | 1.39E-14 | 2.33E-13 | 4.84E-14 | 1.71E-14 |
| *N*-docosahexaenoyl tyrosine | 1.33E-12 | 2.47E-13 | 8.74E-14 | 1.15E-12 | 2.04E-13 | 7.21E-14 |
| ***N*-acyl valine** |  |  |  |  |  |  |
| *N*-palmitoyl valine | 4.1E-13 | 7.29E-14 | 2.58E-14 | 4.02E-13 | 5.94E-14 | 2.1E-14 |
| *N*- stearoyl valine | 2.38E-13 | 7.91E-14 | 2.8E-14 | 1.88E-13 | 9.7E-14 | 3.43E-14 |
| *N*-oleoyl valine | 2.19E-13 | 3.35E-14 | 1.18E-14 | 1.91E-13 | 5.04E-14 | 1.78E-14 |
| *N*-nervonoyl valine | BDL |  |  | BDL |  |  |
| *N*-linoleoyl valine | PISSR |  |  | PISSR |  |  |
| *N*-docosahexaenoyl valine | 2.33E-14 | 1.36E-14 | 4.81E-15 | 2.75E-14 | 9.37E-15 | 3.31E-15 |
| **2-acyl glycerols** |  |  |  |  |  |  |
| 2-palmitoyl glycerol | 3.25E-08 | 1.25E-08 | 4.42E-09 | 3.54E-08 | 1.91E-08 | 6.76E-09 |
| 2-oleoyl glycerol | 4.19E-08 | 1.79E-08 | 6.33E-09 | 3.96E-08 | 1.37E-08 | 4.83E-09 |
| 2-linoleoyl glycerol | 3.49E-10 | 1.69E-10 | 5.99E-11 | 3.49E-10 | 1.2E-10 | 4.26E-11 |
| 2-arachidonoyl glycerol | 4.82E-09 | 1.24E-09 | 4.38E-10 | 4.73E-09 | 9.54E-10 | 3.37E-10 |
| **Free Fatty Acids** |  |  |  |  |  |  |
| Oleic acid | 9.9E-08 | 9.56E-09 | 3.38E-09 | 9.86E-08 | 1.68E-08 | 5.92E-09 |
| Linoleic acid | 2.93E-09 | 3.91E-10 | 1.38E-10 | 2.62E-09 | 2.4E-10 | 8.48E-11 |
| Arachidonic acid | 2.48E-08 | 1.92E-09 | 6.8E-10 | 2.4E-08 | 3.25E-09 | 1.15E-09 |
| **PhosphoLEA** |  |  |  |  |  |  |
| PhosphoLEA | 9.05E-11 | 6.17E-12 | 2.18E-12 | 1.1E-10 | 6.83E-12 | 2.42E-12 |
| **Prostaglandins** |  |  |  |  |  |  |
| PGE_2_ | 1.71E-10 | 2.68E-11 | 9.46E-12 | 1.49E-10 | 1.76E-11 | 6.22E-12 |
| PGF_2α_ | 1.88E-10 | 2.11E-11 | 7.44E-12 | 1.52E-10 | 1.83E-11 | 6.48E-12 |
| 6-ketoPGF_1α_ | 9.55E-12 | 1.18E-12 | 4.18E-13 | 7.69E-12 | 7.59E-13 | 2.68E-13 |
| **THC/CBD** |  |  |  |  |  |  |
| Cannabidiol | BDL |  |  | 8.71E-11 | 1.43E-11 | 5.04E-12 |
| THC | BDL |  |  | BDL |  |  |
| **THC Metabolites** |  |  |  |  |  |  |
| 11-nor-9-carboxyTHC | BDL |  |  | BDL |  |  |
| 11-OH-THC | BDL |  |  | BDL |  |  |

**Supplemental Table 48: List of lipids in the NAPE-PLD KO cerebellum significantly affected by 3mg/kg CBD**

| NAPE-PLD KO Cerebellum Significant Differences in One-Way ANOVA | | | | |
| --- | --- | --- | --- | --- |
| Lipid | F | p | Direction (relative to Veh) | Magnitude (x Veh level) |
| *N*-oleoyl alanine | 47.22 | .000 | ↓ | 0.61 |
| *N*-linoleoyl alanine | 131.20 | .000 | ↓ | 0.43 |
| *N*-arachidonoyl alanine | 4.83 | .045 | ↓ | 0.81 |
| *N*-palmitoyl ethanolamine | 3.32 | .090 | ↓ | 0.82 |
| *N*-arachidonoyl GABA | 4.74 | .047 | ↑ | 1.12 |
| *N*-linoleoyl glycine | 6.31 | .025 | ↓ | 0.82 |
| *N*-arachidonoyl glycine | 5.52 | .034 | ↑ | 1.18 |
| *N*-oleoyl leucine | 16.29 | .001 | ↓ | 0.80 |
| *N*-palmitoyl methionine | 16.18 | .001 | ↓ | 0.76 |
| *N*-stearoyl methionine | 7.66 | .015 | ↓ | 0.80 |
| *N*-oleoyl methionine | 11.91 | .004 | ↓ | 0.72 |
| *N*-arachidonoyl methionine | 9.51 | .008 | ↓ | 0.74 |
| *N*-arachidonoyl serine | 4.46 | .053 | ↓ | 0.78 |
| *N*-palmitoyl tyrosine | 3.7 | .075 | ↓ | 0.87 |
| *N*-arachidonoyl tyrosine | 4.76 | .047 | ↑ | 1.26 |
| Linoleic acid | 3.57 | .080 | ↓ | 0.89 |
| phosphoLEA | 34.57 | .000 | ↑ | 1.22 |
| PGE_2_ | 3.98 | .066 | ↓ | 0.87 |
| PGF_2α_ | 13.43 | .003 | ↓ | 0.81 |
| 6-ketoPGF_1α_ | 14.03 | .002 | ↓ | 0.81 |
| CBD | 298.39 | .000 | ↑ | infinite |

**Supplemental Table 49: Lipid levels in the thalamus of NAPE-PLD KO female mice treated with Vehicle or 3 mg/kg CBD**

|  | Thalamus | | | | | | |
| --- | --- | --- | --- | --- | --- | --- | --- |
|  | Vehicle | | | 3mg/kg CBD | | | |
| ***N*-acyl alanine** | Mean | Std Dev | Std Error | | Mean | Std Dev | Std Error |
| *N*-palmitoyl alanine | 2.54E-11 | 6.87E-12 | 2.43E-12 | | 2.3E-11 | 3.84E-12 | 1.36E-12 |
| *N*-stearoyl alanine | 1.52E-11 | 2.75E-12 | 9.73E-13 | | 1.28E-11 | 1.4E-12 | 4.95E-13 |
| *N*-oleoyl alanine | 1.62E-11 | 3.13E-12 | 1.11E-12 | | 1.21E-11 | 1.24E-12 | 4.38E-13 |
| *N*-linoleoyl alanine | 2.94E-12 | 9.37E-13 | 3.31E-13 | | 2.75E-12 | 5.39E-13 | 1.91E-13 |
| *N*-arachidonoyl alanine | 6.96E-12 | 4.98E-13 | 1.76E-13 | | 5.37E-12 | 5.82E-13 | 2.06E-13 |
| *N*-docosahexaenoyl alanine | 3.35E-12 | 7.14E-13 | 2.53E-13 | | 2.16E-12 | 2.74E-13 | 9.7E-14 |
| ***N*-acyl dopamine** |  |  |  | |  |  |  |
| *N*-oleoyl dopamine | BDL |  |  | | BDL |  |  |
| *N*-arachidonoyl dopamine | BDL |  |  | | BDL |  |  |
| ***N*-acyl ethanolamine** |  |  |  | |  |  |  |
| *N*-palmitoyl ethanolamine | 3E-10 | 1.43E-10 | 5.04E-11 | | 2.89E-10 | 8.96E-11 | 3.17E-11 |
| *N*-stearoyl ethanolamine | 5.14E-11 | 3.17E-11 | 1.12E-11 | | 5.55E-11 | 2.3E-11 | 8.13E-12 |
| *N*-oleoyl ethanolamine | 6.18E-10 | 2.86E-10 | 1.01E-10 | | 7.33E-10 | 2.62E-10 | 9.25E-11 |
| *N*-linoleoyl ethanolamine | 3.12E-11 | 1.73E-11 | 6.11E-12 | | 2.9E-11 | 1.27E-11 | 4.5E-12 |
| *N*-arachidonoyl ethanolamine | 1.74E-11 | 9.89E-12 | 3.5E-12 | | 1.79E-11 | 9.07E-12 | 3.21E-12 |
| *N*-docosahexaenoyl ethanolamine | 2.53E-11 | 7.43E-12 | 2.63E-12 | | 2.04E-11 | 7.7E-12 | 2.72E-12 |
| ***N*-acyl GABA** |  |  |  | |  |  |  |
| *N*-palmitoyl GABA | 2.33E-11 | 2.54E-12 | 8.97E-13 | | 2.33E-11 | 2.63E-12 | 9.3E-13 |
| *N*-stearoyl GABA | 3.21E-11 | 4.66E-12 | 1.65E-12 | | 2.38E-11 | 3.61E-12 | 1.28E-12 |
| *N*-oleoyl GABA | 1.63E-11 | 3.26E-12 | 1.15E-12 | | 1.59E-11 | 2.92E-12 | 1.03E-12 |
| *N*-linoleoyl GABA | 2.72E-12 | 4.5E-13 | 1.59E-13 | | 2.41E-12 | 6.29E-13 | 2.22E-13 |
| *N*-arachidonoyl GABA | 2.75E-11 | 1.14E-12 | 4.04E-13 | | 3.24E-11 | 3.04E-12 | 1.08E-12 |
| *N*-docosahexaenoyl GABA | 6.95E-12 | 9.94E-13 | 3.51E-13 | | 6.2E-12 | 8.59E-13 | 3.04E-13 |
| ***N*-acyl glycine** |  |  |  | |  |  |  |
| *N*-palmitoyl glycine | 3.34E-11 | 7.16E-12 | 2.53E-12 | | 2.17E-11 | 4.8E-12 | 1.7E-12 |
| *N*-stearoyl glycine | 9.7E-12 | 2.68E-12 | 9.48E-13 | | 7.98E-12 | 1.7E-12 | 5.99E-13 |
| *N*-oleoyl glycine | 1.19E-11 | 2.45E-12 | 8.66E-13 | | 1.28E-11 | 4.02E-12 | 1.42E-12 |
| *N*-linoleoyl glycine | 1.52E-12 | 1.02E-13 | 3.59E-14 | | 1.97E-12 | 6.14E-13 | 2.17E-13 |
| *N*-arachidonoyl glycine | 2.15E-11 | 2.1E-12 | 7.44E-13 | | 2.85E-11 | 4.52E-12 | 1.6E-12 |
| *N*-docosahexaenoyl glycine | 3.21E-12 | 3.96E-13 | 1.4E-13 | | 3.8E-12 | 5.54E-13 | 1.96E-13 |
| ***N*-acyl leucine** |  |  |  | |  |  |  |
| *N*-palmitoyl leucine | 4.72E-12 | 8.88E-13 | 3.14E-13 | | 4.19E-12 | 4.99E-13 | 1.76E-13 |
| *N*-stearoyl leucine | 4.43E-12 | 6.73E-13 | 2.38E-13 | | 4.39E-12 | 6.43E-13 | 2.27E-13 |
| *N*-oleoyl leucine | 4.14E-12 | 4.77E-13 | 1.69E-13 | | 5.28E-12 | 4.35E-13 | 1.54E-13 |
| *N*-linoleoyl leucine | PISSR |  |  | | PISSR |  |  |
| *N*-docosahexaenoyl leucine | 3.86E-13 | 6.41E-14 | 2.27E-14 | | 3.32E-13 | 4.31E-14 | 1.52E-14 |
| ***N*-acyl methionine** |  |  |  | |  |  |  |
| *N*-palmitoyl methionine | 8.25E-12 | 2.17E-12 | 7.68E-13 | | 7.17E-12 | 1.85E-12 | 6.54E-13 |
| *N*-stearoyl methionine | 1.1E-12 | 1.57E-13 | 5.55E-14 | | 1.61E-12 | 2.97E-13 | 1.05E-13 |
| *N*-oleoyl methionine | 1.32E-12 | 3.26E-13 | 1.15E-13 | | 1.42E-12 | 5.77E-13 | 2.04E-13 |
| *N*-linoleoyl methionine | BDL |  |  | | BDL |  |  |
| *N*-arachidonoyl methionine | PISSR |  |  | | PISSR |  |  |
| *N*-docosahexaenoyl methionine | PISSR |  |  | | PISSR |  |  |
| ***N*-acyl phenylalanine** |  |  |  | |  |  |  |
| *N*-palmitoyl phenylalanine | 4.26E-12 | 6.4E-13 | 2.26E-13 | | 3.48E-12 | 3.16E-13 | 1.12E-13 |
| *N*-stearoyl phenylalanine | 1.38E-12 | 3.75E-13 | 1.32E-13 | | 1.27E-12 | 1.97E-13 | 6.97E-14 |
| *N*-oleoyl phenylalanine | 2.13E-12 | 3.2E-13 | 1.13E-13 | | 1.68E-12 | 3.3E-13 | 1.17E-13 |
| *N*-linoleoyl phenylalanine | 5.6E-13 | 2.01E-13 | 7.12E-14 | | 4.51E-13 | 1.67E-13 | 5.89E-14 |
| *N*-arachidonoyl phenylalanine | 1.75E-12 | 2.01E-13 | 7.09E-14 | | 2.29E-12 | 3.49E-13 | 1.23E-13 |
| *N*-docosahexaenoyl phenylalanine | 2.29E-12 | 4.09E-13 | 1.45E-13 | | 1.99E-12 | 4.88E-13 | 1.73E-13 |

**Supplemental Table 49: Continued**

|  | Thalamus | | | | | |
| --- | --- | --- | --- | --- | --- | --- |
|  | Vehicle | | | 3mg/kg CBD | | |
| ***N*-acyl proline** | Mean | Std Dev | Std Error | Mean | Std Dev | Std Error |
| *N*-palmitoyl proline | PISSR |  |  | PISSR |  |  |
| *N*-stearoyl proline | PISSR |  |  | PISSR |  |  |
| *N-*oleoyl proline | PISSR |  |  | PISSR |  |  |
| *N*-linoleoyl proline | BDL |  |  | BDL |  |  |
| *N*-arachidonoyl proline | BDL |  |  | BDL |  |  |
| *N*-docosahexaenoyl proline | BDL |  |  | BDL |  |  |
| ***N*-acyl serine** |  |  |  |  |  |  |
| *N*-palmitoyl serine | 6.91E-11 | 1.32E-11 | 4.67E-12 | 7.09E-11 | 1.08E-11 | 3.8E-12 |
| *N*-stearoyl serine | 5.28E-12 | 2.38E-12 | 8.4E-13 | 5.48E-12 | 1.42E-12 | 5.02E-13 |
| *N*-oleoyl serine | 3.58E-10 | 3.71E-11 | 1.31E-11 | 3.59E-10 | 4.1E-11 | 1.45E-11 |
| *N*-linoleoyl serine | 7.38E-11 | 1.05E-11 | 3.72E-12 | 7.63E-11 | 9.02E-12 | 3.19E-12 |
| *N*-arachidonoyl serine | 1.96E-12 | 2.51E-13 | 8.88E-14 | 2.67E-12 | 3.33E-13 | 1.18E-13 |
| *N*-docosahexaenoyl serine | 5.42E-12 | 1.47E-12 | 5.21E-13 | 6.51E-12 | 1.12E-12 | 3.97E-13 |
| ***N*-acyl taurine** |  |  |  |  |  |  |
| *N*-arachidonoyl taurine | 3.58E-11 | 3.31E-12 | 1.17E-12 | 3.36E-11 | 4.58E-12 | 1.62E-12 |
| ***N*-acyl tryptophan** |  |  |  |  |  |  |
| *N*-palmitoyl tryptophan | 1.72E-12 | 4.49E-13 | 1.59E-13 | 2.03E-12 | 2.83E-13 | 9.99E-14 |
| *N*-stearoyl tryptophan | 6.07E-12 | 1.48E-12 | 5.25E-13 | 5.65E-12 | 1.58E-12 | 5.57E-13 |
| *N*-oleoyl tryptophan | PISSR |  |  | PISSR |  |  |
| *N*-linoleoyl tryptophan | BDL |  |  | BDL |  |  |
| *N*-arachidonoyl tryptophan | PISSR |  |  | PISSR |  |  |
| *N*-docosahexaenoyl tryptophan | PISSR |  |  | PISSR |  |  |
| ***N*-acyl tyrosine** |  |  |  |  |  |  |
| *N*-palmitoyl tyrosine | 7.05E-13 | 1.06E-13 | 3.73E-14 | 7.05E-13 | 1.7E-13 | 5.99E-14 |
| *N*-stearoyl tyrosine | 9.64E-14 | 4.07E-14 | 1.44E-14 | 1.04E-13 | 1.75E-14 | 6.18E-15 |
| *N-*oleoyl tyrosine | 5.57E-13 | 1.44E-13 | 5.09E-14 | 6.19E-13 | 1.8E-13 | 6.37E-14 |
| *N*-linoleoyl tyrosine | PISSR |  |  | PISSR |  |  |
| *N*-arachidonoyl tyrosine | 1.89E-13 | 4.01E-14 | 1.42E-14 | 2.41E-13 | 8.04E-14 | 2.84E-14 |
| *N*-docosahexaenoyl tyrosine | 7.03E-13 | 2.01E-13 | 7.09E-14 | 8.09E-13 | 3.65E-13 | 1.29E-13 |
| ***N*-acyl valine** |  |  |  |  |  |  |
| *N*-palmitoyl valine | 3.99E-13 | 1.82E-13 | 6.45E-14 | 6.26E-13 | 1.14E-13 | 4.02E-14 |
| *N*- stearoyl valine | 2E-13 | 7.73E-14 | 2.73E-14 | 3.2E-13 | 4.17E-14 | 1.47E-14 |
| *N*-oleoyl valine | 2.15E-13 | 7.01E-14 | 2.48E-14 | 3.29E-13 | 7.06E-14 | 2.5E-14 |
| *N*-nervonoyl valine | BDL |  |  | BDL |  |  |
| *N*-linoleoyl valine | PISSR |  |  | PISSR |  |  |
| *N*-docosahexaenoyl valine | PISSR |  |  | PISSR |  |  |
| **2-acyl glycerols** |  |  |  |  |  |  |
| 2-palmitoyl glycerol | 3.15E-09 | 8.87E-10 | 3.14E-10 | 3.91E-09 | 1.4E-09 | 4.95E-10 |
| 2-oleoyl glycerol | 3.46E-08 | 5.84E-09 | 2.07E-09 | 4.7E-08 | 1.35E-08 | 4.76E-09 |
| 2-linoleoyl glycerol | 1.47E-10 | 3.38E-11 | 1.2E-11 | 1.8E-10 | 5.52E-11 | 1.95E-11 |
| 2-arachidonoyl glycerol | 1.88E-09 | 1.23E-10 | 4.34E-11 | 2.37E-09 | 4.14E-10 | 1.46E-10 |
| **Free Fatty Acids** |  |  |  |  |  |  |
| Oleic acid | 5.61E-08 | 9.06E-09 | 3.2E-09 | 6.38E-08 | 1.4E-08 | 4.97E-09 |
| Linoleic acid | 7.79E-10 | 7.31E-11 | 2.58E-11 | 8.69E-10 | 8.37E-11 | 2.96E-11 |
| Arachidonic acid | 6.62E-09 | 3.77E-10 | 1.33E-10 | 7.23E-09 | 6.99E-10 | 2.47E-10 |
| **PhosphoLEA** |  |  |  |  |  |  |
| PhosphoLEA | 4.2E-11 | 5.03E-12 | 1.78E-12 | 7.74E-11 | 5.97E-12 | 2.11E-12 |
| **Prostaglandins** |  |  |  |  |  |  |
| PGE_2_ | 2.85E-10 | 3.48E-11 | 1.23E-11 | 2.3E-10 | 3E-11 | 1.06E-11 |
| PGF_2α_ | 2.74E-10 | 2.68E-11 | 9.47E-12 | 2.07E-10 | 3.31E-11 | 1.17E-11 |
| 6-ketoPGF_1α_ | 7.55E-12 | 1.25E-12 | 4.41E-13 | 4.68E-12 | 1.35E-12 | 4.77E-13 |
| **THC/CBD** |  |  |  |  |  |  |
| Cannabidiol | BDL |  |  | 6.2E-11 | 1.18E-11 | 4.18E-12 |
| THC | BDL |  |  | BDL |  |  |
| **THC Metabolites** |  |  |  |  |  |  |
| 11-nor-9-carboxyTHC | BDL |  |  | BDL |  |  |
| 11-OH-THC | BDL |  |  | BDL |  |  |

**Supplemental Table 50: List of lipids in the NAPE-PLD KO thalamus significantly affected by 3mg/kg CBD**

| NAPE-PLD KO Thalamus Significant Differences in One-Way ANOVA | | | | |
| --- | --- | --- | --- | --- |
| Lipid | F | p | Direction (relative to Veh) | Magnitude (x Veh level) |
| *N*-stearoyl alanine | 4.03 | .065 | ↓ | 0.85 |
| *N*-oleoyl alanine | 11.54 | .004 | ↓ | 0.75 |
| *N*-arachidonoyl alanine | 34.50 | .000 | ↓ | 0.77 |
| *N*-docosahexaenoyl alanine | 19.16 | .001 | ↓ | 0.65 |
| *N*-stearoyl GABA | 15.72 | .001 | ↓ | 0.74 |
| *N*-arachidonoyl GABA | 18.15 | .001 | ↑ | 1.18 |
| *N*-palmitoyl glycine | 14.86 | .002 | ↓ | 0.65 |
| *N*-linoleoyl glycine | 4.25 | .058 | ↑ | 1.30 |
| *N*-arachidonoyl glycine | 15.69 | .001 | ↑ | 1.33 |
| *N*-docosahexaenoyl glycine | 5.92 | .029 | ↑ | 1.18 |
| *N*-oleoyl leucine | 24.94 | .000 | ↑ | 1.28 |
| *N*-docosahexaenoyl leucine | 3.91 | .068 | ↓ | 0.86 |
| *N*-stearoyl methionine | 18.45 | .001 | ↑ | 1.46 |
| *N*-palmitoyl phenylalanine | 9.55 | .008 | ↓ | 0.82 |
| *N*-oleoyl phenylalanine | 7.63 | .015 | ↓ | 0.79 |
| *N*-arachidonoyl phenylalanine | 14.75 | .002 | ↑ | 1.31 |
| *N*-arachidonoyl serine | 22.95 | .000 | ↑ | 1.36 |
| *N*-palmitoyl valine | 8.93 | .010 | ↑ | 1.57 |
| *N*-stearoyl valine | 14.79 | .002 | ↑ | 1.60 |
| *N*-oleoyl valine | 10.49 | .006 | ↑ | 1.53 |
| 2-oleoyl glycerol | 5.73 | .031 | ↑ | 1.36 |
| 2-arachidonoyl glycerol | 10.32 | .006 | ↑ | 1.27 |
| Linoleic acid | 5.26 | .038 | ↑ | 1.12 |
| Arachidonic acid | 4.74 | .047 | ↑ | 1.09 |
| phosphoLEA | 164.85 | .000 | ↑ | 1.84 |
| PGE_2_ | 11.49 | .004 | ↓ | 0.81 |
| PGF_2α_ | 19.91 | .001 | ↓ | 0.76 |
| 6-ketoPGF_1α_ | 19.61 | .001 | ↓ | 0.62 |
| CBD | 219.76 | .000 | ↑ | infinite |

**Supplemental Table 51: Lipid levels in the cortex of NAPE-PLD KO female mice treated with Vehicle or 3 mg/kg CBD**

|  | Cortex | | | | | | |
| --- | --- | --- | --- | --- | --- | --- | --- |
|  | Vehicle | | | 3mg/kg CBD | | | |
| ***N*-acyl alanine** | Mean | Std Dev | Std Error | | Mean | Std Dev | Std Error |
| *N*-palmitoyl alanine | 9.41E-12 | 1.73E-12 | 6.1E-13 | | 8.78E-12 | 1.96E-12 | 6.94E-13 |
| *N*-stearoyl alanine | 8.32E-12 | 1.46E-12 | 5.18E-13 | | 8.46E-12 | 1.5E-12 | 5.32E-13 |
| *N*-oleoyl alanine | 6.84E-12 | 1.68E-12 | 5.92E-13 | | 4.41E-12 | 5.88E-13 | 2.08E-13 |
| *N*-linoleoyl alanine | 1.3E-12 | 5.71E-13 | 2.02E-13 | | 7.78E-13 | 1.78E-13 | 6.3E-14 |
| *N*-arachidonoyl alanine | 4.18E-12 | 9.23E-13 | 3.26E-13 | | 3.72E-12 | 6.34E-13 | 2.24E-13 |
| *N*-docosahexaenoyl alanine | 1.31E-12 | 3.3E-13 | 1.17E-13 | | 1.12E-12 | 3.13E-13 | 1.11E-13 |
| ***N*-acyl dopamine** |  |  |  | |  |  |  |
| *N*-oleoyl dopamine | BDL |  |  | | BDL |  |  |
| *N*-arachidonoyl dopamine | BDL |  |  | | BDL |  |  |
| ***N*-acyl ethanolamine** |  |  |  | |  |  |  |
| *N*-palmitoyl ethanolamine | 1.7E-10 | 7.62E-11 | 2.7E-11 | | 1.52E-10 | 7.63E-11 | 2.7E-11 |
| *N*-stearoyl ethanolamine | 3.46E-11 | 1.57E-11 | 5.56E-12 | | 4.15E-11 | 1.63E-11 | 5.76E-12 |
| *N*-oleoyl ethanolamine | 3.19E-10 | 1.34E-10 | 4.75E-11 | | 3.25E-10 | 1.75E-10 | 6.18E-11 |
| *N*-linoleoyl ethanolamine | 2.45E-11 | 1.72E-11 | 6.09E-12 | | 2.16E-11 | 1.34E-11 | 4.73E-12 |
| *N*-arachidonoyl ethanolamine | 1.9E-11 | 9.26E-12 | 3.27E-12 | | 1.95E-11 | 1.02E-11 | 3.61E-12 |
| *N*-docosahexaenoyl ethanolamine | 2.39E-11 | 1.21E-11 | 4.27E-12 | | 2.37E-11 | 1.61E-11 | 5.71E-12 |
| ***N*-acyl GABA** |  |  |  | |  |  |  |
| *N*-palmitoyl GABA | 8.81E-12 | 1.95E-12 | 6.89E-13 | | 8.7E-12 | 1.67E-12 | 5.91E-13 |
| *N*-stearoyl GABA | 1.23E-11 | 2.98E-12 | 1.05E-12 | | 1.22E-11 | 2.82E-12 | 9.95E-13 |
| *N*-oleoyl GABA | 6.06E-12 | 1.03E-12 | 3.65E-13 | | 6.29E-12 | 1.82E-12 | 6.42E-13 |
| *N*-linoleoyl GABA | 1.49E-12 | 2.14E-13 | 7.58E-14 | | 1.31E-12 | 5.08E-13 | 1.8E-13 |
| *N*-arachidonoyl GABA | 2.52E-11 | 3.99E-12 | 1.41E-12 | | 2.36E-11 | 5.46E-12 | 1.93E-12 |
| *N*-docosahexaenoyl GABA | 3.8E-12 | 9.53E-13 | 3.37E-13 | | 3.8E-12 | 1.12E-12 | 3.95E-13 |
| ***N*-acyl glycine** |  |  |  | |  |  |  |
| *N*-palmitoyl glycine | 1.32E-11 | 1.07E-12 | 3.79E-13 | | 1.29E-11 | 7.78E-13 | 2.75E-13 |
| *N*-stearoyl glycine | 6.33E-12 | 3.26E-12 | 1.15E-12 | | 5.3E-12 | 1.97E-12 | 6.95E-13 |
| *N*-oleoyl glycine | 7.62E-12 | 1.57E-12 | 5.56E-13 | | 7.17E-12 | 1.85E-12 | 6.53E-13 |
| *N*-linoleoyl glycine | 1.46E-12 | 3.89E-13 | 1.37E-13 | | 1.21E-12 | 1.71E-13 | 6.03E-14 |
| *N*-arachidonoyl glycine | 4.2E-11 | 9.46E-12 | 3.34E-12 | | 3.73E-11 | 5.69E-12 | 2.01E-12 |
| *N*-docosahexaenoyl glycine | 3.38E-12 | 8.71E-13 | 3.08E-13 | | 2.97E-12 | 4.31E-13 | 1.52E-13 |
| ***N*-acyl leucine** |  |  |  | |  |  |  |
| *N*-palmitoyl leucine | 2.21E-12 | 3.44E-13 | 1.22E-13 | | 2.13E-12 | 1.84E-13 | 6.5E-14 |
| *N*-stearoyl leucine | 3.15E-12 | 3.15E-13 | 1.11E-13 | | 3.24E-12 | 8.04E-13 | 2.84E-13 |
| *N*-oleoyl leucine | 1.9E-12 | 1.68E-13 | 5.93E-14 | | 1.72E-12 | 2.49E-13 | 8.8E-14 |
| *N*-linoleoyl leucine | 4.96E-13 | 1.27E-13 | 4.47E-14 | | 4.07E-13 | 1.27E-13 | 4.49E-14 |
| *N*-docosahexaenoyl leucine | 3.74E-13 | 1.07E-13 | 3.78E-14 | | 3.62E-13 | 8.12E-14 | 2.87E-14 |
| ***N*-acyl methionine** |  |  |  | |  |  |  |
| *N*-palmitoyl methionine | 4.49E-12 | 1.11E-12 | 3.93E-13 | | 4.08E-12 | 9.49E-13 | 3.35E-13 |
| *N*-stearoyl methionine | 1.35E-12 | 3.33E-13 | 1.18E-13 | | 1.33E-12 | 2.46E-13 | 8.68E-14 |
| *N*-oleoyl methionine | 1.09E-12 | 2.68E-13 | 9.48E-14 | | 9.57E-13 | 2.45E-13 | 8.68E-14 |
| *N*-linoleoyl methionine | PISSR |  |  | | PISSR |  |  |
| *N*-arachidonoyl methionine | 8.38E-13 | 2.24E-13 | 7.93E-14 | | 4.08E-13 | 1.44E-13 | 5.08E-14 |
| *N*-docosahexaenoyl methionine | PISSR |  |  | | PISSR |  |  |
| ***N*-acyl phenylalanine** |  |  |  | |  |  |  |
| *N*-palmitoyl phenylalanine | 2.2E-12 | 1.38E-13 | 4.86E-14 | | 2.03E-12 | 2.46E-13 | 8.69E-14 |
| *N*-stearoyl phenylalanine | 1.22E-12 | 1.18E-13 | 4.17E-14 | | 1.14E-12 | 2.28E-13 | 8.08E-14 |
| *N*-oleoyl phenylalanine | 1.03E-12 | 7.67E-14 | 2.71E-14 | | 9.51E-13 | 1.31E-13 | 4.63E-14 |
| *N*-linoleoyl phenylalanine | 2.82E-13 | 1.01E-13 | 3.55E-14 | | 2.53E-13 | 6.69E-14 | 2.36E-14 |
| *N*-arachidonoyl phenylalanine | 1.34E-12 | 2.17E-13 | 7.66E-14 | | 1.1E-12 | 2.08E-13 | 7.37E-14 |
| *N*-docosahexaenoyl phenylalanine | 1.44E-12 | 1.13E-13 | 4E-14 | | 1.32E-12 | 2.41E-13 | 8.51E-14 |

**Supplemental Table 51: Continued**

|  | Cortex | | | | | |
| --- | --- | --- | --- | --- | --- | --- |
|  | Vehicle | | | 3mg/kg CBD | | |
| ***N*-acyl proline** | Mean | Std Dev | Std Error | Mean | Std Dev | Std Error |
| *N*-palmitoyl proline | 3.14E-13 | 1.26E-13 | 4.47E-14 | 3.3E-13 | 7.91E-14 | 2.8E-14 |
| *N*-stearoyl proline | PISSR |  |  | PISSR |  |  |
| *N-*oleoyl proline | PISSR |  |  | PISSR |  |  |
| *N*-linoleoyl proline | BDL |  |  | BDL |  |  |
| *N*-arachidonoyl proline | PISSR |  |  | PISSR |  |  |
| *N*-docosahexaenoyl proline | 1.66E-13 | 4.77E-14 | 1.69E-14 | 1.86E-13 | 1.05E-13 | 3.72E-14 |
| ***N*-acyl serine** |  |  |  |  |  |  |
| *N*-palmitoyl serine | 1.4E-11 | 2.23E-12 | 7.88E-13 | 1.3E-11 | 3.44E-12 | 1.22E-12 |
| *N*-stearoyl serine | 2.21E-12 | 3.75E-13 | 1.32E-13 | 2.06E-12 | 4.15E-13 | 1.47E-13 |
| *N*-oleoyl serine | 1.67E-10 | 1.12E-11 | 3.97E-12 | 1.51E-10 | 1.45E-11 | 5.14E-12 |
| *N*-linoleoyl serine | 3.33E-11 | 2.35E-12 | 8.3E-13 | 3.18E-11 | 3.16E-12 | 1.12E-12 |
| *N*-arachidonoyl serine | 1.19E-12 | 3.41E-13 | 1.21E-13 | 1.21E-12 | 2.03E-13 | 7.19E-14 |
| *N*-docosahexaenoyl serine | 4.66E-12 | 1.43E-12 | 5.06E-13 | 5.47E-12 | 1.38E-12 | 4.87E-13 |
| ***N*-acyl taurine** |  |  |  |  |  |  |
| *N*-arachidonoyl taurine | 8.86E-11 | 9.46E-12 | 3.34E-12 | 8.91E-11 | 1.26E-11 | 4.45E-12 |
| ***N*-acyl tryptophan** |  |  |  |  |  |  |
| *N*-palmitoyl tryptophan | 9.79E-13 | 2.46E-13 | 8.7E-14 | 8.32E-13 | 1.76E-13 | 6.23E-14 |
| *N*-stearoyl tryptophan | 1.91E-12 | 4.74E-13 | 1.68E-13 | 1.98E-12 | 2.36E-13 | 8.34E-14 |
| *N*-oleoyl tryptophan | PISSR |  |  | PISSR |  |  |
| *N*-linoleoyl tryptophan | BDL |  |  | BDL |  |  |
| *N*-arachidonoyl tryptophan | PISSR |  |  | PISSR |  |  |
| *N*-docosahexaenoyl tryptophan | PISSR |  |  | PISSR |  |  |
| ***N*-acyl tyrosine** |  |  |  |  |  |  |
| *N*-palmitoyl tyrosine | 4.32E-13 | 8.05E-14 | 2.85E-14 | 4.66E-13 | 7.75E-14 | 2.74E-14 |
| *N*-stearoyl tyrosine | 6.07E-14 | 3.42E-14 | 1.21E-14 | 5E-14 | 2.09E-14 | 7.38E-15 |
| *N-*oleoyl tyrosine | 3.08E-13 | 3.67E-14 | 1.3E-14 | 3.17E-13 | 9.78E-14 | 3.46E-14 |
| *N*-linoleoyl tyrosine | PISSR |  |  | PISSR |  |  |
| *N*-arachidonoyl tyrosine | 1.17E-13 | 1.79E-14 | 6.33E-15 | 1.23E-13 | 1.23E-14 | 4.33E-15 |
| *N*-docosahexaenoyl tyrosine | 5.04E-13 | 1.1E-13 | 3.89E-14 | 5.3E-13 | 8.28E-14 | 2.93E-14 |
| ***N*-acyl valine** |  |  |  |  |  |  |
| *N*-palmitoyl valine | 3.07E-13 | 5.58E-14 | 1.97E-14 | 3.06E-13 | 3.61E-14 | 1.27E-14 |
| *N*- stearoyl valine | 2.14E-13 | 4.64E-14 | 1.64E-14 | 1.96E-13 | 2.9E-14 | 1.02E-14 |
| *N*-oleoyl valine | 1.97E-13 | 4.64E-14 | 1.64E-14 | 1.69E-13 | 4.02E-14 | 1.42E-14 |
| *N*-nervonoyl valine | BDL |  |  | BDL |  |  |
| *N*-linoleoyl valine | PISSR |  |  | PISSR |  |  |
| *N*-docosahexaenoyl valine | PISSR |  |  | PISSR |  |  |
| **2-acyl glycerols** |  |  |  |  |  |  |
| 2-palmitoyl glycerol | 2.11E-08 | 1.07E-08 | 3.78E-09 | 2.27E-08 | 1.84E-08 | 6.5E-09 |
| 2-oleoyl glycerol | 1.14E-08 | 4.99E-09 | 1.76E-09 | 1.25E-08 | 7.42E-09 | 2.62E-09 |
| 2-linoleoyl glycerol | 8.69E-11 | 4.28E-11 | 1.51E-11 | 8.8E-11 | 5.72E-11 | 2.02E-11 |
| 2-arachidonoyl glycerol | 1.33E-09 | 6.03E-10 | 2.13E-10 | 1.34E-09 | 5.2E-10 | 1.84E-10 |
| **Free Fatty Acids** |  |  |  |  |  |  |
| Oleic acid | 4.07E-07 | 2.18E-07 | 7.71E-08 | 4.35E-07 | 2.92E-07 | 1.03E-07 |
| Linoleic acid | 1.52E-09 | 4.21E-10 | 1.49E-10 | 1.38E-09 | 3.73E-10 | 1.32E-10 |
| Arachidonic acid | 1.39E-08 | 2.44E-09 | 8.64E-10 | 1.41E-08 | 2.07E-09 | 7.33E-10 |
| **PhosphoLEA** |  |  |  |  |  |  |
| PhosphoLEA | 3.7E-11 | 4.21E-12 | 1.49E-12 | 4.98E-11 | 4.65E-12 | 1.64E-12 |
| **Prostaglandins** |  |  |  |  |  |  |
| PGE_2_ | 6.42E-10 | 1.39E-10 | 4.93E-11 | 6.96E-10 | 1.32E-10 | 4.67E-11 |
| PGF_2α_ | 3.49E-10 | 5.98E-11 | 2.12E-11 | 2.81E-10 | 2.71E-11 | 9.6E-12 |
| 6-ketoPGF_1α_ | 9.61E-12 | 1.18E-12 | 4.17E-13 | 6.41E-12 | 1E-12 | 3.55E-13 |
| **THC/CBD** |  |  |  |  |  |  |
| Cannabidiol | BDL |  |  | 7.67E-11 | 2.57E-11 | 9.09E-12 |
| THC | BDL |  |  | BDL |  |  |
| **THC Metabolites** |  |  |  |  |  |  |
| 11-nor-9-carboxyTHC | BDL |  |  | BDL |  |  |
| 11-OH-THC | BDL |  |  | BDL |  |  |

**Supplemental Table 52: List of lipids in the NAPE-PLD KO cortex significantly affected by 3mg/kg CBD**

| NAPE-PLD KO CBD Cortex Significant Differences in One-Way ANOVA | | | | |
| --- | --- | --- | --- | --- |
| Lipid | F | p | Direction (relative to Veh) | Magnitude (x Veh level) |
| *N*-oleoyl alanine | 14.90 | .002 | ↓ | 0.64 |
| *N*-linoleoyl alanine | 6.12 | .027 | ↓ | 0.60 |
| *N*-arachidonoyl methionine | 20.89 | .000 | ↓ | 0.49 |
| *N*-arachidonoyl phenylalanine | 5.32 | .037 | ↓ | 0.82 |
| *N*-oleoyl serine | 5.34 | .037 | ↓ | 0.91 |
| phosphoLEA | 33.35 | .000 | ↑ | 1.35 |
| PGF_2α_ | 8.63 | .011 | ↓ | 0.81 |
| 6-ketoPGF_1α_ | 34.09 | .000 | ↓ | 0.67 |
| CBD | 71.08 | .000 | ↑ | infinite |

**Supplemental Table 53: Lipid levels in the hypothalamus of NAPE-PLD KO female mice treated with Vehicle or 3 mg/kg CBD**

|  | Hypothalamus | | | | | | |
| --- | --- | --- | --- | --- | --- | --- | --- |
|  | Vehicle | | | 3mg/kg CBD | | | |
| ***N*-acyl alanine** | Mean | Std Dev | Std Error | | Mean | Std Dev | Std Error |
| *N*-palmitoyl alanine | 1.88E-11 | 2.9E-12 | 1.02E-12 | | 1.81E-11 | 1.68E-12 | 5.96E-13 |
| *N*-stearoyl alanine | 5.21E-12 | 1.47E-12 | 5.21E-13 | | 5.11E-12 | 8.85E-13 | 3.13E-13 |
| *N*-oleoyl alanine | 1.09E-11 | 3.35E-12 | 1.18E-12 | | 8.34E-12 | 1.29E-12 | 4.58E-13 |
| *N*-linoleoyl alanine | PISSR |  |  | | PISSR |  |  |
| *N*-arachidonoyl alanine | PISSR |  |  | | PISSR |  |  |
| *N*-docosahexaenoyl alanine | BDL |  |  | | BDL |  |  |
| ***N*-acyl dopamine** |  |  |  | |  |  |  |
| *N*-oleoyl dopamine | BDL |  |  | | BDL |  |  |
| *N*-arachidonoyl dopamine | BDL |  |  | | BDL |  |  |
| ***N*-acyl ethanolamine** |  |  |  | |  |  |  |
| *N*-palmitoyl ethanolamine | 3.96E-11 | 1.66E-11 | 5.87E-12 | | 2.09E-11 | 8.16E-12 | 2.88E-12 |
| *N*-stearoyl ethanolamine | 1.56E-11 | 8.38E-12 | 2.96E-12 | | 8.26E-12 | 3.53E-12 | 1.25E-12 |
| *N*-oleoyl ethanolamine | 8.57E-11 | 4.57E-11 | 1.62E-11 | | 4E-11 | 1.61E-11 | 5.68E-12 |
| *N*-linoleoyl ethanolamine | 2.72E-12 | 1.6E-12 | 5.64E-13 | | 1.4E-12 | 6.99E-13 | 2.47E-13 |
| *N*-arachidonoyl ethanolamine | 2.08E-12 | 9.74E-13 | 3.44E-13 | | 9.73E-13 | 3.66E-13 | 1.29E-13 |
| *N*-docosahexaenoyl ethanolamine | 1.48E-11 | 9.74E-12 | 3.44E-12 | | 2.43E-11 | 2.06E-11 | 7.3E-12 |
| ***N*-acyl GABA** |  |  |  | |  |  |  |
| *N*-palmitoyl GABA | 1E-11 | 2.23E-12 | 7.88E-13 | | 8.49E-12 | 1.16E-12 | 4.1E-13 |
| *N*-stearoyl GABA | 8.78E-12 | 2.54E-12 | 8.99E-13 | | 6.77E-12 | 1.61E-12 | 5.71E-13 |
| *N*-oleoyl GABA | 3.73E-12 | 1.48E-12 | 5.23E-13 | | 2.62E-12 | 6.3E-13 | 2.23E-13 |
| *N*-linoleoyl GABA | BDL |  |  | | BDL |  |  |
| *N*-arachidonoyl GABA | 1.18E-11 | 3.11E-12 | 1.1E-12 | | 7.97E-12 | 6.76E-13 | 2.39E-13 |
| *N*-docosahexaenoyl GABA | 7.02E-13 | 4.05E-13 | 1.43E-13 | | 8.05E-13 | 2.13E-13 | 7.52E-14 |
| ***N*-acyl glycine** |  |  |  | |  |  |  |
| *N*-palmitoyl glycine | 1.63E-11 | 4.49E-12 | 1.59E-12 | | 1.37E-11 | 4.77E-12 | 1.69E-12 |
| *N*-stearoyl glycine | 8.16E-12 | 2.33E-12 | 8.25E-13 | | 6.85E-12 | 2.31E-12 | 8.15E-13 |
| *N*-oleoyl glycine | 3.26E-12 | 1.39E-12 | 4.92E-13 | | 2.41E-12 | 5.61E-13 | 1.98E-13 |
| *N*-linoleoyl glycine | 9.35E-13 | 5.86E-13 | 2.07E-13 | | 5.04E-13 | 1.88E-13 | 6.65E-14 |
| *N*-arachidonoyl glycine | 4.13E-12 | 1.44E-12 | 5.08E-13 | | 3.35E-12 | 5.8E-13 | 2.05E-13 |
| *N*-docosahexaenoyl glycine | 7.35E-13 | 3.58E-13 | 1.27E-13 | | 6.88E-13 | 2.42E-13 | 8.56E-14 |
| ***N*-acyl leucine** |  |  |  | |  |  |  |
| *N*-palmitoyl leucine | 6.07E-12 | 1.37E-12 | 4.83E-13 | | 4.77E-12 | 9.8E-13 | 3.46E-13 |
| *N*-stearoyl leucine | 5.09E-12 | 1.37E-12 | 4.84E-13 | | 4.25E-12 | 8.36E-13 | 2.95E-13 |
| *N*-oleoyl leucine | 3.97E-12 | 8.46E-13 | 2.99E-13 | | 3.09E-12 | 6.24E-13 | 2.21E-13 |
| *N*-linoleoyl leucine | PISSR |  |  | | PISSR |  |  |
| *N*-docosahexaenoyl leucine | PISSR |  |  | | PISSR |  |  |
| ***N*-acyl methionine** |  |  |  | |  |  |  |
| *N*-palmitoyl methionine | 4.29E-12 | 1.5E-12 | 5.32E-13 | | 4.9E-12 | 2.92E-12 | 1.03E-12 |
| *N*-stearoyl methionine | PISSR |  |  | | PISSR |  |  |
| *N*-oleoyl methionine | PISSR |  |  | | PISSR |  |  |
| *N*-linoleoyl methionine | BDL |  |  | | BDL |  |  |
| *N*-arachidonoyl methionine | PISSR |  |  | | PISSR |  |  |
| *N*-docosahexaenoyl methionine | PISSR |  |  | | PISSR |  |  |
| ***N*-acyl phenylalanine** |  |  |  | |  |  |  |
| *N*-palmitoyl phenylalanine | 3.43E-12 | 8.28E-13 | 2.93E-13 | | 2.82E-12 | 5.14E-13 | 1.82E-13 |
| *N*-stearoyl phenylalanine | 1.16E-12 | 4.42E-13 | 1.56E-13 | | 1.17E-12 | 3.52E-13 | 1.24E-13 |
| *N*-oleoyl phenylalanine | 1.6E-12 | 5.94E-13 | 2.1E-13 | | 1.16E-12 | 1.88E-13 | 6.66E-14 |
| *N*-linoleoyl phenylalanine | PISSR |  |  | | PISSR |  |  |
| *N*-arachidonoyl phenylalanine | 1.62E-12 | 7.79E-13 | 2.75E-13 | | 1.2E-12 | 3.11E-13 | 1.1E-13 |
| *N*-docosahexaenoyl phenylalanine | PISSR |  |  | | PISSR |  |  |

**Supplemental Table 53: Continued**

|  | Hypothalamus | | | | | |
| --- | --- | --- | --- | --- | --- | --- |
|  | Vehicle | | | 3mg/kg CBD | | |
| ***N*-acyl proline** | Mean | Std Dev | Std Error | Mean | Std Dev | Std Error |
| *N*-palmitoyl proline | 5.66E-13 | 1.6E-13 | 5.65E-14 | 4.89E-13 | 1.68E-13 | 5.93E-14 |
| *N*-stearoyl proline | PISSR |  |  | PISSR |  |  |
| *N-*oleoyl proline | PISSR |  |  | PISSR |  |  |
| *N*-linoleoyl proline | BDL |  |  | BDL |  |  |
| *N*-arachidonoyl proline | PISSR |  |  | PISSR |  |  |
| *N*-docosahexaenoyl proline | BDL |  |  | BDL |  |  |
| ***N*-acyl serine** |  |  |  |  |  |  |
| *N*-palmitoyl serine | 1.04E-11 | 1.3E-12 | 4.6E-13 | 1.47E-11 | 2.59E-12 | 9.15E-13 |
| *N*-stearoyl serine | 7.07E-12 | 2.3E-12 | 8.15E-13 | 8.72E-12 | 3.24E-12 | 1.15E-12 |
| *N*-oleoyl serine | 8.55E-10 | 7.01E-11 | 2.48E-11 | 1.06E-09 | 2.59E-10 | 9.15E-11 |
| *N*-linoleoyl serine | 1.76E-10 | 1.69E-11 | 5.99E-12 | 2.1E-10 | 4.86E-11 | 1.72E-11 |
| *N*-arachidonoyl serine | 4.3E-13 | 1.78E-13 | 6.3E-14 | 4.16E-13 | 2.52E-13 | 8.9E-14 |
| *N*-docosahexaenoyl serine | 1.77E-12 | 6.45E-13 | 2.28E-13 | 2.7E-12 | 7.68E-13 | 2.72E-13 |
| ***N*-acyl taurine** |  |  |  |  |  |  |
| *N*-arachidonoyl taurine | 6.05E-12 | 1.24E-12 | 4.38E-13 | 4.23E-12 | 6.4E-13 | 2.26E-13 |
| ***N*-acyl tryptophan** |  |  |  |  |  |  |
| *N*-palmitoyl tryptophan | PISSR |  |  | PISSR |  |  |
| *N*-stearoyl tryptophan | 4.07E-12 | 2.53E-12 | 8.94E-13 | 5.05E-12 | 3.61E-12 | 1.28E-12 |
| *N*-oleoyl tryptophan | PISSR |  |  | PISSR |  |  |
| *N*-linoleoyl tryptophan | BDL |  |  | BDL |  |  |
| *N*-arachidonoyl tryptophan | BDL |  |  | BDL |  |  |
| *N*-docosahexaenoyl tryptophan | BDL |  |  | BDL |  |  |
| ***N*-acyl tyrosine** |  |  |  |  |  |  |
| *N*-palmitoyl tyrosine | 5.2E-13 | 1.53E-13 | 5.41E-14 | 4.58E-13 | 8.49E-14 | 3E-14 |
| *N*-stearoyl tyrosine | PISSR |  |  | PISSR |  |  |
| *N-*oleoyl tyrosine | PISSR |  |  | PISSR |  |  |
| *N*-linoleoyl tyrosine | PISSR |  |  | PISSR |  |  |
| *N*-arachidonoyl tyrosine | PISSR |  |  | PISSR |  |  |
| *N*-docosahexaenoyl tyrosine | PISSR |  |  | PISSR |  |  |
| ***N*-acyl valine** |  |  |  |  |  |  |
| *N*-palmitoyl valine | 9.57E-13 | 2.32E-13 | 8.2E-14 | 8.71E-13 | 2.69E-13 | 9.52E-14 |
| *N*- stearoyl valine | 3.92E-13 | 1.48E-13 | 5.24E-14 | 3.02E-13 | 1.06E-13 | 3.74E-14 |
| *N*-oleoyl valine | 3.54E-13 | 2.25E-13 | 7.94E-14 | 2.57E-13 | 9.54E-14 | 3.37E-14 |
| *N*-nervonoyl valine | BDL |  |  | BDL |  |  |
| *N*-linoleoyl valine | BDL |  |  | BDL |  |  |
| *N*-docosahexaenoyl valine | PISSR |  |  | PISSR |  |  |
| **2-acyl glycerols** |  |  |  |  |  |  |
| 2-palmitoyl glycerol | 2.17E-08 | 1.12E-08 | 3.95E-09 | 1.4E-08 | 1.08E-08 | 3.82E-09 |
| 2-oleoyl glycerol | 2.33E-08 | 1.56E-08 | 5.53E-09 | 6.95E-09 | 3.94E-09 | 1.39E-09 |
| 2-linoleoyl glycerol | 7.43E-11 | 3.16E-11 | 1.12E-11 | 3.4E-11 | 1.37E-11 | 4.84E-12 |
| 2-arachidonoyl glycerol | 2.27E-09 | 6.36E-10 | 2.25E-10 | 1.6E-09 | 7.39E-10 | 2.61E-10 |
| **Free Fatty Acids** |  |  |  |  |  |  |
| Oleic acid | 2.77E-09 | 5.95E-10 | 2.1E-10 | 1.94E-09 | 3.31E-10 | 1.17E-10 |
| Linoleic acid | 8.84E-10 | 1.4E-10 | 4.93E-11 | 7.67E-10 | 1.25E-10 | 4.43E-11 |
| Arachidonic acid | 8.8E-09 | 1.16E-09 | 4.11E-10 | 6.14E-09 | 3.55E-10 | 1.26E-10 |
| **PhosphoLEA** |  |  |  |  |  |  |
| PhosphoLEA | 2.86E-10 | 7.56E-11 | 2.67E-11 | 4.32E-10 | 3.65E-11 | 1.29E-11 |
| **Prostaglandins** |  |  |  |  |  |  |
| PGE_2_ | 1.46E-10 | 4.42E-11 | 1.56E-11 | 1.07E-10 | 1.29E-11 | 4.56E-12 |
| PGF_2α_ | 2.13E-10 | 5.4E-11 | 1.91E-11 | 1.51E-10 | 1.54E-11 | 5.46E-12 |
| 6-ketoPGF_1α_ | 4.24E-12 | 1.49E-12 | 5.28E-13 | 4E-12 | 5.65E-13 | 2E-13 |
| **THC/CBD** |  |  |  |  |  |  |
| Cannabidiol | BDL |  |  | 6.72E-12 | 9.91E-13 | 3.51E-13 |
| THC | BDL |  |  | BDL |  |  |
| **THC Metabolites** |  |  |  |  |  |  |
| 11-nor-9-carboxyTHC | BDL |  |  | BDL |  |  |
| 11-OH-THC | BDL |  |  | BDL |  |  |

**Supplemental Table 54: List of lipids in the NAPE-PLD KO hypothalamus significantly affected by 3mg/kg CBD**

| NAPE-PLD KO Hypothalamus Significant Differences in One-Way ANOVA | | | | |
| --- | --- | --- | --- | --- |
| Lipid | F | p | Direction (relative to Veh) | Magnitude (x Veh level) |
| *N*-oleoyl alanine | 4.08 | .063 | ↓ | 0.77 |
| *N*-palmitoyl ethanolamine | 8.18 | .013 | ↓ | 0.53 |
| *N*-stearoyl ethanolamine | 5.17 | .039 | ↓ | 0.53 |
| *N*-oleoyl ethanolamine | 7.11 | .018 | ↓ | 0.47 |
| *N*-linoleoyl ethanolamine | 4.60 | .050 | ↓ | 0.52 |
| *N*-arachidonoyl ethanolamine | 9.05 | .009 | ↓ | 0.47 |
| *N*-stearoyl GABA | 3.56 | .080 | ↓ | 0.77 |
| *N*-oleoyl GABA | 3.81 | .071 | ↓ | 0.70 |
| *N*-arachidonoyl GABA | 11.49 | .004 | ↓ | 0.68 |
| *N*-linoleoyl glycine | 3.92 | .068 | ↓ | 0.54 |
| *N*-palmitoyl leucine | 4.77 | .047 | ↓ | 0.79 |
| *N*-oleoyl leucine | 5.60 | .033 | ↓ | 0.78 |
| *N*-palmitoyl phenylalanine | 3.18 | .096 | ↓ | 0.82 |
| *N*-oleoyl phenylalanine | 4.07 | .063 | ↓ | 0.72 |
| *N*-palmitoyl serine | 18.32 | .001 | ↑ | 1.42 |
| *N*-oleoyl serine | 4.71 | .048 | ↑ | 1.24 |
| *N*-linoleoyl serine | 3.45 | .084 | ↑ | 1.19 |
| *N*-docosahexaenoyl serine | 6.94 | .020 | ↑ | 1.53 |
| *N*-arachidonoyl taurine | 13.55 | .002 | ↓ | 0.70 |
| 2-oleoyl glycerol | 8.22 | .012 | ↓ | 0.30 |
| 2-linoleoyl glycerol | 10.93 | .005 | ↓ | 0.46 |
| 2-arachidonoyl glycerol | 3.72 | .074 | ↓ | 0.71 |
| Oleic acid | 11.85 | .004 | ↓ | 0.70 |
| Linoleic acid | 3.12 | .099 | ↓ | 0.87 |
| Arachidonic acid | 38.45 | .000 | ↓ | 0.70 |
| PhosphoLEA | 24.25 | .000 | ↑ | 1.51 |
| PGE_2_ | 5.75 | .031 | ↓ | 0.73 |
| PGF_2α_ | 9.68 | .008 | ↓ | 0.71 |
| CBD | 367.73 | .000 | ↑ | infinite |

**Supplemental Table 55: Lipid levels in the midbrain of NAPE-PLD KO female mice treated with Vehicle or 3 mg/kg CBD**

|  | Midbrain | | | | | | |
| --- | --- | --- | --- | --- | --- | --- | --- |
|  | Vehicle | | | 3mg/kg CBD | | | |
| ***N*-acyl alanine** | Mean | Std Dev | Std Error | | Mean | Std Dev | Std Error |
| *N*-palmitoyl alanine | 8.03E-12 | 2.02E-12 | 7.13E-13 | | 9.69E-12 | 1.82E-12 | 6.42E-13 |
| *N*-stearoyl alanine | 2.61E-12 | 8.55E-13 | 3.02E-13 | | 2.63E-12 | 7.12E-13 | 2.52E-13 |
| *N*-oleoyl alanine | 5.01E-12 | 1.32E-12 | 4.66E-13 | | 4.05E-12 | 7.34E-13 | 2.59E-13 |
| *N*-linoleoyl alanine | 9.34E-13 | 3.02E-13 | 1.07E-13 | | 7.44E-13 | 2.86E-13 | 1.01E-13 |
| *N*-arachidonoyl alanine | 3.11E-12 | 8.9E-13 | 3.15E-13 | | 3.25E-12 | 9.05E-13 | 3.2E-13 |
| *N*-docosahexaenoyl alanine | PISSR |  |  | | PISSR |  |  |
| ***N*-acyl dopamine** |  |  |  | |  |  |  |
| *N*-oleoyl dopamine | BDL |  |  | | BDL |  |  |
| *N*-arachidonoyl dopamine | BDL |  |  | | BDL |  |  |
| ***N*-acyl ethanolamine** |  |  |  | |  |  |  |
| *N*-palmitoyl ethanolamine | 3.41E-10 | 7.55E-11 | 2.67E-11 | | 2.91E-10 | 1.25E-10 | 4.42E-11 |
| *N*-stearoyl ethanolamine | 4.09E-11 | 1.56E-11 | 5.53E-12 | | 4.22E-11 | 2.48E-11 | 8.75E-12 |
| *N*-oleoyl ethanolamine | 8.01E-10 | 1.69E-10 | 5.98E-11 | | 7.46E-10 | 3.2E-10 | 1.13E-10 |
| *N*-linoleoyl ethanolamine | 3.92E-11 | 1.11E-11 | 3.94E-12 | | 3.38E-11 | 9.56E-12 | 3.38E-12 |
| *N*-arachidonoyl ethanolamine | 2.87E-11 | 6.67E-12 | 2.36E-12 | | 2.52E-11 | 9.04E-12 | 3.2E-12 |
| *N*-docosahexaenoyl ethanolamine | 4.28E-11 | 1.32E-11 | 4.65E-12 | | 3.82E-11 | 1.25E-11 | 4.41E-12 |
| ***N*-acyl GABA** |  |  |  | |  |  |  |
| *N*-palmitoyl GABA | 1.47E-11 | 1.99E-12 | 7.04E-13 | | 1.52E-11 | 2.77E-12 | 9.8E-13 |
| *N*-stearoyl GABA | 1.17E-11 | 2.98E-12 | 1.05E-12 | | 1.33E-11 | 2.49E-12 | 8.81E-13 |
| *N*-oleoyl GABA | 8.34E-12 | 1.74E-12 | 6.15E-13 | | 8.85E-12 | 1.29E-12 | 4.55E-13 |
| *N*-linoleoyl GABA | 1.42E-12 | 2.42E-13 | 8.57E-14 | | 1.53E-12 | 4.67E-13 | 1.65E-13 |
| *N*-arachidonoyl GABA | 2.91E-11 | 2.7E-12 | 9.53E-13 | | 3.17E-11 | 4.52E-12 | 1.6E-12 |
| *N*-docosahexaenoyl GABA | 3.9E-12 | 5.52E-13 | 1.95E-13 | | 3.96E-12 | 5.13E-13 | 1.81E-13 |
| ***N*-acyl glycine** |  |  |  | |  |  |  |
| *N*-palmitoyl glycine | 2.72E-11 | 6.61E-12 | 2.34E-12 | | 2.47E-11 | 4.11E-12 | 1.45E-12 |
| *N*-stearoyl glycine | 1.76E-12 | 8.29E-13 | 2.93E-13 | | 1.61E-12 | 9.94E-13 | 3.52E-13 |
| *N*-oleoyl glycine | 9.91E-12 | 1.58E-12 | 5.59E-13 | | 8.37E-12 | 1.53E-12 | 5.39E-13 |
| *N*-linoleoyl glycine | 1.85E-12 | 3.03E-13 | 1.07E-13 | | 1.69E-12 | 2.44E-13 | 8.63E-14 |
| *N*-arachidonoyl glycine | 3.21E-11 | 3.64E-12 | 1.29E-12 | | 3.15E-11 | 3.7E-12 | 1.31E-12 |
| *N*-docosahexaenoyl glycine | 3.5E-12 | 3.95E-13 | 1.4E-13 | | 3.55E-12 | 4.66E-13 | 1.65E-13 |
| ***N*-acyl leucine** |  |  |  | |  |  |  |
| *N*-palmitoyl leucine | 2.76E-12 | 5.95E-13 | 2.1E-13 | | 2.99E-12 | 5.86E-13 | 2.07E-13 |
| *N*-stearoyl leucine | 2.56E-12 | 5.04E-13 | 1.78E-13 | | 2.63E-12 | 4.85E-13 | 1.71E-13 |
| *N*-oleoyl leucine | 5.33E-12 | 1.26E-12 | 4.47E-13 | | 5.48E-12 | 1.04E-12 | 3.67E-13 |
| *N*-linoleoyl leucine | 1.07E-12 | 3.67E-13 | 1.3E-13 | | 1E-12 | 1.21E-13 | 4.29E-14 |
| *N*-docosahexaenoyl leucine | 4.13E-13 | 1.62E-13 | 5.73E-14 | | 4.01E-13 | 9.2E-14 | 3.25E-14 |
| ***N*-acyl methionine** |  |  |  | |  |  |  |
| *N*-palmitoyl methionine | 2.07E-11 | 7.29E-12 | 2.58E-12 | | 2.02E-11 | 6.24E-12 | 2.21E-12 |
| *N*-stearoyl methionine | 2.69E-12 | 8.4E-13 | 2.97E-13 | | 2.72E-12 | 8.56E-13 | 3.03E-13 |
| *N*-oleoyl methionine | 2.64E-12 | 4.21E-13 | 1.49E-13 | | 3.42E-12 | 7.79E-13 | 2.76E-13 |
| *N*-linoleoyl methionine | PISSR |  |  | | PISSR |  |  |
| *N*-arachidonoyl methionine | 3.24E-12 | 8.47E-13 | 3E-13 | | 3.25E-12 | 8.53E-13 | 3.01E-13 |
| *N*-docosahexaenoyl methionine | BDL |  |  | | BDL |  |  |
| ***N*-acyl phenylalanine** |  |  |  | |  |  |  |
| *N*-palmitoyl phenylalanine | 4.23E-12 | 8.93E-13 | 3.16E-13 | | 4.13E-12 | 6E-13 | 2.12E-13 |
| *N*-stearoyl phenylalanine | 1.14E-12 | 3.6E-13 | 1.27E-13 | | 9.58E-13 | 3.62E-13 | 1.28E-13 |
| *N*-oleoyl phenylalanine | 2.35E-12 | 4.78E-13 | 1.69E-13 | | 2.14E-12 | 3.1E-13 | 1.09E-13 |
| *N*-linoleoyl phenylalanine | 8.86E-13 | 2.41E-13 | 8.54E-14 | | 6.52E-13 | 1.81E-13 | 6.39E-14 |
| *N*-arachidonoyl phenylalanine | 3.8E-12 | 5.24E-13 | 1.85E-13 | | 2.98E-12 | 4.26E-13 | 1.51E-13 |
| *N*-docosahexaenoyl phenylalanine | 2.78E-12 | 3.98E-13 | 1.41E-13 | | 2.69E-12 | 3.38E-13 | 1.2E-13 |

**Supplemental Table 55: Continued**

|  | Midbrain | | | | | |
| --- | --- | --- | --- | --- | --- | --- |
|  | Vehicle | | | 3mg/kg CBD | | |
| ***N*-acyl proline** | Mean | Std Dev | Std Error | Mean | Std Dev | Std Error |
| *N*-palmitoyl proline | 2.82E-13 | 9.76E-14 | 3.45E-14 | 2.91E-13 | 7.59E-14 | 2.69E-14 |
| *N*-stearoyl proline | PISSR |  |  | PISSR |  |  |
| *N-*oleoyl proline | PISSR |  |  | PISSR |  |  |
| *N*-linoleoyl proline | PISSR |  |  | PISSR |  |  |
| *N*-arachidonoyl proline | PISSR |  |  | PISSR |  |  |
| *N*-docosahexaenoyl proline | PISSR |  |  | PISSR |  |  |
| ***N*-acyl serine** |  |  |  |  |  |  |
| *N*-palmitoyl serine | 7.25E-12 | 2.61E-12 | 9.22E-13 | 7.95E-12 | 2.32E-12 | 8.19E-13 |
| *N*-stearoyl serine | 5.55E-12 | 2.12E-12 | 7.49E-13 | 5.48E-12 | 1E-12 | 3.55E-13 |
| *N*-oleoyl serine | 3.43E-10 | 3.43E-11 | 1.21E-11 | 3.39E-10 | 4.33E-11 | 1.53E-11 |
| *N*-linoleoyl serine | 6.75E-11 | 7.42E-12 | 2.62E-12 | 7.11E-11 | 1.22E-11 | 4.33E-12 |
| *N*-arachidonoyl serine | 1.63E-12 | 2.35E-13 | 8.31E-14 | 1.36E-12 | 2.34E-13 | 8.27E-14 |
| *N*-docosahexaenoyl serine | 4.46E-12 | 1.01E-12 | 3.56E-13 | 4.8E-12 | 1.85E-12 | 6.53E-13 |
| ***N*-acyl taurine** |  |  |  |  |  |  |
| *N*-arachidonoyl taurine | 1.55E-11 | 1.98E-12 | 6.99E-13 | 1.52E-11 | 1.28E-12 | 4.51E-13 |
| ***N*-acyl tryptophan** |  |  |  |  |  |  |
| *N*-palmitoyl tryptophan | 9.36E-13 | 4.9E-13 | 1.73E-13 | 8.25E-13 | 4.32E-13 | 1.53E-13 |
| *N*-stearoyl tryptophan | 3.87E-12 | 1.01E-12 | 3.55E-13 | 4.59E-12 | 7.38E-13 | 2.61E-13 |
| *N*-oleoyl tryptophan | PISSR |  |  | PISSR |  |  |
| *N*-linoleoyl tryptophan | BDL |  |  | BDL |  |  |
| *N*-arachidonoyl tryptophan | PISSR |  |  | PISSR |  |  |
| *N*-docosahexaenoyl tryptophan | PISSR |  |  | PISSR |  |  |
| ***N*-acyl tyrosine** |  |  |  |  |  |  |
| *N*-palmitoyl tyrosine | 4.99E-13 | 1.07E-13 | 3.79E-14 | 4.79E-13 | 7.65E-14 | 2.7E-14 |
| *N*-stearoyl tyrosine | PISSR |  |  | PISSR |  |  |
| *N-*oleoyl tyrosine | 4.01E-13 | 1.76E-13 | 6.22E-14 | 4.19E-13 | 1.02E-13 | 3.61E-14 |
| *N*-linoleoyl tyrosine | PISSR |  |  | PISSR |  |  |
| *N*-arachidonoyl tyrosine | 2.25E-13 | 6.06E-14 | 2.14E-14 | 2.11E-13 | 8.07E-14 | 2.85E-14 |
| *N*-docosahexaenoyl tyrosine | 5.91E-13 | 2.33E-13 | 8.22E-14 | 4.28E-13 | 1.91E-13 | 6.74E-14 |
| ***N*-acyl valine** |  |  |  |  |  |  |
| *N*-palmitoyl valine | 5.46E-13 | 1.2E-13 | 4.25E-14 | 5.88E-13 | 1.78E-13 | 6.3E-14 |
| *N*- stearoyl valine | 2.45E-13 | 1.75E-13 | 6.17E-14 | 2.1E-13 | 6.89E-14 | 2.43E-14 |
| *N*-oleoyl valine | 2.55E-13 | 8.71E-14 | 3.08E-14 | 2.71E-13 | 7.85E-14 | 2.77E-14 |
| *N*-nervonoyl valine | BDL |  |  | BDL |  |  |
| *N*-linoleoyl valine | PISSR |  |  | PISSR |  |  |
| *N*-docosahexaenoyl valine | PISSR |  |  | PISSR |  |  |
| **2-acyl glycerols** |  |  |  |  |  |  |
| 2-palmitoyl glycerol | 5.01E-09 | 1.83E-09 | 6.48E-10 | 5.06E-09 | 1.49E-09 | 5.27E-10 |
| 2-oleoyl glycerol | 6.77E-08 | 1.77E-08 | 6.27E-09 | 6.39E-08 | 1.89E-08 | 6.67E-09 |
| 2-linoleoyl glycerol | 2.89E-10 | 1.47E-10 | 5.19E-11 | 2.77E-10 | 1.45E-10 | 5.12E-11 |
| 2-arachidonoyl glycerol | 2.82E-09 | 6.24E-10 | 2.21E-10 | 2.46E-09 | 6.05E-10 | 2.14E-10 |
| **Free Fatty Acids** |  |  |  |  |  |  |
| Oleic acid | 1.03E-07 | 4.97E-08 | 1.76E-08 | 1.36E-07 | 6.29E-08 | 2.22E-08 |
| Linoleic acid | 1.14E-09 | 3.15E-10 | 1.11E-10 | 1.23E-09 | 3.14E-10 | 1.11E-10 |
| Arachidonic acid | 1.01E-08 | 2.61E-09 | 9.24E-10 | 1.29E-08 | 2.89E-09 | 1.02E-09 |
| **PhosphoLEA** |  |  |  |  |  |  |
| PhosphoLEA | 6.85E-11 | 2.88E-12 | 1.02E-12 | 8.17E-11 | 4.88E-12 | 1.73E-12 |
| **Prostaglandins** |  |  |  |  |  |  |
| PGE_2_ | 2.55E-10 | 4.09E-11 | 1.45E-11 | 2.25E-10 | 1.46E-11 | 5.16E-12 |
| PGF_2α_ | 2.33E-10 | 3.56E-11 | 1.26E-11 | 1.84E-10 | 1.93E-11 | 6.83E-12 |
| 6-ketoPGF_1α_ | 7.61E-12 | 1.16E-12 | 4.11E-13 | 6.1E-12 | 1.24E-12 | 4.38E-13 |
| **THC/CBD** |  |  |  |  |  |  |
| Cannabidiol | BDL |  |  | 3.47E-11 | 5.99E-12 | 2.12E-12 |
| THC | BDL |  |  | BDL |  |  |
| **THC Metabolites** |  |  |  |  |  |  |
| 11-nor-9-carboxyTHC | BDL |  |  | BDL |  |  |
| 11-OH-THC | BDL |  |  | BDL |  |  |

**Supplemental Table 56: List of lipids in the NAPE-PLD KO midbrain significantly affected by 3mg/kg CBD**

| NAPE-PLD KO CBD Midbrain Significant Differences in One-Way ANOVA | | | | |
| --- | --- | --- | --- | --- |
| Lipid | F | p | Direction (relative to Veh) | Magnitude (x Veh level) |
| *N*-oleoyl alanine | 3.28 | .092 | ↓ | 0.81 |
| *N*-oleoyl glycine | 3.92 | .068 | ↓ | 0.84 |
| *N*-oleoyl methionine | 6.22 | .026 | ↑ | 1.30 |
| *N*-linoleoyl phenylalanine | 4.81 | .046 | ↓ | 0.74 |
| *N*-arachidonoyl phenylalanine | 11.74 | .004 | ↓ | 0.78 |
| *N*-arachidonoyl serine | 5.45 | .035 | ↓ | 0.83 |
| PhosphoLEA | 43.64 | .000 | ↑ | 1.19 |
| PGE_2_ | 3.86 | .070 | ↓ | 0.88 |
| PGF_2α_ | 11.94 | .004 | ↓ | 0.79 |
| 6-ketoPGF_1α_ | 6.30 | .025 | ↓ | 0.80 |
| CBD | 268.65 | .000 | ↑ | infinite |

**Supplemental Table 57: Lipid levels in the brainstem of NAPE-PLD KO female mice treated with Vehicle or 3 mg/kg CBD**

|  | Brainstem | | | | | | |
| --- | --- | --- | --- | --- | --- | --- | --- |
|  | Vehicle | | | 3mg/kg CBD | | | |
| ***N*-acyl alanine** | Mean | Std Dev | Std Error | | Mean | Std Dev | Std Error |
| *N*-palmitoyl alanine | 2.22E-11 | 2.76E-12 | 9.76E-13 | | 2.28E-11 | 3.66E-12 | 1.29E-12 |
| *N*-stearoyl alanine | 1.35E-11 | 2.4E-12 | 8.47E-13 | | 1.4E-11 | 2.75E-12 | 9.72E-13 |
| *N*-oleoyl alanine | 1.65E-11 | 6.42E-12 | 2.27E-12 | | 1.24E-11 | 2.27E-12 | 8.02E-13 |
| *N*-linoleoyl alanine | 3.03E-12 | 8.87E-13 | 3.14E-13 | | 2.56E-12 | 5.88E-13 | 2.08E-13 |
| *N*-arachidonoyl alanine | 4.52E-12 | 5.32E-13 | 1.88E-13 | | 5.46E-12 | 7.56E-13 | 2.67E-13 |
| *N*-docosahexaenoyl alanine | 2.26E-12 | 4.28E-13 | 1.51E-13 | | 2.34E-12 | 8.36E-13 | 2.95E-13 |
| ***N*-acyl dopamine** |  |  |  | |  |  |  |
| *N*-oleoyl dopamine | BDL |  |  | | BDL |  |  |
| *N*-arachidonoyl dopamine | BDL |  |  | | BDL |  |  |
| ***N*-acyl ethanolamine** |  |  |  | |  |  |  |
| *N*-palmitoyl ethanolamine | 5.56E-10 | 2.05E-10 | 7.26E-11 | | 4.73E-10 | 2.11E-10 | 7.46E-11 |
| *N*-stearoyl ethanolamine | 9.16E-11 | 4.03E-11 | 1.42E-11 | | 8.71E-11 | 5.76E-11 | 2.04E-11 |
| *N*-oleoyl ethanolamine | 9.63E-10 | 3.19E-10 | 1.13E-10 | | 8.78E-10 | 3.49E-10 | 1.23E-10 |
| *N*-linoleoyl ethanolamine | 3.41E-11 | 1.36E-11 | 4.81E-12 | | 3.42E-11 | 1.16E-11 | 4.11E-12 |
| *N*-arachidonoyl ethanolamine | 2.63E-11 | 8.72E-12 | 3.08E-12 | | 2.49E-11 | 9.69E-12 | 3.43E-12 |
| *N*-docosahexaenoyl ethanolamine | 3.98E-11 | 1.36E-11 | 4.81E-12 | | 3.82E-11 | 9.66E-12 | 3.42E-12 |
| ***N*-acyl GABA** |  |  |  | |  |  |  |
| *N*-palmitoyl GABA | 1.6E-11 | 2.02E-12 | 7.15E-13 | | 1.67E-11 | 2.84E-12 | 1.01E-12 |
| *N*-stearoyl GABA | 1.69E-11 | 3.26E-12 | 1.15E-12 | | 1.77E-11 | 2.78E-12 | 9.82E-13 |
| *N*-oleoyl GABA | 1.32E-11 | 1.06E-12 | 3.76E-13 | | 1.46E-11 | 2.85E-12 | 1.01E-12 |
| *N*-linoleoyl GABA | 1.72E-12 | 2.36E-13 | 8.36E-14 | | 2.12E-12 | 6.48E-13 | 2.29E-13 |
| *N*-arachidonoyl GABA | 1.36E-11 | 1.6E-12 | 5.66E-13 | | 1.7E-11 | 2.81E-12 | 9.94E-13 |
| *N*-docosahexaenoyl GABA | 3.41E-12 | 4.75E-13 | 1.68E-13 | | 4.18E-12 | 8.57E-13 | 3.03E-13 |
| ***N*-acyl glycine** |  |  |  | |  |  |  |
| *N*-palmitoyl glycine | 3.09E-11 | 5.93E-12 | 2.1E-12 | | 2.91E-11 | 4.75E-12 | 1.68E-12 |
| *N*-stearoyl glycine | 1.89E-11 | 6.59E-12 | 2.33E-12 | | 1.65E-11 | 3.66E-12 | 1.29E-12 |
| *N*-oleoyl glycine | 2.34E-11 | 5.23E-12 | 1.85E-12 | | 2.18E-11 | 5.53E-12 | 1.96E-12 |
| *N*-linoleoyl glycine | 1.88E-12 | 3.98E-13 | 1.41E-13 | | 1.65E-12 | 4E-13 | 1.41E-13 |
| *N*-arachidonoyl glycine | 2.71E-11 | 4.28E-12 | 1.51E-12 | | 2.58E-11 | 5.41E-12 | 1.91E-12 |
| *N*-docosahexaenoyl glycine | 7.35E-13 | 3.58E-13 | 1.27E-13 | | 6.88E-13 | 2.42E-13 | 8.56E-14 |
| ***N*-acyl leucine** |  |  |  | |  |  |  |
| *N*-palmitoyl leucine | 2.99E-12 | 5.76E-13 | 2.03E-13 | | 3.5E-12 | 7.09E-13 | 2.51E-13 |
| *N*-stearoyl leucine | 2.87E-12 | 5.22E-13 | 1.85E-13 | | 3.3E-12 | 8.13E-13 | 2.87E-13 |
| *N*-oleoyl leucine | 4.64E-12 | 4.64E-13 | 1.64E-13 | | 4.32E-12 | 9.28E-13 | 3.28E-13 |
| *N*-linoleoyl leucine | PISSR |  |  | | PISSR |  |  |
| *N*-docosahexaenoyl leucine | PISSR |  |  | | PISSR |  |  |
| ***N*-acyl methionine** |  |  |  | |  |  |  |
| *N*-palmitoyl methionine | 1.27E-11 | 6.76E-12 | 2.39E-12 | | 1.29E-11 | 5.79E-12 | 2.05E-12 |
| *N*-stearoyl methionine | 3.88E-12 | 1.55E-12 | 5.49E-13 | | 3.77E-12 | 1.09E-12 | 3.85E-13 |
| *N*-oleoyl methionine | 3.54E-12 | 1.14E-12 | 4.03E-13 | | 3.59E-12 | 1.4E-12 | 4.95E-13 |
| *N*-linoleoyl methionine | PISSR |  |  | | PISSR |  |  |
| *N*-arachidonoyl methionine | 2.15E-12 | 1.03E-12 | 3.64E-13 | | 1.61E-12 | 7.98E-13 | 2.82E-13 |
| *N*-docosahexaenoyl methionine | PISSR |  |  | | PISSR |  |  |
| ***N*-acyl phenylalanine** |  |  |  | |  |  |  |
| *N*-palmitoyl phenylalanine | 5.14E-12 | 1.29E-12 | 4.58E-13 | | 4.84E-12 | 1.09E-12 | 3.85E-13 |
| *N*-stearoyl phenylalanine | 1.63E-12 | 5.15E-13 | 1.82E-13 | | 1.43E-12 | 3.42E-13 | 1.21E-13 |
| *N*-oleoyl phenylalanine | 2.55E-12 | 7.56E-13 | 2.67E-13 | | 2.54E-12 | 6.99E-13 | 2.47E-13 |
| *N*-linoleoyl phenylalanine | 9.09E-13 | 3E-13 | 1.06E-13 | | 7.17E-13 | 2.13E-13 | 7.55E-14 |
| *N*-arachidonoyl phenylalanine | 2.62E-12 | 3.83E-13 | 1.36E-13 | | 2.92E-12 | 6.23E-13 | 2.2E-13 |
| *N*-docosahexaenoyl phenylalanine | 2.25E-12 | 3.16E-13 | 1.12E-13 | | 2.44E-12 | 3.74E-13 | 1.32E-13 |

**Supplemental Table 57: Continued**

|  | Brainstem | | | | | |
| --- | --- | --- | --- | --- | --- | --- |
|  | Vehicle | | | 3mg/kg CBD | | |
| ***N*-acyl proline** | Mean | Std Dev | Std Error | Mean | Std Dev | Std Error |
| *N*-palmitoyl proline | BDL |  |  | BDL |  |  |
| *N*-stearoyl proline | BDL |  |  | BDL |  |  |
| *N-*oleoyl proline | BDL |  |  | BDL |  |  |
| *N*-linoleoyl proline | BDL |  |  | BDL |  |  |
| *N*-arachidonoyl proline | BDL |  |  | BDL |  |  |
| *N*-docosahexaenoyl proline | BDL |  |  | BDL |  |  |
| ***N*-acyl serine** |  |  |  |  |  |  |
| *N*-palmitoyl serine | 6.03E-11 | 1.71E-11 | 6.04E-12 | 5.7E-11 | 1.48E-11 | 5.23E-12 |
| *N*-stearoyl serine | 8.88E-12 | 2.26E-12 | 7.99E-13 | 8.94E-12 | 1.9E-12 | 6.71E-13 |
| *N*-oleoyl serine | 2.99E-10 | 5.76E-11 | 2.04E-11 | 3.12E-10 | 4.96E-11 | 1.75E-11 |
| *N*-linoleoyl serine | 6.54E-11 | 9.88E-12 | 3.49E-12 | 6.44E-11 | 1.07E-11 | 3.77E-12 |
| *N*-arachidonoyl serine | 2.29E-12 | 7.09E-13 | 2.51E-13 | 2.34E-12 | 7.8E-13 | 2.76E-13 |
| *N*-docosahexaenoyl serine | 7.53E-12 | 1.75E-12 | 6.19E-13 | 7.36E-12 | 2.6E-12 | 9.2E-13 |
| ***N*-acyl taurine** |  |  |  |  |  |  |
| *N*-arachidonoyl taurine | 1.67E-11 | 1.34E-12 | 4.74E-13 | 1.46E-11 | 1.67E-12 | 5.92E-13 |
| ***N*-acyl tryptophan** |  |  |  |  |  |  |
| *N*-palmitoyl tryptophan | BDL |  |  | BDL |  |  |
| *N*-stearoyl tryptophan | BDL |  |  | BDL |  |  |
| *N*-oleoyl tryptophan | BDL |  |  | BDL |  |  |
| *N*-linoleoyl tryptophan | BDL |  |  | BDL |  |  |
| *N*-arachidonoyl tryptophan | BDL |  |  | BDL |  |  |
| *N*-docosahexaenoyl tryptophan | BDL |  |  | BDL |  |  |
| ***N*-acyl tyrosine** |  |  |  |  |  |  |
| *N*-palmitoyl tyrosine | 1.03E-12 | 8.87E-14 | 3.14E-14 | 9.69E-13 | 1.08E-13 | 3.82E-14 |
| *N*-stearoyl tyrosine | 6.88E-14 | 2.32E-14 | 8.21E-15 | 8.3E-14 | 6.59E-14 | 2.33E-14 |
| *N-*oleoyl tyrosine | 8.88E-13 | 2.54E-13 | 8.97E-14 | 7.69E-13 | 1.2E-13 | 4.23E-14 |
| *N*-linoleoyl tyrosine | PISSR |  |  | PISSR |  |  |
| *N*-arachidonoyl tyrosine | 2.42E-13 | 9.08E-14 | 3.21E-14 | 2.93E-13 | 7.97E-14 | 2.82E-14 |
| *N*-docosahexaenoyl tyrosine | 1.18E-12 | 3.69E-13 | 1.3E-13 | 1.19E-12 | 2.61E-13 | 9.22E-14 |
| ***N*-acyl valine** |  |  |  |  |  |  |
| *N*-palmitoyl valine | 3.28E-13 | 5.43E-14 | 1.92E-14 | 3.86E-13 | 1.09E-13 | 3.85E-14 |
| *N*- stearoyl valine | 1.89E-13 | 3.45E-14 | 1.22E-14 | 2.18E-13 | 1.09E-13 | 3.85E-14 |
| *N*-oleoyl valine | 2.58E-13 | 8.76E-14 | 3.1E-14 | 2.25E-13 | 5.05E-14 | 1.79E-14 |
| *N*-nervonoyl valine | BDL |  |  | BDL |  |  |
| *N*-linoleoyl valine | PISSR |  |  | PISSR |  |  |
| *N*-docosahexaenoyl valine | 5.02E-14 | 2.87E-14 | 1.01E-14 | 5.34E-14 | 3.14E-14 | 1.11E-14 |
| **2-acyl glycerols** |  |  |  |  |  |  |
| 2-palmitoyl glycerol | 2.17E-09 | 7.33E-10 | 2.59E-10 | 2.67E-09 | 7.1E-10 | 2.51E-10 |
| 2-oleoyl glycerol | 5.06E-08 | 1.34E-08 | 4.75E-09 | 5.04E-08 | 1.23E-08 | 4.33E-09 |
| 2-linoleoyl glycerol | 2.21E-10 | 4.61E-11 | 1.63E-11 | 2.92E-10 | 6E-11 | 2.12E-11 |
| 2-arachidonoyl glycerol | 2.33E-09 | 1.97E-10 | 6.95E-11 | 2.87E-09 | 5.8E-10 | 2.05E-10 |
| **Free Fatty Acids** |  |  |  |  |  |  |
| Oleic acid | 4.72E-08 | 1.01E-08 | 3.57E-09 | 6.25E-08 | 1.47E-08 | 5.19E-09 |
| Linoleic acid | 7.53E-10 | 4.81E-11 | 1.7E-11 | 9.03E-10 | 7.9E-11 | 2.79E-11 |
| Arachidonic acid | 4E-09 | 6.66E-10 | 2.35E-10 | 4.94E-09 | 5.79E-10 | 2.05E-10 |
| **PhosphoLEA** |  |  |  |  |  |  |
| PhosphoLEA | 5.63E-11 | 9.57E-12 | 3.38E-12 | 7.9E-11 | 7.39E-12 | 2.61E-12 |
| **Prostaglandins** |  |  |  |  |  |  |
| PGE_2_ | 3.33E-10 | 5.19E-11 | 1.83E-11 | 3E-10 | 3.3E-11 | 1.17E-11 |
| PGF_2α_ | 2.53E-10 | 4.87E-11 | 1.72E-11 | 2.02E-10 | 3.78E-11 | 1.34E-11 |
| 6-ketoPGF_1α_ | 1.19E-11 | 9.47E-13 | 3.35E-13 | 1.08E-11 | 1.59E-12 | 5.61E-13 |
| **THC/CBD** |  |  |  |  |  |  |
| Cannabidiol | BDL |  |  | 8.97E-11 | 2.12E-11 | 7.48E-12 |
| THC | BDL |  |  | BDL |  |  |
| **THC Metabolites** |  |  |  |  |  |  |
| 11-nor-9-carboxyTHC | BDL |  |  | BDL |  |  |
| 11-OH-THC | BDL |  |  | BDL |  |  |

**Supplemental Table 58: List of lipids in the NAPE-PLD KO brainstem significantly affected by 3mg/kg CBD**

| NAPE-PLD KO Brainstem Significant Differences in One-Way ANOVA | | | | |
| --- | --- | --- | --- | --- |
| Lipid | F | p | Direction (relative to Veh) | Magnitude (x Veh level) |
| *N*-arachidonoyl alanine | 8.21 | .012 | ↑ | 1.21 |
| *N*-arachidonoyl GABA | 8.69 | .011 | ↑ | 1.25 |
| *N*-docosahexaenoyl GABA | 4.94 | .043 | ↑ | 1.23 |
| *N*-docosahexaenoyl glycine | 4.10 | .062 | ↑ | 1.17 |
| *N*-arachidonoyl taurine | 7.80 | .014 | ↓ | 0.87 |
| 2-linoleoyl glycerol | 6.81 | .020 | ↑ | 1.32 |
| 2-arachidonoyl glycerol | 6.21 | .023 | ↑ | 1.23 |
| Oleic acid | 5.93 | .039 | ↑ | 1.32 |
| Linoleic acid | 21.21 | .000 | ↑ | 1.20 |
| Arachidonic acid | 9.14 | .009 | ↑ | 1.23 |
| PhosphoLEA | 28.37 | .000 | ↑ | 1.40 |
| PGF_2α_ | 5.46 | .035 | ↓ | 0.80 |
| 6-ketoPGF_1α_ | 3.17 | .091 | ↓ | 0.91 |
| CBD | 143.73 | .000 | ↑ | infinite |

Supplemental Table 59: CBD level descriptive statistics in 8 brain regions of C57 WT female mice 2 hours after an acute 3 mg/kg CBD injection. Levels are in moles per gram of tissue.

|  | N | Mean | Std. Deviation | Std. Error | 95% Confidence Interval for Mean | | Minimum | Maximum |
| --- | --- | --- | --- | --- | --- | --- | --- | --- |
|  |  |  |  |  | Lower Bound | Upper Bound | |  |
| STR | 8 | 3.92E-11 | 9.70E-12 | 3.43E-12 | 3.11E-11 | 4.74E-11 | 2.78E-11 | 5.45E-11 |
| HIPP | 9 | 3.56E-11 | 1.00E-11 | 3.34E-12 | 2.79E-11 | 4.33E-11 | 2.41E-11 | 5.88E-11 |
| CER | 9 | 3.46E-11 | 9.78E-12 | 3.26E-12 | 2.71E-11 | 4.21E-11 | 2.56E-11 | 5.38E-11 |
| THAL | 9 | 3.55E-11 | 9.76E-12 | 3.25E-12 | 2.80E-11 | 4.30E-11 | 2.66E-11 | 5.13E-11 |
| CTX | 9 | 2.58E-11 | 8.02E-12 | 2.67E-12 | 1.97E-11 | 3.20E-11 | 1.68E-11 | 3.84E-11 |
| HYP | 8 | 7.64E-12 | 1.22E-12 | 4.31E-13 | 6.62E-12 | 8.66E-12 | 6.13E-12 | 9.98E-12 |
| MID | 9 | 3.36E-11 | 7.94E-12 | 2.65E-12 | 2.75E-11 | 3.97E-11 | 2.45E-11 | 4.42E-11 |
| STEM | 9 | 2.46E-11 | 4.40E-12 | 1.47E-12 | 2.13E-11 | 2.80E-11 | 2.06E-11 | 3.15E-11 |
| Total | 70 | 2.98E-11 | 1.21E-11 | 1.45E-12 | 2.69E-11 | 3.26E-11 | 6.13E-12 | 5.88E-11 |

Supplemental Table 60: Results of ANOVA for WT animals to determine effect of brain region on levels of CBD

|  | Sum of Squares | df | Mean Square | F | Sig. |
| --- | --- | --- | --- | --- | --- |
| Between Groups | 5.96E-21 | 7 | 8.51E-22 | 12.634 | 6.02E-10 |
| Within Groups | 4.18E-21 | 62 | 6.73E-23 |  |  |
| Total | 1.01E-20 | 69 |  |  |  |

Supplemental Table 61: Results of post-hoc Fisher’s LSD for WT animals

| (I) Region | (J) Region | Mean Difference (I-J) | Std. Error | Sig. | 95% Confidence Interval | |
| --- | --- | --- | --- | --- | --- | --- |
|  |  |  |  |  | Lower Bound | Upper Bound |
| STR | HIPP | 3.62E-12 | 3.99E-12 | 0.368 | -4.36E-12 | 1.16E-11 |
|  | CER | 4.64E-12 | 3.99E-12 | 0.249 | -3.33E-12 | 1.26E-11 |
|  | THAL | 3.78E-12 | 3.99E-12 | 0.347 | -4.19E-12 | 1.17E-11 |
|  | CTX | 1.34010E-011* | 3.99E-12 | 0.001 | 5.43E-12 | 2.14E-11 |
|  | HYP | 3.16053E-011* | 4.10E-12 | 0 | 2.34E-11 | 3.98E-11 |
|  | MID | 5.63E-12 | 3.99E-12 | 0.163 | -2.34E-12 | 1.36E-11 |
|  | STEM | 1.46017E-011* | 3.99E-12 | 0.001 | 6.63E-12 | 2.26E-11 |
| HIPP | STR | -3.62E-12 | 3.99E-12 | 0.368 | -1.16E-11 | 4.36E-12 |
|  | CER | 1.03E-12 | 3.87E-12 | 0.791 | -6.70E-12 | 8.76E-12 |
|  | THAL | 1.61E-13 | 3.87E-12 | 0.967 | -7.57E-12 | 7.89E-12 |
|  | CTX | 9.78599E-012* | 3.87E-12 | 0.014 | 2.05E-12 | 1.75E-11 |
|  | HYP | 2.79903E-011* | 3.99E-12 | 0 | 2.00E-11 | 3.60E-11 |
|  | MID | 2.02E-12 | 3.87E-12 | 0.604 | -5.72E-12 | 9.75E-12 |
|  | STEM | 1.09866E-011* | 3.87E-12 | 0.006 | 3.25E-12 | 1.87E-11 |
| CER | STR | -4.64E-12 | 3.99E-12 | 0.249 | -1.26E-11 | 3.33E-12 |
|  | HIPP | -1.03E-12 | 3.87E-12 | 0.791 | -8.76E-12 | 6.70E-12 |
|  | THAL | -8.68E-13 | 3.87E-12 | 0.823 | -8.60E-12 | 6.86E-12 |
|  | CTX | 8.75662E-012* | 3.87E-12 | 0.027 | 1.02E-12 | 1.65E-11 |
|  | HYP | 2.69610E-011* | 3.99E-12 | 0 | 1.90E-11 | 3.49E-11 |
|  | MID | 9.86E-13 | 3.87E-12 | 0.8 | -6.75E-12 | 8.72E-12 |
|  | STEM | 9.95728E-012* | 3.87E-12 | 0.012 | 2.22E-12 | 1.77E-11 |
| THAL | STR | -3.78E-12 | 3.99E-12 | 0.347 | -1.17E-11 | 4.19E-12 |
|  | HIPP | -1.61E-13 | 3.87E-12 | 0.967 | -7.89E-12 | 7.57E-12 |
|  | CER | 8.68E-13 | 3.87E-12 | 0.823 | -6.86E-12 | 8.60E-12 |
|  | CTX | 9.62476E-012* | 3.87E-12 | 0.016 | 1.89E-12 | 1.74E-11 |
|  | HYP | 2.78291E-011* | 3.99E-12 | 0 | 1.99E-11 | 3.58E-11 |
|  | MID | 1.85E-12 | 3.87E-12 | 0.633 | -5.88E-12 | 9.59E-12 |
|  | STEM | 1.08254E-011* | 3.87E-12 | 0.007 | 3.09E-12 | 1.86E-11 |
| CTX | STR | -1.34010E-011* | 3.99E-12 | 0.001 | -2.14E-11 | -5.43E-12 |
|  | HIPP | -9.78599E-012* | 3.87E-12 | 0.014 | -1.75E-11 | -2.05E-12 |
|  | CER | -8.75662E-012* | 3.87E-12 | 0.027 | -1.65E-11 | -1.02E-12 |
|  | THAL | -9.62476E-012* | 3.87E-12 | 0.016 | -1.74E-11 | -1.89E-12 |
|  | HYP | 1.82043E-011* | 3.99E-12 | 0 | 1.02E-11 | 2.62E-11 |
|  | MID | -7.77052E-012* | 3.87E-12 | 0.049 | -1.55E-11 | -3.75E-14 |
|  | STEM | 1.20E-12 | 3.87E-12 | 0.757 | -6.53E-12 | 8.93E-12 |
| HYP | STR | -3.16053E-011* | 4.10E-12 | 0 | -3.98E-11 | -2.34E-11 |
|  | HIPP | -2.79903E-011* | 3.99E-12 | 0 | -3.60E-11 | -2.00E-11 |
|  | CER | -2.69610E-011* | 3.99E-12 | 0 | -3.49E-11 | -1.90E-11 |
|  | THAL | -2.78291E-011* | 3.99E-12 | 0 | -3.58E-11 | -1.99E-11 |
|  | CTX | -1.82043E-011* | 3.99E-12 | 0 | -2.62E-11 | -1.02E-11 |
|  | MID | -2.59749E-011* | 3.99E-12 | 0 | -3.39E-11 | -1.80E-11 |
|  | STEM | -1.70037E-011* | 3.99E-12 | 0 | -2.50E-11 | -9.03E-12 |
| MID | STR | -5.63E-12 | 3.99E-12 | 0.163 | -1.36E-11 | 2.34E-12 |
|  | HIPP | -2.02E-12 | 3.87E-12 | 0.604 | -9.75E-12 | 5.72E-12 |
|  | CER | -9.86E-13 | 3.87E-12 | 0.8 | -8.72E-12 | 6.75E-12 |
|  | THAL | -1.85E-12 | 3.87E-12 | 0.633 | -9.59E-12 | 5.88E-12 |
|  | CTX | 7.77052E-012* | 3.87E-12 | 0.049 | 3.75E-14 | 1.55E-11 |
|  | HYP | 2.59749E-011* | 3.99E-12 | 0 | 1.80E-11 | 3.39E-11 |
|  | STEM | 8.97118E-012* | 3.87E-12 | 0.024 | 1.24E-12 | 1.67E-11 |
| STEM | STR | -1.46017E-011* | 3.99E-12 | 0.001 | -2.26E-11 | -6.63E-12 |
|  | HIPP | -1.09866E-011* | 3.87E-12 | 0.006 | -1.87E-11 | -3.25E-12 |
|  | CER | -9.95728E-012* | 3.87E-12 | 0.012 | -1.77E-11 | -2.22E-12 |
|  | THAL | -1.08254E-011* | 3.87E-12 | 0.007 | -1.86E-11 | -3.09E-12 |
|  | CTX | -1.20E-12 | 3.87E-12 | 0.757 | -8.93E-12 | 6.53E-12 |
|  | HYP | 1.70037E-011* | 3.99E-12 | 0 | 9.03E-12 | 2.50E-11 |
|  | MID | -8.97118E-012* | 3.87E-12 | 0.024 | -1.67E-11 | -1.24E-12 |

Supplemental Table 62: Descriptive statistics for CBD levels in 8 brain areas of NAPE-PLD KO animals

|  | N | Mean | Std. Deviation | Std. Error | 95% Confidence Interval for Mean | | Minimum | Maximum |
| --- | --- | --- | --- | --- | --- | --- | --- | --- |
|  |  |  |  |  | Lower Bound | Upper Bound | |  |
| STR | 8 | 4.73E-11 | 1.65E-11 | 5.83E-12 | 3.35E-11 | 6.10E-11 | 2.18E-11 | 7.45E-11 |
| HIPP | 8 | 3.88E-11 | 1.23E-11 | 4.35E-12 | 2.85E-11 | 4.90E-11 | 2.40E-11 | 5.75E-11 |
| CER | 8 | 8.71E-11 | 1.43E-11 | 5.04E-12 | 7.52E-11 | 9.90E-11 | 5.99E-11 | 1.04E-10 |
| THAL | 8 | 6.20E-11 | 1.18E-11 | 4.18E-12 | 5.21E-11 | 7.19E-11 | 4.56E-11 | 7.60E-11 |
| CTX | 8 | 7.67E-11 | 2.57E-11 | 9.09E-12 | 5.52E-11 | 9.82E-11 | 3.71E-11 | 1.11E-10 |
| HYP | 8 | 6.72E-12 | 9.92E-13 | 3.51E-13 | 5.89E-12 | 7.55E-12 | 5.50E-12 | 8.65E-12 |
| MID | 8 | 3.47E-11 | 5.99E-12 | 2.12E-12 | 2.97E-11 | 3.97E-11 | 2.82E-11 | 4.37E-11 |
| STEM | 8 | 8.97E-11 | 2.12E-11 | 7.48E-12 | 7.20E-11 | 1.07E-10 | 5.87E-11 | 1.14E-10 |
| Total | 64 | 5.54E-11 | 3.08E-11 | 3.86E-12 | 4.77E-11 | 6.31E-11 | 5.50E-12 | 1.14E-10 |

Supplemental Table 63: Results of ANOVA for NAPE-PLD KO animals to determine effect of brain region on levels of CBD

|  | Sum of Squares | df | Mean Square | F | Sig. |
| --- | --- | --- | --- | --- | --- |
| Between Groups | 4.65E-20 | 7 | 6.65E-21 | 27.817 | 4.68E-16 |
| Within Groups | 1.34E-20 | 56 | 2.39E-22 |  |  |
| Total | 5.99E-20 | 63 |  |  |  |

Supplemental Table 64: Results of post-hoc Fisher’s LSD for NAPE-PLD KO

| (I) Region | (J) Region | Mean Difference (I-J) | Std. Error | Sig. | 95% Confidence Interval | |
| --- | --- | --- | --- | --- | --- | --- |
|  |  |  |  |  | Lower Bound | Upper Bound |
| STR | HIPP | 8.51E-12 | 7.73E-12 | 0.276 | -6.98E-12 | 2.40E-11 |
|  | CER | -3.98353E-011* | 7.73E-12 | 0 | -5.53E-11 | -2.44E-11 |
|  | THAL | -1.47E-11 | 7.73E-12 | 0.062 | -3.02E-11 | 7.53E-13 |
|  | CTX | -2.94030E-011* | 7.73E-12 | 0 | -4.49E-11 | -1.39E-11 |
|  | HYP | 4.05372E-011* | 7.73E-12 | 0 | 2.51E-11 | 5.60E-11 |
|  | MID | 1.26E-11 | 7.73E-12 | 0.11 | -2.92E-12 | 2.80E-11 |
|  | STEM | -4.24317E-011* | 7.73E-12 | 0 | -5.79E-11 | -2.69E-11 |
| HIPP | STR | -8.51E-12 | 7.73E-12 | 0.276 | -2.40E-11 | 6.98E-12 |
|  | CER | -4.83442E-011* | 7.73E-12 | 0 | -6.38E-11 | -3.29E-11 |
|  | THAL | -2.32408E-011* | 7.73E-12 | 0.004 | -3.87E-11 | -7.76E-12 |
|  | CTX | -3.79119E-011* | 7.73E-12 | 0 | -5.34E-11 | -2.24E-11 |
|  | HYP | 3.20283E-011* | 7.73E-12 | 0 | 1.65E-11 | 4.75E-11 |
|  | MID | 4.06E-12 | 7.73E-12 | 0.602 | -1.14E-11 | 1.95E-11 |
|  | STEM | -5.09406E-011* | 7.73E-12 | 0 | -6.64E-11 | -3.55E-11 |
| CER | STR | 3.98353E-011* | 7.73E-12 | 0 | 2.44E-11 | 5.53E-11 |
|  | HIPP | 4.83442E-011* | 7.73E-12 | 0 | 3.29E-11 | 6.38E-11 |
|  | THAL | 2.51034E-011* | 7.73E-12 | 0.002 | 9.62E-12 | 4.06E-11 |
|  | CTX | 1.04E-11 | 7.73E-12 | 0.183 | -5.05E-12 | 2.59E-11 |
|  | HYP | 8.03725E-011* | 7.73E-12 | 0 | 6.49E-11 | 9.59E-11 |
|  | MID | 5.23992E-011* | 7.73E-12 | 0 | 3.69E-11 | 6.79E-11 |
|  | STEM | -2.60E-12 | 7.73E-12 | 0.738 | -1.81E-11 | 1.29E-11 |
| THAL | STR | 1.47E-11 | 7.73E-12 | 0.062 | -7.53E-13 | 3.02E-11 |
|  | HIPP | 2.32408E-011* | 7.73E-12 | 0.004 | 7.76E-12 | 3.87E-11 |
|  | CER | -2.51034E-011* | 7.73E-12 | 0.002 | -4.06E-11 | -9.62E-12 |
|  | CTX | -1.47E-11 | 7.73E-12 | 0.063 | -3.02E-11 | 8.14E-13 |
|  | HYP | 5.52691E-011* | 7.73E-12 | 0 | 3.98E-11 | 7.08E-11 |
|  | MID | 2.72958E-011* | 7.73E-12 | 0.001 | 1.18E-11 | 4.28E-11 |
|  | STEM | -2.76998E-011* | 7.73E-12 | 0.001 | -4.32E-11 | -1.22E-11 |
| CTX | STR | 2.94030E-011* | 7.73E-12 | 0 | 1.39E-11 | 4.49E-11 |
|  | HIPP | 3.79119E-011* | 7.73E-12 | 0 | 2.24E-11 | 5.34E-11 |
|  | CER | -1.04E-11 | 7.73E-12 | 0.183 | -2.59E-11 | 5.05E-12 |
|  | THAL | 1.47E-11 | 7.73E-12 | 0.063 | -8.14E-13 | 3.02E-11 |
|  | HYP | 6.99402E-011* | 7.73E-12 | 0 | 5.45E-11 | 8.54E-11 |
|  | MID | 4.19669E-011* | 7.73E-12 | 0 | 2.65E-11 | 5.75E-11 |
|  | STEM | -1.30E-11 | 7.73E-12 | 0.097 | -2.85E-11 | 2.46E-12 |
| HYP | STR | -4.05372E-011* | 7.73E-12 | 0 | -5.60E-11 | -2.51E-11 |
|  | HIPP | -3.20283E-011* | 7.73E-12 | 0 | -4.75E-11 | -1.65E-11 |
|  | CER | -8.03725E-011* | 7.73E-12 | 0 | -9.59E-11 | -6.49E-11 |
|  | THAL | -5.52691E-011* | 7.73E-12 | 0 | -7.08E-11 | -3.98E-11 |
|  | CTX | -6.99402E-011* | 7.73E-12 | 0 | -8.54E-11 | -5.45E-11 |
|  | MID | -2.79733E-011* | 7.73E-12 | 0.001 | -4.35E-11 | -1.25E-11 |
|  | STEM | -8.29689E-011* | 7.73E-12 | 0 | -9.85E-11 | -6.75E-11 |
| MID | STR | -1.26E-11 | 7.73E-12 | 0.11 | -2.80E-11 | 2.92E-12 |
|  | HIPP | -4.06E-12 | 7.73E-12 | 0.602 | -1.95E-11 | 1.14E-11 |
|  | CER | -5.23992E-011* | 7.73E-12 | 0 | -6.79E-11 | -3.69E-11 |
|  | THAL | -2.72958E-011* | 7.73E-12 | 0.001 | -4.28E-11 | -1.18E-11 |
|  | CTX | -4.19669E-011* | 7.73E-12 | 0 | -5.75E-11 | -2.65E-11 |
|  | HYP | 2.79733E-011* | 7.73E-12 | 0.001 | 1.25E-11 | 4.35E-11 |
|  | STEM | -5.49956E-011* | 7.73E-12 | 0 | -7.05E-11 | -3.95E-11 |
| STEM | STR | 4.24317E-011* | 7.73E-12 | 0 | 2.69E-11 | 5.79E-11 |
|  | HIPP | 5.09406E-011* | 7.73E-12 | 0 | 3.55E-11 | 6.64E-11 |
|  | CER | 2.60E-12 | 7.73E-12 | 0.738 | -1.29E-11 | 1.81E-11 |
|  | THAL | 2.76998E-011* | 7.73E-12 | 0.001 | 1.22E-11 | 4.32E-11 |
|  | CTX | 1.30E-11 | 7.73E-12 | 0.097 | -2.46E-12 | 2.85E-11 |
|  | HYP | 8.29689E-011* | 7.73E-12 | 0 | 6.75E-11 | 9.85E-11 |
|  | MID | 5.49956E-011* | 7.73E-12 | 0 | 3.95E-11 | 7.05E-11 |
